# Supplementary material for: Application of Pattern Mining Methods to Assess Exposures to Multiple Airborne Chemical Agents in Two Large Occupational Exposure Databases from France
Source: Int J Environ Res Public Health. 2022 Feb 3;19(3):1746. doi: 10.3390/ijerph19031746 (PMC8835122; doi:10.3390/ijerph19031746)
Supplement: Supplementary file 1 [file ijerph-19-01746-s001.zip › ijerph-1540532-supplementary.html]

Application of pattern mining methods to assess exposures to multiple airborne chemical agents in two large French occupational exposure databases


# Application of pattern mining methods to assess exposures to multiple airborne chemical agents in two large French occupational exposure databases

#### Supplementary tables

#### January 27, 2022

**Jean-François Sauvé 1, Andrea Emili 1, Gautier Mater 1**

1 Pollutants Metrology Department, Institut National de Recherche et de Sécurité (INRS), Vandoeuvre-lès-Nancy, France

# 

## Table S1: List of agents

List of agents included in the analysis, IARC carcinogencity classification, number of records, number of work situations, percentage of work situations with coexposure to at least another agent, industry sectors with the most coexposure

| Category | Family | Agent | IARC | N records | N WS | % Coex | Industries with most coexposures |
| --- | --- | --- | --- | --- | --- | --- | --- |
| Acids | Mineral acids | Hydrogen chloride | 3 | 1324 | 565 | 66.0% | Manufacture of chemicals and chemical products (N=89); Manufacture of fabricated metal products, except machinery and equipment (N=34); Wholesale trade, except of motor vehicles and motorcycles (N=27) |
|  |  | Hydrogen fluoride | — | 1038 | 299 | 57.5% | Manufacture of basic metals (N=39); Manufacture of fabricated metal products, except machinery and equipment (N=20); Manufacture of computer, electronic and optical products (N=17) |
|  |  | Nitric acid | — | 324 | 178 | 74.7% | Manufacture of chemicals and chemical products (N=33); Manufacture of fabricated metal products, except machinery and equipment (N=13); Manufacture of electrical equipment (N=11) |
|  |  | Orthophosphoric acid | — | 163 | 81 | 76.5% | Manufacture of fabricated metal products, except machinery and equipment (N=15); Manufacture of electrical equipment (N= 8); Manufacture of chemicals and chemical products (N= 5) |
|  |  | Peracetic acid | — | 107 | 44 | 36.4% | Human health activities (N=12); Crop and animal production, hunting and related service activities (N= 1); Manufacture of beverages (N= 1) |
|  |  | Sulphuric acid | — | 756 | 324 | 70.7% | Waste collection, treatment and disposal activities: materials recovery (N=39); Manufacture of chemicals and chemical products (N=30); Manufacture of fabricated metal products, except machinery and equipment (N=24) |
| Agents not elsewhere classified | Agents not elsewhere classified | Bisphenol A | — | 108 | 24 | 37.5% | Manufacture of chemicals and chemical products (N=2); Civil engineering (N=1); Electricity, gas, steam and air conditioning supply (N=1) |
| Alkali and alkaline earth metals | Barium | Barium | — | 324 | 127 | 96.9% | Manufacture of fabricated metal products, except machinery and equipment (N=35); Waste collection, treatment and disposal activities: materials recovery (N=16); Manufacture of chemicals and chemical products (N=11) |
|  | Magnesium | Magnesium | — | 276 | 103 | 100.0% | Manufacture of basic metals (N=17); Manufacture of fabricated metal products, except machinery and equipment (N=16); Waste collection, treatment and disposal activities: materials recovery (N= 9) |
| Dusts | Inhalable dust | Metalworking fluids (inhalable fraction) | — | 1125 | 200 | 79.0% | Manufacture of fabricated metal products, except machinery and equipment (N=60); Manufacture of machinery and equipment n.e.c. (N=29); Manufacture of basic metals (N=16) |
|  |  | Welding fumes\* | 1 | 1021 | 141 | 94.3% | Manufacture of fabricated metal products, except machinery and equipment (N=43); Manufacture of machinery and equipment n.e.c. (N=20); Manufacture of other transport equipment (N=11) |
|  | Wood dust | Wood dust\* | 1 | 53137 | 7809 | 6.0% | Manufacture of wood and of products of wood and cork, except furniture: manufacture of articles of straw and plaiting materials (N=90); Waste collection, treatment and disposal activities: materials recovery (N=78); Electricity, gas, steam and air conditioning supply (N=32) |
| Fibers | Asbestos fibers | Asbestos\* | 1 | 5144 | 237 | 8.0% | Specialised construction activities (N=7); Land transport and transport via pipelines (N=6); Civil engineering (N=1) |
|  | Ceramic fibers | Refractory ceramic fibers (L>5um D<3um)\* | 2B | 2622 | 831 | 35.3% | Manufacture of basic metals (N=101); Manufacture of other non-metallic mineral products (N= 94); Manufacture of fabricated metal products, except machinery and equipment (N= 19) |
|  | Fibres | Fibres (L>5um D<3um) | — | 228 | 71 | 36.6% | Specialised construction activities (N=5); Manufacture of fabricated metal products, except machinery and equipment (N=4); Manufacture of basic metals (N=3) |
| Halogens | Chlorine derivatives | Chlorine | — | 226 | 84 | 56.0% | Manufacture of chemicals and chemical products (N=31); Manufacture of basic metals (N= 6); Wholesale trade, except of motor vehicles and motorcycles (N= 3) |
|  | Fluorine derivatives | Fluorides | — | 187 | 67 | 46.3% | Manufacture of basic metals (N=9); Manufacture of machinery and equipment n.e.c. (N=7); Manufacture of fabricated metal products, except machinery and equipment (N=4) |
| Metals | Aluminum | Aluminium | — | 1619 | 496 | 95.4% | Manufacture of fabricated metal products, except machinery and equipment (N=101); Waste collection, treatment and disposal activities: materials recovery (N= 54); Manufacture of basic metals (N= 50) |
|  | Boron | Boron | — | 208 | 62 | 98.4% | Manufacture of fabricated metal products, except machinery and equipment (N=20); Manufacture of machinery and equipment n.e.c. (N= 8); Manufacture of motor vehicles, trailers and semi-trailers (N= 5) |
|  | Cadmium | Cadmium\* | 1 | 181 | 66 | 95.5% | Waste collection, treatment and disposal activities: materials recovery (N=17); Manufacture of fabricated metal products, except machinery and equipment (N=10); Manufacture of basic metals (N= 7) |
|  | Chromium | Chromium (metal and inorganic Cr(II)/Cr(III) compounds) | 3 | 3130 | 904 | 79.0% | Manufacture of basic metals (N=170); Manufacture of fabricated metal products, except machinery and equipment (N=109); Waste collection, treatment and disposal activities: materials recovery (N= 83) |
|  |  | Hexavalent chromium\* | 1 | 8604 | 2024 | 58.0% | Manufacture of fabricated metal products, except machinery and equipment (N=178); Manufacture of basic metals (N=167); Waste collection, treatment and disposal activities: materials recovery (N=157) |
|  |  | Total chromium | 3 | 1754 | 473 | 100.0% | Manufacture of fabricated metal products, except machinery and equipment (N=138); Manufacture of machinery and equipment n.e.c. (N= 51); Repair and installation of machinery and equipment (N= 50) |
|  | Cobalt | Cobalt\* | 2B | 583 | 186 | 90.9% | Manufacture of fabricated metal products, except machinery and equipment (N=53); Manufacture of machinery and equipment n.e.c. (N=27); Other manufacturing (N=16) |
|  | Copper | Copper | — | 2968 | 661 | 98.5% | Manufacture of fabricated metal products, except machinery and equipment (N=160); Manufacture of machinery and equipment n.e.c. (N= 80); Manufacture of basic metals (N= 59) |
|  | Iron | Diiron trioxide | 3 | 346 | 55 | 100.0% | Manufacture of fabricated metal products, except machinery and equipment (N=19); Manufacture of machinery and equipment n.e.c. (N=11); Manufacture of motor vehicles, trailers and semi-trailers (N= 8) |
|  |  | Iron | — | 5724 | 1171 | 97.0% | Manufacture of fabricated metal products, except machinery and equipment (N=278); Manufacture of machinery and equipment n.e.c. (N=108); Specialised construction activities (N= 94) |
|  | Lead | Lead\* | 2B | 13287 | 3708 | 36.4% | Waste collection, treatment and disposal activities: materials recovery (N=316); Manufacture of basic metals (N=262); Manufacture of fabricated metal products, except machinery and equipment (N=110) |
|  | Manganese | Manganese | — | 4920 | 840 | 99.8% | Manufacture of fabricated metal products, except machinery and equipment (N=215); Manufacture of machinery and equipment n.e.c. (N= 92); Repair and installation of machinery and equipment (N= 65) |
|  | Mercury | Mercury | 3 | 833 | 219 | 56.6% | Waste collection, treatment and disposal activities: materials recovery (N=68); Scientific research and development (N= 9); Manufacture of chemicals and chemical products (N= 7) |
|  | Molybdenum | Molybdenum | — | 259 | 106 | 100.0% | Manufacture of fabricated metal products, except machinery and equipment (N=40); Repair and installation of machinery and equipment (N=17); Manufacture of machinery and equipment n.e.c. (N=13) |
|  | Nickel | Nickel\* | 1 | 2519 | 614 | 98.2% | Manufacture of fabricated metal products, except machinery and equipment (N=153); Manufacture of machinery and equipment n.e.c. (N= 74); Repair and installation of machinery and equipment (N= 51) |
|  | Silicium | Cristobalite\* | 1 | 4199 | 1540 | 94.4% | Manufacture of other non-metallic mineral products (N=556); Other mining and quarrying (N=247); Manufacture of basic metals (N=179) |
|  |  | Quartz\* | 1 | 32566 | 6443 | 36.8% | Manufacture of other non-metallic mineral products (N=812); Manufacture of basic metals (N=312); Other mining and quarrying (N=276) |
|  |  | Tridymite | — | 1551 | 473 | 78.4% | Manufacture of other non-metallic mineral products (N=203); Other mining and quarrying (N= 53); Manufacture of chemicals and chemical products (N= 23) |
|  | Silver | Silver | — | 110 | 46 | 54.3% | Manufacture of fabricated metal products, except machinery and equipment (N=6); Manufacture of electrical equipment (N=4); Land transport and transport via pipelines (N=3) |
|  | Tin | Tin | — | 140 | 73 | 97.3% | Manufacture of machinery and equipment n.e.c. (N=15); Manufacture of basic metals (N= 9); Manufacture of fabricated metal products, except machinery and equipment (N= 9) |
|  | Titanium | Titanium | — | 811 | 278 | 98.2% | Manufacture of fabricated metal products, except machinery and equipment (N=65); Waste collection, treatment and disposal activities: materials recovery (N=24); Repair and installation of machinery and equipment (N=21) |
|  | Tungsten | Tungsten | — | 188 | 62 | 100.0% | Manufacture of fabricated metal products, except machinery and equipment (N=26); Repair and installation of machinery and equipment (N= 9); Manufacture of machinery and equipment n.e.c. (N= 8) |
|  | Zinc | Zinc | — | 2697 | 710 | 99.3% | Manufacture of fabricated metal products, except machinery and equipment (N=165); Manufacture of machinery and equipment n.e.c. (N= 69); Specialised construction activities (N= 60) |
| Other categories | Carbon | Bitumen fumes\* | 2A/2B | 305 | 41 | 26.8% | Civil engineering (N=9); Manufacture of paper and paper products (N=1); Specialised construction activities (N=1) |
|  |  | Diesel exhaust, elemental carbon\* | 1 | 330 | 138 | 40.6% | Waste collection, treatment and disposal activities: materials recovery (N=14); Civil engineering (N= 7); Specialised construction activities (N= 6) |
|  | Gases | Carbon dioxide | — | 156 | 54 | 53.7% | Land transport and transport via pipelines (N=10); Other mining and quarrying (N= 6); Manufacture of computer, electronic and optical products (N= 2) |
|  |  | Hydrogen sulphide | — | 366 | 149 | 61.7% | Sewerage (N=15); Waste collection, treatment and disposal activities: materials recovery (N=15); Manufacture of chemicals and chemical products (N=12) |
|  | Nitrogen derivatives | Ammonia, anhydrous | — | 5307 | 1408 | 55.0% | Manufacture of chemicals and chemical products (N=164); Waste collection, treatment and disposal activities: materials recovery (N=156); Manufacture of other non-metallic mineral products (N=112) |
| Volatile organic compounds | Alcohols | 2-Methylpropan-1-ol | — | 172 | 65 | 100.0% | Manufacture of chemicals and chemical products (N=16); Manufacture of fabricated metal products, except machinery and equipment (N=13); Manufacture of furniture (N= 7) |
|  |  | Butan-1-ol | — | 227 | 73 | 100.0% | Manufacture of chemicals and chemical products (N=17); Manufacture of fabricated metal products, except machinery and equipment (N= 9); Wholesale and retail trade and repair of motor vehicles and motorcycles (N= 7) |
|  |  | Ethanol\* | 1 | 1536 | 414 | 94.0% | Manufacture of rubber and plastic products (N=74); Human health activities (N=58); Manufacture of chemicals and chemical products (N=31) |
|  |  | Methanol | — | 1389 | 461 | 78.5% | Manufacture of chemicals and chemical products (N=56); Manufacture of basic pharmaceutical products and pharmaceutical preparations (N=39); Manufacture of rubber and plastic products (N=25) |
|  |  | Propan-2-ol | 3 | 1084 | 299 | 97.3% | Manufacture of rubber and plastic products (N=51); Manufacture of chemicals and chemical products (N=28); Printing and reproduction of recorded media (N=28) |
|  | Aldehydes | Acetaldehyde\* | 2B | 377 | 136 | 98.5% | Manufacture of rubber and plastic products (N=26); Waste collection, treatment and disposal activities: materials recovery (N=15); Manufacture of food products (N=11) |
|  |  | Formaldehyde\* | 1 | 2413 | 542 | 61.3% | Human health activities (N=53); Manufacture of rubber and plastic products (N=49); Manufacture of basic metals (N=28) |
|  | Aliphatic and cycloaliphatic hydrocarbons | 2-Methylbutane | — | 261 | 77 | 87.0% | Manufacture of rubber and plastic products (N=24); Manufacture of coke and refined petroleum products (N=15); Warehousing and support activities for transportation (N= 8) |
|  |  | Aliphatic and alicyclic hydrocarbons | — | 1940 | 441 | 94.8% | Manufacture of rubber and plastic products (N=77); Manufacture of fabricated metal products, except machinery and equipment (N=36); Manufacture of chemicals and chemical products (N=24) |
|  |  | C6-C12 hydrocarbons | — | 829 | 244 | 94.7% | Manufacture of rubber and plastic products (N=43); Manufacture of chemicals and chemical products (N=27); Manufacture of fabricated metal products, except machinery and equipment (N=25) |
|  |  | Cyclohexane | — | 1833 | 612 | 94.1% | Manufacture of rubber and plastic products (N=76); Waste collection, treatment and disposal activities: materials recovery (N=72); Manufacture of chemicals and chemical products (N=62) |
|  |  | Heptane | — | 2259 | 701 | 87.0% | Waste collection, treatment and disposal activities: materials recovery (N=78); Manufacture of chemicals and chemical products (N=67); Manufacture of rubber and plastic products (N=60) |
|  |  | N-hexane | — | 3705 | 1040 | 90.3% | Manufacture of chemicals and chemical products (N=107); Waste collection, treatment and disposal activities: materials recovery (N=102); Manufacture of rubber and plastic products (N= 89) |
|  |  | Pentane | — | 1217 | 487 | 89.3% | Waste collection, treatment and disposal activities: materials recovery (N=80); Manufacture of rubber and plastic products (N=55); Manufacture of coke and refined petroleum products (N=54) |
|  | Amides | 1-Methyl-2-pyrrolidone | — | 269 | 79 | 53.2% | Manufacture of fabricated metal products, except machinery and equipment (N=13); Manufacture of chemicals and chemical products (N=10); Architectural and engineering activities: technical testing and analysis (N= 2) |
|  |  | N,N-dimethylacetamide\* | 2B | 110 | 41 | 61.0% | Manufacture of basic pharmaceutical products and pharmaceutical preparations (N=8); Manufacture of chemicals and chemical products (N=5); Waste collection, treatment and disposal activities: materials recovery (N=5) |
|  |  | N,N-Dimethylformamide\* | 2A | 322 | 105 | 68.6% | Manufacture of chemicals and chemical products (N=28); Manufacture of basic pharmaceutical products and pharmaceutical preparations (N=11); Scientific research and development (N= 7) |
|  | Amines | 2-Aminoethanol | — | 330 | 87 | 54.0% | Manufacture of machinery and equipment n.e.c. (N=11); Manufacture of basic metals (N= 8); Manufacture of motor vehicles, trailers and semi-trailers (N= 6) |
|  |  | Triethylamine | — | 103 | 35 | 74.3% | Manufacture of chemicals and chemical products (N=8); Manufacture of fabricated metal products, except machinery and equipment (N=7); Manufacture of basic pharmaceutical products and pharmaceutical preparations (N=5) |
|  | Anesthetics | Dinitrogen oxide | — | 338 | 67 | 46.3% | Human health activities (N=25); Public administration and defence: compulsory social security (N= 3); Residential care activities (N= 2) |
|  |  | Isoflurane | — | 131 | 17 | 76.5% | Veterinary activities (N=8); Architectural and engineering activities: technical testing and analysis (N=2); Scientific research and development (N=2) |
|  |  | Sevoflurane | — | 191 | 43 | 86.0% | Human health activities (N=30); Public administration and defence: compulsory social security (N= 4); Architectural and engineering activities: technical testing and analysis (N= 2) |
|  | Aromatic hydrocarbons | 1,2,3-Trimethylbenzene | — | 490 | 198 | 99.5% | Manufacture of chemicals and chemical products (N=26); Waste collection, treatment and disposal activities: materials recovery (N=21); Manufacture of basic metals (N=19) |
|  |  | 1,2,4-Trimethylbenzene | — | 2152 | 670 | 96.0% | Manufacture of chemicals and chemical products (N=92); Waste collection, treatment and disposal activities: materials recovery (N=88); Manufacture of fabricated metal products, except machinery and equipment (N=41) |
|  |  | 2-Phenylpropene | — | 291 | 35 | 97.1% | Manufacture of rubber and plastic products (N=12); Manufacture of other transport equipment (N= 9); Manufacture of chemicals and chemical products (N= 4) |
|  |  | Benzene\* | 1 | 4849 | 1209 | 65.7% | Manufacture of coke and refined petroleum products (N=170); Waste collection, treatment and disposal activities: materials recovery (N=100); Manufacture of chemicals and chemical products (N= 99) |
|  |  | C9-C12 benzenic hydrocarbons | — | 273 | 100 | 94.0% | Manufacture of rubber and plastic products (N=16); Manufacture of fabricated metal products, except machinery and equipment (N=11); Manufacture of electrical equipment (N= 7) |
|  |  | Cumene\* | 2B | 385 | 143 | 98.6% | Manufacture of chemicals and chemical products (N=40); Manufacture of fabricated metal products, except machinery and equipment (N=12); Manufacture of basic metals (N=11) |
|  |  | Ethylbenzene\* | 2B | 5712 | 1657 | 98.9% | Manufacture of chemicals and chemical products (N=180); Waste collection, treatment and disposal activities: materials recovery (N=172); Manufacture of rubber and plastic products (N=108) |
|  |  | Mesitylene | — | 897 | 312 | 99.0% | Manufacture of chemicals and chemical products (N=43); Manufacture of machinery and equipment n.e.c. (N=36); Manufacture of fabricated metal products, except machinery and equipment (N=21) |
|  |  | Monocyclic aromatic hydrocarbons | — | 527 | 118 | 97.5% | Manufacture of fabricated metal products, except machinery and equipment (N=15); Manufacture of rubber and plastic products (N=14); Manufacture of chemicals and chemical products (N=12) |
|  |  | Styrene\* | 2B | 5134 | 619 | 82.6% | Manufacture of rubber and plastic products (N=144); Manufacture of chemicals and chemical products (N= 62); Manufacture of other transport equipment (N= 62) |
|  |  | Toluene | 3 | 10826 | 2714 | 87.0% | Manufacture of chemicals and chemical products (N=307); Waste collection, treatment and disposal activities: materials recovery (N=221); Manufacture of rubber and plastic products (N=172) |
|  |  | Trimethylbenzene | — | 241 | 69 | 100.0% | Manufacture of chemicals and chemical products (N=20); Printing and reproduction of recorded media (N=14); Manufacture of fabricated metal products, except machinery and equipment (N= 6) |
|  |  | Xylene | 3 | 10163 | 2517 | 90.2% | Manufacture of chemicals and chemical products (N=265); Waste collection, treatment and disposal activities: materials recovery (N=206); Manufacture of rubber and plastic products (N=147) |
|  | Esters | Butyl acrylate | 3 | 100 | 29 | 89.7% | Manufacture of chemicals and chemical products (N=18); Civil engineering (N= 3); Construction of buildings (N= 1) |
|  |  | Ethyl acetate | — | 1704 | 406 | 99.3% | Manufacture of rubber and plastic products (N=93); Manufacture of chemicals and chemical products (N=49); Printing and reproduction of recorded media (N=26) |
|  |  | Ethyl methacrylate | — | 103 | 7 | 100.0% | Other personal service activities (N=7) |
|  |  | Isopropyl acetate | — | 240 | 70 | 100.0% | Manufacture of rubber and plastic products (N=20); Printing and reproduction of recorded media (N=13); Manufacture of chemicals and chemical products (N=11) |
|  |  | Methyl methacrylate | 3 | 1294 | 265 | 83.0% | Manufacture of rubber and plastic products (N=41); Manufacture of chemicals and chemical products (N=36); Manufacture of other transport equipment (N=33) |
|  |  | N-butyl acetate | — | 1129 | 312 | 99.7% | Manufacture of chemicals and chemical products (N=44); Manufacture of fabricated metal products, except machinery and equipment (N=31); Manufacture of rubber and plastic products (N=31) |
|  |  | Propyl acetate | — | 219 | 53 | 100.0% | Manufacture of rubber and plastic products (N=22); Manufacture of paper and paper products (N= 9); Printing and reproduction of recorded media (N= 7) |
|  |  | Vinyl acetate\* | 2B | 304 | 94 | 70.2% | Manufacture of chemicals and chemical products (N=49); Unclassified (N= 7); Manufacture of rubber and plastic products (N= 3) |
|  | Ether oxides | 1,4-Dioxane\* | 2B | 105 | 24 | 87.5% | Manufacture of basic pharmaceutical products and pharmaceutical preparations (N=13); Manufacture of chemicals and chemical products (N= 5); Scientific research and development (N= 2) |
|  |  | Diethyl ether | — | 360 | 101 | 93.1% | Manufacture of food products (N=19); Manufacture of chemicals and chemical products (N=12); Manufacture of basic pharmaceutical products and pharmaceutical preparations (N= 8) |
|  |  | Tetrahydrofuran\* | 2B | 784 | 233 | 83.3% | Manufacture of chemicals and chemical products (N=26); Manufacture of basic pharmaceutical products and pharmaceutical preparations (N=23); Waste collection, treatment and disposal activities: materials recovery (N=21) |
|  | Gasoline, solvents, petroleum | Water-soluble metalworking fluids | — | 745 | 144 | 97.2% | Manufacture of fabricated metal products, except machinery and equipment (N=55); Manufacture of machinery and equipment n.e.c. (N=28); Manufacture of basic metals (N=13) |
|  | Glycol ethers derived from ethylene glycol | 2-Butoxyethanol | 3 | 627 | 238 | 76.1% | Manufacture of chemicals and chemical products (N=34); Manufacture of fabricated metal products, except machinery and equipment (N=26); Manufacture of basic metals (N=19) |
|  |  | 2-Butoxyethyl acetate | — | 431 | 156 | 78.2% | Financial service activities, except insurance and pension funding (N=23); Manufacture of rubber and plastic products (N=14); Manufacture of fabricated metal products, except machinery and equipment (N=13) |
|  | Glycol ethers derived from propylene glycol | (2-Methoxymethylethoxy)propanol | — | 186 | 82 | 79.3% | Manufacture of fabricated metal products, except machinery and equipment (N=14); Manufacture of chemicals and chemical products (N=12); Manufacture of computer, electronic and optical products (N= 5) |
|  |  | 1-Ethoxypropan-2-ol | — | 111 | 29 | 100.0% | Manufacture of rubber and plastic products (N=17); Printing and reproduction of recorded media (N= 5); Architectural and engineering activities: technical testing and analysis (N= 2) |
|  |  | 1-Methoxypropan-2-ol | — | 3123 | 815 | 83.6% | Manufacture of other transport equipment (N=90); Manufacture of chemicals and chemical products (N=78); Manufacture of fabricated metal products, except machinery and equipment (N=73) |
|  |  | 2-Methoxy-1-methylethyl acetate | — | 2553 | 678 | 95.4% | Manufacture of other transport equipment (N=110); Manufacture of fabricated metal products, except machinery and equipment (N= 82); Manufacture of chemicals and chemical products (N= 71) |
|  | Halogenated compounds | Chlorobenzene | — | 159 | 68 | 79.4% | Manufacture of chemicals and chemical products (N=19); Waste collection, treatment and disposal activities: materials recovery (N=14); Architectural and engineering activities: technical testing and analysis (N= 5) |
|  |  | Chloroethylene\* | 1 | 1077 | 243 | 56.4% | Manufacture of chemicals and chemical products (N=83); Manufacture of coke and refined petroleum products (N=28); Waste collection, treatment and disposal activities: materials recovery (N= 6) |
|  |  | Chloroform\* | 2B | 1278 | 331 | 79.2% | Manufacture of chemicals and chemical products (N=76); Waste collection, treatment and disposal activities: materials recovery (N=40); Manufacture of basic pharmaceutical products and pharmaceutical preparations (N=24) |
|  |  | Dichloromethane\* | 2A | 1793 | 486 | 73.3% | Waste collection, treatment and disposal activities: materials recovery (N=59); Manufacture of basic pharmaceutical products and pharmaceutical preparations (N=43); Manufacture of chemicals and chemical products (N=38) |
|  |  | Tetrachloroethylene\* | 2A | 1460 | 385 | 48.3% | Manufacture of fabricated metal products, except machinery and equipment (N=35); Waste collection, treatment and disposal activities: materials recovery (N=19); Architectural and engineering activities: technical testing and analysis (N=13) |
|  | Ketones | 4-Hydroxy-4-methylpentan-2-one | — | 174 | 39 | 100.0% | Manufacture of rubber and plastic products (N=10); Printing and reproduction of recorded media (N= 8); Manufacture of machinery and equipment n.e.c. (N= 3) |
|  |  | 4-Methylpentan-2-one\* | 2B | 1957 | 577 | 90.6% | Manufacture of chemicals and chemical products (N=74); Manufacture of other transport equipment (N=52); Manufacture of fabricated metal products, except machinery and equipment (N=42) |
|  |  | Acetone | — | 12798 | 2681 | 74.1% | Manufacture of chemicals and chemical products (N=228); Manufacture of rubber and plastic products (N=206); Manufacture of other transport equipment (N=182) |
|  |  | Butanone | — | 8763 | 2109 | 70.9% | Manufacture of rubber and plastic products (N=187); Manufacture of chemicals and chemical products (N=163); Manufacture of other transport equipment (N=150) |
|  |  | Cyclohexanone | 3 | 925 | 284 | 75.7% | Manufacture of rubber and plastic products (N=43); Manufacture of chemicals and chemical products (N=31); Manufacture of electrical equipment (N=23) |
|  | Other nitrogen compounds | Acetonitrile | — | 655 | 219 | 89.0% | Manufacture of basic pharmaceutical products and pharmaceutical preparations (N=35); Manufacture of chemicals and chemical products (N=33); Scientific research and development (N=20) |
|  | Phenols | Phenol | 3 | 1652 | 521 | 62.8% | Manufacture of other non-metallic mineral products (N=82); Manufacture of chemicals and chemical products (N=56); Manufacture of basic metals (N=51) |
|  | Polycyclic aromatic hydrocarbons | Benz[a]anthracene\* | 2B | 247 | 76 | 100.0% | Manufacture of basic metals (N=15); Manufacture of wood and of products of wood and cork, except furniture: manufacture of articles of straw and plaiting materials (N=15); Manufacture of fabricated metal products, except machinery and equipment (N= 8) |
|  |  | Benzo(e)acephenanthrylene\* | 2B | 232 | 80 | 97.5% | Manufacture of basic metals (N=16); Manufacture of wood and of products of wood and cork, except furniture: manufacture of articles of straw and plaiting materials (N=15); Repair and installation of machinery and equipment (N= 8) |
|  |  | Benzo(k)fluoranthene\* | 2B | 238 | 74 | 100.0% | Manufacture of basic metals (N=15); Manufacture of wood and of products of wood and cork, except furniture: manufacture of articles of straw and plaiting materials (N=15); Repair and installation of machinery and equipment (N= 7) |
|  |  | Benzo[def]chrysene\* | 1 | 290 | 95 | 95.8% | Manufacture of basic metals (N=16); Manufacture of wood and of products of wood and cork, except furniture: manufacture of articles of straw and plaiting materials (N=15); Manufacture of fabricated metal products, except machinery and equipment (N= 8) |
|  |  | Benzo[ghi]perylene | 3 | 219 | 64 | 100.0% | Manufacture of wood and of products of wood and cork, except furniture: manufacture of articles of straw and plaiting materials (N=15); Manufacture of basic metals (N=13); Repair and installation of machinery and equipment (N= 6) |
|  |  | Indeno[1,2,3-cd]pyrene\* | 2B | 135 | 39 | 100.0% | Manufacture of wood and of products of wood and cork, except furniture: manufacture of articles of straw and plaiting materials (N=15); Manufacture of basic metals (N= 9); Manufacture of fabricated metal products, except machinery and equipment (N= 5) |
|  |  | Pyrene | 3 | 162 | 59 | 84.7% | Manufacture of basic metals (N=11); Manufacture of wood and of products of wood and cork, except furniture: manufacture of articles of straw and plaiting materials (N= 7); Repair and installation of machinery and equipment (N= 5) |
|  | Terpenes | D-Limonene | — | 134 | 5 | 100.0% | Waste collection, treatment and disposal activities: materials recovery (N=4); Manufacture of chemicals and chemical products (N=1) |

## Table S2: List of industry sectors, all agents

List of industry sectors with exposure to at least one agent, number of WS, percentage of WS with coexposure, most frequent agents identified in WS with coexposure (number of WS in parentheses), and most frequent mixture of agents across WS (number of WS in parentheses)

| Division | Group | N WS | % Coex | Most frequent agents | Most frequent mixture |
| --- | --- | --- | --- | --- | --- |
| 01: Crop and animal production, hunting and related service activities | 011: Growing of non-perennial crops | 16 | 25% | Quartz (N=2)/ Wood dust (N=2)/ Acetone (N=1)/ Acetonitrile (N=1)/ Iron (N=1) | Quartz,Wood dust (N=2) |
|  | 012: Growing of perennial crops | 12 | 8.3% | Dinitrogen oxide (N=1)/ Formaldehyde (N=1)/ Peracetic acid (N=1) | Dinitrogen oxide,Formaldehyde,Peracetic acid (N=1) |
|  | 013: Plant propagation | 2 | 50% | Quartz (N=1)/ Tridymite (N=1) | Quartz,Tridymite (N=1) |
|  | 014: Animal production | 3 | 0% |  |  |
|  | 015: Mixed farming | 2 | 50% | Chromium (metal and inorganic Cr(II)/Cr(III) compounds) (N=1)/ Copper (N=1)/ Diiron trioxide (N=1)/ Manganese (N=1)/ Nickel (N=1) | Chromium (metal and inorganic Cr(II)/Cr(III) compounds),Copper,Diiron trioxide,Manganese,Nickel,Welding fumes (N=1) |
|  | 016: Support activities to agriculture and post-harvest crop activities | 4 | 0% |  |  |
| 02: Forestry and logging | 021: Silviculture and other forestry activities | 3 | 0% |  |  |
|  | 022: Logging | 167 | 0% |  |  |
|  | 024: Support services to forestry | 51 | 2% | 2-Aminoethanol (N=1)/ N-hexane (N=1)/ Toluene (N=1)/ Wood dust (N=1) | 2-Aminoethanol,N-hexane,Toluene,Wood dust (N=1) |
| 03: Fishing and aquaculture | 031: Fishing | 2 | 0% |  |  |
| 06: Extraction of crude petroleum and natural gas | 062: Extraction of natural gas | 2 | 50% | Benzene (N=1)/ Toluene (N=1) | Benzene,Toluene (N=1) |
| 07: Mining of metal ores | 072: Mining of non-ferrous metal ores | 7 | 0% |  |  |
| 08: Other mining and quarrying | 081: Quarrying of stone, sand and clay | 1380 | 19.9% | Quartz (N=270)/ Cristobalite (N=243)/ Tridymite (N= 53)/ Lead (N= 9)/ Carbon dioxide (N= 6) | Cristobalite,Quartz (N=243) |
|  | 089: Mining and quarrying n.e.c. | 82 | 13.4% | Quartz (N=6)/ Cristobalite (N=4)/ Ammonia, anhydrous (N=2)/ Chloroethylene (N=2)/ Chromium (metal and inorganic Cr(II)/Cr(III) compounds) (N=2) | Cristobalite,Quartz (N=4) |
| 09: Mining support service activities | 091: Support activities for petroleum and natural gas extraction | 4 | 75% | Benzene (N=2)/ Cyclohexane (N=2)/ Ethylbenzene (N=2)/ Heptane (N=2)/ N-hexane (N=2) | Benzene,Cyclohexane,Ethylbenzene,Heptane,N-hexane,Toluene,Xylene (N=2) |
|  | 099: Support activities for other mining and quarrying | 2 | 0% |  |  |
| 10: Manufacture of food products | 101: Processing and preserving of meat and production of meat products | 28 | 39.3% | Formaldehyde (N=8)/ Acetaldehyde (N=7)/ Ammonia, anhydrous (N=2)/ Benz[a]anthracene (N=2)/ Benzo(e)acephenanthrylene (N=1) | Acetaldehyde,Formaldehyde (N=6) |
|  | 102: Processing and preserving of fish, crustaceans and molluscs | 4 | 75% | Acetaldehyde (N=1)/ Aliphatic and alicyclic hydrocarbons (N=1)/ Benz[a]anthracene (N=1)/ Benzo(k)fluoranthene (N=1)/ Benzo[def]chrysene (N=1) | Acetaldehyde,Formaldehyde (N=1) |
|  | 103: Processing and preserving of fruit and vegetables | 14 | 14.3% | Hydrogen fluoride (N=1)/ Metalworking fluids (inhalable fraction) (N=1)/ Nitric acid (N=1)/ Water-soluble metalworking fluids (N=1) | Hydrogen fluoride,Nitric acid (N=1) |
|  | 104: Manufacture of vegetable and animal oils and fats | 50 | 70% | N-hexane (N=26)/ Diethyl ether (N=12)/ Ethanol (N=11)/ Aliphatic and alicyclic hydrocarbons (N= 9)/ Acetone (N= 7) | Diethyl ether,N-hexane (N=10) |
|  | 105: Manufacture of dairy products | 39 | 20.5% | N-hexane (N=4)/ Diethyl ether (N=3)/ Orthophosphoric acid (N=2)/ Sulphuric acid (N=2)/ Ammonia, anhydrous (N=1) | Diethyl ether,N-hexane (N=3) |
|  | 106: Manufacture of grain mill products, starches and starch products | 18 | 50% | Acetone (N=4)/ Chloroform (N=4)/ Cristobalite (N=4)/ N-hexane (N=4)/ Quartz (N=4) | Acetone,Chloroform (N=4) |
|  | 107: Manufacture of bakery and farinaceous products | 34 | 50% | Butanone (N=11)/ Acetone (N= 4)/ Pentane (N= 4)/ N-hexane (N= 3)/ Acetaldehyde (N= 2) | Acetone,Butanone (N=4) |
|  | 108: Manufacture of other food products | 62 | 21% | N-hexane (N=5)/ Acetone (N=3)/ Butanone (N=3)/ Toluene (N=3)/ Aliphatic and alicyclic hydrocarbons (N=2) | Acetone,N-hexane (N=2) |
|  | 109: Manufacture of prepared animal feeds | 56 | 44.6% | Manganese (N=20)/ Zinc (N=17)/ Iron (N=11)/ Cobalt (N= 8)/ Copper (N= 6) | Manganese,Zinc (N=17) |
| 11: Manufacture of beverages | 110: Manufacture of beverages | 104 | 26.9% | Butanone (N=10)/ Xylene (N= 7)/ 2-Butoxyethanol (N= 6)/ Ethylbenzene (N= 6)/ Acetone (N= 4) | Ethylbenzene,Xylene (N=6) |
| 12: Manufacture of tobacco products | 120: Manufacture of tobacco products | 2 | 50% | Aliphatic and alicyclic hydrocarbons (N=1)/ Ethanol (N=1)/ Ethyl acetate (N=1) | Aliphatic and alicyclic hydrocarbons,Ethanol,Ethyl acetate (N=1) |
| 13: Manufacture of textiles | 131: Preparation and spinning of textile fibres | 24 | 75% | Ammonia, anhydrous (N=10)/ Toluene (N=10)/ Quartz (N= 6)/ Cristobalite (N= 5)/ Boron (N= 2) | Ammonia, anhydrous,Toluene (N=10) |
|  | 132: Weaving of textiles | 31 | 45.2% | Butanone (N=7)/ Toluene (N=6)/ Xylene (N=5)/ 1-Methoxypropan-2-ol (N=3)/ Acetone (N=3) | Butanone,Toluene (N=5) |
|  | 133: Finishing of textiles | 12 | 41.7% | Acetone (N=2)/ 1-Methoxypropan-2-ol (N=1)/ 1,2,3-Trimethylbenzene (N=1)/ 1,2,4-Trimethylbenzene (N=1)/ 2-Methoxy-1-methylethyl acetate (N=1) | 1-Methoxypropan-2-ol,4-Hydroxy-4-methylpentan-2-one,Cyclohexanone,Monocyclic aromatic hydrocarbons (N=1) |
|  | 139: Manufacture of other textiles | 74 | 37.8% | Butanone (N=14)/ Toluene (N=14)/ Acetone (N= 9)/ Ethyl acetate (N= 6)/ Xylene (N= 5) | Butanone,Toluene (N=9) |
| 14: Manufacture of wearing apparel | 141: Manufacture of wearing apparel, except fur apparel | 8 | 62.5% | Ethyl acetate (N=4)/ Acetone (N=3)/ Aliphatic and alicyclic hydrocarbons (N=2)/ Butanone (N=2)/ C6-C12 hydrocarbons (N=2) | Acetone,Ethyl acetate (N=3) |
|  | 143: Manufacture of knitted and crocheted apparel | 3 | 0% |  |  |
| 15: Manufacture of leather and related products | 151: Tanning and dressing of leather: manufacture of luggage, handbags, saddlery and harness: dressing and dyeing of fur | 95 | 44.2% | Acetone (N=22)/ Butanone (N=11)/ Cyclohexane (N= 9)/ Iron (N= 8)/ 1-Methoxypropan-2-ol (N= 7) | Acetone,Butanone (N=11) |
|  | 152: Manufacture of footwear | 20 | 75% | Acetone (N=11)/ Butanone (N= 7)/ Aliphatic and alicyclic hydrocarbons (N= 6)/ Ethyl acetate (N= 4)/ N-hexane (N= 4) | Acetone,Butanone (N=7) |
| 16: Manufacture of wood and of products of wood and cork, except furniture: manufacture of articles of straw and plaiting materials | 161: Sawmilling and planing of wood | 531 | 1.7% | Wood dust (N=7)/ 1-Methoxypropan-2-ol (N=3)/ Cobalt (N=3)/ Iron (N=3)/ Aliphatic and alicyclic hydrocarbons (N=2) | Cobalt,Iron,Wood dust (N=3) |
|  | 162: Manufacture of products of wood, cork, straw and plaiting materials | 1598 | 6.3% | Wood dust (N=83)/ Lead (N=28)/ Formaldehyde (N=21)/ Toluene (N=16)/ Benz[a]anthracene (N=15) | Lead,Wood dust (N=27) |
| 17: Manufacture of paper and paper products | 171: Manufacture of pulp, paper and paperboard | 233 | 19.7% | Ammonia, anhydrous (N=30)/ Wood dust (N=25)/ Acetone (N= 8)/ Sulphuric acid (N= 6)/ Chromium (metal and inorganic Cr(II)/Cr(III) compounds) (N= 5) | Ammonia, anhydrous,Wood dust (N=24) |
|  | 172: Manufacture of articles of paper and paperboard | 99 | 42.4% | Ethyl acetate (N=16)/ Ethanol (N=14)/ Propan-2-ol (N=13)/ Butanone (N=12)/ Propyl acetate (N= 9) | Ethanol,Ethyl acetate (N=13) |
| 18: Printing and reproduction of recorded media | 181: Printing and service activities related to printing | 152 | 49.3% | Ethanol (N=30)/ Propan-2-ol (N=28)/ Ethyl acetate (N=26)/ Acetone (N=23)/ 1-Methoxypropan-2-ol (N=22) | Ethanol,Ethyl acetate (N=19) |
| 19: Manufacture of coke and refined petroleum products | 191: Manufacture of coke oven products | 13 | 7.7% | 2-Aminoethanol (N=1)/ Ammonia, anhydrous (N=1) | 2-Aminoethanol,Ammonia, anhydrous (N=1) |
|  | 192: Manufacture of refined petroleum products | 368 | 60.9% | Benzene (N=170)/ Toluene (N=170)/ Xylene (N=131)/ Ethylbenzene (N= 93)/ N-hexane (N= 81) | Benzene,Toluene (N=142) |
| 20: Manufacture of chemicals and chemical products | 201: Manufacture of basic chemicals, fertilisers and nitrogen compounds, plastics and synthetic rubber in primary forms | 979 | 37.7% | Toluene (N=129)/ Acetone (N= 95)/ Ammonia, anhydrous (N= 95)/ Benzene (N= 86)/ Chloroethylene (N= 83) | Acetone,Toluene (N=50) |
|  | 202: Manufacture of pesticides and other agrochemical products | 110 | 29.1% | Chlorobenzene (N=10)/ Cyclohexanone (N= 8)/ Methanol (N= 7)/ Toluene (N= 7)/ Ammonia, anhydrous (N= 6) | Ammonia, anhydrous,Chlorobenzene (N=5) |
|  | 203: Manufacture of paints, varnishes and similar coatings, printing ink and mastics | 374 | 59.6% | Xylene (N=137)/ Ethylbenzene (N=107)/ Toluene (N= 84)/ Butanone (N= 83)/ Acetone (N= 63) | Ethylbenzene,Xylene (N=100) |
|  | 204: Manufacture of soap and detergents, cleaning and polishing preparations, perfumes and toilet preparations | 139 | 33.8% | Chloroform (N=15)/ Acetone (N=14)/ Butanone (N=11)/ Hydrogen chloride (N=10)/ Nitric acid (N= 9) | Acetone,Chloroform (N=7) |
|  | 205: Manufacture of other chemical products | 523 | 37.7% | Toluene (N=83)/ Xylene (N=63)/ Acetone (N=51)/ N-hexane (N=44)/ Butanone (N=43) | Toluene,Xylene (N=51) |
|  | 206: Manufacture of man-made fibres | 6 | 16.7% | Chloroform (N=1)/ Phenol (N=1) | Chloroform,Phenol (N=1) |
| 21: Manufacture of basic pharmaceutical products and pharmaceutical preparations | 211: Manufacture of basic pharmaceutical products | 106 | 43.4% | Dichloromethane (N=24)/ Toluene (N=22)/ Acetone (N=21)/ Methanol (N=18)/ Cyclohexane (N=14) | Dichloromethane,Toluene (N=16) |
|  | 212: Manufacture of pharmaceutical preparations | 184 | 33.2% | Acetonitrile (N=24)/ Acetone (N=23)/ Methanol (N=21)/ Chloroform (N=19)/ Dichloromethane (N=19) | Acetonitrile,Methanol (N=18) |
| 22: Manufacture of rubber and plastic products | 221: Manufacture of rubber products | 354 | 43.8% | Toluene (N=71)/ Butanone (N=48)/ N-hexane (N=39)/ Heptane (N=38)/ Cyclohexane (N=36) | Butanone,Toluene (N=32) |
|  | 222: Manufacture of plastics products | 988 | 53% | Acetone (N=184)/ Butanone (N=139)/ Styrene (N=139)/ Xylene (N=123)/ Toluene (N=101) | Acetone,Styrene (N=111) |
| 23: Manufacture of other non-metallic mineral products | 231: Manufacture of glass and glass products | 697 | 31.1% | Quartz (N=109)/ Lead (N= 68)/ Ammonia, anhydrous (N= 52)/ Cristobalite (N= 44)/ Refractory ceramic fibers (L>5um D<3um) (N= 40) | Cristobalite,Quartz (N=41) |
|  | 232: Manufacture of refractory products | 163 | 23.3% | Quartz (N=29)/ Cristobalite (N=22)/ Hexavalent chromium (N= 9)/ Chromium (metal and inorganic Cr(II)/Cr(III) compounds) (N= 6)/ Refractory ceramic fibers (L>5um D<3um) (N= 6) | Cristobalite,Quartz (N=21) |
|  | 233: Manufacture of clay building materials | 860 | 27.2% | Quartz (N=231)/ Cristobalite (N=165)/ Tridymite (N= 64)/ Refractory ceramic fibers (L>5um D<3um) (N= 17)/ Wood dust (N= 12) | Cristobalite,Quartz (N=165) |
|  | 234: Manufacture of other porcelain and ceramic products | 180 | 38.9% | Quartz (N=58)/ Cristobalite (N=52)/ Acetone (N= 7)/ Lead (N= 7)/ Butanone (N= 6) | Cristobalite,Quartz (N=52) |
|  | 235: Manufacture of cement, lime and plaster | 325 | 39.4% | Quartz (N=104)/ Hexavalent chromium (N= 58)/ Cristobalite (N= 55)/ Tridymite (N= 49)/ Iron (N= 14) | Cristobalite,Quartz (N=53) |
|  | 236: Manufacture of articles of concrete, cement and plaster | 698 | 27.4% | Quartz (N=151)/ Cristobalite (N= 91)/ Tridymite (N= 62)/ Hexavalent chromium (N= 16)/ Styrene (N= 14) | Cristobalite,Quartz (N=88) |
|  | 237: Cutting, shaping and finishing of stone | 66 | 43.9% | Cristobalite (N=25)/ Quartz (N=25)/ Toluene (N= 3)/ 1-Methoxypropan-2-ol (N= 1)/ 1,2,4-Trimethylbenzene (N= 1) | Cristobalite,Quartz (N=25) |
|  | 239: Manufacture of abrasive products and non-metallic mineral products n.e.c. | 519 | 33.3% | Quartz (N=105)/ Cristobalite (N=102)/ Ammonia, anhydrous (N= 57)/ Phenol (N= 55)/ Acetone (N= 19) | Cristobalite,Quartz (N=95) |
| 24: Manufacture of basic metals | 241: Manufacture of basic iron and steel and of ferro-alloys | 572 | 42.3% | Lead (N=88)/ Quartz (N=84)/ Hexavalent chromium (N=66)/ Chromium (metal and inorganic Cr(II)/Cr(III) compounds) (N=63)/ Cristobalite (N=48) | Cristobalite,Quartz (N=47) |
|  | 242: Manufacture of tubes, pipes, hollow profiles and related fittings, of steel | 88 | 54.5% | Hexavalent chromium (N=16)/ Acetone (N=13)/ Lead (N=12)/ Butanone (N= 9)/ Manganese (N= 8) | Hexavalent chromium,Lead (N=10) |
|  | 243: Manufacture of other products of first processing of steel | 124 | 28.2% | Butanone (N=11)/ Lead (N=10)/ Xylene (N=10)/ 2-Methoxy-1-methylethyl acetate (N= 7)/ Ethylbenzene (N= 7) | Ethylbenzene,Xylene (N=7) |
|  | 244: Manufacture of basic precious and other non-ferrous metals | 587 | 29% | Lead (N=108)/ Chromium (metal and inorganic Cr(II)/Cr(III) compounds) (N= 79)/ Hexavalent chromium (N= 67)/ Refractory ceramic fibers (L>5um D<3um) (N= 28)/ Hydrogen fluoride (N= 20) | Chromium (metal and inorganic Cr(II)/Cr(III) compounds),Hexavalent chromium (N=56) |
|  | 245: Casting of metals | 734 | 42.9% | Quartz (N=209)/ Cristobalite (N=124)/ Xylene (N= 59)/ Ethylbenzene (N= 54)/ Iron (N= 52) | Cristobalite,Quartz (N=123) |
| 25: Manufacture of fabricated metal products, except machinery and equipment | 251: Manufacture of structural metal products | 285 | 48.1% | Manganese (N=75)/ Iron (N=73)/ Copper (N=51)/ Zinc (N=48)/ Nickel (N=44) | Iron,Manganese (N=68) |
|  | 252: Manufacture of tanks, reservoirs and containers of metal | 103 | 47.6% | Iron (N=23)/ Manganese (N=17)/ Zinc (N=17)/ Copper (N=14)/ Nickel (N=14) | Iron,Manganese (N=17) |
|  | 253: Manufacture of steam generators, except central heating hot water boilers | 19 | 42.1% | 2-Methoxy-1-methylethyl acetate (N=2)/ Chromium (metal and inorganic Cr(II)/Cr(III) compounds) (N=2)/ Cobalt (N=2)/ Copper (N=2)/ Diiron trioxide (N=2) | Chromium (metal and inorganic Cr(II)/Cr(III) compounds),Cobalt,Copper,Diiron trioxide,Hexavalent chromium,Manganese,Nickel (N=2) |
|  | 254: Manufacture of weapons and ammunition | 45 | 15.6% | Lead (N=6)/ Iron (N=4)/ Barium (N=3)/ Butanone (N=1)/ Dichloromethane (N=1) | Iron,Lead (N=4) |
|  | 255: Forging, pressing, stamping and roll-forming of metal: powder metallurgy | 147 | 53.7% | Iron (N=41)/ Manganese (N=32)/ Zinc (N=26)/ Copper (N=20)/ Hexavalent chromium (N=18) | Iron,Manganese (N=31) |
|  | 256: Treatment and coating of metals: machining | 588 | 46.4% | Hexavalent chromium (N=96)/ Iron (N=69)/ Lead (N=63)/ Nickel (N=56)/ Total chromium (N=51) | Iron,Zinc (N=42) |
|  | 257: Manufacture of cutlery, tools and general hardware | 110 | 47.3% | Iron (N=22)/ Cobalt (N=12)/ Total chromium (N=12)/ Copper (N= 9)/ Metalworking fluids (inhalable fraction) (N= 9) | Iron,Total chromium (N=10) |
|  | 259: Manufacture of other fabricated metal products | 466 | 42.5% | Xylene (N=52)/ Iron (N=46)/ Manganese (N=42)/ Ethylbenzene (N=34)/ 2-Methoxy-1-methylethyl acetate (N=29) | Iron,Manganese (N=41) |
| 26: Manufacture of computer, electronic and optical products | 261: Manufacture of electronic components and boards | 310 | 41% | Xylene (N=51)/ Ethylbenzene (N=38)/ Toluene (N=38)/ Butanone (N=37)/ Acetone (N=32) | Ethylbenzene,Xylene (N=38) |
|  | 262: Manufacture of computers and peripheral equipment | 1 | 100% | Cristobalite (N=1)/ Quartz (N=1) | Cristobalite,Quartz (N=1) |
|  | 263: Manufacture of communication equipment | 45 | 33.3% | Butanone (N=8)/ 2-Methoxy-1-methylethyl acetate (N=7)/ Toluene (N=7)/ 1-Methoxypropan-2-ol (N=4)/ Chromium (metal and inorganic Cr(II)/Cr(III) compounds) (N=4) | 2-Methoxy-1-methylethyl acetate,Butanone (N=5) |
|  | 264: Manufacture of consumer electronics | 10 | 30% | Acetone (N=2)/ Butanone (N=1)/ Heptane (N=1)/ Toluene (N=1)/ Xylene (N=1) | Acetone,Heptane (N=1) |
|  | 265: Manufacture of instruments and appliances for measuring, testing and navigation: watches and clocks | 236 | 28.8% | Acetone (N=31)/ Toluene (N=28)/ Xylene (N=26)/ Butanone (N=18)/ Ethylbenzene (N=18) | Toluene,Xylene (N=17) |
|  | 266: Manufacture of irradiation, electromedical and electrotherapeutic equipment | 27 | 63% | Ethylbenzene (N=16)/ Xylene (N=16)/ Hexavalent chromium (N= 1)/ Lead (N= 1) | Ethylbenzene,Xylene (N=16) |
|  | 267: Manufacture of optical instruments and photographic equipment | 16 | 43.8% | Acetone (N=6)/ 4-Methylpentan-2-one (N=2)/ Aliphatic and alicyclic hydrocarbons (N=2)/ Butanone (N=2)/ Diethyl ether (N=2) | 4-Methylpentan-2-one,Acetone,Aliphatic and alicyclic hydrocarbons,Butanone,Ethyl acetate,N-butyl acetate,Propan-2-ol (N=2) |
|  | 268: Manufacture of magnetic and optical media | 5 | 100% | Tetrahydrofuran (N=5)/ Toluene (N=5)/ Butanone (N=3)/ Isopropyl acetate (N=3)/ Cyclohexanone (N=2) | Tetrahydrofuran,Toluene (N=5) |
| 27: Manufacture of electrical equipment | 271: Manufacture of electric motors, generators, transformers and electricity distribution and control apparatus | 270 | 37% | Xylene (N=37)/ Butanone (N=32)/ Ethylbenzene (N=26)/ Toluene (N=22)/ Acetone (N=21) | Ethylbenzene,Xylene (N=25) |
|  | 272: Manufacture of batteries and accumulators | 464 | 3.7% | Lead (N=8)/ Sulphuric acid (N=5)/ Butanone (N=4)/ Heptane (N=4)/ N-hexane (N=4) | Lead,Sulphuric acid (N=5) |
|  | 273: Manufacture of wiring and wiring devices | 145 | 35.2% | Butanone (N=21)/ Acetone (N= 9)/ Toluene (N= 8)/ 4-Methylpentan-2-one (N= 7)/ Cyclohexanone (N= 7) | Butanone,Toluene (N=5) |
|  | 274: Manufacture of electric lighting equipment | 51 | 43.1% | Butanone (N=10)/ Methyl methacrylate (N= 7)/ Xylene (N= 7)/ Acetone (N= 6)/ Cyclohexanone (N= 6) | Acetone,Butanone (N=5) |
|  | 275: Manufacture of domestic appliances | 93 | 48.4% | Quartz (N=19)/ Cristobalite (N=16)/ Iron (N=15)/ Manganese (N= 8)/ Nickel (N= 8) | Cristobalite,Quartz (N=16) |
|  | 279: Manufacture of other electrical equipment | 86 | 30.2% | Hexavalent chromium (N=8)/ Toluene (N=6)/ Lead (N=5)/ Quartz (N=5)/ 2-Methoxy-1-methylethyl acetate (N=4) | 2-Methoxy-1-methylethyl acetate,Toluene (N=4) |
| 28: Manufacture of machinery and equipment n.e.c. | 281: Manufacture of general-purpose machinery | 316 | 51.3% | Xylene (N=36)/ Acetone (N=33)/ Zinc (N=33)/ Iron (N=31)/ Ethylbenzene (N=27) | Ethylbenzene,Xylene (N=27) |
|  | 282: Manufacture of other general-purpose machinery | 261 | 42.5% | Xylene (N=41)/ Ethylbenzene (N=34)/ Iron (N=21)/ Manganese (N=21)/ Acetone (N=20) | Ethylbenzene,Xylene (N=34) |
|  | 283: Manufacture of agricultural and forestry machinery | 67 | 77.6% | Ethylbenzene (N=25)/ Xylene (N=25)/ Iron (N=21)/ Manganese (N=21)/ Mesitylene (N=15) | Ethylbenzene,Xylene (N=25) |
|  | 284: Manufacture of metal forming machinery and machine tools | 26 | 46.2% | Ethylbenzene (N=4)/ Metalworking fluids (inhalable fraction) (N=4)/ Water-soluble metalworking fluids (N=4)/ Xylene (N=4)/ 1,2,4-Trimethylbenzene (N=3) | Ethylbenzene,Xylene (N=4) |
|  | 289: Manufacture of other special-purpose machinery | 163 | 47.2% | Manganese (N=36)/ Nickel (N=36)/ Iron (N=34)/ Total chromium (N=26)/ Copper (N=22) | Manganese,Nickel (N=36) |
| 29: Manufacture of motor vehicles, trailers and semi-trailers | 291: Manufacture of motor vehicles | 179 | 46.9% | Xylene (N=41)/ Ethylbenzene (N=34)/ Quartz (N=24)/ Cristobalite (N=20)/ 1,2,4-Trimethylbenzene (N=17) | Ethylbenzene,Xylene (N=34) |
|  | 292: Manufacture of bodies (coachwork) for motor vehicles: manufacture of trailers and semi-trailers | 252 | 30.6% | Acetone (N=47)/ Toluene (N=41)/ Styrene (N=29)/ Xylene (N=29)/ Ethylbenzene (N=22) | Acetone,Toluene (N=36) |
|  | 293: Manufacture of parts and accessories for motor vehicles | 350 | 33.1% | Xylene (N=39)/ Toluene (N=35)/ Butanone (N=32)/ Acetone (N=24)/ Ethylbenzene (N=17) | Toluene,Xylene (N=22) |
| 30: Manufacture of other transport equipment | 301: Building of ships and boats | 237 | 52.7% | Acetone (N=83)/ Styrene (N=60)/ Methyl methacrylate (N=30)/ Butanone (N=24)/ Chromium (metal and inorganic Cr(II)/Cr(III) compounds) (N=19) | Acetone,Styrene (N=60) |
|  | 302: Manufacture of railway locomotives and rolling stock | 38 | 57.9% | Quartz (N=8)/ 1-Methoxypropan-2-ol (N=7)/ Lead (N=7)/ Xylene (N=7)/ 2-Methoxy-1-methylethyl acetate (N=6) | 1-Methoxypropan-2-ol,2-Methoxy-1-methylethyl acetate (N=6) |
|  | 303: Manufacture of air and spacecraft and related machinery | 668 | 41.3% | Butanone (N=116)/ Acetone (N= 95)/ 2-Methoxy-1-methylethyl acetate (N= 91)/ Hexavalent chromium (N= 88)/ Toluene (N= 84) | 1-Methoxypropan-2-ol,2-Methoxy-1-methylethyl acetate (N=61) |
|  | 304: Manufacture of military fighting vehicles | 8 | 12.5% | Iron (N=1)/ Nickel (N=1)/ Zinc (N=1) | Iron,Nickel,Zinc (N=1) |
|  | 309: Manufacture of transport equipment n.e.c. | 24 | 87.5% | Toluene (N=14)/ Ethylbenzene (N=10)/ Xylene (N=10)/ 1,2,4-Trimethylbenzene (N= 7)/ 2-Methoxy-1-methylethyl acetate (N= 7) | Ethylbenzene,Xylene (N=10) |
| 31: Manufacture of furniture | 310: Manufacture of furniture | 650 | 8.6% | Acetone (N=25)/ Toluene (N=25)/ Xylene (N=21)/ Butanone (N=19)/ Ethylbenzene (N=19) | Acetone,Toluene (N=20) |
| 32: Other manufacturing | 321: Manufacture of jewellery, bijouterie and related articles | 32 | 43.8% | Butanone (N=4)/ Xylene (N=3)/ 1-Methoxypropan-2-ol (N=2)/ 2-Methoxy-1-methylethyl acetate (N=2)/ Acetone (N=2) | Butanone,Xylene (N=3) |
|  | 322: Manufacture of musical instruments | 34 | 17.6% | Butanone (N=3)/ Ethylbenzene (N=3)/ Xylene (N=3)/ Copper (N=2)/ Lead (N=2) | Butanone,Ethylbenzene,Xylene (N=3) |
|  | 323: Manufacture of sports goods | 40 | 32.5% | Acetone (N=8)/ Butanone (N=8)/ Styrene (N=8)/ Methyl methacrylate (N=5)/ Xylene (N=3) | Acetone,Styrene (N=8) |
|  | 324: Manufacture of games and toys | 10 | 20% | 2-Methoxy-1-methylethyl acetate (N=2)/ 1-Methoxypropan-2-ol (N=1)/ Cyclohexanone (N=1)/ Ethylbenzene (N=1)/ Xylene (N=1) | 1-Methoxypropan-2-ol,2-Methoxy-1-methylethyl acetate,Cyclohexanone (N=1) |
|  | 325: Manufacture of medical and dental instruments and supplies | 248 | 36.3% | Acetone (N=22)/ Butanone (N=22)/ Xylene (N=15)/ Nickel (N=14)/ Ethylbenzene (N=13) | Ethylbenzene,Xylene (N=13) |
|  | 329: Manufacturing n.e.c. | 84 | 44% | Butanone (N=19)/ Toluene (N=18)/ Acetone (N=13)/ Xylene (N= 9)/ Ethylbenzene (N= 8) | Butanone,Toluene (N=17) |
| 33: Repair and installation of machinery and equipment | 331: Repair of fabricated metal products, machinery and equipment | 369 | 43.4% | Hexavalent chromium (N=42)/ Iron (N=42)/ Xylene (N=42)/ Toluene (N=40)/ Manganese (N=36) | Iron,Manganese (N=33) |
|  | 332: Installation of industrial machinery and equipment | 117 | 53% | Iron (N=32)/ Manganese (N=29)/ Nickel (N=27)/ Hexavalent chromium (N=25)/ Total chromium (N=23) | Iron,Manganese (N=28) |
| 35: Electricity, gas, steam and air conditioning supply | 351: Electric power generation, transmission and distribution | 220 | 34.5% | Acetone (N=28)/ Toluene (N=21)/ Xylene (N=15)/ Heptane (N=14)/ Ammonia, anhydrous (N=13) | Acetone,Heptane (N=14) |
|  | 352: Manufacture of gas: distribution of gaseous fuels through mains | 9 | 44.4% | Ammonia, anhydrous (N=2)/ Butanone (N=2)/ Hydrogen sulphide (N=2)/ 2-Methylbutane (N=1)/ Aluminium (N=1) | Ammonia, anhydrous,Butanone,Hydrogen sulphide (N=2) |
|  | 353: Steam and air conditioning supply | 204 | 25% | Quartz (N=27)/ Wood dust (N=24)/ Lead (N=16)/ Hexavalent chromium (N=14)/ Cristobalite (N=10) | Quartz,Wood dust (N=17) |
| 36: Water collection, treatment and supply | 360: Water collection, treatment and supply | 40 | 30% | Acetone (N=3)/ N-hexane (N=3)/ Acetonitrile (N=2)/ Ammonia, anhydrous (N=2)/ Chloroform (N=2) | Acetone,N-hexane (N=3) |
| 37: Sewerage | 370: Sewerage | 94 | 34% | Hydrogen sulphide (N=15)/ Ammonia, anhydrous (N=13)/ Toluene (N= 8)/ Acetone (N= 7)/ N-hexane (N= 6) | Ammonia, anhydrous,Hydrogen sulphide (N=10) |
| 38: Waste collection, treatment and disposal activities: materials recovery | 381: Waste collection | 273 | 59.3% | Lead (N=66)/ Toluene (N=64)/ Xylene (N=57)/ Ethylbenzene (N=49)/ Acetone (N=44) | Toluene,Xylene (N=54) |
|  | 382: Waste treatment and disposal | 574 | 60.6% | Lead (N=172)/ Ammonia, anhydrous (N=117)/ Toluene (N=116)/ Xylene (N=110)/ Ethylbenzene (N= 95) | Toluene,Xylene (N=94) |
|  | 383: Materials recovery | 380 | 42.9% | Lead (N=78)/ Quartz (N=41)/ Toluene (N=41)/ Xylene (N=39)/ Hexavalent chromium (N=38) | Toluene,Xylene (N=38) |
| 39: Remediation activities and other waste management services | 390: Remediation activities and other waste management services | 108 | 23.1% | Toluene (N=13)/ Xylene (N=11)/ Ethylbenzene (N=10)/ Benzene (N= 8)/ Lead (N= 8) | Toluene,Xylene (N=11) |
| 41: Construction of buildings | 411: Development of building projects | 2 | 0% |  |  |
|  | 412: Construction of residential and non-residential buildings | 170 | 23.5% | Quartz (N=18)/ Iron (N= 9)/ Manganese (N= 8)/ Cristobalite (N= 7)/ Tridymite (N= 6) | Iron,Manganese (N=8) |
| 42: Civil engineering | 421: Construction of roads and railways | 238 | 33.2% | Quartz (N=53)/ Cristobalite (N=37)/ Iron (N= 9)/ Aluminium (N= 8)/ Bitumen fumes (N= 8) | Cristobalite,Quartz (N=37) |
|  | 422: Construction of utility projects | 42 | 50% | Manganese (N=11)/ Iron (N= 9)/ Quartz (N= 8)/ Nickel (N= 7)/ Copper (N= 6) | Iron,Manganese (N=9) |
|  | 429: Construction of other civil engineering projects | 55 | 32.7% | Quartz (N=7)/ Butanone (N=4)/ Cristobalite (N=4)/ Diesel exhaust, elemental carbon (N=3)/ Iron (N=3) | Cristobalite,Quartz (N=4) |
| 43: Specialised construction activities | 431: Demolition and site preparation | 249 | 27.7% | Quartz (N=43)/ Lead (N=33)/ Iron (N=22)/ Cristobalite (N=16)/ Zinc (N=14) | Iron,Lead (N=18) |
|  | 432: Electrical, plumbing and other construction installation activities | 122 | 30.3% | Iron (N=15)/ Zinc (N=10)/ Copper (N= 9)/ Manganese (N= 8)/ Nickel (N= 7) | Iron,Zinc (N=10) |
|  | 433: Building completion and finishing | 458 | 19.7% | Iron (N=37)/ Lead (N=27)/ Zinc (N=25)/ Wood dust (N=24)/ Manganese (N=22) | Iron,Zinc (N=25) |
|  | 439: Other specialised construction activities | 317 | 14.5% | Iron (N=20)/ Manganese (N=13)/ Quartz (N=12)/ Zinc (N=11)/ Lead (N=10) | Iron,Manganese (N=12) |
| 45: Wholesale and retail trade and repair of motor vehicles and motorcycles | 451: Sale of motor vehicles | 85 | 55.3% | Xylene (N=30)/ Ethylbenzene (N=26)/ Toluene (N=24)/ Butanone (N=21)/ Acetone (N=17) | Ethylbenzene,Xylene (N=26) |
|  | 452: Maintenance and repair of motor vehicles | 55 | 60% | Toluene (N=21)/ Xylene (N=19)/ Acetone (N=18)/ Butanone (N=18)/ Ethylbenzene (N=15) | Toluene,Xylene (N=19) |
|  | 453: Sale of motor vehicle parts and accessories | 20 | 60% | Aliphatic and alicyclic hydrocarbons (N=3)/ Aluminium (N=3)/ Iron (N=3)/ Magnesium (N=3)/ N-hexane (N=3) | Aluminium,Iron,Magnesium (N=3) |
|  | 454: Sale, maintenance and repair of motorcycles and related parts and accessories | 5 | 40% | Toluene (N=2)/ Xylene (N=2)/ Benzene (N=1)/ Butanone (N=1)/ C6-C12 hydrocarbons (N=1) | Toluene,Xylene (N=2) |
| 46: Wholesale trade, except of motor vehicles and motorcycles | 461: Wholesale on a fee or contract basis | 128 | 12.5% | Xylene (N=8)/ 1-Methoxypropan-2-ol (N=5)/ Ethylbenzene (N=5)/ 1,2,4-Trimethylbenzene (N=4)/ 2-Methoxy-1-methylethyl acetate (N=4) | Ethylbenzene,Xylene (N=5) |
|  | 462: Wholesale of agricultural raw materials and live animals | 17 | 41.2% | Methanol (N=4)/ Propan-2-ol (N=4)/ Ethylbenzene (N=2)/ Xylene (N=2)/ 1,2,4-Trimethylbenzene (N=1) | Methanol,Propan-2-ol (N=4) |
|  | 463: Wholesale of food, beverages and tobacco | 28 | 21.4% | Chloroform (N=2)/ Cyclohexane (N=2)/ Methanol (N=2)/ 1-Methoxypropan-2-ol (N=1)/ Acetone (N=1) | 1-Methoxypropan-2-ol,Acetone,Butanone,N-hexane,Tetrahydrofuran,Toluene,Xylene (N=1) |
|  | 464: Wholesale of household goods | 77 | 26% | Acetone (N=7)/ Butanone (N=4)/ Toluene (N=4)/ 4-Methylpentan-2-one (N=3)/ Chloroform (N=3) | 4-Methylpentan-2-one,Butanone (N=3) |
|  | 465: Wholesale of information and communication equipment | 3 | 33.3% | Tridymite (N=1)/ Wood dust (N=1) | Tridymite,Wood dust (N=1) |
|  | 466: Wholesale of other machinery, equipment and supplies | 121 | 43% | Iron (N=16)/ Xylene (N=16)/ Toluene (N=15)/ Acetone (N=13)/ Ethylbenzene (N=11) | Ethylbenzene,Xylene (N=11) |
|  | 467: Other specialised wholesale | 646 | 27.6% | Acetone (N=59)/ Xylene (N=53)/ Toluene (N=51)/ Butanone (N=39)/ Ethylbenzene (N=37) | Ethylbenzene,Xylene (N=35) |
|  | 469: Non-specialised wholesale trade | 15 | 20% | Xylene (N=3)/ Ethylbenzene (N=2)/ Propan-2-ol (N=2)/ (2-Methoxymethylethoxy)propanol (N=1)/ 1-Methoxypropan-2-ol (N=1) | Ethylbenzene,Propan-2-ol,Xylene (N=2) |
| 47: Retail trade, except of motor vehicles and motorcycles | 471: Retail sale in non-specialised stores | 30 | 13.3% | Toluene (N=2)/ Acetone (N=1)/ Aliphatic and alicyclic hydrocarbons (N=1)/ Benzene (N=1)/ Butanone (N=1) | Acetone,Butanone (N=1) |
|  | 473: Retail sale of automotive fuel in specialised stores | 12 | 58.3% | Benzene (N=6)/ Toluene (N=6)/ Ethylbenzene (N=5)/ Xylene (N=5)/ Heptane (N=4) | Benzene,Toluene (N=5) |
|  | 474: Retail sale of information and communication equipment in specialised stores | 1 | 0% |  |  |
|  | 475: Retail sale of other household equipment in specialised stores | 205 | 2.4% | Ethylbenzene (N=3)/ Xylene (N=3)/ Formaldehyde (N=2)/ N-butyl acetate (N=2)/ Pentane (N=2) | Ethylbenzene,Xylene (N=3) |
|  | 476: Retail sale of cultural and recreation goods in specialised stores | 18 | 50% | Acetone (N=5)/ Tetrahydrofuran (N=4)/ Chloroform (N=3)/ Aliphatic and alicyclic hydrocarbons (N=2)/ Butanone (N=2) | Acetone,Tetrahydrofuran (N=4) |
|  | 477: Retail sale of other goods in specialised stores | 21 | 9.5% | 1-Methoxypropan-2-ol (N=1)/ Acetone (N=1)/ Benzene (N=1)/ C6-C12 hydrocarbons (N=1)/ Propan-2-ol (N=1) | 1-Methoxypropan-2-ol,Acetone,C6-C12 hydrocarbons,Propan-2-ol (N=1) |
|  | 479: Retail trade not in stores, stalls or markets | 5 | 60% | Heptane (N=3)/ Tetrahydrofuran (N=3)/ N-hexane (N=2)/ Toluene (N=2)/ Hydrogen chloride (N=1) | Heptane,Tetrahydrofuran (N=3) |
| 49: Land transport and transport via pipelines | 491: Passenger rail transport, interurban | 175 | 43.4% | Quartz (N=31)/ Lead (N=29)/ Chromium (metal and inorganic Cr(II)/Cr(III) compounds) (N=23)/ Acetone (N=21)/ Cristobalite (N=19) | Chromium (metal and inorganic Cr(II)/Cr(III) compounds),Lead (N=20) |
|  | 492: Freight rail transport | 9 | 44.4% | Acetone (N=3)/ Cyclohexane (N=3)/ Heptane (N=3)/ N-hexane (N=3)/ Pentane (N=3) | Acetone,Cyclohexane,Heptane,N-hexane,Pentane (N=3) |
|  | 493: Other passenger land transport | 106 | 34.9% | Acetone (N=18)/ Xylene (N=12)/ Butanone (N=11)/ Quartz (N=11)/ Carbon dioxide (N= 9) | Acetone,Butanone (N=10) |
|  | 494: Freight transport by road and removal services | 49 | 38.8% | Toluene (N=9)/ Xylene (N=9)/ N-hexane (N=7)/ Ethylbenzene (N=6)/ Quartz (N=6) | N-hexane,Toluene (N=7) |
|  | 495: Transport via pipeline | 43 | 46.5% | Toluene (N=12)/ N-hexane (N= 9)/ Benzene (N= 8)/ Pentane (N= 7)/ Xylene (N= 7) | Benzene,N-hexane (N=7) |
| 50: Water transport | 501: Sea and coastal passenger water transport | 7 | 57.1% | Ethylbenzene (N=2)/ Xylene (N=2)/ (2-Methoxymethylethoxy)propanol (N=1)/ 2-Aminoethanol (N=1)/ Aliphatic and alicyclic hydrocarbons (N=1) | Ethylbenzene,Xylene (N=2) |
|  | 504: Inland freight water transport | 7 | 71.4% | Benzene (N=5)/ Chloroethylene (N=5) | Benzene,Chloroethylene (N=5) |
| 51: Air transport | 511: Passenger air transport | 13 | 61.5% | Toluene (N=6)/ Butanone (N=4)/ Xylene (N=4)/ Acetone (N=3)/ Cyclohexane (N=2) | Butanone,Toluene (N=4) |
| 52: Warehousing and support activities for transportation | 521: Warehousing and storage | 234 | 54.3% | Toluene (N=112)/ Benzene (N= 84)/ Xylene (N= 79)/ N-hexane (N= 63)/ Ethylbenzene (N= 48) | Benzene,Toluene (N=79) |
|  | 522: Support activities for transportation | 149 | 21.5% | Toluene (N=11)/ Xylene (N= 8)/ Ethylbenzene (N= 7)/ Pentane (N= 5)/ 2-Methylbutane (N= 4) | Ethylbenzene,Xylene (N=4) |
| 53: Postal and courier activities | 531: Postal activities under universal service obligation | 11 | 27.3% | Hexavalent chromium (N=2)/ Sulphuric acid (N=2)/ Acetone (N=1)/ Chloroform (N=1) | Hexavalent chromium,Sulphuric acid (N=2) |
| 55: Accommodation | 551: Hotels and similar accommodation | 2 | 50% | Acetone (N=1)/ Styrene (N=1) | Acetone,Styrene (N=1) |
|  | 553: Camping grounds, recreational vehicle parks and trailer parks | 1 | 0% |  |  |
|  | 559: Other accommodation | 8 | 0% |  |  |
| 56: Food and beverage service activities | 561: Restaurants and mobile food service activities | 14 | 42.9% | Lead (N=4)/ Mercury (N=4)/ Benz[a]anthracene (N=2)/ Benzo(e)acephenanthrylene (N=2)/ Benzo(k)fluoranthene (N=2) | Lead,Mercury (N=4) |
|  | 562: Event catering and other food service activities | 9 | 22.2% | Ethylbenzene (N=2)/ Toluene (N=2)/ Xylene (N=2)/ 1,2,4-Trimethylbenzene (N=1)/ 2-Methoxy-1-methylethyl acetate (N=1) | Ethylbenzene,Toluene,Xylene (N=2) |
| 58: Publishing activities | 581: Publishing of books, periodicals and other publishing activities | 22 | 54.5% | 1,2,4-Trimethylbenzene (N=4)/ Heptane (N=4)/ Mesitylene (N=4)/ 2-Butoxyethanol (N=3)/ Cumene (N=3) | 1,2,4-Trimethylbenzene,Mesitylene (N=3) |
| 59: Motion picture, video and television programme production, sound recording and music publishing activities | 591: Motion picture, video and television programme activities | 1 | 0% |  |  |
| 60: Programming and broadcasting activities | 602: Television programming and broadcasting activities | 3 | 0% |  |  |
| 61: Telecommunications | 611: Wired telecommunications activities | 6 | 0% |  |  |
| 62: Computer programming, consultancy and related activities | 620: Computer programming, consultancy and related activities | 13 | 23.1% | Lead (N=3)/ Quartz (N=3)/ Cristobalite (N=1)/ Hexavalent chromium (N=1) | Lead,Quartz (N=3) |
| 63: Information service activities | 639: Other information service activities | 7 | 57.1% | Acetone (N=4)/ Diethyl ether (N=2)/ Hydrogen chloride (N=2)/ Ammonia, anhydrous (N=1)/ Toluene (N=1) | Acetone,Diethyl ether (N=2) |
| 64: Financial service activities, except insurance and pension funding | 641: Monetary intermediation | 13 | 23.1% | 1-Methoxypropan-2-ol (N=2)/ Acetone (N=1)/ Butanone (N=1)/ Hexavalent chromium (N=1)/ Quartz (N=1) | 1-Methoxypropan-2-ol,Acetone,Butanone (N=1) |
|  | 642: Activities of holding companies | 160 | 31.9% | 1,2,4-Trimethylbenzene (N=26)/ 2-Butoxyethyl acetate (N=23)/ 1,2,3-Trimethylbenzene (N=18)/ Mesitylene (N=13)/ Xylene (N=11) | 1,2,4-Trimethylbenzene,2-Butoxyethyl acetate (N=23) |
|  | 643: Trusts, funds and similar financial entities | 6 | 83.3% | Acetonitrile (N=5)/ N-hexane (N=5)/ Tetrahydrofuran (N=4)/ Dichloromethane (N=3)/ Toluene (N=3) | Acetonitrile,N-hexane (N=5) |
| 65: Insurance, reinsurance and pension funding, except compulsory social security | 651: Insurance, reinsurance and pension funding, except compulsory social security | 3 | 33.3% | Aliphatic and alicyclic hydrocarbons (N=1)/ Monocyclic aromatic hydrocarbons (N=1) | Aliphatic and alicyclic hydrocarbons,Monocyclic aromatic hydrocarbons (N=1) |
| 66: Activities auxiliary to financial services and insurance activities | 661: Activities auxiliary to financial services, except insurance and pension funding | 6 | 0% |  |  |
| 68: Real estate activities | 681: Buying and selling of own real estate | 1 | 0% |  |  |
|  | 682: Renting and operating of own or leased real estate | 97 | 15.5% | Acetone (N=5)/ Butanone (N=4)/ Cristobalite (N=4)/ Quartz (N=4)/ Styrene (N=4) | Acetone,Styrene (N=4) |
|  | 683: Real estate activities on a fee or contract basis | 13 | 7.7% | 1-Methoxypropan-2-ol (N=1)/ Cyclohexane (N=1)/ Diethyl ether (N=1) | 1-Methoxypropan-2-ol,Cyclohexane,Diethyl ether (N=1) |
| 69: Legal and accounting activities | 692: Accounting, bookkeeping and auditing activities: tax consultancy | 2 | 0% |  |  |
| 70: Activities of head offices: management consultancy activities | 701: Activities of head offices | 174 | 20.1% | Toluene (N=15)/ Butanone (N= 8)/ Acetone (N= 7)/ Xylene (N= 7)/ Ethylbenzene (N= 6) | Butanone,Toluene (N=7) |
|  | 702: Management consultancy activities | 22 | 4.5% | Acetone (N=1)/ Chloroform (N=1) | Acetone,Chloroform (N=1) |
| 71: Architectural and engineering activities: technical testing and analysis | 711: Architectural and engineering activities and related technical consultancy | 211 | 39.8% | Toluene (N=22)/ Heptane (N=20)/ Xylene (N=20)/ Acetone (N=17)/ Quartz (N=17) | Ethylbenzene,Xylene (N=14) |
|  | 712: Technical testing and analysis | 196 | 32.7% | Toluene (N=35)/ Acetone (N=25)/ N-hexane (N=21)/ Heptane (N=20)/ Xylene (N=18) | Acetone,Toluene (N=18) |
| 72: Scientific research and development | 721: Research and experimental development on natural sciences and engineering | 297 | 26.9% | Acetone (N=34)/ Toluene (N=29)/ Dichloromethane (N=22)/ Acetonitrile (N=20)/ Methanol (N=16) | Acetone,Toluene (N=18) |
|  | 722: Research and experimental development on social sciences and humanities | 7 | 28.6% | Dichloromethane (N=1)/ Iron (N=1)/ Methanol (N=1)/ Nickel (N=1) | Dichloromethane,Methanol (N=1) |
| 73: Advertising and market research | 731: Advertising | 56 | 37.5% | 2-Butoxyethyl acetate (N=12)/ 1,2,4-Trimethylbenzene (N=10)/ Mesitylene (N= 7)/ 1,2,3-Trimethylbenzene (N= 6)/ Cristobalite (N= 4) | 1,2,4-Trimethylbenzene,2-Butoxyethyl acetate (N=9) |
| 74: Other professional, scientific and technical activities | 741: Specialised design activities | 22 | 22.7% | Acetone (N=2)/ Cyclohexane (N=2)/ Ethyl acetate (N=2)/ N-hexane (N=2)/ 2-Methoxy-1-methylethyl acetate (N=1) | Cyclohexane,Ethyl acetate,N-hexane (N=2) |
|  | 749: Other professional, scientific and technical activities n.e.c. | 64 | 31.2% | Formaldehyde (N=5)/ Xylene (N=5)/ Acetaldehyde (N=4)/ Acetone (N=4)/ Chloroform (N=4) | Acetaldehyde,Formaldehyde (N=4) |
| 75: Veterinary activities | 750: Veterinary activities | 12 | 66.7% | Diethyl ether (N=8)/ Ethanol (N=8)/ Isoflurane (N=8) | Diethyl ether,Ethanol,Isoflurane (N=8) |
| 77: Rental and leasing activities | 771: Renting and leasing of motor vehicles | 7 | 42.9% | N-hexane (N=2)/ Benzene (N=1)/ Butanone (N=1)/ Heptane (N=1)/ Toluene (N=1) | Benzene,N-hexane (N=1) |
|  | 772: Renting and leasing of personal and household goods | 18 | 44.4% | Ethylbenzene (N=3)/ Xylene (N=3)/ Aliphatic and alicyclic hydrocarbons (N=2)/ Copper (N=2)/ Iron (N=2) | Ethylbenzene,Xylene (N=3) |
|  | 773: Renting and leasing of other machinery, equipment and tangible goods | 89 | 30.3% | Xylene (N=9)/ Cyclohexane (N=7)/ Toluene (N=6)/ Acetone (N=5)/ Iron (N=5) | Cyclohexane,Xylene (N=6) |
|  | 774: Leasing of intellectual property and similar products, except copyrighted works | 3 | 66.7% | Acetone (N=2)/ Butanone (N=2)/ Tetrahydrofuran (N=1) | Acetone,Butanone (N=2) |
| 78: Employment activities | 781: Activities of employment placement agencies | 6 | 50% | Iron (N=2)/ Manganese (N=2)/ Chromium (metal and inorganic Cr(II)/Cr(III) compounds) (N=1)/ Hexavalent chromium (N=1)/ Lead (N=1) | Iron,Manganese (N=2) |
|  | 782: Temporary employment agency activities | 36 | 63.9% | Manganese (N=8)/ Metalworking fluids (inhalable fraction) (N=8)/ Water-soluble metalworking fluids (N=8)/ Chromium (metal and inorganic Cr(II)/Cr(III) compounds) (N=6)/ Copper (N=6) | Metalworking fluids (inhalable fraction),Water-soluble metalworking fluids (N=8) |
|  | 783: Other human resources provision | 4 | 50% | Acetone (N=1)/ Aliphatic and alicyclic hydrocarbons (N=1)/ Butanone (N=1)/ Cyclohexanone (N=1)/ Ethyl acetate (N=1) | Acetone,Aliphatic and alicyclic hydrocarbons,Ethyl acetate,N-hexane (N=1) |
| 79: Travel agency, tour operator and other reservation service and related activities | 799: Other reservation service and related activities | 3 | 0% |  |  |
| 80: Security and investigation activities | 801: Private security activities | 12 | 8.3% | Copper (N=1)/ Iron (N=1)/ Zinc (N=1) | Copper,Iron,Zinc (N=1) |
|  | 802: Security systems service activities | 3 | 0% |  |  |
| 81: Services to buildings and landscape activities | 811: Combined facilities support activities | 8 | 0% |  |  |
|  | 812: Cleaning activities | 83 | 43.4% | Quartz (N=12)/ Toluene (N=12)/ Xylene (N= 9)/ Aliphatic and alicyclic hydrocarbons (N= 8)/ Acetone (N= 6) | Toluene,Xylene (N=8) |
|  | 813: Landscape service activities | 10 | 10% | Cristobalite (N=1)/ Quartz (N=1) | Cristobalite,Quartz (N=1) |
| 82: Office administrative, office support and other business support activities | 821: Office administrative and support activities | 16 | 25% | Butanone (N=3)/ Toluene (N=3)/ Acetaldehyde (N=2)/ Formaldehyde (N=2)/ C6-C12 hydrocarbons (N=1) | Butanone,Toluene (N=3) |
|  | 823: Organisation of conventions and trade shows | 8 | 0% |  |  |
|  | 829: Business support service activities n.e.c. | 117 | 39.3% | Toluene (N=21)/ 2-Methoxy-1-methylethyl acetate (N=18)/ Xylene (N=16)/ 1-Methoxypropan-2-ol (N=15)/ Butanone (N=14) | 1-Methoxypropan-2-ol,2-Methoxy-1-methylethyl acetate (N=13) |
| 84: Public administration and defence: compulsory social security | 841: Administration of the State and the economic and social policy of the community | 374 | 21.7% | Xylene (N=19)/ Acetone (N=15)/ Ethylbenzene (N=15)/ Toluene (N=15)/ Butanone (N=12) | Ethylbenzene,Xylene (N=13) |
|  | 842: Provision of services to the community as a whole | 203 | 17.7% | Toluene (N=16)/ Xylene (N=16)/ Butanone (N=11)/ Acetone (N=10)/ Ethylbenzene (N=10) | Toluene,Xylene (N=11) |
|  | 843: Compulsory social security activities | 30 | 23.3% | Hydrogen fluoride (N=3)/ 1,2,4-Trimethylbenzene (N=2)/ Mesitylene (N=2)/ Nitric acid (N=2)/ Xylene (N=2) | 1,2,4-Trimethylbenzene,Mesitylene (N=2) |
| 85: Education | 851: Pre-primary education | 1 | 0% |  |  |
|  | 852: Primary education | 1 | 0% |  |  |
|  | 853: Secondary education | 263 | 7.2% | Iron (N=8)/ Copper (N=6)/ Zinc (N=5)/ Mesitylene (N=4)/ Quartz (N=4) | Copper,Iron (N=6) |
|  | 854: Higher education | 97 | 19.6% | Acetone (N=8)/ Ethanol (N=5)/ Quartz (N=5)/ Toluene (N=5)/ Dichloromethane (N=4) | Acetone,Ethanol (N=4) |
|  | 855: Other education | 95 | 7.4% | Acetone (N=4)/ Butanone (N=3)/ Styrene (N=3)/ Aliphatic and alicyclic hydrocarbons (N=2)/ Iron (N=2) | Acetone,Aliphatic and alicyclic hydrocarbons,Butanone,Toluene,Xylene (N=2) |
| 86: Human health activities | 861: Hospital activities | 234 | 31.2% | Ethanol (N=41)/ Formaldehyde (N=30)/ Sevoflurane (N=30)/ Xylene (N=28)/ Dinitrogen oxide (N=25) | Ethanol,Sevoflurane (N=24) |
|  | 862: Medical and dental practice activities | 50 | 46% | Formaldehyde (N=16)/ Xylene (N=13)/ Ethanol (N=10)/ Ethylbenzene (N= 9)/ Toluene (N= 8) | Formaldehyde,Xylene (N=12) |
|  | 869: Other human health activities | 16 | 68.8% | Methanol (N=9)/ Ethanol (N=7)/ Formaldehyde (N=7)/ Toluene (N=6)/ Xylene (N=5) | Ethanol,Formaldehyde (N=6) |
| 87: Residential care activities | 871: Residential nursing care activities | 86 | 5.8% | Dinitrogen oxide (N=2)/ 1-Methoxypropan-2-ol (N=1)/ 4-Hydroxy-4-methylpentan-2-one (N=1)/ Aliphatic and alicyclic hydrocarbons (N=1)/ Butan-1-ol (N=1) | 1-Methoxypropan-2-ol,4-Hydroxy-4-methylpentan-2-one,Aliphatic and alicyclic hydrocarbons,Butan-1-ol,Ethylbenzene,N-butyl acetate,Toluene,Xylene (N=1) |
|  | 872: Residential care activities for mental retardation, mental health and substance abuse | 62 | 8.1% | Quartz (N=4)/ Cristobalite (N=3)/ Tridymite (N=3)/ Ethanol (N=1)/ Formaldehyde (N=1) | Cristobalite,Quartz,Tridymite (N=3) |
|  | 873: Residential care activities for the elderly and disabled | 2 | 0% |  |  |
|  | 879: Other residential care activities | 10 | 0% |  |  |
| 88: Social work activities without accommodation | 881: Social work activities without accommodation for the elderly and disabled | 239 | 9.6% | Iron (N=13)/ Zinc (N= 9)/ Aluminium (N= 8)/ Manganese (N= 7)/ Copper (N= 6) | Iron,Zinc (N=9) |
|  | 889: Other social work activities without accommodation | 57 | 7% | Iron (N=3)/ Manganese (N=3)/ Copper (N=1)/ Lead (N=1)/ Mercury (N=1) | Iron,Manganese (N=3) |
| 90: Creative, arts and entertainment activities | 900: Creative, arts and entertainment activities | 52 | 3.8% | Acetone (N=2)/ Methyl methacrylate (N=2)/ Styrene (N=2) | Acetone,Methyl methacrylate,Styrene (N=2) |
| 91: Libraries, archives, museums and other cultural activities | 910: Libraries, archives, museums and other cultural activities | 66 | 9.1% | Acetone (N=4)/ Butanone (N=3)/ Wood dust (N=3)/ Hydrogen chloride (N=2)/ 2-Methoxy-1-methylethyl acetate (N=1) | Butanone,Wood dust (N=3) |
| 93: Sports activities and amusement and recreation activities | 931: Sports activities | 36 | 33.3% | Hexavalent chromium (N=11)/ Quartz (N=11)/ Aluminium (N= 1)/ Barium (N= 1)/ Copper (N= 1) | Hexavalent chromium,Quartz (N=11) |
|  | 932: Amusement and recreation activities | 14 | 21.4% | Acetone (N=2)/ Aliphatic and alicyclic hydrocarbons (N=2)/ Styrene (N=2)/ Pentane (N=1)/ Wood dust (N=1) | Acetone,Aliphatic and alicyclic hydrocarbons,Styrene (N=2) |
| 94: Activities of membership organisations | 941: Activities of business, employers and professional membership organisations | 65 | 12.3% | N-hexane (N=6)/ Toluene (N=4)/ Xylene (N=4)/ Acetone (N=3)/ Benzene (N=3) | N-hexane,Toluene (N=4) |
|  | 942: Activities of trade unions | 102 | 31.4% | 2-Methoxy-1-methylethyl acetate (N=12)/ 1-Methoxypropan-2-ol (N=10)/ Cristobalite (N=10)/ Quartz (N=10)/ Acetone (N= 5) | Cristobalite,Quartz (N=10) |
|  | 949: Activities of other membership organisations | 77 | 16.9% | Lead (N=7)/ Hexavalent chromium (N=3)/ Nickel (N=3)/ Zinc (N=3)/ Aluminium (N=2) | Lead,Nickel,Zinc (N=3) |
| 95: Repair of computers and personal and household goods | 952: Repair of personal and household goods | 8 | 75% | Ethyl acetate (N=3)/ 1-Methoxypropan-2-ol (N=2)/ Acetone (N=2)/ Aliphatic and alicyclic hydrocarbons (N=2)/ Cyclohexane (N=2) | 1-Methoxypropan-2-ol,Ethanol,Propan-2-ol (N=2) |
| 96: Other personal service activities | 960: Other personal service activities | 102 | 28.4% | Tetrachloroethylene (N=13)/ Acetone (N=10)/ Ethanol (N= 9)/ N-butyl acetate (N= 8)/ Aliphatic and alicyclic hydrocarbons (N= 7) | Acetone,Ethanol (N=7) |
| 99: Activities of extraterritorial organisations and bodies | 990: Activities of extraterritorial organisations and bodies | 2 | 100% | Acetonitrile (N=2)/ N-hexane (N=2)/ Methanol (N=1)/ N,N-Dimethylformamide (N=1) | Acetonitrile,N-hexane (N=2) |
| ZZ: Unclassified | ZZZ: Unclassified | 123 | 18.7% | Benzene (N=11)/ Refractory ceramic fibers (L>5um D<3um) (N= 8)/ Vinyl acetate (N= 7)/ Toluene (N= 6)/ Hydrogen chloride (N= 4) | Benzene,Toluene (N=4) |

## Table S3: List of tasks, all agents

List of tasks with exposure to at least one agent, number of WS, percentage of WS with coexposure, most frequent agents identified in WS with coexposure (number of WS in parentheses), and most frequent mixture of agents across WS (number of WS in parentheses)

| Category | Task group | N WS | % Coex | Most frequent agents | Most frequent mixture |
| --- | --- | --- | --- | --- | --- |
| Machining, assembling, welding, bonding, chain assembly lines | Arc welding | 660 | 70% | Manganese (N=369)/ Iron (N=363)/ Copper (N=260)/ Zinc (N=238)/ Nickel (N=231) | Iron,Manganese (N=339) |
| Control, sterilization, cleaning, repair | Area cleaning | 224 | 24.1% | Quartz (N=25)/ Lead (N=20)/ Cristobalite (N=13)/ Hexavalent chromium (N= 8)/ Wood dust (N= 8) | Cristobalite,Quartz (N=13) |
|  | Assembling | 992 | 20.6% | Toluene (N=56)/ Butanone (N=54)/ Xylene (N=53)/ Acetone (N=48)/ Ethylbenzene (N=39) | Ethylbenzene,Xylene (N=38) |
| Bottling, filling, winding, dosing | Bagging (including packing in crates, big bags, etc.) | 347 | 24.2% | Quartz (N=47)/ Cristobalite (N=35)/ Butanone (N=13)/ Toluene (N=11)/ Acetone (N=10) | Cristobalite,Quartz (N=34) |
|  | Bottling | 83 | 48.2% | Butanone (N=16)/ Toluene (N=14)/ Acetone (N=13)/ Xylene (N= 9)/ Ethanol (N= 7) | Toluene,Xylene (N=7) |
|  | Bottling, filling, winding, dosing, warehousing, loading; other tasks n.e.c. | 6 | 16.7% | 1,2,4-Trimethylbenzene (N=1)/ Benzene (N=1)/ Ethylbenzene (N=1)/ Lead (N=1)/ Toluene (N=1) | 1,2,4-Trimethylbenzene,Benzene,Ethylbenzene,Lead,Toluene,Xylene (N=1) |
| Preparation, treatment, protection | Chemical treatment and protection of surfaces | 539 | 39.1% | Hexavalent chromium (N=59)/ Acetone (N=44)/ Hydrogen chloride (N=39)/ Hydrogen fluoride (N=36)/ Toluene (N=36) | Ethylbenzene,Xylene (N=22) |
|  | Cleaning of materials or finished parts | 610 | 33.3% | Acetone (N=82)/ Butanone (N=54)/ Xylene (N=54)/ Toluene (N=53)/ 1-Methoxypropan-2-ol (N=41) | Ethylbenzene,Xylene (N=31) |
|  | Cleaning of tools and machines | 719 | 26.4% | Toluene (N=45)/ Xylene (N=45)/ Acetone (N=39)/ Butanone (N=32)/ Quartz (N=31) | Toluene,Xylene (N=23) |
|  | Coating | 112 | 51.8% | Butanone (N=31)/ Toluene (N=31)/ Acetone (N=15)/ Xylene (N=12)/ Ethyl acetate (N= 9) | Butanone,Toluene (N=22) |
| Metallurgy and primary transformation of ferrous and non-ferrous metals | Coke oven plants and ancillary units | 59 | 40.7% | Benzene (N=21)/ Ammonia, anhydrous (N=11)/ Toluene (N=11)/ Benz[a]anthracene (N= 6)/ Benzo(e)acephenanthrylene (N= 6) | Ammonia, anhydrous,Benzene (N=11) |
| Collection and treatment of waste and water | Collection and treatment of waste | 568 | 41.7% | Lead (N=113)/ Quartz (N= 53)/ Toluene (N= 51)/ Ammonia, anhydrous (N= 48)/ Xylene (N= 44) | Toluene,Xylene (N=39) |
| Construction and public works | Demolition work | 102 | 37.3% | Lead (N=26)/ Iron (N=25)/ Zinc (N=19)/ Quartz (N=17)/ Nickel (N=15) | Iron,Zinc (N=19) |
| Textile | Dry cleaning | 67 | 29.9% | Tetrachloroethylene (N=14)/ Aliphatic and alicyclic hydrocarbons (N= 7)/ N-butyl acetate (N= 6)/ Ethanol (N= 4)/ Toluene (N= 4) | Aliphatic and alicyclic hydrocarbons,Tetrachloroethylene (N=7) |
| Operating and monitoring of melting, sintering, grilling, drying, casting facilities | Dryers | 87 | 17.2% | Quartz (N=8)/ Cristobalite (N=6)/ Toluene (N=4)/ Tridymite (N=4)/ Xylene (N=4) | Cristobalite,Quartz (N=6) |
|  | Dyeing | 15 | 26.7% | 1-Methoxypropan-2-ol (N=3)/ Ethylbenzene (N=2)/ Xylene (N=2)/ (2-Methoxymethylethoxy)propanol (N=1)/ 1,2,4-Trimethylbenzene (N=1) | Ethylbenzene,Xylene (N=2) |
| Printing, serigraphy, reproduction of documents | Electronic printing processes | 17 | 52.9% | 2-Butoxyethyl acetate (N=3)/ 2-Methoxy-1-methylethyl acetate (N=3)/ Cyclohexanone (N=3)/ Xylene (N=3)/ 1-Methoxypropan-2-ol (N=2) | 2-Butoxyethyl acetate,Cyclohexanone (N=3) |
|  | Extraction or drilling work | 332 | 39.2% | Quartz (N=118)/ Cristobalite (N= 91)/ Tridymite (N= 24)/ Hexavalent chromium (N= 13)/ Diesel exhaust, elemental carbon (N= 7) | Cristobalite,Quartz (N=90) |
|  | Fabrication of electrodes for electrometallurgy | 21 | 14.3% | Barium (N=2)/ Chlorine (N=2)/ Cristobalite (N=1)/ Hydrogen chloride (N=1)/ Lead (N=1) | Barium,Chlorine (N=2) |
|  | Filling | 94 | 47.9% | Xylene (N=20)/ Toluene (N=18)/ Acetone (N=14)/ Ethylbenzene (N=12)/ Butanone (N=11) | Toluene,Xylene (N=13) |
|  | Finishing and maintenance of buildings | 462 | 16.2% | Quartz (N=23)/ Lead (N=19)/ Xylene (N=18)/ Cristobalite (N=12)/ Ethylbenzene (N=12) | Cristobalite,Quartz (N=12) |
| Foundry | Finishing of foundry molds and cores | 337 | 24% | Quartz (N=29)/ Cristobalite (N=17)/ Butanone (N=15)/ Xylene (N=15)/ Toluene (N=13) | Cristobalite,Quartz (N=17) |
|  | Flexography | 51 | 60.8% | Ethanol (N=22)/ Ethyl acetate (N=22)/ 1-Methoxypropan-2-ol (N=13)/ Propan-2-ol (N=12)/ Butanone (N=11) | Ethanol,Ethyl acetate (N=21) |
| Agriculture and agri-food industries | Food industries | 91 | 44% | Formaldehyde (N=11)/ Acetaldehyde (N=10)/ N-hexane (N= 9)/ Benz[a]anthracene (N= 5)/ Diethyl ether (N= 5) | Acetaldehyde,Formaldehyde (N=9) |
|  | Foundries, other tasks n.e.c. | 120 | 30% | Lead (N=21)/ Quartz (N=15)/ Hexavalent chromium (N=10)/ Iron (N= 8)/ Copper (N= 7) | Lead,Quartz (N=9) |
| Storage and transport operations of raw materials / intermediate products / finished products | Fuel distribution | 41 | 48.8% | Toluene (N=19)/ Benzene (N=12)/ Xylene (N=10)/ N-hexane (N= 9)/ Ethylbenzene (N= 7) | Benzene,Toluene (N=12) |
|  | Gas welding or brazing (torch) | 146 | 37.7% | Iron (N=27)/ Copper (N=22)/ Zinc (N=20)/ Lead (N=17)/ Manganese (N=16) | Iron,Manganese (N=16) |
|  | Gluing | 406 | 46.3% | Butanone (N=104)/ Acetone (N= 88)/ Toluene (N= 75)/ Aliphatic and alicyclic hydrocarbons (N= 40)/ Ethyl acetate (N= 39) | Acetone,Butanone (N=57) |
| Special cases | Hair salons / nails salons | 7 | 85.7% | Acetone (N=6)/ Ethanol (N=6)/ Ethyl acetate (N=4)/ N-butyl acetate (N=4)/ Propan-2-ol (N=4) | Acetone,Ethanol (N=6) |
|  | Hopper silos loading / unloading operations | 1000 | 24.5% | Quartz (N=109)/ Cristobalite (N= 90)/ Xylene (N= 53)/ Toluene (N= 41)/ Lead (N= 38) | Cristobalite,Quartz (N=88) |
| Hospitals, medical and diagnostic laboratories | Hospitals, medical and diagnostic laboratories | 336 | 48.2% | Ethanol (N=76)/ Formaldehyde (N=60)/ Xylene (N=53)/ Toluene (N=52)/ Sevoflurane (N=37) | Ethanol,Formaldehyde (N=38) |
|  | Impregnation of surfaces or core (wood, bricks) | 66 | 19.7% | Xylene (N=5)/ Butanone (N=4)/ Ethylbenzene (N=4)/ Phenol (N=4)/ Acetone (N=3) | Ethylbenzene,Xylene (N=4) |
|  | Inspecting, cleaning, repairing; other tasks n.e.c. | 911 | 25.5% | Toluene (N=63)/ Quartz (N=58)/ Acetone (N=47)/ Xylene (N=47)/ Benzene (N=33) | Cristobalite,Quartz (N=31) |
|  | Knitting and sewing | 20 | 10% | Acetone (N=1)/ Aliphatic and alicyclic hydrocarbons (N=1)/ Butanone (N=1)/ Ethyl acetate (N=1)/ Iron (N=1) | Acetone,Aliphatic and alicyclic hydrocarbons,Butanone,Ethyl acetate,Propyl acetate,Toluene (N=1) |
|  | Machining | 3415 | 17.1% | Iron (N=185)/ Manganese (N=136)/ Metalworking fluids (inhalable fraction) (N=120)/ Water-soluble metalworking fluids (N=108)/ Quartz (N=107) | Iron,Manganese (N=126) |
|  | Machining, assembling, welding, gluing, chain assembling: other tasks n.e.c. | 2068 | 20% | Acetone (N=92)/ Butanone (N=92)/ Quartz (N=75)/ Chromium (metal and inorganic Cr(II)/Cr(III) compounds) (N=71)/ Xylene (N=65) | Chromium (metal and inorganic Cr(II)/Cr(III) compounds),Hexavalent chromium (N=42) |
|  | Malting plants, breweries | 1 | 0% |  |  |
| Mixing, molding, compression, reaction, rolling, calendering | Manufacture of composite material parts | 386 | 63.7% | Styrene (N=188)/ Acetone (N=182)/ Methyl methacrylate (N= 69)/ Butanone (N= 59)/ Aliphatic and alicyclic hydrocarbons (N= 43) | Acetone,Styrene (N=175) |
|  | Manufacture of cores | 67 | 40.3% | Quartz (N=21)/ Phenol (N=12)/ Cristobalite (N=10)/ Formaldehyde (N= 9)/ Aluminium (N= 3) | Phenol,Quartz (N=12) |
|  | Manufacture of foundry molds | 87 | 46% | Quartz (N=29)/ Cristobalite (N=16)/ Phenol (N=11)/ Formaldehyde (N= 9)/ Tridymite (N= 6) | Cristobalite,Quartz (N=16) |
|  | Manufacturing control, sterilization (except medical field) | 1156 | 31.7% | Quartz (N=88)/ Xylene (N=79)/ Acetone (N=76)/ Toluene (N=75)/ Cristobalite (N=56) | Cristobalite,Quartz (N=53) |
|  | Manufacturing of artificial mineral fibers | 60 | 23.3% | Ammonia, anhydrous (N=12)/ Phenol (N= 9)/ Quartz (N= 5)/ Cristobalite (N= 3)/ Refractory ceramic fibers (L>5um D<3um) (N= 3) | Ammonia, anhydrous,Phenol (N=9) |
|  | Manufacturing of porcelain, ceramic and pottery products | 82 | 35.4% | Quartz (N=23)/ Cristobalite (N=16)/ Lead (N= 9)/ Cadmium (N= 3)/ Cobalt (N= 2) | Cristobalite,Quartz (N=16) |
|  | Mechanical surface treatment | 1006 | 26.8% | Iron (N=92)/ Quartz (N=70)/ Manganese (N=67)/ Nickel (N=58)/ Zinc (N=54) | Iron,Manganese (N=58) |
|  | Melting, sintering, grilling, drying, casting; other tasks n.e.c. | 9 | 11.1% | Cristobalite (N=1)/ Quartz (N=1) | Cristobalite,Quartz (N=1) |
|  | Metal making and casting | 556 | 33.6% | Quartz (N=104)/ Lead (N= 71)/ Cristobalite (N= 54)/ Refractory ceramic fibers (L>5um D<3um) (N= 40)/ Hexavalent chromium (N= 39) | Cristobalite,Quartz (N=53) |
|  | Metallurgy and primary transformation of ferrous and non-ferrous metals, other tasks n.e.c. | 167 | 38.9% | Lead (N=41)/ Quartz (N=24)/ Chromium (metal and inorganic Cr(II)/Cr(III) compounds) (N=19)/ Hexavalent chromium (N=18)/ Cristobalite (N=15) | Chromium (metal and inorganic Cr(II)/Cr(III) compounds),Lead (N=18) |
| Operating and monitoring of milling and sorting facilities | Milling and sorting; other tasks n.e.c. | 18 | 16.7% | Cristobalite (N=2)/ Quartz (N=2)/ Hydrogen chloride (N=1)/ Lead (N=1) | Cristobalite,Quartz (N=2) |
|  | Miscellaneous | 7 | 42.9% | Benz[a]anthracene (N=1)/ Benzo(e)acephenanthrylene (N=1)/ Benzo(k)fluoranthene (N=1)/ Benzo[def]chrysene (N=1)/ Benzo[ghi]perylene (N=1) | Benz[a]anthracene,Benzo(e)acephenanthrylene,Benzo(k)fluoranthene,Benzo[def]chrysene,Benzo[ghi]perylene (N=1) |
|  | Miscellaneous printing tasks | 25 | 48% | 1,2,4-Trimethylbenzene (N=7)/ Mesitylene (N=6)/ 2-Butoxyethyl acetate (N=5)/ Butanone (N=4)/ 1,2,3-Trimethylbenzene (N=3) | 1,2,4-Trimethylbenzene,Mesitylene (N=6) |
|  | Mixing | 439 | 45.1% | Quartz (N=75)/ Butanone (N=51)/ Xylene (N=50)/ Toluene (N=43)/ Ethylbenzene (N=42) | Ethylbenzene,Xylene (N=41) |
|  | Mixing, compression, molding, reaction; other tasks n.e.c. | 10 | 20% | Acetone (N=1)/ Butyl acrylate (N=1)/ Cadmium (N=1)/ Methyl methacrylate (N=1)/ Zinc (N=1) | Acetone,Butyl acrylate,Methyl methacrylate (N=1) |
|  | Molding, pelletizing, calendering, rolling facilities | 291 | 36.4% | Quartz (N=40)/ Cristobalite (N=25)/ Tridymite (N=18)/ Iron (N=14)/ Styrene (N=14) | Cristobalite,Quartz (N=24) |
|  | Offset printing | 83 | 65.1% | Propan-2-ol (N=22)/ Aliphatic and alicyclic hydrocarbons (N=17)/ Toluene (N=13)/ 1,2,4-Trimethylbenzene (N=11)/ Butanone (N=11) | Aliphatic and alicyclic hydrocarbons,Propan-2-ol (N=12) |
|  | Operating and monitoring of milling and sorting facilities | 946 | 24.9% | Quartz (N=110)/ Cristobalite (N= 78)/ Lead (N= 69)/ Toluene (N= 46)/ Xylene (N= 44) | Cristobalite,Quartz (N=76) |
|  | Operating handling trolleys, road machinery and other vehicles | 942 | 26.5% | Quartz (N=143)/ Cristobalite (N=100)/ Lead (N= 60)/ Wood dust (N= 36)/ Hexavalent chromium (N= 34) | Cristobalite,Quartz (N=97) |
|  | Other construction tasks, n.e.c. | 239 | 24.3% | Quartz (N=41)/ Cristobalite (N=25)/ Lead (N=11)/ Toluene (N=11)/ Hexavalent chromium (N=10) | Cristobalite,Quartz (N=25) |
| Leather industry | Other leather industry tasks n.e.c. | 21 | 33.3% | Acetone (N=4)/ Cyclohexane (N=3)/ Butanone (N=2)/ Ethyl acetate (N=2)/ N-hexane (N=2) | Acetone,Butanone,Cyclohexane,N-hexane (N=2) |
|  | Other particular work n.e.c. | 318 | 13.8% | Quartz (N=11)/ Xylene (N=11)/ Tridymite (N=10)/ Toluene (N= 9)/ Acetone (N= 6) | Quartz,Tridymite (N=8) |
|  | Other printing processes | 29 | 41.4% | Ethylbenzene (N=5)/ Xylene (N=5)/ 1-Methoxypropan-2-ol (N=4)/ 1,2,4-Trimethylbenzene (N=4)/ Acetone (N=4) | Ethylbenzene,Xylene (N=4) |
|  | Other welding processes | 207 | 28% | Iron (N=28)/ Zinc (N=18)/ Copper (N=17)/ Lead (N=16)/ Hexavalent chromium (N=13) | Iron,Zinc (N=17) |
|  | Ovens (excluding foundries) | 277 | 28.5% | Quartz (N=38)/ Cristobalite (N=26)/ Refractory ceramic fibers (L>5um D<3um) (N=25)/ Lead (N=15)/ Hexavalent chromium (N=14) | Cristobalite,Quartz (N=24) |
|  | Packaging, palletization | 322 | 11.5% | Quartz (N=11)/ Cristobalite (N=10)/ Acetone (N= 6)/ Styrene (N= 6)/ Ethylbenzene (N= 5) | Cristobalite,Quartz (N=10) |
|  | Papermaking | 32 | 34.4% | Ammonia, anhydrous (N=5)/ Wood dust (N=5)/ 2-Butoxyethyl acetate (N=4)/ 1,2,4-Trimethylbenzene (N=3)/ Acetaldehyde (N=2) | Ammonia, anhydrous,Wood dust (N=5) |
|  | Photogravure | 239 | 33.9% | Ethyl acetate (N=30)/ Ethanol (N=28)/ Butanone (N=24)/ Toluene (N=23)/ Xylene (N=19) | Ethanol,Ethyl acetate (N=28) |
|  | Press control and surveillance, extruder, injection molding machines | 413 | 32.7% | Formaldehyde (N=45)/ Xylene (N=28)/ Toluene (N=27)/ Acetaldehyde (N=23)/ Ethylbenzene (N=23) | Acetaldehyde,Formaldehyde (N=23) |
|  | Printing: other tasks n.e.c. | 78 | 56.4% | Ethanol (N=17)/ Propan-2-ol (N=16)/ Butanone (N=15)/ Ethyl acetate (N=13)/ 2-Methoxy-1-methylethyl acetate (N=11) | Ethanol,Propan-2-ol (N=13) |
|  | Protection and treatment of surfaces by application of paints, varnishes, powders, release agents | 1609 | 57.4% | Xylene (N=587)/ Ethylbenzene (N=480)/ Toluene (N=392)/ Butanone (N=311)/ Acetone (N=304) | Ethylbenzene,Xylene (N=466) |
|  | Public safety, protection and emergency services | 1 | 0% |  |  |
|  | Reaction (polymerization units, distillation, extraction, desulfurization, extraction plants, etc.) | 495 | 48.9% | Toluene (N=121)/ Xylene (N= 75)/ Benzene (N= 73)/ N-hexane (N= 68)/ Ammonia, anhydrous (N= 58) | Toluene,Xylene (N=58) |
|  | Repair, maintenance, inspection | 2251 | 36.8% | Quartz (N=256)/ Toluene (N=214)/ Xylene (N=199)/ Benzene (N=167)/ Lead (N=140) | Cristobalite,Quartz (N=133) |
|  | Roadwork - sealing of structures and basins | 112 | 25.9% | Quartz (N=14)/ Cristobalite (N=10)/ Bitumen fumes (N= 9)/ Ethylbenzene (N= 5)/ Pyrene (N= 5) | Cristobalite,Quartz (N=9) |
|  | Sand regeneration facilities | 45 | 31.1% | Quartz (N=12)/ Cristobalite (N= 9)/ Lead (N= 2)/ Phenol (N= 2)/ 1-Methoxypropan-2-ol (N= 1) | Cristobalite,Quartz (N=9) |
|  | Screen printing | 150 | 64.7% | Acetone (N=40)/ Toluene (N=39)/ 2-Methoxy-1-methylethyl acetate (N=34)/ Cyclohexanone (N=34)/ Xylene (N=30) | 2-Methoxy-1-methylethyl acetate,Cyclohexanone (N=21) |
|  | Shared services, areas without specific pollution | 320 | 11.6% | Quartz (N=23)/ Cristobalite (N=20)/ Toluene (N= 6)/ Xylene (N= 6)/ Ethylbenzene (N= 5) | Cristobalite,Quartz (N=20) |
|  | Shared services, general areas with specific pollution n.e.c. | 1280 | 45.1% | Acetone (N=245)/ Toluene (N=200)/ N-hexane (N=143)/ Chloroform (N=110)/ Xylene (N=103) | Acetone,Toluene (N=108) |
|  | Shooting ranges, pyrotechnics | 101 | 9.9% | Lead (N=9)/ Quartz (N=6)/ Acetone (N=2)/ Heptane (N=2)/ Iron (N=2) | Lead,Quartz (N=6) |
|  | Social work | 1 | 0% |  |  |
|  | Spinning mills | 348 | 33.3% | Xylene (N=42)/ Quartz (N=39)/ Ethylbenzene (N=30)/ Cristobalite (N=25)/ Toluene (N=22) | Ethylbenzene,Xylene (N=28) |
|  | Storage and transport: other tasks n.e.c. | 16 | 6.2% | Pentane (N=1)/ Toluene (N=1) | Pentane,Toluene (N=1) |
|  | Structural work | 374 | 19.5% | Quartz (N=49)/ Cristobalite (N=25)/ Refractory ceramic fibers (L>5um D<3um) (N=16)/ Lead (N=11)/ Hexavalent chromium (N=10) | Cristobalite,Quartz (N=23) |
|  | Surface preparation, treatment, protection; other tasks n.e.c. | 14 | 35.7% | Lead (N=2)/ Acetonitrile (N=1)/ Butanone (N=1)/ Ethylbenzene (N=1)/ Hydrogen chloride (N=1) | Acetonitrile,Methanol (N=1) |
|  | Tanning and tawing | 54 | 48.1% | 1-Methoxypropan-2-ol (N=11)/ Xylene (N=10)/ Butanone (N= 9)/ Acetone (N= 6)/ Ethylbenzene (N= 6) | Butanone,Xylene (N=5) |
|  | Textile industry: other tasks n.e.c. | 48 | 52.1% | Toluene (N=12)/ Ammonia, anhydrous (N= 9)/ Butanone (N= 7)/ Aliphatic and alicyclic hydrocarbons (N= 6)/ Acetone (N= 4) | Ammonia, anhydrous,Toluene (N=9) |
|  | Thermal or thermo-chemical surface treatment | 153 | 37.9% | Lead (N=15)/ Xylene (N=15)/ Hydrogen fluoride (N=14)/ Ethylbenzene (N=12)/ Hydrogen chloride (N=11) | Ethylbenzene,Xylene (N=12) |
|  | Thermal stations, swimming pools | 7 | 0% |  |  |
|  | Thermometrics | 2 | 0% |  |  |
|  | Toll booth of car parks, highways, fuel distribution | 33 | 21.2% | Toluene (N=7)/ 1,2,4-Trimethylbenzene (N=3)/ Benzene (N=3)/ Ethylbenzene (N=3)/ Xylene (N=3) | 1,2,4-Trimethylbenzene,Toluene (N=3) |
|  | Transport of raw materials / intermediate products / finished products | 1063 | 36.6% | Toluene (N=161)/ Quartz (N=105)/ Xylene (N= 96)/ Benzene (N= 90)/ Cristobalite (N= 70) | Toluene,Xylene (N=85) |
|  | Treatment and electrochemical protection of surfaces | 81 | 39.5% | Hexavalent chromium (N=18)/ Nickel (N=15)/ Total chromium (N=10)/ Copper (N= 9)/ Iron (N= 8) | Hexavalent chromium,Nickel (N=11) |
|  | Viticulture | 6 | 33.3% | Cristobalite (N=2)/ Quartz (N=2) | Cristobalite,Quartz (N=2) |
|  | Water collection and treatment | 126 | 41.3% | Toluene (N=26)/ Hydrogen sulphide (N=19)/ Benzene (N=18)/ Xylene (N=18)/ Ammonia, anhydrous (N=16) | Toluene,Xylene (N=16) |
|  | Weaving | 9 | 0% |  |  |
|  | Weighing, dosing | 226 | 34.5% | Quartz (N=20)/ Toluene (N=17)/ Xylene (N=16)/ Ethylbenzene (N=12)/ 2-Methoxy-1-methylethyl acetate (N=11) | Cristobalite,Quartz (N=11) |
|  | Winding | 101 | 31.7% | Butanone (N=6)/ Lead (N=5)/ Quartz (N=5)/ Xylene (N=5)/ Acetone (N=4) | Cristobalite,Quartz (N=4) |

## Table S4: List of industry sectors, carcinogens

List of industry sectors with exposure to at least one carcinogen, number of work situations, percentage of work situation with coexposure, most frequent agents among work situation with coexposure, most frequent mixture of agents across work situations

| Division | Group | N WS | % Coex | Most frequent agents | Most frequent mixture |
| --- | --- | --- | --- | --- | --- |
| 01: Crop and animal production, hunting and related service activities | 011: Growing of non-perennial crops | 16 | 25% | Quartz (N=2)/ Wood dust (N=2)/ Acetone (N=1)/ Acetonitrile (N=1)/ Iron (N=1) | Quartz,Wood dust (N=2) |
|  | 012: Growing of perennial crops | 12 | 8.3% | Dinitrogen oxide (N=1)/ Formaldehyde (N=1)/ Peracetic acid (N=1) | Dinitrogen oxide,Formaldehyde,Peracetic acid (N=1) |
|  | 013: Plant propagation | 2 | 50% | Quartz (N=1)/ Tridymite (N=1) | Quartz,Tridymite (N=1) |
|  | 014: Animal production | 3 | 0% |  |  |
|  | 015: Mixed farming | 2 | 50% | Chromium (metal and inorganic Cr(II)/Cr(III) compounds) (N=1)/ Copper (N=1)/ Diiron trioxide (N=1)/ Manganese (N=1)/ Nickel (N=1) | Chromium (metal and inorganic Cr(II)/Cr(III) compounds),Copper,Diiron trioxide,Manganese,Nickel,Welding fumes (N=1) |
|  | 016: Support activities to agriculture and post-harvest crop activities | 4 | 0% |  |  |
| 02: Forestry and logging | 021: Silviculture and other forestry activities | 3 | 0% |  |  |
|  | 022: Logging | 167 | 0% |  |  |
|  | 024: Support services to forestry | 51 | 2% | 2-Aminoethanol (N=1)/ N-hexane (N=1)/ Toluene (N=1)/ Wood dust (N=1) | 2-Aminoethanol,N-hexane,Toluene,Wood dust (N=1) |
| 03: Fishing and aquaculture | 031: Fishing | 2 | 0% |  |  |
| 06: Extraction of crude petroleum and natural gas | 062: Extraction of natural gas | 2 | 50% | Benzene (N=1)/ Toluene (N=1) | Benzene,Toluene (N=1) |
| 07: Mining of metal ores | 072: Mining of non-ferrous metal ores | 7 | 0% |  |  |
| 08: Other mining and quarrying | 081: Quarrying of stone, sand and clay | 1380 | 19.9% | Quartz (N=270)/ Cristobalite (N=243)/ Tridymite (N= 53)/ Lead (N= 9)/ Carbon dioxide (N= 6) | Cristobalite,Quartz (N=243) |
|  | 089: Mining and quarrying n.e.c. | 82 | 13.4% | Quartz (N=6)/ Cristobalite (N=4)/ Ammonia, anhydrous (N=2)/ Chloroethylene (N=2)/ Chromium (metal and inorganic Cr(II)/Cr(III) compounds) (N=2) | Cristobalite,Quartz (N=4) |
| 09: Mining support service activities | 091: Support activities for petroleum and natural gas extraction | 4 | 75% | Benzene (N=2)/ Cyclohexane (N=2)/ Ethylbenzene (N=2)/ Heptane (N=2)/ N-hexane (N=2) | Benzene,Cyclohexane,Ethylbenzene,Heptane,N-hexane,Toluene,Xylene (N=2) |
|  | 099: Support activities for other mining and quarrying | 2 | 0% |  |  |
| 10: Manufacture of food products | 101: Processing and preserving of meat and production of meat products | 28 | 39.3% | Formaldehyde (N=8)/ Acetaldehyde (N=7)/ Ammonia, anhydrous (N=2)/ Benz[a]anthracene (N=2)/ Benzo(e)acephenanthrylene (N=1) | Acetaldehyde,Formaldehyde (N=6) |
|  | 102: Processing and preserving of fish, crustaceans and molluscs | 4 | 75% | Acetaldehyde (N=1)/ Aliphatic and alicyclic hydrocarbons (N=1)/ Benz[a]anthracene (N=1)/ Benzo(k)fluoranthene (N=1)/ Benzo[def]chrysene (N=1) | Acetaldehyde,Formaldehyde (N=1) |
|  | 103: Processing and preserving of fruit and vegetables | 14 | 14.3% | Hydrogen fluoride (N=1)/ Metalworking fluids (inhalable fraction) (N=1)/ Nitric acid (N=1)/ Water-soluble metalworking fluids (N=1) | Hydrogen fluoride,Nitric acid (N=1) |
|  | 104: Manufacture of vegetable and animal oils and fats | 50 | 70% | N-hexane (N=26)/ Diethyl ether (N=12)/ Ethanol (N=11)/ Aliphatic and alicyclic hydrocarbons (N= 9)/ Acetone (N= 7) | Diethyl ether,N-hexane (N=10) |
|  | 105: Manufacture of dairy products | 39 | 20.5% | N-hexane (N=4)/ Diethyl ether (N=3)/ Orthophosphoric acid (N=2)/ Sulphuric acid (N=2)/ Ammonia, anhydrous (N=1) | Diethyl ether,N-hexane (N=3) |
|  | 106: Manufacture of grain mill products, starches and starch products | 18 | 50% | Acetone (N=4)/ Chloroform (N=4)/ Cristobalite (N=4)/ N-hexane (N=4)/ Quartz (N=4) | Acetone,Chloroform (N=4) |
|  | 107: Manufacture of bakery and farinaceous products | 34 | 50% | Butanone (N=11)/ Acetone (N= 4)/ Pentane (N= 4)/ N-hexane (N= 3)/ Acetaldehyde (N= 2) | Acetone,Butanone (N=4) |
|  | 108: Manufacture of other food products | 62 | 21% | N-hexane (N=5)/ Acetone (N=3)/ Butanone (N=3)/ Toluene (N=3)/ Aliphatic and alicyclic hydrocarbons (N=2) | Acetone,N-hexane (N=2) |
|  | 109: Manufacture of prepared animal feeds | 56 | 44.6% | Manganese (N=20)/ Zinc (N=17)/ Iron (N=11)/ Cobalt (N= 8)/ Copper (N= 6) | Manganese,Zinc (N=17) |
| 11: Manufacture of beverages | 110: Manufacture of beverages | 104 | 26.9% | Butanone (N=10)/ Xylene (N= 7)/ 2-Butoxyethanol (N= 6)/ Ethylbenzene (N= 6)/ Acetone (N= 4) | Ethylbenzene,Xylene (N=6) |
| 12: Manufacture of tobacco products | 120: Manufacture of tobacco products | 2 | 50% | Aliphatic and alicyclic hydrocarbons (N=1)/ Ethanol (N=1)/ Ethyl acetate (N=1) | Aliphatic and alicyclic hydrocarbons,Ethanol,Ethyl acetate (N=1) |
| 13: Manufacture of textiles | 131: Preparation and spinning of textile fibres | 24 | 75% | Ammonia, anhydrous (N=10)/ Toluene (N=10)/ Quartz (N= 6)/ Cristobalite (N= 5)/ Boron (N= 2) | Ammonia, anhydrous,Toluene (N=10) |
|  | 132: Weaving of textiles | 31 | 45.2% | Butanone (N=7)/ Toluene (N=6)/ Xylene (N=5)/ 1-Methoxypropan-2-ol (N=3)/ Acetone (N=3) | Butanone,Toluene (N=5) |
|  | 133: Finishing of textiles | 12 | 41.7% | Acetone (N=2)/ 1-Methoxypropan-2-ol (N=1)/ 1,2,3-Trimethylbenzene (N=1)/ 1,2,4-Trimethylbenzene (N=1)/ 2-Methoxy-1-methylethyl acetate (N=1) | 1-Methoxypropan-2-ol,4-Hydroxy-4-methylpentan-2-one,Cyclohexanone,Monocyclic aromatic hydrocarbons (N=1) |
|  | 139: Manufacture of other textiles | 74 | 37.8% | Butanone (N=14)/ Toluene (N=14)/ Acetone (N= 9)/ Ethyl acetate (N= 6)/ Xylene (N= 5) | Butanone,Toluene (N=9) |
| 14: Manufacture of wearing apparel | 141: Manufacture of wearing apparel, except fur apparel | 8 | 62.5% | Ethyl acetate (N=4)/ Acetone (N=3)/ Aliphatic and alicyclic hydrocarbons (N=2)/ Butanone (N=2)/ C6-C12 hydrocarbons (N=2) | Acetone,Ethyl acetate (N=3) |
|  | 143: Manufacture of knitted and crocheted apparel | 3 | 0% |  |  |
| 15: Manufacture of leather and related products | 151: Tanning and dressing of leather: manufacture of luggage, handbags, saddlery and harness: dressing and dyeing of fur | 95 | 44.2% | Acetone (N=22)/ Butanone (N=11)/ Cyclohexane (N= 9)/ Iron (N= 8)/ 1-Methoxypropan-2-ol (N= 7) | Acetone,Butanone (N=11) |
|  | 152: Manufacture of footwear | 20 | 75% | Acetone (N=11)/ Butanone (N= 7)/ Aliphatic and alicyclic hydrocarbons (N= 6)/ Ethyl acetate (N= 4)/ N-hexane (N= 4) | Acetone,Butanone (N=7) |
| 16: Manufacture of wood and of products of wood and cork, except furniture: manufacture of articles of straw and plaiting materials | 161: Sawmilling and planing of wood | 531 | 1.7% | Wood dust (N=7)/ 1-Methoxypropan-2-ol (N=3)/ Cobalt (N=3)/ Iron (N=3)/ Aliphatic and alicyclic hydrocarbons (N=2) | Cobalt,Iron,Wood dust (N=3) |
|  | 162: Manufacture of products of wood, cork, straw and plaiting materials | 1598 | 6.3% | Wood dust (N=83)/ Lead (N=28)/ Formaldehyde (N=21)/ Toluene (N=16)/ Benz[a]anthracene (N=15) | Lead,Wood dust (N=27) |
| 17: Manufacture of paper and paper products | 171: Manufacture of pulp, paper and paperboard | 233 | 19.7% | Ammonia, anhydrous (N=30)/ Wood dust (N=25)/ Acetone (N= 8)/ Sulphuric acid (N= 6)/ Chromium (metal and inorganic Cr(II)/Cr(III) compounds) (N= 5) | Ammonia, anhydrous,Wood dust (N=24) |
|  | 172: Manufacture of articles of paper and paperboard | 99 | 42.4% | Ethyl acetate (N=16)/ Ethanol (N=14)/ Propan-2-ol (N=13)/ Butanone (N=12)/ Propyl acetate (N= 9) | Ethanol,Ethyl acetate (N=13) |
| 18: Printing and reproduction of recorded media | 181: Printing and service activities related to printing | 152 | 49.3% | Ethanol (N=30)/ Propan-2-ol (N=28)/ Ethyl acetate (N=26)/ Acetone (N=23)/ 1-Methoxypropan-2-ol (N=22) | Ethanol,Ethyl acetate (N=19) |
| 19: Manufacture of coke and refined petroleum products | 191: Manufacture of coke oven products | 13 | 7.7% | 2-Aminoethanol (N=1)/ Ammonia, anhydrous (N=1) | 2-Aminoethanol,Ammonia, anhydrous (N=1) |
|  | 192: Manufacture of refined petroleum products | 368 | 60.9% | Benzene (N=170)/ Toluene (N=170)/ Xylene (N=131)/ Ethylbenzene (N= 93)/ N-hexane (N= 81) | Benzene,Toluene (N=142) |
| 20: Manufacture of chemicals and chemical products | 201: Manufacture of basic chemicals, fertilisers and nitrogen compounds, plastics and synthetic rubber in primary forms | 979 | 37.7% | Toluene (N=129)/ Acetone (N= 95)/ Ammonia, anhydrous (N= 95)/ Benzene (N= 86)/ Chloroethylene (N= 83) | Acetone,Toluene (N=50) |
|  | 202: Manufacture of pesticides and other agrochemical products | 110 | 29.1% | Chlorobenzene (N=10)/ Cyclohexanone (N= 8)/ Methanol (N= 7)/ Toluene (N= 7)/ Ammonia, anhydrous (N= 6) | Ammonia, anhydrous,Chlorobenzene (N=5) |
|  | 203: Manufacture of paints, varnishes and similar coatings, printing ink and mastics | 374 | 59.6% | Xylene (N=137)/ Ethylbenzene (N=107)/ Toluene (N= 84)/ Butanone (N= 83)/ Acetone (N= 63) | Ethylbenzene,Xylene (N=100) |
|  | 204: Manufacture of soap and detergents, cleaning and polishing preparations, perfumes and toilet preparations | 139 | 33.8% | Chloroform (N=15)/ Acetone (N=14)/ Butanone (N=11)/ Hydrogen chloride (N=10)/ Nitric acid (N= 9) | Acetone,Chloroform (N=7) |
|  | 205: Manufacture of other chemical products | 523 | 37.7% | Toluene (N=83)/ Xylene (N=63)/ Acetone (N=51)/ N-hexane (N=44)/ Butanone (N=43) | Toluene,Xylene (N=51) |
|  | 206: Manufacture of man-made fibres | 6 | 16.7% | Chloroform (N=1)/ Phenol (N=1) | Chloroform,Phenol (N=1) |
| 21: Manufacture of basic pharmaceutical products and pharmaceutical preparations | 211: Manufacture of basic pharmaceutical products | 106 | 43.4% | Dichloromethane (N=24)/ Toluene (N=22)/ Acetone (N=21)/ Methanol (N=18)/ Cyclohexane (N=14) | Dichloromethane,Toluene (N=16) |
|  | 212: Manufacture of pharmaceutical preparations | 184 | 33.2% | Acetonitrile (N=24)/ Acetone (N=23)/ Methanol (N=21)/ Chloroform (N=19)/ Dichloromethane (N=19) | Acetonitrile,Methanol (N=18) |
| 22: Manufacture of rubber and plastic products | 221: Manufacture of rubber products | 354 | 43.8% | Toluene (N=71)/ Butanone (N=48)/ N-hexane (N=39)/ Heptane (N=38)/ Cyclohexane (N=36) | Butanone,Toluene (N=32) |
|  | 222: Manufacture of plastics products | 988 | 53% | Acetone (N=184)/ Butanone (N=139)/ Styrene (N=139)/ Xylene (N=123)/ Toluene (N=101) | Acetone,Styrene (N=111) |
| 23: Manufacture of other non-metallic mineral products | 231: Manufacture of glass and glass products | 697 | 31.1% | Quartz (N=109)/ Lead (N= 68)/ Ammonia, anhydrous (N= 52)/ Cristobalite (N= 44)/ Refractory ceramic fibers (L>5um D<3um) (N= 40) | Cristobalite,Quartz (N=41) |
|  | 232: Manufacture of refractory products | 163 | 23.3% | Quartz (N=29)/ Cristobalite (N=22)/ Hexavalent chromium (N= 9)/ Chromium (metal and inorganic Cr(II)/Cr(III) compounds) (N= 6)/ Refractory ceramic fibers (L>5um D<3um) (N= 6) | Cristobalite,Quartz (N=21) |
|  | 233: Manufacture of clay building materials | 860 | 27.2% | Quartz (N=231)/ Cristobalite (N=165)/ Tridymite (N= 64)/ Refractory ceramic fibers (L>5um D<3um) (N= 17)/ Wood dust (N= 12) | Cristobalite,Quartz (N=165) |
|  | 234: Manufacture of other porcelain and ceramic products | 180 | 38.9% | Quartz (N=58)/ Cristobalite (N=52)/ Acetone (N= 7)/ Lead (N= 7)/ Butanone (N= 6) | Cristobalite,Quartz (N=52) |
|  | 235: Manufacture of cement, lime and plaster | 325 | 39.4% | Quartz (N=104)/ Hexavalent chromium (N= 58)/ Cristobalite (N= 55)/ Tridymite (N= 49)/ Iron (N= 14) | Cristobalite,Quartz (N=53) |
|  | 236: Manufacture of articles of concrete, cement and plaster | 698 | 27.4% | Quartz (N=151)/ Cristobalite (N= 91)/ Tridymite (N= 62)/ Hexavalent chromium (N= 16)/ Styrene (N= 14) | Cristobalite,Quartz (N=88) |
|  | 237: Cutting, shaping and finishing of stone | 66 | 43.9% | Cristobalite (N=25)/ Quartz (N=25)/ Toluene (N= 3)/ 1-Methoxypropan-2-ol (N= 1)/ 1,2,4-Trimethylbenzene (N= 1) | Cristobalite,Quartz (N=25) |
|  | 239: Manufacture of abrasive products and non-metallic mineral products n.e.c. | 519 | 33.3% | Quartz (N=105)/ Cristobalite (N=102)/ Ammonia, anhydrous (N= 57)/ Phenol (N= 55)/ Acetone (N= 19) | Cristobalite,Quartz (N=95) |
| 24: Manufacture of basic metals | 241: Manufacture of basic iron and steel and of ferro-alloys | 572 | 42.3% | Lead (N=88)/ Quartz (N=84)/ Hexavalent chromium (N=66)/ Chromium (metal and inorganic Cr(II)/Cr(III) compounds) (N=63)/ Cristobalite (N=48) | Cristobalite,Quartz (N=47) |
|  | 242: Manufacture of tubes, pipes, hollow profiles and related fittings, of steel | 88 | 54.5% | Hexavalent chromium (N=16)/ Acetone (N=13)/ Lead (N=12)/ Butanone (N= 9)/ Manganese (N= 8) | Hexavalent chromium,Lead (N=10) |
|  | 243: Manufacture of other products of first processing of steel | 124 | 28.2% | Butanone (N=11)/ Lead (N=10)/ Xylene (N=10)/ 2-Methoxy-1-methylethyl acetate (N= 7)/ Ethylbenzene (N= 7) | Ethylbenzene,Xylene (N=7) |
|  | 244: Manufacture of basic precious and other non-ferrous metals | 587 | 29% | Lead (N=108)/ Chromium (metal and inorganic Cr(II)/Cr(III) compounds) (N= 79)/ Hexavalent chromium (N= 67)/ Refractory ceramic fibers (L>5um D<3um) (N= 28)/ Hydrogen fluoride (N= 20) | Chromium (metal and inorganic Cr(II)/Cr(III) compounds),Hexavalent chromium (N=56) |
|  | 245: Casting of metals | 734 | 42.9% | Quartz (N=209)/ Cristobalite (N=124)/ Xylene (N= 59)/ Ethylbenzene (N= 54)/ Iron (N= 52) | Cristobalite,Quartz (N=123) |
| 25: Manufacture of fabricated metal products, except machinery and equipment | 251: Manufacture of structural metal products | 285 | 48.1% | Manganese (N=75)/ Iron (N=73)/ Copper (N=51)/ Zinc (N=48)/ Nickel (N=44) | Iron,Manganese (N=68) |
|  | 252: Manufacture of tanks, reservoirs and containers of metal | 103 | 47.6% | Iron (N=23)/ Manganese (N=17)/ Zinc (N=17)/ Copper (N=14)/ Nickel (N=14) | Iron,Manganese (N=17) |
|  | 253: Manufacture of steam generators, except central heating hot water boilers | 19 | 42.1% | 2-Methoxy-1-methylethyl acetate (N=2)/ Chromium (metal and inorganic Cr(II)/Cr(III) compounds) (N=2)/ Cobalt (N=2)/ Copper (N=2)/ Diiron trioxide (N=2) | Chromium (metal and inorganic Cr(II)/Cr(III) compounds),Cobalt,Copper,Diiron trioxide,Hexavalent chromium,Manganese,Nickel (N=2) |
|  | 254: Manufacture of weapons and ammunition | 45 | 15.6% | Lead (N=6)/ Iron (N=4)/ Barium (N=3)/ Butanone (N=1)/ Dichloromethane (N=1) | Iron,Lead (N=4) |
|  | 255: Forging, pressing, stamping and roll-forming of metal: powder metallurgy | 147 | 53.7% | Iron (N=41)/ Manganese (N=32)/ Zinc (N=26)/ Copper (N=20)/ Hexavalent chromium (N=18) | Iron,Manganese (N=31) |
|  | 256: Treatment and coating of metals: machining | 588 | 46.4% | Hexavalent chromium (N=96)/ Iron (N=69)/ Lead (N=63)/ Nickel (N=56)/ Total chromium (N=51) | Iron,Zinc (N=42) |
|  | 257: Manufacture of cutlery, tools and general hardware | 110 | 47.3% | Iron (N=22)/ Cobalt (N=12)/ Total chromium (N=12)/ Copper (N= 9)/ Metalworking fluids (inhalable fraction) (N= 9) | Iron,Total chromium (N=10) |
|  | 259: Manufacture of other fabricated metal products | 466 | 42.5% | Xylene (N=52)/ Iron (N=46)/ Manganese (N=42)/ Ethylbenzene (N=34)/ 2-Methoxy-1-methylethyl acetate (N=29) | Iron,Manganese (N=41) |
| 26: Manufacture of computer, electronic and optical products | 261: Manufacture of electronic components and boards | 310 | 41% | Xylene (N=51)/ Ethylbenzene (N=38)/ Toluene (N=38)/ Butanone (N=37)/ Acetone (N=32) | Ethylbenzene,Xylene (N=38) |
|  | 262: Manufacture of computers and peripheral equipment | 1 | 100% | Cristobalite (N=1)/ Quartz (N=1) | Cristobalite,Quartz (N=1) |
|  | 263: Manufacture of communication equipment | 45 | 33.3% | Butanone (N=8)/ 2-Methoxy-1-methylethyl acetate (N=7)/ Toluene (N=7)/ 1-Methoxypropan-2-ol (N=4)/ Chromium (metal and inorganic Cr(II)/Cr(III) compounds) (N=4) | 2-Methoxy-1-methylethyl acetate,Butanone (N=5) |
|  | 264: Manufacture of consumer electronics | 10 | 30% | Acetone (N=2)/ Butanone (N=1)/ Heptane (N=1)/ Toluene (N=1)/ Xylene (N=1) | Acetone,Heptane (N=1) |
|  | 265: Manufacture of instruments and appliances for measuring, testing and navigation: watches and clocks | 236 | 28.8% | Acetone (N=31)/ Toluene (N=28)/ Xylene (N=26)/ Butanone (N=18)/ Ethylbenzene (N=18) | Toluene,Xylene (N=17) |
|  | 266: Manufacture of irradiation, electromedical and electrotherapeutic equipment | 27 | 63% | Ethylbenzene (N=16)/ Xylene (N=16)/ Hexavalent chromium (N= 1)/ Lead (N= 1) | Ethylbenzene,Xylene (N=16) |
|  | 267: Manufacture of optical instruments and photographic equipment | 16 | 43.8% | Acetone (N=6)/ 4-Methylpentan-2-one (N=2)/ Aliphatic and alicyclic hydrocarbons (N=2)/ Butanone (N=2)/ Diethyl ether (N=2) | 4-Methylpentan-2-one,Acetone,Aliphatic and alicyclic hydrocarbons,Butanone,Ethyl acetate,N-butyl acetate,Propan-2-ol (N=2) |
|  | 268: Manufacture of magnetic and optical media | 5 | 100% | Tetrahydrofuran (N=5)/ Toluene (N=5)/ Butanone (N=3)/ Isopropyl acetate (N=3)/ Cyclohexanone (N=2) | Tetrahydrofuran,Toluene (N=5) |
| 27: Manufacture of electrical equipment | 271: Manufacture of electric motors, generators, transformers and electricity distribution and control apparatus | 270 | 37% | Xylene (N=37)/ Butanone (N=32)/ Ethylbenzene (N=26)/ Toluene (N=22)/ Acetone (N=21) | Ethylbenzene,Xylene (N=25) |
|  | 272: Manufacture of batteries and accumulators | 464 | 3.7% | Lead (N=8)/ Sulphuric acid (N=5)/ Butanone (N=4)/ Heptane (N=4)/ N-hexane (N=4) | Lead,Sulphuric acid (N=5) |
|  | 273: Manufacture of wiring and wiring devices | 145 | 35.2% | Butanone (N=21)/ Acetone (N= 9)/ Toluene (N= 8)/ 4-Methylpentan-2-one (N= 7)/ Cyclohexanone (N= 7) | Butanone,Toluene (N=5) |
|  | 274: Manufacture of electric lighting equipment | 51 | 43.1% | Butanone (N=10)/ Methyl methacrylate (N= 7)/ Xylene (N= 7)/ Acetone (N= 6)/ Cyclohexanone (N= 6) | Acetone,Butanone (N=5) |
|  | 275: Manufacture of domestic appliances | 93 | 48.4% | Quartz (N=19)/ Cristobalite (N=16)/ Iron (N=15)/ Manganese (N= 8)/ Nickel (N= 8) | Cristobalite,Quartz (N=16) |
|  | 279: Manufacture of other electrical equipment | 86 | 30.2% | Hexavalent chromium (N=8)/ Toluene (N=6)/ Lead (N=5)/ Quartz (N=5)/ 2-Methoxy-1-methylethyl acetate (N=4) | 2-Methoxy-1-methylethyl acetate,Toluene (N=4) |
| 28: Manufacture of machinery and equipment n.e.c. | 281: Manufacture of general-purpose machinery | 316 | 51.3% | Xylene (N=36)/ Acetone (N=33)/ Zinc (N=33)/ Iron (N=31)/ Ethylbenzene (N=27) | Ethylbenzene,Xylene (N=27) |
|  | 282: Manufacture of other general-purpose machinery | 261 | 42.5% | Xylene (N=41)/ Ethylbenzene (N=34)/ Iron (N=21)/ Manganese (N=21)/ Acetone (N=20) | Ethylbenzene,Xylene (N=34) |
|  | 283: Manufacture of agricultural and forestry machinery | 67 | 77.6% | Ethylbenzene (N=25)/ Xylene (N=25)/ Iron (N=21)/ Manganese (N=21)/ Mesitylene (N=15) | Ethylbenzene,Xylene (N=25) |
|  | 284: Manufacture of metal forming machinery and machine tools | 26 | 46.2% | Ethylbenzene (N=4)/ Metalworking fluids (inhalable fraction) (N=4)/ Water-soluble metalworking fluids (N=4)/ Xylene (N=4)/ 1,2,4-Trimethylbenzene (N=3) | Ethylbenzene,Xylene (N=4) |
|  | 289: Manufacture of other special-purpose machinery | 163 | 47.2% | Manganese (N=36)/ Nickel (N=36)/ Iron (N=34)/ Total chromium (N=26)/ Copper (N=22) | Manganese,Nickel (N=36) |
| 29: Manufacture of motor vehicles, trailers and semi-trailers | 291: Manufacture of motor vehicles | 179 | 46.9% | Xylene (N=41)/ Ethylbenzene (N=34)/ Quartz (N=24)/ Cristobalite (N=20)/ 1,2,4-Trimethylbenzene (N=17) | Ethylbenzene,Xylene (N=34) |
|  | 292: Manufacture of bodies (coachwork) for motor vehicles: manufacture of trailers and semi-trailers | 252 | 30.6% | Acetone (N=47)/ Toluene (N=41)/ Styrene (N=29)/ Xylene (N=29)/ Ethylbenzene (N=22) | Acetone,Toluene (N=36) |
|  | 293: Manufacture of parts and accessories for motor vehicles | 350 | 33.1% | Xylene (N=39)/ Toluene (N=35)/ Butanone (N=32)/ Acetone (N=24)/ Ethylbenzene (N=17) | Toluene,Xylene (N=22) |
| 30: Manufacture of other transport equipment | 301: Building of ships and boats | 237 | 52.7% | Acetone (N=83)/ Styrene (N=60)/ Methyl methacrylate (N=30)/ Butanone (N=24)/ Chromium (metal and inorganic Cr(II)/Cr(III) compounds) (N=19) | Acetone,Styrene (N=60) |
|  | 302: Manufacture of railway locomotives and rolling stock | 38 | 57.9% | Quartz (N=8)/ 1-Methoxypropan-2-ol (N=7)/ Lead (N=7)/ Xylene (N=7)/ 2-Methoxy-1-methylethyl acetate (N=6) | 1-Methoxypropan-2-ol,2-Methoxy-1-methylethyl acetate (N=6) |
|  | 303: Manufacture of air and spacecraft and related machinery | 668 | 41.3% | Butanone (N=116)/ Acetone (N= 95)/ 2-Methoxy-1-methylethyl acetate (N= 91)/ Hexavalent chromium (N= 88)/ Toluene (N= 84) | 1-Methoxypropan-2-ol,2-Methoxy-1-methylethyl acetate (N=61) |
|  | 304: Manufacture of military fighting vehicles | 8 | 12.5% | Iron (N=1)/ Nickel (N=1)/ Zinc (N=1) | Iron,Nickel,Zinc (N=1) |
|  | 309: Manufacture of transport equipment n.e.c. | 24 | 87.5% | Toluene (N=14)/ Ethylbenzene (N=10)/ Xylene (N=10)/ 1,2,4-Trimethylbenzene (N= 7)/ 2-Methoxy-1-methylethyl acetate (N= 7) | Ethylbenzene,Xylene (N=10) |
| 31: Manufacture of furniture | 310: Manufacture of furniture | 650 | 8.6% | Acetone (N=25)/ Toluene (N=25)/ Xylene (N=21)/ Butanone (N=19)/ Ethylbenzene (N=19) | Acetone,Toluene (N=20) |
| 32: Other manufacturing | 321: Manufacture of jewellery, bijouterie and related articles | 32 | 43.8% | Butanone (N=4)/ Xylene (N=3)/ 1-Methoxypropan-2-ol (N=2)/ 2-Methoxy-1-methylethyl acetate (N=2)/ Acetone (N=2) | Butanone,Xylene (N=3) |
|  | 322: Manufacture of musical instruments | 34 | 17.6% | Butanone (N=3)/ Ethylbenzene (N=3)/ Xylene (N=3)/ Copper (N=2)/ Lead (N=2) | Butanone,Ethylbenzene,Xylene (N=3) |
|  | 323: Manufacture of sports goods | 40 | 32.5% | Acetone (N=8)/ Butanone (N=8)/ Styrene (N=8)/ Methyl methacrylate (N=5)/ Xylene (N=3) | Acetone,Styrene (N=8) |
|  | 324: Manufacture of games and toys | 10 | 20% | 2-Methoxy-1-methylethyl acetate (N=2)/ 1-Methoxypropan-2-ol (N=1)/ Cyclohexanone (N=1)/ Ethylbenzene (N=1)/ Xylene (N=1) | 1-Methoxypropan-2-ol,2-Methoxy-1-methylethyl acetate,Cyclohexanone (N=1) |
|  | 325: Manufacture of medical and dental instruments and supplies | 248 | 36.3% | Acetone (N=22)/ Butanone (N=22)/ Xylene (N=15)/ Nickel (N=14)/ Ethylbenzene (N=13) | Ethylbenzene,Xylene (N=13) |
|  | 329: Manufacturing n.e.c. | 84 | 44% | Butanone (N=19)/ Toluene (N=18)/ Acetone (N=13)/ Xylene (N= 9)/ Ethylbenzene (N= 8) | Butanone,Toluene (N=17) |
| 33: Repair and installation of machinery and equipment | 331: Repair of fabricated metal products, machinery and equipment | 369 | 43.4% | Hexavalent chromium (N=42)/ Iron (N=42)/ Xylene (N=42)/ Toluene (N=40)/ Manganese (N=36) | Iron,Manganese (N=33) |
|  | 332: Installation of industrial machinery and equipment | 117 | 53% | Iron (N=32)/ Manganese (N=29)/ Nickel (N=27)/ Hexavalent chromium (N=25)/ Total chromium (N=23) | Iron,Manganese (N=28) |
| 35: Electricity, gas, steam and air conditioning supply | 351: Electric power generation, transmission and distribution | 220 | 34.5% | Acetone (N=28)/ Toluene (N=21)/ Xylene (N=15)/ Heptane (N=14)/ Ammonia, anhydrous (N=13) | Acetone,Heptane (N=14) |
|  | 352: Manufacture of gas: distribution of gaseous fuels through mains | 9 | 44.4% | Ammonia, anhydrous (N=2)/ Butanone (N=2)/ Hydrogen sulphide (N=2)/ 2-Methylbutane (N=1)/ Aluminium (N=1) | Ammonia, anhydrous,Butanone,Hydrogen sulphide (N=2) |
|  | 353: Steam and air conditioning supply | 204 | 25% | Quartz (N=27)/ Wood dust (N=24)/ Lead (N=16)/ Hexavalent chromium (N=14)/ Cristobalite (N=10) | Quartz,Wood dust (N=17) |
| 36: Water collection, treatment and supply | 360: Water collection, treatment and supply | 40 | 30% | Acetone (N=3)/ N-hexane (N=3)/ Acetonitrile (N=2)/ Ammonia, anhydrous (N=2)/ Chloroform (N=2) | Acetone,N-hexane (N=3) |
| 37: Sewerage | 370: Sewerage | 94 | 34% | Hydrogen sulphide (N=15)/ Ammonia, anhydrous (N=13)/ Toluene (N= 8)/ Acetone (N= 7)/ N-hexane (N= 6) | Ammonia, anhydrous,Hydrogen sulphide (N=10) |
| 38: Waste collection, treatment and disposal activities: materials recovery | 381: Waste collection | 273 | 59.3% | Lead (N=66)/ Toluene (N=64)/ Xylene (N=57)/ Ethylbenzene (N=49)/ Acetone (N=44) | Toluene,Xylene (N=54) |
|  | 382: Waste treatment and disposal | 574 | 60.6% | Lead (N=172)/ Ammonia, anhydrous (N=117)/ Toluene (N=116)/ Xylene (N=110)/ Ethylbenzene (N= 95) | Toluene,Xylene (N=94) |
|  | 383: Materials recovery | 380 | 42.9% | Lead (N=78)/ Quartz (N=41)/ Toluene (N=41)/ Xylene (N=39)/ Hexavalent chromium (N=38) | Toluene,Xylene (N=38) |
| 39: Remediation activities and other waste management services | 390: Remediation activities and other waste management services | 108 | 23.1% | Toluene (N=13)/ Xylene (N=11)/ Ethylbenzene (N=10)/ Benzene (N= 8)/ Lead (N= 8) | Toluene,Xylene (N=11) |
| 41: Construction of buildings | 411: Development of building projects | 2 | 0% |  |  |
|  | 412: Construction of residential and non-residential buildings | 170 | 23.5% | Quartz (N=18)/ Iron (N= 9)/ Manganese (N= 8)/ Cristobalite (N= 7)/ Tridymite (N= 6) | Iron,Manganese (N=8) |
| 42: Civil engineering | 421: Construction of roads and railways | 238 | 33.2% | Quartz (N=53)/ Cristobalite (N=37)/ Iron (N= 9)/ Aluminium (N= 8)/ Bitumen fumes (N= 8) | Cristobalite,Quartz (N=37) |
|  | 422: Construction of utility projects | 42 | 50% | Manganese (N=11)/ Iron (N= 9)/ Quartz (N= 8)/ Nickel (N= 7)/ Copper (N= 6) | Iron,Manganese (N=9) |
|  | 429: Construction of other civil engineering projects | 55 | 32.7% | Quartz (N=7)/ Butanone (N=4)/ Cristobalite (N=4)/ Diesel exhaust, elemental carbon (N=3)/ Iron (N=3) | Cristobalite,Quartz (N=4) |
| 43: Specialised construction activities | 431: Demolition and site preparation | 249 | 27.7% | Quartz (N=43)/ Lead (N=33)/ Iron (N=22)/ Cristobalite (N=16)/ Zinc (N=14) | Iron,Lead (N=18) |
|  | 432: Electrical, plumbing and other construction installation activities | 122 | 30.3% | Iron (N=15)/ Zinc (N=10)/ Copper (N= 9)/ Manganese (N= 8)/ Nickel (N= 7) | Iron,Zinc (N=10) |
|  | 433: Building completion and finishing | 458 | 19.7% | Iron (N=37)/ Lead (N=27)/ Zinc (N=25)/ Wood dust (N=24)/ Manganese (N=22) | Iron,Zinc (N=25) |
|  | 439: Other specialised construction activities | 317 | 14.5% | Iron (N=20)/ Manganese (N=13)/ Quartz (N=12)/ Zinc (N=11)/ Lead (N=10) | Iron,Manganese (N=12) |
| 45: Wholesale and retail trade and repair of motor vehicles and motorcycles | 451: Sale of motor vehicles | 85 | 55.3% | Xylene (N=30)/ Ethylbenzene (N=26)/ Toluene (N=24)/ Butanone (N=21)/ Acetone (N=17) | Ethylbenzene,Xylene (N=26) |
|  | 452: Maintenance and repair of motor vehicles | 55 | 60% | Toluene (N=21)/ Xylene (N=19)/ Acetone (N=18)/ Butanone (N=18)/ Ethylbenzene (N=15) | Toluene,Xylene (N=19) |
|  | 453: Sale of motor vehicle parts and accessories | 20 | 60% | Aliphatic and alicyclic hydrocarbons (N=3)/ Aluminium (N=3)/ Iron (N=3)/ Magnesium (N=3)/ N-hexane (N=3) | Aluminium,Iron,Magnesium (N=3) |
|  | 454: Sale, maintenance and repair of motorcycles and related parts and accessories | 5 | 40% | Toluene (N=2)/ Xylene (N=2)/ Benzene (N=1)/ Butanone (N=1)/ C6-C12 hydrocarbons (N=1) | Toluene,Xylene (N=2) |
| 46: Wholesale trade, except of motor vehicles and motorcycles | 461: Wholesale on a fee or contract basis | 128 | 12.5% | Xylene (N=8)/ 1-Methoxypropan-2-ol (N=5)/ Ethylbenzene (N=5)/ 1,2,4-Trimethylbenzene (N=4)/ 2-Methoxy-1-methylethyl acetate (N=4) | Ethylbenzene,Xylene (N=5) |
|  | 462: Wholesale of agricultural raw materials and live animals | 17 | 41.2% | Methanol (N=4)/ Propan-2-ol (N=4)/ Ethylbenzene (N=2)/ Xylene (N=2)/ 1,2,4-Trimethylbenzene (N=1) | Methanol,Propan-2-ol (N=4) |
|  | 463: Wholesale of food, beverages and tobacco | 28 | 21.4% | Chloroform (N=2)/ Cyclohexane (N=2)/ Methanol (N=2)/ 1-Methoxypropan-2-ol (N=1)/ Acetone (N=1) | 1-Methoxypropan-2-ol,Acetone,Butanone,N-hexane,Tetrahydrofuran,Toluene,Xylene (N=1) |
|  | 464: Wholesale of household goods | 77 | 26% | Acetone (N=7)/ Butanone (N=4)/ Toluene (N=4)/ 4-Methylpentan-2-one (N=3)/ Chloroform (N=3) | 4-Methylpentan-2-one,Butanone (N=3) |
|  | 465: Wholesale of information and communication equipment | 3 | 33.3% | Tridymite (N=1)/ Wood dust (N=1) | Tridymite,Wood dust (N=1) |
|  | 466: Wholesale of other machinery, equipment and supplies | 121 | 43% | Iron (N=16)/ Xylene (N=16)/ Toluene (N=15)/ Acetone (N=13)/ Ethylbenzene (N=11) | Ethylbenzene,Xylene (N=11) |
|  | 467: Other specialised wholesale | 646 | 27.6% | Acetone (N=59)/ Xylene (N=53)/ Toluene (N=51)/ Butanone (N=39)/ Ethylbenzene (N=37) | Ethylbenzene,Xylene (N=35) |
|  | 469: Non-specialised wholesale trade | 15 | 20% | Xylene (N=3)/ Ethylbenzene (N=2)/ Propan-2-ol (N=2)/ (2-Methoxymethylethoxy)propanol (N=1)/ 1-Methoxypropan-2-ol (N=1) | Ethylbenzene,Propan-2-ol,Xylene (N=2) |
| 47: Retail trade, except of motor vehicles and motorcycles | 471: Retail sale in non-specialised stores | 30 | 13.3% | Toluene (N=2)/ Acetone (N=1)/ Aliphatic and alicyclic hydrocarbons (N=1)/ Benzene (N=1)/ Butanone (N=1) | Acetone,Butanone (N=1) |
|  | 473: Retail sale of automotive fuel in specialised stores | 12 | 58.3% | Benzene (N=6)/ Toluene (N=6)/ Ethylbenzene (N=5)/ Xylene (N=5)/ Heptane (N=4) | Benzene,Toluene (N=5) |
|  | 474: Retail sale of information and communication equipment in specialised stores | 1 | 0% |  |  |
|  | 475: Retail sale of other household equipment in specialised stores | 205 | 2.4% | Ethylbenzene (N=3)/ Xylene (N=3)/ Formaldehyde (N=2)/ N-butyl acetate (N=2)/ Pentane (N=2) | Ethylbenzene,Xylene (N=3) |
|  | 476: Retail sale of cultural and recreation goods in specialised stores | 18 | 50% | Acetone (N=5)/ Tetrahydrofuran (N=4)/ Chloroform (N=3)/ Aliphatic and alicyclic hydrocarbons (N=2)/ Butanone (N=2) | Acetone,Tetrahydrofuran (N=4) |
|  | 477: Retail sale of other goods in specialised stores | 21 | 9.5% | 1-Methoxypropan-2-ol (N=1)/ Acetone (N=1)/ Benzene (N=1)/ C6-C12 hydrocarbons (N=1)/ Propan-2-ol (N=1) | 1-Methoxypropan-2-ol,Acetone,C6-C12 hydrocarbons,Propan-2-ol (N=1) |
|  | 479: Retail trade not in stores, stalls or markets | 5 | 60% | Heptane (N=3)/ Tetrahydrofuran (N=3)/ N-hexane (N=2)/ Toluene (N=2)/ Hydrogen chloride (N=1) | Heptane,Tetrahydrofuran (N=3) |
| 49: Land transport and transport via pipelines | 491: Passenger rail transport, interurban | 175 | 43.4% | Quartz (N=31)/ Lead (N=29)/ Chromium (metal and inorganic Cr(II)/Cr(III) compounds) (N=23)/ Acetone (N=21)/ Cristobalite (N=19) | Chromium (metal and inorganic Cr(II)/Cr(III) compounds),Lead (N=20) |
|  | 492: Freight rail transport | 9 | 44.4% | Acetone (N=3)/ Cyclohexane (N=3)/ Heptane (N=3)/ N-hexane (N=3)/ Pentane (N=3) | Acetone,Cyclohexane,Heptane,N-hexane,Pentane (N=3) |
|  | 493: Other passenger land transport | 106 | 34.9% | Acetone (N=18)/ Xylene (N=12)/ Butanone (N=11)/ Quartz (N=11)/ Carbon dioxide (N= 9) | Acetone,Butanone (N=10) |
|  | 494: Freight transport by road and removal services | 49 | 38.8% | Toluene (N=9)/ Xylene (N=9)/ N-hexane (N=7)/ Ethylbenzene (N=6)/ Quartz (N=6) | N-hexane,Toluene (N=7) |
|  | 495: Transport via pipeline | 43 | 46.5% | Toluene (N=12)/ N-hexane (N= 9)/ Benzene (N= 8)/ Pentane (N= 7)/ Xylene (N= 7) | Benzene,N-hexane (N=7) |
| 50: Water transport | 501: Sea and coastal passenger water transport | 7 | 57.1% | Ethylbenzene (N=2)/ Xylene (N=2)/ (2-Methoxymethylethoxy)propanol (N=1)/ 2-Aminoethanol (N=1)/ Aliphatic and alicyclic hydrocarbons (N=1) | Ethylbenzene,Xylene (N=2) |
|  | 504: Inland freight water transport | 7 | 71.4% | Benzene (N=5)/ Chloroethylene (N=5) | Benzene,Chloroethylene (N=5) |
| 51: Air transport | 511: Passenger air transport | 13 | 61.5% | Toluene (N=6)/ Butanone (N=4)/ Xylene (N=4)/ Acetone (N=3)/ Cyclohexane (N=2) | Butanone,Toluene (N=4) |
| 52: Warehousing and support activities for transportation | 521: Warehousing and storage | 234 | 54.3% | Toluene (N=112)/ Benzene (N= 84)/ Xylene (N= 79)/ N-hexane (N= 63)/ Ethylbenzene (N= 48) | Benzene,Toluene (N=79) |
|  | 522: Support activities for transportation | 149 | 21.5% | Toluene (N=11)/ Xylene (N= 8)/ Ethylbenzene (N= 7)/ Pentane (N= 5)/ 2-Methylbutane (N= 4) | Ethylbenzene,Xylene (N=4) |
| 53: Postal and courier activities | 531: Postal activities under universal service obligation | 11 | 27.3% | Hexavalent chromium (N=2)/ Sulphuric acid (N=2)/ Acetone (N=1)/ Chloroform (N=1) | Hexavalent chromium,Sulphuric acid (N=2) |
| 55: Accommodation | 551: Hotels and similar accommodation | 2 | 50% | Acetone (N=1)/ Styrene (N=1) | Acetone,Styrene (N=1) |
|  | 553: Camping grounds, recreational vehicle parks and trailer parks | 1 | 0% |  |  |
|  | 559: Other accommodation | 8 | 0% |  |  |
| 56: Food and beverage service activities | 561: Restaurants and mobile food service activities | 14 | 42.9% | Lead (N=4)/ Mercury (N=4)/ Benz[a]anthracene (N=2)/ Benzo(e)acephenanthrylene (N=2)/ Benzo(k)fluoranthene (N=2) | Lead,Mercury (N=4) |
|  | 562: Event catering and other food service activities | 9 | 22.2% | Ethylbenzene (N=2)/ Toluene (N=2)/ Xylene (N=2)/ 1,2,4-Trimethylbenzene (N=1)/ 2-Methoxy-1-methylethyl acetate (N=1) | Ethylbenzene,Toluene,Xylene (N=2) |
| 58: Publishing activities | 581: Publishing of books, periodicals and other publishing activities | 22 | 54.5% | 1,2,4-Trimethylbenzene (N=4)/ Heptane (N=4)/ Mesitylene (N=4)/ 2-Butoxyethanol (N=3)/ Cumene (N=3) | 1,2,4-Trimethylbenzene,Mesitylene (N=3) |
| 59: Motion picture, video and television programme production, sound recording and music publishing activities | 591: Motion picture, video and television programme activities | 1 | 0% |  |  |
| 60: Programming and broadcasting activities | 602: Television programming and broadcasting activities | 3 | 0% |  |  |
| 61: Telecommunications | 611: Wired telecommunications activities | 6 | 0% |  |  |
| 62: Computer programming, consultancy and related activities | 620: Computer programming, consultancy and related activities | 13 | 23.1% | Lead (N=3)/ Quartz (N=3)/ Cristobalite (N=1)/ Hexavalent chromium (N=1) | Lead,Quartz (N=3) |
| 63: Information service activities | 639: Other information service activities | 7 | 57.1% | Acetone (N=4)/ Diethyl ether (N=2)/ Hydrogen chloride (N=2)/ Ammonia, anhydrous (N=1)/ Toluene (N=1) | Acetone,Diethyl ether (N=2) |
| 64: Financial service activities, except insurance and pension funding | 641: Monetary intermediation | 13 | 23.1% | 1-Methoxypropan-2-ol (N=2)/ Acetone (N=1)/ Butanone (N=1)/ Hexavalent chromium (N=1)/ Quartz (N=1) | 1-Methoxypropan-2-ol,Acetone,Butanone (N=1) |
|  | 642: Activities of holding companies | 160 | 31.9% | 1,2,4-Trimethylbenzene (N=26)/ 2-Butoxyethyl acetate (N=23)/ 1,2,3-Trimethylbenzene (N=18)/ Mesitylene (N=13)/ Xylene (N=11) | 1,2,4-Trimethylbenzene,2-Butoxyethyl acetate (N=23) |
|  | 643: Trusts, funds and similar financial entities | 6 | 83.3% | Acetonitrile (N=5)/ N-hexane (N=5)/ Tetrahydrofuran (N=4)/ Dichloromethane (N=3)/ Toluene (N=3) | Acetonitrile,N-hexane (N=5) |
| 65: Insurance, reinsurance and pension funding, except compulsory social security | 651: Insurance, reinsurance and pension funding, except compulsory social security | 3 | 33.3% | Aliphatic and alicyclic hydrocarbons (N=1)/ Monocyclic aromatic hydrocarbons (N=1) | Aliphatic and alicyclic hydrocarbons,Monocyclic aromatic hydrocarbons (N=1) |
| 66: Activities auxiliary to financial services and insurance activities | 661: Activities auxiliary to financial services, except insurance and pension funding | 6 | 0% |  |  |
| 68: Real estate activities | 681: Buying and selling of own real estate | 1 | 0% |  |  |
|  | 682: Renting and operating of own or leased real estate | 97 | 15.5% | Acetone (N=5)/ Butanone (N=4)/ Cristobalite (N=4)/ Quartz (N=4)/ Styrene (N=4) | Acetone,Styrene (N=4) |
|  | 683: Real estate activities on a fee or contract basis | 13 | 7.7% | 1-Methoxypropan-2-ol (N=1)/ Cyclohexane (N=1)/ Diethyl ether (N=1) | 1-Methoxypropan-2-ol,Cyclohexane,Diethyl ether (N=1) |
| 69: Legal and accounting activities | 692: Accounting, bookkeeping and auditing activities: tax consultancy | 2 | 0% |  |  |
| 70: Activities of head offices: management consultancy activities | 701: Activities of head offices | 174 | 20.1% | Toluene (N=15)/ Butanone (N= 8)/ Acetone (N= 7)/ Xylene (N= 7)/ Ethylbenzene (N= 6) | Butanone,Toluene (N=7) |
|  | 702: Management consultancy activities | 22 | 4.5% | Acetone (N=1)/ Chloroform (N=1) | Acetone,Chloroform (N=1) |
| 71: Architectural and engineering activities: technical testing and analysis | 711: Architectural and engineering activities and related technical consultancy | 211 | 39.8% | Toluene (N=22)/ Heptane (N=20)/ Xylene (N=20)/ Acetone (N=17)/ Quartz (N=17) | Ethylbenzene,Xylene (N=14) |
|  | 712: Technical testing and analysis | 196 | 32.7% | Toluene (N=35)/ Acetone (N=25)/ N-hexane (N=21)/ Heptane (N=20)/ Xylene (N=18) | Acetone,Toluene (N=18) |
| 72: Scientific research and development | 721: Research and experimental development on natural sciences and engineering | 297 | 26.9% | Acetone (N=34)/ Toluene (N=29)/ Dichloromethane (N=22)/ Acetonitrile (N=20)/ Methanol (N=16) | Acetone,Toluene (N=18) |
|  | 722: Research and experimental development on social sciences and humanities | 7 | 28.6% | Dichloromethane (N=1)/ Iron (N=1)/ Methanol (N=1)/ Nickel (N=1) | Dichloromethane,Methanol (N=1) |
| 73: Advertising and market research | 731: Advertising | 56 | 37.5% | 2-Butoxyethyl acetate (N=12)/ 1,2,4-Trimethylbenzene (N=10)/ Mesitylene (N= 7)/ 1,2,3-Trimethylbenzene (N= 6)/ Cristobalite (N= 4) | 1,2,4-Trimethylbenzene,2-Butoxyethyl acetate (N=9) |
| 74: Other professional, scientific and technical activities | 741: Specialised design activities | 22 | 22.7% | Acetone (N=2)/ Cyclohexane (N=2)/ Ethyl acetate (N=2)/ N-hexane (N=2)/ 2-Methoxy-1-methylethyl acetate (N=1) | Cyclohexane,Ethyl acetate,N-hexane (N=2) |
|  | 749: Other professional, scientific and technical activities n.e.c. | 64 | 31.2% | Formaldehyde (N=5)/ Xylene (N=5)/ Acetaldehyde (N=4)/ Acetone (N=4)/ Chloroform (N=4) | Acetaldehyde,Formaldehyde (N=4) |
| 75: Veterinary activities | 750: Veterinary activities | 12 | 66.7% | Diethyl ether (N=8)/ Ethanol (N=8)/ Isoflurane (N=8) | Diethyl ether,Ethanol,Isoflurane (N=8) |
| 77: Rental and leasing activities | 771: Renting and leasing of motor vehicles | 7 | 42.9% | N-hexane (N=2)/ Benzene (N=1)/ Butanone (N=1)/ Heptane (N=1)/ Toluene (N=1) | Benzene,N-hexane (N=1) |
|  | 772: Renting and leasing of personal and household goods | 18 | 44.4% | Ethylbenzene (N=3)/ Xylene (N=3)/ Aliphatic and alicyclic hydrocarbons (N=2)/ Copper (N=2)/ Iron (N=2) | Ethylbenzene,Xylene (N=3) |
|  | 773: Renting and leasing of other machinery, equipment and tangible goods | 89 | 30.3% | Xylene (N=9)/ Cyclohexane (N=7)/ Toluene (N=6)/ Acetone (N=5)/ Iron (N=5) | Cyclohexane,Xylene (N=6) |
|  | 774: Leasing of intellectual property and similar products, except copyrighted works | 3 | 66.7% | Acetone (N=2)/ Butanone (N=2)/ Tetrahydrofuran (N=1) | Acetone,Butanone (N=2) |
| 78: Employment activities | 781: Activities of employment placement agencies | 6 | 50% | Iron (N=2)/ Manganese (N=2)/ Chromium (metal and inorganic Cr(II)/Cr(III) compounds) (N=1)/ Hexavalent chromium (N=1)/ Lead (N=1) | Iron,Manganese (N=2) |
|  | 782: Temporary employment agency activities | 36 | 63.9% | Manganese (N=8)/ Metalworking fluids (inhalable fraction) (N=8)/ Water-soluble metalworking fluids (N=8)/ Chromium (metal and inorganic Cr(II)/Cr(III) compounds) (N=6)/ Copper (N=6) | Metalworking fluids (inhalable fraction),Water-soluble metalworking fluids (N=8) |
|  | 783: Other human resources provision | 4 | 50% | Acetone (N=1)/ Aliphatic and alicyclic hydrocarbons (N=1)/ Butanone (N=1)/ Cyclohexanone (N=1)/ Ethyl acetate (N=1) | Acetone,Aliphatic and alicyclic hydrocarbons,Ethyl acetate,N-hexane (N=1) |
| 79: Travel agency, tour operator and other reservation service and related activities | 799: Other reservation service and related activities | 3 | 0% |  |  |
| 80: Security and investigation activities | 801: Private security activities | 12 | 8.3% | Copper (N=1)/ Iron (N=1)/ Zinc (N=1) | Copper,Iron,Zinc (N=1) |
|  | 802: Security systems service activities | 3 | 0% |  |  |
| 81: Services to buildings and landscape activities | 811: Combined facilities support activities | 8 | 0% |  |  |
|  | 812: Cleaning activities | 83 | 43.4% | Quartz (N=12)/ Toluene (N=12)/ Xylene (N= 9)/ Aliphatic and alicyclic hydrocarbons (N= 8)/ Acetone (N= 6) | Toluene,Xylene (N=8) |
|  | 813: Landscape service activities | 10 | 10% | Cristobalite (N=1)/ Quartz (N=1) | Cristobalite,Quartz (N=1) |
| 82: Office administrative, office support and other business support activities | 821: Office administrative and support activities | 16 | 25% | Butanone (N=3)/ Toluene (N=3)/ Acetaldehyde (N=2)/ Formaldehyde (N=2)/ C6-C12 hydrocarbons (N=1) | Butanone,Toluene (N=3) |
|  | 823: Organisation of conventions and trade shows | 8 | 0% |  |  |
|  | 829: Business support service activities n.e.c. | 117 | 39.3% | Toluene (N=21)/ 2-Methoxy-1-methylethyl acetate (N=18)/ Xylene (N=16)/ 1-Methoxypropan-2-ol (N=15)/ Butanone (N=14) | 1-Methoxypropan-2-ol,2-Methoxy-1-methylethyl acetate (N=13) |
| 84: Public administration and defence: compulsory social security | 841: Administration of the State and the economic and social policy of the community | 374 | 21.7% | Xylene (N=19)/ Acetone (N=15)/ Ethylbenzene (N=15)/ Toluene (N=15)/ Butanone (N=12) | Ethylbenzene,Xylene (N=13) |
|  | 842: Provision of services to the community as a whole | 203 | 17.7% | Toluene (N=16)/ Xylene (N=16)/ Butanone (N=11)/ Acetone (N=10)/ Ethylbenzene (N=10) | Toluene,Xylene (N=11) |
|  | 843: Compulsory social security activities | 30 | 23.3% | Hydrogen fluoride (N=3)/ 1,2,4-Trimethylbenzene (N=2)/ Mesitylene (N=2)/ Nitric acid (N=2)/ Xylene (N=2) | 1,2,4-Trimethylbenzene,Mesitylene (N=2) |
| 85: Education | 851: Pre-primary education | 1 | 0% |  |  |
|  | 852: Primary education | 1 | 0% |  |  |
|  | 853: Secondary education | 263 | 7.2% | Iron (N=8)/ Copper (N=6)/ Zinc (N=5)/ Mesitylene (N=4)/ Quartz (N=4) | Copper,Iron (N=6) |
|  | 854: Higher education | 97 | 19.6% | Acetone (N=8)/ Ethanol (N=5)/ Quartz (N=5)/ Toluene (N=5)/ Dichloromethane (N=4) | Acetone,Ethanol (N=4) |
|  | 855: Other education | 95 | 7.4% | Acetone (N=4)/ Butanone (N=3)/ Styrene (N=3)/ Aliphatic and alicyclic hydrocarbons (N=2)/ Iron (N=2) | Acetone,Aliphatic and alicyclic hydrocarbons,Butanone,Toluene,Xylene (N=2) |
| 86: Human health activities | 861: Hospital activities | 234 | 31.2% | Ethanol (N=41)/ Formaldehyde (N=30)/ Sevoflurane (N=30)/ Xylene (N=28)/ Dinitrogen oxide (N=25) | Ethanol,Sevoflurane (N=24) |
|  | 862: Medical and dental practice activities | 50 | 46% | Formaldehyde (N=16)/ Xylene (N=13)/ Ethanol (N=10)/ Ethylbenzene (N= 9)/ Toluene (N= 8) | Formaldehyde,Xylene (N=12) |
|  | 869: Other human health activities | 16 | 68.8% | Methanol (N=9)/ Ethanol (N=7)/ Formaldehyde (N=7)/ Toluene (N=6)/ Xylene (N=5) | Ethanol,Formaldehyde (N=6) |
| 87: Residential care activities | 871: Residential nursing care activities | 86 | 5.8% | Dinitrogen oxide (N=2)/ 1-Methoxypropan-2-ol (N=1)/ 4-Hydroxy-4-methylpentan-2-one (N=1)/ Aliphatic and alicyclic hydrocarbons (N=1)/ Butan-1-ol (N=1) | 1-Methoxypropan-2-ol,4-Hydroxy-4-methylpentan-2-one,Aliphatic and alicyclic hydrocarbons,Butan-1-ol,Ethylbenzene,N-butyl acetate,Toluene,Xylene (N=1) |
|  | 872: Residential care activities for mental retardation, mental health and substance abuse | 62 | 8.1% | Quartz (N=4)/ Cristobalite (N=3)/ Tridymite (N=3)/ Ethanol (N=1)/ Formaldehyde (N=1) | Cristobalite,Quartz,Tridymite (N=3) |
|  | 873: Residential care activities for the elderly and disabled | 2 | 0% |  |  |
|  | 879: Other residential care activities | 10 | 0% |  |  |
| 88: Social work activities without accommodation | 881: Social work activities without accommodation for the elderly and disabled | 239 | 9.6% | Iron (N=13)/ Zinc (N= 9)/ Aluminium (N= 8)/ Manganese (N= 7)/ Copper (N= 6) | Iron,Zinc (N=9) |
|  | 889: Other social work activities without accommodation | 57 | 7% | Iron (N=3)/ Manganese (N=3)/ Copper (N=1)/ Lead (N=1)/ Mercury (N=1) | Iron,Manganese (N=3) |
| 90: Creative, arts and entertainment activities | 900: Creative, arts and entertainment activities | 52 | 3.8% | Acetone (N=2)/ Methyl methacrylate (N=2)/ Styrene (N=2) | Acetone,Methyl methacrylate,Styrene (N=2) |
| 91: Libraries, archives, museums and other cultural activities | 910: Libraries, archives, museums and other cultural activities | 66 | 9.1% | Acetone (N=4)/ Butanone (N=3)/ Wood dust (N=3)/ Hydrogen chloride (N=2)/ 2-Methoxy-1-methylethyl acetate (N=1) | Butanone,Wood dust (N=3) |
| 93: Sports activities and amusement and recreation activities | 931: Sports activities | 36 | 33.3% | Hexavalent chromium (N=11)/ Quartz (N=11)/ Aluminium (N= 1)/ Barium (N= 1)/ Copper (N= 1) | Hexavalent chromium,Quartz (N=11) |
|  | 932: Amusement and recreation activities | 14 | 21.4% | Acetone (N=2)/ Aliphatic and alicyclic hydrocarbons (N=2)/ Styrene (N=2)/ Pentane (N=1)/ Wood dust (N=1) | Acetone,Aliphatic and alicyclic hydrocarbons,Styrene (N=2) |
| 94: Activities of membership organisations | 941: Activities of business, employers and professional membership organisations | 65 | 12.3% | N-hexane (N=6)/ Toluene (N=4)/ Xylene (N=4)/ Acetone (N=3)/ Benzene (N=3) | N-hexane,Toluene (N=4) |
|  | 942: Activities of trade unions | 102 | 31.4% | 2-Methoxy-1-methylethyl acetate (N=12)/ 1-Methoxypropan-2-ol (N=10)/ Cristobalite (N=10)/ Quartz (N=10)/ Acetone (N= 5) | Cristobalite,Quartz (N=10) |
|  | 949: Activities of other membership organisations | 77 | 16.9% | Lead (N=7)/ Hexavalent chromium (N=3)/ Nickel (N=3)/ Zinc (N=3)/ Aluminium (N=2) | Lead,Nickel,Zinc (N=3) |
| 95: Repair of computers and personal and household goods | 952: Repair of personal and household goods | 8 | 75% | Ethyl acetate (N=3)/ 1-Methoxypropan-2-ol (N=2)/ Acetone (N=2)/ Aliphatic and alicyclic hydrocarbons (N=2)/ Cyclohexane (N=2) | 1-Methoxypropan-2-ol,Ethanol,Propan-2-ol (N=2) |
| 96: Other personal service activities | 960: Other personal service activities | 102 | 28.4% | Tetrachloroethylene (N=13)/ Acetone (N=10)/ Ethanol (N= 9)/ N-butyl acetate (N= 8)/ Aliphatic and alicyclic hydrocarbons (N= 7) | Acetone,Ethanol (N=7) |
| 99: Activities of extraterritorial organisations and bodies | 990: Activities of extraterritorial organisations and bodies | 2 | 100% | Acetonitrile (N=2)/ N-hexane (N=2)/ Methanol (N=1)/ N,N-Dimethylformamide (N=1) | Acetonitrile,N-hexane (N=2) |
| ZZ: Unclassified | ZZZ: Unclassified | 123 | 18.7% | Benzene (N=11)/ Refractory ceramic fibers (L>5um D<3um) (N= 8)/ Vinyl acetate (N= 7)/ Toluene (N= 6)/ Hydrogen chloride (N= 4) | Benzene,Toluene (N=4) |

## Table S5: List of tasks, carcinogens

List of tasks with exposure to at least one carcinogen, number of work situations, percentage of work situation with coexposure, most frequent agents among work situation with coexposure, most frequent mixture of agents across work situations

| Category | Task group | N WS | % Coex | Most frequent agents | Most frequent mixture |
| --- | --- | --- | --- | --- | --- |
| Machining, assembling, welding, bonding, chain assembly lines | Arc welding | 660 | 70% | Manganese (N=369)/ Iron (N=363)/ Copper (N=260)/ Zinc (N=238)/ Nickel (N=231) | Iron,Manganese (N=339) |
| Control, sterilization, cleaning, repair | Area cleaning | 224 | 24.1% | Quartz (N=25)/ Lead (N=20)/ Cristobalite (N=13)/ Hexavalent chromium (N= 8)/ Wood dust (N= 8) | Cristobalite,Quartz (N=13) |
|  | Assembling | 992 | 20.6% | Toluene (N=56)/ Butanone (N=54)/ Xylene (N=53)/ Acetone (N=48)/ Ethylbenzene (N=39) | Ethylbenzene,Xylene (N=38) |
| Bottling, filling, winding, dosing | Bagging (including packing in crates, big bags, etc.) | 347 | 24.2% | Quartz (N=47)/ Cristobalite (N=35)/ Butanone (N=13)/ Toluene (N=11)/ Acetone (N=10) | Cristobalite,Quartz (N=34) |
|  | Bottling | 83 | 48.2% | Butanone (N=16)/ Toluene (N=14)/ Acetone (N=13)/ Xylene (N= 9)/ Ethanol (N= 7) | Toluene,Xylene (N=7) |
|  | Bottling, filling, winding, dosing, warehousing, loading; other tasks n.e.c. | 6 | 16.7% | 1,2,4-Trimethylbenzene (N=1)/ Benzene (N=1)/ Ethylbenzene (N=1)/ Lead (N=1)/ Toluene (N=1) | 1,2,4-Trimethylbenzene,Benzene,Ethylbenzene,Lead,Toluene,Xylene (N=1) |
| Preparation, treatment, protection | Chemical treatment and protection of surfaces | 539 | 39.1% | Hexavalent chromium (N=59)/ Acetone (N=44)/ Hydrogen chloride (N=39)/ Hydrogen fluoride (N=36)/ Toluene (N=36) | Ethylbenzene,Xylene (N=22) |
|  | Cleaning of materials or finished parts | 610 | 33.3% | Acetone (N=82)/ Butanone (N=54)/ Xylene (N=54)/ Toluene (N=53)/ 1-Methoxypropan-2-ol (N=41) | Ethylbenzene,Xylene (N=31) |
|  | Cleaning of tools and machines | 719 | 26.4% | Toluene (N=45)/ Xylene (N=45)/ Acetone (N=39)/ Butanone (N=32)/ Quartz (N=31) | Toluene,Xylene (N=23) |
|  | Coating | 112 | 51.8% | Butanone (N=31)/ Toluene (N=31)/ Acetone (N=15)/ Xylene (N=12)/ Ethyl acetate (N= 9) | Butanone,Toluene (N=22) |
| Metallurgy and primary transformation of ferrous and non-ferrous metals | Coke oven plants and ancillary units | 59 | 40.7% | Benzene (N=21)/ Ammonia, anhydrous (N=11)/ Toluene (N=11)/ Benz[a]anthracene (N= 6)/ Benzo(e)acephenanthrylene (N= 6) | Ammonia, anhydrous,Benzene (N=11) |
| Collection and treatment of waste and water | Collection and treatment of waste | 568 | 41.7% | Lead (N=113)/ Quartz (N= 53)/ Toluene (N= 51)/ Ammonia, anhydrous (N= 48)/ Xylene (N= 44) | Toluene,Xylene (N=39) |
| Construction and public works | Demolition work | 102 | 37.3% | Lead (N=26)/ Iron (N=25)/ Zinc (N=19)/ Quartz (N=17)/ Nickel (N=15) | Iron,Zinc (N=19) |
| Textile | Dry cleaning | 67 | 29.9% | Tetrachloroethylene (N=14)/ Aliphatic and alicyclic hydrocarbons (N= 7)/ N-butyl acetate (N= 6)/ Ethanol (N= 4)/ Toluene (N= 4) | Aliphatic and alicyclic hydrocarbons,Tetrachloroethylene (N=7) |
| Operating and monitoring of melting, sintering, grilling, drying, casting facilities | Dryers | 87 | 17.2% | Quartz (N=8)/ Cristobalite (N=6)/ Toluene (N=4)/ Tridymite (N=4)/ Xylene (N=4) | Cristobalite,Quartz (N=6) |
|  | Dyeing | 15 | 26.7% | 1-Methoxypropan-2-ol (N=3)/ Ethylbenzene (N=2)/ Xylene (N=2)/ (2-Methoxymethylethoxy)propanol (N=1)/ 1,2,4-Trimethylbenzene (N=1) | Ethylbenzene,Xylene (N=2) |
| Printing, serigraphy, reproduction of documents | Electronic printing processes | 17 | 52.9% | 2-Butoxyethyl acetate (N=3)/ 2-Methoxy-1-methylethyl acetate (N=3)/ Cyclohexanone (N=3)/ Xylene (N=3)/ 1-Methoxypropan-2-ol (N=2) | 2-Butoxyethyl acetate,Cyclohexanone (N=3) |
|  | Extraction or drilling work | 332 | 39.2% | Quartz (N=118)/ Cristobalite (N= 91)/ Tridymite (N= 24)/ Hexavalent chromium (N= 13)/ Diesel exhaust, elemental carbon (N= 7) | Cristobalite,Quartz (N=90) |
|  | Fabrication of electrodes for electrometallurgy | 21 | 14.3% | Barium (N=2)/ Chlorine (N=2)/ Cristobalite (N=1)/ Hydrogen chloride (N=1)/ Lead (N=1) | Barium,Chlorine (N=2) |
|  | Filling | 94 | 47.9% | Xylene (N=20)/ Toluene (N=18)/ Acetone (N=14)/ Ethylbenzene (N=12)/ Butanone (N=11) | Toluene,Xylene (N=13) |
|  | Finishing and maintenance of buildings | 462 | 16.2% | Quartz (N=23)/ Lead (N=19)/ Xylene (N=18)/ Cristobalite (N=12)/ Ethylbenzene (N=12) | Cristobalite,Quartz (N=12) |
| Foundry | Finishing of foundry molds and cores | 337 | 24% | Quartz (N=29)/ Cristobalite (N=17)/ Butanone (N=15)/ Xylene (N=15)/ Toluene (N=13) | Cristobalite,Quartz (N=17) |
|  | Flexography | 51 | 60.8% | Ethanol (N=22)/ Ethyl acetate (N=22)/ 1-Methoxypropan-2-ol (N=13)/ Propan-2-ol (N=12)/ Butanone (N=11) | Ethanol,Ethyl acetate (N=21) |
| Agriculture and agri-food industries | Food industries | 91 | 44% | Formaldehyde (N=11)/ Acetaldehyde (N=10)/ N-hexane (N= 9)/ Benz[a]anthracene (N= 5)/ Diethyl ether (N= 5) | Acetaldehyde,Formaldehyde (N=9) |
|  | Foundries, other tasks n.e.c. | 120 | 30% | Lead (N=21)/ Quartz (N=15)/ Hexavalent chromium (N=10)/ Iron (N= 8)/ Copper (N= 7) | Lead,Quartz (N=9) |
| Storage and transport operations of raw materials / intermediate products / finished products | Fuel distribution | 41 | 48.8% | Toluene (N=19)/ Benzene (N=12)/ Xylene (N=10)/ N-hexane (N= 9)/ Ethylbenzene (N= 7) | Benzene,Toluene (N=12) |
|  | Gas welding or brazing (torch) | 146 | 37.7% | Iron (N=27)/ Copper (N=22)/ Zinc (N=20)/ Lead (N=17)/ Manganese (N=16) | Iron,Manganese (N=16) |
|  | Gluing | 406 | 46.3% | Butanone (N=104)/ Acetone (N= 88)/ Toluene (N= 75)/ Aliphatic and alicyclic hydrocarbons (N= 40)/ Ethyl acetate (N= 39) | Acetone,Butanone (N=57) |
| Special cases | Hair salons / nails salons | 7 | 85.7% | Acetone (N=6)/ Ethanol (N=6)/ Ethyl acetate (N=4)/ N-butyl acetate (N=4)/ Propan-2-ol (N=4) | Acetone,Ethanol (N=6) |
|  | Hopper silos loading / unloading operations | 1000 | 24.5% | Quartz (N=109)/ Cristobalite (N= 90)/ Xylene (N= 53)/ Toluene (N= 41)/ Lead (N= 38) | Cristobalite,Quartz (N=88) |
| Hospitals, medical and diagnostic laboratories | Hospitals, medical and diagnostic laboratories | 336 | 48.2% | Ethanol (N=76)/ Formaldehyde (N=60)/ Xylene (N=53)/ Toluene (N=52)/ Sevoflurane (N=37) | Ethanol,Formaldehyde (N=38) |
|  | Impregnation of surfaces or core (wood, bricks) | 66 | 19.7% | Xylene (N=5)/ Butanone (N=4)/ Ethylbenzene (N=4)/ Phenol (N=4)/ Acetone (N=3) | Ethylbenzene,Xylene (N=4) |
|  | Inspecting, cleaning, repairing; other tasks n.e.c. | 911 | 25.5% | Toluene (N=63)/ Quartz (N=58)/ Acetone (N=47)/ Xylene (N=47)/ Benzene (N=33) | Cristobalite,Quartz (N=31) |
|  | Knitting and sewing | 20 | 10% | Acetone (N=1)/ Aliphatic and alicyclic hydrocarbons (N=1)/ Butanone (N=1)/ Ethyl acetate (N=1)/ Iron (N=1) | Acetone,Aliphatic and alicyclic hydrocarbons,Butanone,Ethyl acetate,Propyl acetate,Toluene (N=1) |
|  | Machining | 3415 | 17.1% | Iron (N=185)/ Manganese (N=136)/ Metalworking fluids (inhalable fraction) (N=120)/ Water-soluble metalworking fluids (N=108)/ Quartz (N=107) | Iron,Manganese (N=126) |
|  | Machining, assembling, welding, gluing, chain assembling: other tasks n.e.c. | 2068 | 20% | Acetone (N=92)/ Butanone (N=92)/ Quartz (N=75)/ Chromium (metal and inorganic Cr(II)/Cr(III) compounds) (N=71)/ Xylene (N=65) | Chromium (metal and inorganic Cr(II)/Cr(III) compounds),Hexavalent chromium (N=42) |
|  | Malting plants, breweries | 1 | 0% |  |  |
| Mixing, molding, compression, reaction, rolling, calendering | Manufacture of composite material parts | 386 | 63.7% | Styrene (N=188)/ Acetone (N=182)/ Methyl methacrylate (N= 69)/ Butanone (N= 59)/ Aliphatic and alicyclic hydrocarbons (N= 43) | Acetone,Styrene (N=175) |
|  | Manufacture of cores | 67 | 40.3% | Quartz (N=21)/ Phenol (N=12)/ Cristobalite (N=10)/ Formaldehyde (N= 9)/ Aluminium (N= 3) | Phenol,Quartz (N=12) |
|  | Manufacture of foundry molds | 87 | 46% | Quartz (N=29)/ Cristobalite (N=16)/ Phenol (N=11)/ Formaldehyde (N= 9)/ Tridymite (N= 6) | Cristobalite,Quartz (N=16) |
|  | Manufacturing control, sterilization (except medical field) | 1156 | 31.7% | Quartz (N=88)/ Xylene (N=79)/ Acetone (N=76)/ Toluene (N=75)/ Cristobalite (N=56) | Cristobalite,Quartz (N=53) |
|  | Manufacturing of artificial mineral fibers | 60 | 23.3% | Ammonia, anhydrous (N=12)/ Phenol (N= 9)/ Quartz (N= 5)/ Cristobalite (N= 3)/ Refractory ceramic fibers (L>5um D<3um) (N= 3) | Ammonia, anhydrous,Phenol (N=9) |
|  | Manufacturing of porcelain, ceramic and pottery products | 82 | 35.4% | Quartz (N=23)/ Cristobalite (N=16)/ Lead (N= 9)/ Cadmium (N= 3)/ Cobalt (N= 2) | Cristobalite,Quartz (N=16) |
|  | Mechanical surface treatment | 1006 | 26.8% | Iron (N=92)/ Quartz (N=70)/ Manganese (N=67)/ Nickel (N=58)/ Zinc (N=54) | Iron,Manganese (N=58) |
|  | Melting, sintering, grilling, drying, casting; other tasks n.e.c. | 9 | 11.1% | Cristobalite (N=1)/ Quartz (N=1) | Cristobalite,Quartz (N=1) |
|  | Metal making and casting | 556 | 33.6% | Quartz (N=104)/ Lead (N= 71)/ Cristobalite (N= 54)/ Refractory ceramic fibers (L>5um D<3um) (N= 40)/ Hexavalent chromium (N= 39) | Cristobalite,Quartz (N=53) |
|  | Metallurgy and primary transformation of ferrous and non-ferrous metals, other tasks n.e.c. | 167 | 38.9% | Lead (N=41)/ Quartz (N=24)/ Chromium (metal and inorganic Cr(II)/Cr(III) compounds) (N=19)/ Hexavalent chromium (N=18)/ Cristobalite (N=15) | Chromium (metal and inorganic Cr(II)/Cr(III) compounds),Lead (N=18) |
| Operating and monitoring of milling and sorting facilities | Milling and sorting; other tasks n.e.c. | 18 | 16.7% | Cristobalite (N=2)/ Quartz (N=2)/ Hydrogen chloride (N=1)/ Lead (N=1) | Cristobalite,Quartz (N=2) |
|  | Miscellaneous | 7 | 42.9% | Benz[a]anthracene (N=1)/ Benzo(e)acephenanthrylene (N=1)/ Benzo(k)fluoranthene (N=1)/ Benzo[def]chrysene (N=1)/ Benzo[ghi]perylene (N=1) | Benz[a]anthracene,Benzo(e)acephenanthrylene,Benzo(k)fluoranthene,Benzo[def]chrysene,Benzo[ghi]perylene (N=1) |
|  | Miscellaneous printing tasks | 25 | 48% | 1,2,4-Trimethylbenzene (N=7)/ Mesitylene (N=6)/ 2-Butoxyethyl acetate (N=5)/ Butanone (N=4)/ 1,2,3-Trimethylbenzene (N=3) | 1,2,4-Trimethylbenzene,Mesitylene (N=6) |
|  | Mixing | 439 | 45.1% | Quartz (N=75)/ Butanone (N=51)/ Xylene (N=50)/ Toluene (N=43)/ Ethylbenzene (N=42) | Ethylbenzene,Xylene (N=41) |
|  | Mixing, compression, molding, reaction; other tasks n.e.c. | 10 | 20% | Acetone (N=1)/ Butyl acrylate (N=1)/ Cadmium (N=1)/ Methyl methacrylate (N=1)/ Zinc (N=1) | Acetone,Butyl acrylate,Methyl methacrylate (N=1) |
|  | Molding, pelletizing, calendering, rolling facilities | 291 | 36.4% | Quartz (N=40)/ Cristobalite (N=25)/ Tridymite (N=18)/ Iron (N=14)/ Styrene (N=14) | Cristobalite,Quartz (N=24) |
|  | Offset printing | 83 | 65.1% | Propan-2-ol (N=22)/ Aliphatic and alicyclic hydrocarbons (N=17)/ Toluene (N=13)/ 1,2,4-Trimethylbenzene (N=11)/ Butanone (N=11) | Aliphatic and alicyclic hydrocarbons,Propan-2-ol (N=12) |
|  | Operating and monitoring of milling and sorting facilities | 946 | 24.9% | Quartz (N=110)/ Cristobalite (N= 78)/ Lead (N= 69)/ Toluene (N= 46)/ Xylene (N= 44) | Cristobalite,Quartz (N=76) |
|  | Operating handling trolleys, road machinery and other vehicles | 942 | 26.5% | Quartz (N=143)/ Cristobalite (N=100)/ Lead (N= 60)/ Wood dust (N= 36)/ Hexavalent chromium (N= 34) | Cristobalite,Quartz (N=97) |
|  | Other construction tasks, n.e.c. | 239 | 24.3% | Quartz (N=41)/ Cristobalite (N=25)/ Lead (N=11)/ Toluene (N=11)/ Hexavalent chromium (N=10) | Cristobalite,Quartz (N=25) |
| Leather industry | Other leather industry tasks n.e.c. | 21 | 33.3% | Acetone (N=4)/ Cyclohexane (N=3)/ Butanone (N=2)/ Ethyl acetate (N=2)/ N-hexane (N=2) | Acetone,Butanone,Cyclohexane,N-hexane (N=2) |
|  | Other particular work n.e.c. | 318 | 13.8% | Quartz (N=11)/ Xylene (N=11)/ Tridymite (N=10)/ Toluene (N= 9)/ Acetone (N= 6) | Quartz,Tridymite (N=8) |
|  | Other printing processes | 29 | 41.4% | Ethylbenzene (N=5)/ Xylene (N=5)/ 1-Methoxypropan-2-ol (N=4)/ 1,2,4-Trimethylbenzene (N=4)/ Acetone (N=4) | Ethylbenzene,Xylene (N=4) |
|  | Other welding processes | 207 | 28% | Iron (N=28)/ Zinc (N=18)/ Copper (N=17)/ Lead (N=16)/ Hexavalent chromium (N=13) | Iron,Zinc (N=17) |
|  | Ovens (excluding foundries) | 277 | 28.5% | Quartz (N=38)/ Cristobalite (N=26)/ Refractory ceramic fibers (L>5um D<3um) (N=25)/ Lead (N=15)/ Hexavalent chromium (N=14) | Cristobalite,Quartz (N=24) |
|  | Packaging, palletization | 322 | 11.5% | Quartz (N=11)/ Cristobalite (N=10)/ Acetone (N= 6)/ Styrene (N= 6)/ Ethylbenzene (N= 5) | Cristobalite,Quartz (N=10) |
|  | Papermaking | 32 | 34.4% | Ammonia, anhydrous (N=5)/ Wood dust (N=5)/ 2-Butoxyethyl acetate (N=4)/ 1,2,4-Trimethylbenzene (N=3)/ Acetaldehyde (N=2) | Ammonia, anhydrous,Wood dust (N=5) |
|  | Photogravure | 239 | 33.9% | Ethyl acetate (N=30)/ Ethanol (N=28)/ Butanone (N=24)/ Toluene (N=23)/ Xylene (N=19) | Ethanol,Ethyl acetate (N=28) |
|  | Press control and surveillance, extruder, injection molding machines | 413 | 32.7% | Formaldehyde (N=45)/ Xylene (N=28)/ Toluene (N=27)/ Acetaldehyde (N=23)/ Ethylbenzene (N=23) | Acetaldehyde,Formaldehyde (N=23) |
|  | Printing: other tasks n.e.c. | 78 | 56.4% | Ethanol (N=17)/ Propan-2-ol (N=16)/ Butanone (N=15)/ Ethyl acetate (N=13)/ 2-Methoxy-1-methylethyl acetate (N=11) | Ethanol,Propan-2-ol (N=13) |
|  | Protection and treatment of surfaces by application of paints, varnishes, powders, release agents | 1609 | 57.4% | Xylene (N=587)/ Ethylbenzene (N=480)/ Toluene (N=392)/ Butanone (N=311)/ Acetone (N=304) | Ethylbenzene,Xylene (N=466) |
|  | Public safety, protection and emergency services | 1 | 0% |  |  |
|  | Reaction (polymerization units, distillation, extraction, desulfurization, extraction plants, etc.) | 495 | 48.9% | Toluene (N=121)/ Xylene (N= 75)/ Benzene (N= 73)/ N-hexane (N= 68)/ Ammonia, anhydrous (N= 58) | Toluene,Xylene (N=58) |
|  | Repair, maintenance, inspection | 2251 | 36.8% | Quartz (N=256)/ Toluene (N=214)/ Xylene (N=199)/ Benzene (N=167)/ Lead (N=140) | Cristobalite,Quartz (N=133) |
|  | Roadwork - sealing of structures and basins | 112 | 25.9% | Quartz (N=14)/ Cristobalite (N=10)/ Bitumen fumes (N= 9)/ Ethylbenzene (N= 5)/ Pyrene (N= 5) | Cristobalite,Quartz (N=9) |
|  | Sand regeneration facilities | 45 | 31.1% | Quartz (N=12)/ Cristobalite (N= 9)/ Lead (N= 2)/ Phenol (N= 2)/ 1-Methoxypropan-2-ol (N= 1) | Cristobalite,Quartz (N=9) |
|  | Screen printing | 150 | 64.7% | Acetone (N=40)/ Toluene (N=39)/ 2-Methoxy-1-methylethyl acetate (N=34)/ Cyclohexanone (N=34)/ Xylene (N=30) | 2-Methoxy-1-methylethyl acetate,Cyclohexanone (N=21) |
|  | Shared services, areas without specific pollution | 320 | 11.6% | Quartz (N=23)/ Cristobalite (N=20)/ Toluene (N= 6)/ Xylene (N= 6)/ Ethylbenzene (N= 5) | Cristobalite,Quartz (N=20) |
|  | Shared services, general areas with specific pollution n.e.c. | 1280 | 45.1% | Acetone (N=245)/ Toluene (N=200)/ N-hexane (N=143)/ Chloroform (N=110)/ Xylene (N=103) | Acetone,Toluene (N=108) |
|  | Shooting ranges, pyrotechnics | 101 | 9.9% | Lead (N=9)/ Quartz (N=6)/ Acetone (N=2)/ Heptane (N=2)/ Iron (N=2) | Lead,Quartz (N=6) |
|  | Social work | 1 | 0% |  |  |
|  | Spinning mills | 348 | 33.3% | Xylene (N=42)/ Quartz (N=39)/ Ethylbenzene (N=30)/ Cristobalite (N=25)/ Toluene (N=22) | Ethylbenzene,Xylene (N=28) |
|  | Storage and transport: other tasks n.e.c. | 16 | 6.2% | Pentane (N=1)/ Toluene (N=1) | Pentane,Toluene (N=1) |
|  | Structural work | 374 | 19.5% | Quartz (N=49)/ Cristobalite (N=25)/ Refractory ceramic fibers (L>5um D<3um) (N=16)/ Lead (N=11)/ Hexavalent chromium (N=10) | Cristobalite,Quartz (N=23) |
|  | Surface preparation, treatment, protection; other tasks n.e.c. | 14 | 35.7% | Lead (N=2)/ Acetonitrile (N=1)/ Butanone (N=1)/ Ethylbenzene (N=1)/ Hydrogen chloride (N=1) | Acetonitrile,Methanol (N=1) |
|  | Tanning and tawing | 54 | 48.1% | 1-Methoxypropan-2-ol (N=11)/ Xylene (N=10)/ Butanone (N= 9)/ Acetone (N= 6)/ Ethylbenzene (N= 6) | Butanone,Xylene (N=5) |
|  | Textile industry: other tasks n.e.c. | 48 | 52.1% | Toluene (N=12)/ Ammonia, anhydrous (N= 9)/ Butanone (N= 7)/ Aliphatic and alicyclic hydrocarbons (N= 6)/ Acetone (N= 4) | Ammonia, anhydrous,Toluene (N=9) |
|  | Thermal or thermo-chemical surface treatment | 153 | 37.9% | Lead (N=15)/ Xylene (N=15)/ Hydrogen fluoride (N=14)/ Ethylbenzene (N=12)/ Hydrogen chloride (N=11) | Ethylbenzene,Xylene (N=12) |
|  | Thermal stations, swimming pools | 7 | 0% |  |  |
|  | Thermometrics | 2 | 0% |  |  |
|  | Toll booth of car parks, highways, fuel distribution | 33 | 21.2% | Toluene (N=7)/ 1,2,4-Trimethylbenzene (N=3)/ Benzene (N=3)/ Ethylbenzene (N=3)/ Xylene (N=3) | 1,2,4-Trimethylbenzene,Toluene (N=3) |
|  | Transport of raw materials / intermediate products / finished products | 1063 | 36.6% | Toluene (N=161)/ Quartz (N=105)/ Xylene (N= 96)/ Benzene (N= 90)/ Cristobalite (N= 70) | Toluene,Xylene (N=85) |
|  | Treatment and electrochemical protection of surfaces | 81 | 39.5% | Hexavalent chromium (N=18)/ Nickel (N=15)/ Total chromium (N=10)/ Copper (N= 9)/ Iron (N= 8) | Hexavalent chromium,Nickel (N=11) |
|  | Viticulture | 6 | 33.3% | Cristobalite (N=2)/ Quartz (N=2) | Cristobalite,Quartz (N=2) |
|  | Water collection and treatment | 126 | 41.3% | Toluene (N=26)/ Hydrogen sulphide (N=19)/ Benzene (N=18)/ Xylene (N=18)/ Ammonia, anhydrous (N=16) | Toluene,Xylene (N=16) |
|  | Weaving | 9 | 0% |  |  |
|  | Weighing, dosing | 226 | 34.5% | Quartz (N=20)/ Toluene (N=17)/ Xylene (N=16)/ Ethylbenzene (N=12)/ 2-Methoxy-1-methylethyl acetate (N=11) | Cristobalite,Quartz (N=11) |
|  | Winding | 101 | 31.7% | Butanone (N=6)/ Lead (N=5)/ Quartz (N=5)/ Xylene (N=5)/ Acetone (N=4) | Cristobalite,Quartz (N=4) |

## Table S6: Most frequent itemsets, all agents

List of the 100 most frequent itemsets identified by level of support among WS exposed to a minimum of 2 agents. Minimum level of support = 0.1%, minimum of 2 items per itemset.

| Itemset | N items | N WS | Support |
| --- | --- | --- | --- |
| {Ethylbenzene,Xylene} | 2 | 1550 | 14.7% |
| {Cristobalite,Quartz} | 2 | 1417 | 13.4% |
| {Toluene,Xylene} | 2 | 1305 | 12.4% |
| {Ethylbenzene,Toluene} | 2 | 995 | 9.4% |
| {Ethylbenzene,Toluene,Xylene} | 3 | 945 | 9.0% |
| {Acetone,Toluene} | 2 | 768 | 7.3% |
| {Iron,Manganese} | 2 | 767 | 7.3% |
| {Butanone,Toluene} | 2 | 679 | 6.4% |
| {Acetone,Xylene} | 2 | 675 | 6.4% |
| {Acetone,Butanone} | 2 | 664 | 6.3% |
| {Iron,Zinc} | 2 | 656 | 6.2% |
| {Butanone,Xylene} | 2 | 642 | 6.1% |
| {Benzene,Toluene} | 2 | 569 | 5.4% |
| {Copper,Iron} | 2 | 564 | 5.3% |
| {N-hexane,Toluene} | 2 | 552 | 5.2% |
| {Iron,Nickel} | 2 | 508 | 4.8% |
| {Copper,Manganese} | 2 | 502 | 4.8% |
| {Acetone,Ethylbenzene} | 2 | 500 | 4.7% |
| {1,2,4-Trimethylbenzene,Xylene} | 2 | 492 | 4.7% |
| {Butanone,Ethylbenzene} | 2 | 475 | 4.5% |
| {Acetone,Ethylbenzene,Xylene} | 3 | 475 | 4.5% |
| {Manganese,Zinc} | 2 | 473 | 4.5% |
| {Copper,Iron,Manganese} | 3 | 463 | 4.4% |
| {Iron,Manganese,Zinc} | 3 | 462 | 4.4% |
| {Manganese,Nickel} | 2 | 456 | 4.3% |
| {Acetone,Toluene,Xylene} | 3 | 455 | 4.3% |
| {Butanone,Ethylbenzene,Xylene} | 3 | 454 | 4.3% |
| {1,2,4-Trimethylbenzene,Ethylbenzene} | 2 | 431 | 4.1% |
| {Copper,Zinc} | 2 | 431 | 4.1% |
| {Iron,Total chromium} | 2 | 424 | 4.0% |
| {Aluminium,Iron} | 2 | 419 | 4.0% |
| {1,2,4-Trimethylbenzene,Ethylbenzene,Xylene} | 3 | 414 | 3.9% |
| {Iron,Manganese,Nickel} | 3 | 411 | 3.9% |
| {Chromium (metal and inorganic Cr(II)/Cr(III) compounds),Hexavalent chromium} | 2 | 408 | 3.9% |
| {Copper,Iron,Zinc} | 3 | 408 | 3.9% |
| {Benzene,Xylene} | 2 | 406 | 3.8% |
| {Butanone,Toluene,Xylene} | 3 | 406 | 3.8% |
| {2-Methoxy-1-methylethyl acetate,Xylene} | 2 | 394 | 3.7% |
| {Copper,Nickel} | 2 | 389 | 3.7% |
| {4-Methylpentan-2-one,Xylene} | 2 | 388 | 3.7% |
| {N-hexane,Xylene} | 2 | 388 | 3.7% |
| {Acetone,Styrene} | 2 | 381 | 3.6% |
| {Acetone,Ethylbenzene,Toluene} | 3 | 375 | 3.6% |
| {Benzene,Toluene,Xylene} | 3 | 374 | 3.5% |
| {Hexavalent chromium,Lead} | 2 | 368 | 3.5% |
| {1,2,4-Trimethylbenzene,Toluene} | 2 | 367 | 3.5% |
| {Nickel,Total chromium} | 2 | 365 | 3.5% |
| {Manganese,Total chromium} | 2 | 363 | 3.4% |
| {Acetone,Ethylbenzene,Toluene,Xylene} | 4 | 360 | 3.4% |
| {Iron,Manganese,Total chromium} | 3 | 351 | 3.3% |
| {Copper,Iron,Nickel} | 3 | 348 | 3.3% |
| {Copper,Manganese,Zinc} | 3 | 344 | 3.3% |
| {Quartz,Tridymite} | 2 | 338 | 3.2% |
| {Iron,Nickel,Total chromium} | 3 | 338 | 3.2% |
| {Copper,Iron,Manganese,Zinc} | 4 | 337 | 3.2% |
| {Lead,Quartz} | 2 | 337 | 3.2% |
| {Copper,Manganese,Nickel} | 3 | 336 | 3.2% |
| {Butanone,Ethylbenzene,Toluene} | 3 | 332 | 3.1% |
| {4-Methylpentan-2-one,Toluene} | 2 | 330 | 3.1% |
| {1-Methoxypropan-2-ol,Xylene} | 2 | 330 | 3.1% |
| {N-hexane,Toluene,Xylene} | 3 | 330 | 3.1% |
| {1,2,4-Trimethylbenzene,Toluene,Xylene} | 3 | 329 | 3.1% |
| {Acetone,Butanone,Xylene} | 3 | 328 | 3.1% |
| {2-Methoxy-1-methylethyl acetate,Ethylbenzene} | 2 | 327 | 3.1% |
| {Nickel,Zinc} | 2 | 327 | 3.1% |
| {4-Methylpentan-2-one,Ethylbenzene} | 2 | 325 | 3.1% |
| {1-Methoxypropan-2-ol,2-Methoxy-1-methylethyl acetate} | 2 | 324 | 3.1% |
| {Acetone,Butanone,Toluene} | 3 | 324 | 3.1% |
| {Acetone,N-hexane} | 2 | 323 | 3.1% |
| {Heptane,Toluene} | 2 | 321 | 3.0% |
| {Butanone,Ethylbenzene,Toluene,Xylene} | 4 | 321 | 3.0% |
| {4-Methylpentan-2-one,Ethylbenzene,Xylene} | 3 | 316 | 3.0% |
| {Iron,Nickel,Zinc} | 3 | 316 | 3.0% |
| {Benzene,Ethylbenzene} | 2 | 314 | 3.0% |
| {Aluminium,Manganese} | 2 | 313 | 3.0% |
| {Aluminium,Zinc} | 2 | 312 | 3.0% |
| {Manganese,Nickel,Total chromium} | 3 | 311 | 2.9% |
| {2-Methoxy-1-methylethyl acetate,Ethylbenzene,Xylene} | 3 | 311 | 2.9% |
| {Copper,Iron,Manganese,Nickel} | 4 | 308 | 2.9% |
| {2-Methoxy-1-methylethyl acetate,Butanone} | 2 | 307 | 2.9% |
| {Aluminium,Iron,Zinc} | 3 | 302 | 2.9% |
| {Iron,Manganese,Nickel,Total chromium} | 4 | 302 | 2.9% |
| {4-Methylpentan-2-one,Butanone} | 2 | 302 | 2.9% |
| {Benzene,N-hexane} | 2 | 302 | 2.9% |
| {Aluminium,Iron,Manganese} | 3 | 300 | 2.8% |
| {2-Methoxy-1-methylethyl acetate,Toluene} | 2 | 299 | 2.8% |
| {Benzene,Ethylbenzene,Xylene} | 3 | 298 | 2.8% |
| {Copper,Total chromium} | 2 | 297 | 2.8% |
| {1,2,4-Trimethylbenzene,Ethylbenzene,Toluene} | 3 | 295 | 2.8% |
| {Ethylbenzene,N-hexane} | 2 | 292 | 2.8% |
| {Cyclohexane,Toluene} | 2 | 291 | 2.8% |
| {Benzene,Ethylbenzene,Toluene} | 3 | 290 | 2.7% |
| {1,2,4-Trimethylbenzene,Ethylbenzene,Toluene,Xylene} | 4 | 289 | 2.7% |
| {Copper,Iron,Total chromium} | 3 | 287 | 2.7% |
| {Total chromium,Zinc} | 2 | 287 | 2.7% |
| {Iron,Total chromium,Zinc} | 3 | 285 | 2.7% |
| {Acetone,Heptane} | 2 | 285 | 2.7% |
| {Chromium (metal and inorganic Cr(II)/Cr(III) compounds),Lead} | 2 | 284 | 2.7% |
| {1-Methoxypropan-2-ol,Butanone} | 2 | 284 | 2.7% |
| {Aluminium,Copper} | 2 | 283 | 2.7% |

## Table S7: Most frequent itemsets, carcinogens

List of the 100 most frequent itemsets of carcinogens (IARC groups 1, 2A, and 2B) identified by level of support among WS exposed to a minimum of 2 carcinogens. Minimum level of support = 0.1%, minimum of 2 items per itemset.

| Itemset | N items | N WS | Support |
| --- | --- | --- | --- |
| {Cristobalite,Quartz} | 2 | 1417 | 32.2% |
| {Hexavalent chromium,Lead} | 2 | 368 | 8.4% |
| {Lead,Quartz} | 2 | 337 | 7.7% |
| {4-Methylpentan-2-one,Ethylbenzene} | 2 | 325 | 7.4% |
| {Benzene,Ethylbenzene} | 2 | 314 | 7.1% |
| {Hexavalent chromium,Quartz} | 2 | 245 | 5.6% |
| {Hexavalent chromium,Nickel} | 2 | 198 | 4.5% |
| {Lead,Nickel} | 2 | 166 | 3.8% |
| {Quartz,Refractory ceramic fibers (L>5um D<3um)} | 2 | 139 | 3.2% |
| {Acetaldehyde,Formaldehyde} | 2 | 131 | 3.0% |
| {Lead,Wood dust} | 2 | 117 | 2.7% |
| {Cobalt,Nickel} | 2 | 113 | 2.6% |
| {Ethylbenzene,Styrene} | 2 | 109 | 2.5% |
| {Quartz,Wood dust} | 2 | 102 | 2.3% |
| {Dichloromethane,Ethylbenzene} | 2 | 98 | 2.2% |
| {Ethylbenzene,Lead} | 2 | 97 | 2.2% |
| {Cumene,Ethylbenzene} | 2 | 95 | 2.2% |
| {Hexavalent chromium,Lead,Quartz} | 3 | 92 | 2.1% |
| {Cristobalite,Lead} | 2 | 89 | 2.0% |
| {Cristobalite,Lead,Quartz} | 3 | 87 | 2.0% |
| {Ethanol,Ethylbenzene} | 2 | 86 | 2.0% |
| {Lead,Refractory ceramic fibers (L>5um D<3um)} | 2 | 84 | 1.9% |
| {Ethylbenzene,Quartz} | 2 | 80 | 1.8% |
| {Nickel,Welding fumes} | 2 | 79 | 1.8% |
| {Benzene,Chloroethylene} | 2 | 77 | 1.7% |
| {Benzene,Lead} | 2 | 70 | 1.6% |
| {Benzo(k)fluoranthene,Benzo[def]chrysene} | 2 | 69 | 1.6% |
| {Benzo(e)acephenanthrylene,Benzo[def]chrysene} | 2 | 69 | 1.6% |
| {Chloroform,Dichloromethane} | 2 | 69 | 1.6% |
| {Benzo(e)acephenanthrylene,Benzo(k)fluoranthene} | 2 | 66 | 1.5% |
| {Nickel,Quartz} | 2 | 65 | 1.5% |
| {Hexavalent chromium,Welding fumes} | 2 | 64 | 1.5% |
| {Benzene,Quartz} | 2 | 64 | 1.5% |
| {Benz[a]anthracene,Benzo[def]chrysene} | 2 | 63 | 1.4% |
| {Benzo(e)acephenanthrylene,Benzo(k)fluoranthene,Benzo[def]chrysene} | 3 | 63 | 1.4% |
| {Dichloromethane,Tetrahydrofuran} | 2 | 63 | 1.4% |
| {Cristobalite,Hexavalent chromium} | 2 | 61 | 1.4% |
| {Benz[a]anthracene,Benzo(e)acephenanthrylene} | 2 | 60 | 1.4% |
| {Cristobalite,Refractory ceramic fibers (L>5um D<3um)} | 2 | 59 | 1.3% |
| {Benz[a]anthracene,Benzo(k)fluoranthene} | 2 | 58 | 1.3% |
| {Ethanol,Formaldehyde} | 2 | 58 | 1.3% |
| {Benz[a]anthracene,Benzo(e)acephenanthrylene,Benzo[def]chrysene} | 3 | 57 | 1.3% |
| {Cristobalite,Quartz,Refractory ceramic fibers (L>5um D<3um)} | 3 | 57 | 1.3% |
| {Benz[a]anthracene,Benzo(k)fluoranthene,Benzo[def]chrysene} | 3 | 56 | 1.3% |
| {Cristobalite,Hexavalent chromium,Quartz} | 3 | 55 | 1.2% |
| {Benz[a]anthracene,Benzo(e)acephenanthrylene,Benzo(k)fluoranthene} | 3 | 54 | 1.2% |
| {Ethylbenzene,Formaldehyde} | 2 | 54 | 1.2% |
| {Benz[a]anthracene,Benzo(e)acephenanthrylene,Benzo(k)fluoranthene,Benzo[def]chrysene} | 4 | 53 | 1.2% |
| {Benzene,Chloroform} | 2 | 53 | 1.2% |
| {Ethylbenzene,Hexavalent chromium} | 2 | 52 | 1.2% |
| {Chloroform,Ethylbenzene} | 2 | 51 | 1.2% |
| {Benzene,Styrene} | 2 | 50 | 1.1% |
| {Benzene,Dichloromethane} | 2 | 50 | 1.1% |
| {Hexavalent chromium,Nickel,Welding fumes} | 3 | 49 | 1.1% |
| {Hexavalent chromium,Lead,Nickel} | 3 | 49 | 1.1% |
| {4-Methylpentan-2-one,Hexavalent chromium} | 2 | 48 | 1.1% |
| {Cadmium,Lead} | 2 | 44 | 1.0% |
| {Cobalt,Hexavalent chromium} | 2 | 44 | 1.0% |
| {Chloroform,Tetrahydrofuran} | 2 | 44 | 1.0% |
| {Hexavalent chromium,Refractory ceramic fibers (L>5um D<3um)} | 2 | 42 | 1.0% |
| {Ethylbenzene,Tetrachloroethylene} | 2 | 41 | 0.9% |
| {Ethylbenzene,Wood dust} | 2 | 41 | 0.9% |
| {Ethylbenzene,Tetrahydrofuran} | 2 | 40 | 0.9% |
| {Benzo[def]chrysene,Indeno[1,2,3-cd]pyrene} | 2 | 39 | 0.9% |
| {Benz[a]anthracene,Benzo[def]chrysene,Indeno[1,2,3-cd]pyrene} | 3 | 38 | 0.9% |
| {Benz[a]anthracene,Indeno[1,2,3-cd]pyrene} | 2 | 38 | 0.9% |
| {Cobalt,Hexavalent chromium,Nickel} | 3 | 38 | 0.9% |
| {Benzene,Ethylbenzene,Lead} | 3 | 38 | 0.9% |
| {Dichloromethane,Tetrachloroethylene} | 2 | 37 | 0.8% |
| {4-Methylpentan-2-one,Dichloromethane} | 2 | 37 | 0.8% |
| {Benz[a]anthracene,Benzo(e)acephenanthrylene,Benzo[def]chrysene,Indeno[1,2,3-cd]pyrene} | 4 | 36 | 0.8% |
| {Benz[a]anthracene,Benzo(e)acephenanthrylene,Indeno[1,2,3-cd]pyrene} | 3 | 36 | 0.8% |
| {Benzo(e)acephenanthrylene,Benzo[def]chrysene,Indeno[1,2,3-cd]pyrene} | 3 | 36 | 0.8% |
| {Benzo(e)acephenanthrylene,Indeno[1,2,3-cd]pyrene} | 2 | 36 | 0.8% |
| {Benzene,Tetrahydrofuran} | 2 | 36 | 0.8% |
| {4-Methylpentan-2-one,Ethanol} | 2 | 36 | 0.8% |
| {Lead,Quartz,Refractory ceramic fibers (L>5um D<3um)} | 3 | 35 | 0.8% |
| {Benzene,Refractory ceramic fibers (L>5um D<3um)} | 2 | 35 | 0.8% |
| {Benz[a]anthracene,Benzo(k)fluoranthene,Benzo[def]chrysene,Indeno[1,2,3-cd]pyrene} | 4 | 34 | 0.8% |
| {Benz[a]anthracene,Benzo(k)fluoranthene,Indeno[1,2,3-cd]pyrene} | 3 | 34 | 0.8% |
| {Benzo(k)fluoranthene,Benzo[def]chrysene,Indeno[1,2,3-cd]pyrene} | 3 | 34 | 0.8% |
| {Benzo(k)fluoranthene,Indeno[1,2,3-cd]pyrene} | 2 | 34 | 0.8% |
| {4-Methylpentan-2-one,Benzene} | 2 | 34 | 0.8% |
| {Benz[a]anthracene,Benzo(e)acephenanthrylene,Benzo(k)fluoranthene,Benzo[def]chrysene,Indeno[1,2,3-cd]pyrene} | 5 | 33 | 0.7% |
| {Benz[a]anthracene,Benzo(e)acephenanthrylene,Benzo(k)fluoranthene,Indeno[1,2,3-cd]pyrene} | 4 | 33 | 0.7% |
| {Benzo(e)acephenanthrylene,Benzo(k)fluoranthene,Benzo[def]chrysene,Indeno[1,2,3-cd]pyrene} | 4 | 33 | 0.7% |
| {Benzo(e)acephenanthrylene,Benzo(k)fluoranthene,Indeno[1,2,3-cd]pyrene} | 3 | 33 | 0.7% |
| {Benzene,Cumene} | 2 | 33 | 0.7% |
| {Cobalt,Lead} | 2 | 33 | 0.7% |
| {Formaldehyde,Quartz} | 2 | 32 | 0.7% |
| {Dichloromethane,Quartz} | 2 | 32 | 0.7% |
| {Formaldehyde,Wood dust} | 2 | 31 | 0.7% |
| {Lead,Nickel,Quartz} | 3 | 31 | 0.7% |
| {4-Methylpentan-2-one,Cumene} | 2 | 30 | 0.7% |
| {4-Methylpentan-2-one,Styrene} | 2 | 30 | 0.7% |
| {Dichloromethane,Lead} | 2 | 30 | 0.7% |
| {4-Methylpentan-2-one,Ethylbenzene,Hexavalent chromium} | 3 | 30 | 0.7% |
| {Chloroethylene,Ethylbenzene} | 2 | 28 | 0.6% |
| {Chloroethylene,Chloroform} | 2 | 28 | 0.6% |
| {4-Methylpentan-2-one,Cumene,Ethylbenzene} | 3 | 28 | 0.6% |

## Table S8: Association rules with the largest confidence

List of the 500 association rules with the largest confidence among WS exposed to a minimum of 2 agents. Minimum level of support = 5%, minimum confidence 0.1.

| Antecedent | Consequent | Confidence | Support | N WS |
| --- | --- | --- | --- | --- |
| {Cristobalite} | {Quartz} | 97.5% | 13.4% | 1417 |
| {Ethylbenzene,Toluene} | {Xylene} | 95.0% | 9.0% | 945 |
| {Ethylbenzene} | {Xylene} | 94.6% | 14.7% | 1550 |
| {Zinc} | {Iron} | 93.0% | 6.2% | 656 |
| {Manganese} | {Iron} | 91.5% | 7.3% | 767 |
| {Copper} | {Iron} | 86.6% | 5.3% | 564 |
| {Toluene,Xylene} | {Ethylbenzene} | 72.4% | 9.0% | 945 |
| {Benzene} | {Toluene} | 71.7% | 5.4% | 569 |
| {Xylene} | {Ethylbenzene} | 68.3% | 14.7% | 1550 |
| {Iron} | {Manganese} | 67.5% | 7.3% | 767 |
| {Ethylbenzene,Xylene} | {Toluene} | 61.0% | 9.0% | 945 |
| {Ethylbenzene} | {Toluene} | 60.7% | 9.4% | 995 |
| {Quartz} | {Cristobalite} | 59.7% | 13.4% | 1417 |
| {N-hexane} | {Toluene} | 58.8% | 5.2% | 552 |
| {Iron} | {Zinc} | 57.7% | 6.2% | 656 |
| {Xylene} | {Toluene} | 57.5% | 12.4% | 1305 |
| {Toluene} | {Xylene} | 55.3% | 12.4% | 1305 |
| {Iron} | {Copper} | 49.6% | 5.3% | 564 |
| {Butanone} | {Toluene} | 45.4% | 6.4% | 679 |
| {Butanone} | {Acetone} | 44.4% | 6.3% | 664 |
| {Butanone} | {Xylene} | 42.9% | 6.1% | 642 |
| {Toluene} | {Ethylbenzene} | 42.1% | 9.4% | 995 |
| {Acetone} | {Toluene} | 38.7% | 7.3% | 768 |
| {Acetone} | {Xylene} | 34.0% | 6.4% | 675 |
| {Acetone} | {Butanone} | 33.4% | 6.3% | 664 |
| {Toluene} | {Acetone} | 32.5% | 7.3% | 768 |
| {Xylene} | {Acetone} | 29.7% | 6.4% | 675 |
| {Toluene} | {Butanone} | 28.8% | 6.4% | 679 |
| {Xylene} | {Butanone} | 28.3% | 6.1% | 642 |
| {Toluene} | {Benzene} | 24.1% | 5.4% | 569 |
| {Toluene} | {N-hexane} | 23.4% | 5.2% | 552 |

## Table S9: Association rules with the largest lift

List of the 500 association rules with the largest lift among WS exposed to a minimum of 2 agents. Minimum level of support = 1 %, minimum confidence 0.1.

| Antecedent | Consequent | Lift | Conf (A\(\rightarrow\)C) | Conf (C\(\rightarrow\)A) | Support | N WS |
| --- | --- | --- | --- | --- | --- | --- |
| {Water-soluble metalworking fluids} | {Metalworking fluids (inhalable fraction)} | 62.46 | 93.6% | 82.9% | 1.2% | 131 |
| {Acetaldehyde} | {Formaldehyde} | 31.06 | 97.8% | 39.5% | 1.2% | 131 |
| {1,2,3-Trimethylbenzene} | {Mesitylene} | 23.56 | 69.0% | 44.0% | 1.3% | 136 |
| {1,2,3-Trimethylbenzene,Mesitylene} | {1,2,4-Trimethylbenzene} | 16.16 | 98.5% | 20.8% | 1.3% | 134 |
| {1,2,3-Trimethylbenzene} | {1,2,4-Trimethylbenzene} | 14.74 | 89.8% | 27.5% | 1.7% | 177 |
| {Nickel,Titanium,Total chromium} | {Copper} | 14.70 | 90.8% | 16.6% | 1.0% | 108 |
| {Mesitylene} | {1,2,4-Trimethylbenzene} | 14.39 | 87.7% | 42.1% | 2.6% | 271 |
| {Aluminium,Nickel,Total chromium} | {Copper} | 14.36 | 88.6% | 21.5% | 1.3% | 140 |
| {Titanium,Total chromium} | {Nickel} | 14.16 | 81.0% | 19.7% | 1.1% | 119 |
| {Aluminium,Total chromium} | {Nickel} | 14.03 | 80.2% | 26.2% | 1.5% | 158 |
| {Aluminium,Copper,Titanium} | {Zinc} | 13.81 | 92.3% | 15.3% | 1.0% | 108 |
| {Titanium,Total chromium} | {Copper} | 13.56 | 83.7% | 18.9% | 1.2% | 123 |
| {Propan-2-ol} | {Ethanol} | 13.51 | 49.8% | 37.3% | 1.4% | 145 |
| {Total chromium} | {Nickel} | 13.50 | 77.2% | 60.5% | 3.5% | 365 |
| {Titanium} | {Aluminium} | 13.48 | 60.4% | 34.9% | 1.6% | 165 |
| {Copper,Nickel,Titanium} | {Zinc} | 13.42 | 89.7% | 16.0% | 1.1% | 113 |
| {Copper,Titanium} | {Zinc} | 13.32 | 89.0% | 20.7% | 1.4% | 146 |
| {Copper,Titanium,Total chromium} | {Zinc} | 13.26 | 88.6% | 15.5% | 1.0% | 109 |
| {Aluminium,Nickel} | {Copper} | 13.25 | 81.8% | 28.3% | 1.7% | 184 |
| {Aluminium,Total chromium} | {Copper} | 13.16 | 81.2% | 24.6% | 1.5% | 160 |
| {Aluminium,Copper,Total chromium} | {Zinc} | 13.09 | 87.5% | 19.9% | 1.3% | 140 |
| {Aluminium,Copper,Nickel,Total chromium} | {Zinc} | 13.04 | 87.1% | 17.3% | 1.2% | 122 |
| {Nickel,Titanium} | {Copper} | 13.00 | 80.3% | 19.4% | 1.2% | 126 |
| {Titanium,Total chromium} | {Zinc} | 12.72 | 85.0% | 17.7% | 1.2% | 125 |
| {N-butyl acetate} | {Ethyl acetate} | 12.54 | 47.9% | 37.0% | 1.4% | 149 |
| {Aluminium,Nickel,Total chromium} | {Zinc} | 12.50 | 83.5% | 18.7% | 1.3% | 132 |
| {Nickel,Titanium} | {Zinc} | 12.39 | 82.8% | 18.4% | 1.2% | 130 |
| {Aluminium,Copper,Nickel} | {Zinc} | 12.36 | 82.6% | 21.6% | 1.4% | 152 |
| {Chromium (metal and inorganic Cr(II)/Cr(III) compounds),Copper,Nickel} | {Manganese} | 12.27 | 97.5% | 13.8% | 1.1% | 116 |
| {Aluminium,Total chromium} | {Zinc} | 12.15 | 81.2% | 22.7% | 1.5% | 160 |
| {Copper,Nickel,Total chromium} | {Zinc} | 12.13 | 81.1% | 29.2% | 2.0% | 206 |
| {Aluminium,Nickel,Total chromium,Zinc} | {Manganese} | 12.11 | 96.2% | 15.2% | 1.2% | 127 |
| {Aluminium,Nickel,Total chromium} | {Manganese} | 12.11 | 96.2% | 18.1% | 1.4% | 152 |
| {Aluminium,Copper,Nickel,Total chromium,Zinc} | {Manganese} | 12.07 | 95.9% | 14.0% | 1.1% | 117 |
| {Aluminium,Copper,Nickel,Total chromium} | {Manganese} | 12.05 | 95.7% | 16.0% | 1.3% | 134 |
| {Chromium (metal and inorganic Cr(II)/Cr(III) compounds),Nickel} | {Manganese} | 12.04 | 95.7% | 18.5% | 1.5% | 155 |
| {Titanium} | {Total chromium} | 12.01 | 53.8% | 31.1% | 1.4% | 147 |
| {Aluminium,Copper} | {Zinc} | 12.00 | 80.2% | 32.2% | 2.2% | 227 |
| {Copper,Nickel,Total chromium,Zinc} | {Manganese} | 11.97 | 95.1% | 23.4% | 1.9% | 196 |
| {Aluminium,Titanium} | {Zinc} | 11.97 | 80.0% | 18.7% | 1.3% | 132 |
| {Copper,Total chromium} | {Zinc} | 11.94 | 79.8% | 33.6% | 2.2% | 237 |
| {Copper,Nickel,Titanium,Zinc} | {Manganese} | 11.92 | 94.7% | 12.8% | 1.0% | 107 |
| {Copper,Hexavalent chromium,Total chromium} | {Manganese} | 11.91 | 94.6% | 12.6% | 1.0% | 106 |
| {Chromium (metal and inorganic Cr(II)/Cr(III) compounds),Nickel} | {Copper} | 11.90 | 73.5% | 18.3% | 1.1% | 119 |
| {Nickel,Total chromium,Zinc} | {Manganese} | 11.89 | 94.5% | 26.5% | 2.1% | 222 |
| {Propan-2-ol} | {Ethyl acetate} | 11.87 | 45.4% | 32.8% | 1.3% | 132 |
| {Copper,Titanium,Total chromium} | {Manganese} | 11.87 | 94.3% | 13.8% | 1.1% | 116 |
| {Chromium (metal and inorganic Cr(II)/Cr(III) compounds),Copper} | {Manganese} | 11.87 | 94.3% | 15.8% | 1.3% | 132 |
| {Nickel,Titanium,Total chromium} | {Manganese} | 11.85 | 94.1% | 13.4% | 1.1% | 112 |
| {Hexavalent chromium,Nickel,Zinc} | {Manganese} | 11.84 | 94.1% | 13.2% | 1.1% | 111 |
| {Copper,Nickel,Titanium} | {Manganese} | 11.79 | 93.7% | 14.1% | 1.1% | 118 |
| {Aluminium,Copper,Total chromium,Zinc} | {Manganese} | 11.78 | 93.6% | 15.6% | 1.2% | 131 |
| {Copper,Nickel,Total chromium} | {Manganese} | 11.74 | 93.3% | 28.3% | 2.2% | 237 |
| {Copper,Total chromium,Zinc} | {Manganese} | 11.74 | 93.2% | 26.4% | 2.1% | 221 |
| {Copper,Hexavalent chromium,Nickel} | {Manganese} | 11.72 | 93.2% | 16.2% | 1.3% | 136 |
| {Aluminium,Copper,Total chromium} | {Manganese} | 11.72 | 93.1% | 17.8% | 1.4% | 149 |
| {Cobalt} | {Nickel} | 11.70 | 66.9% | 18.7% | 1.1% | 113 |
| {Titanium,Total chromium,Zinc} | {Manganese} | 11.68 | 92.8% | 13.8% | 1.1% | 116 |
| {Ethanol} | {Ethyl acetate} | 11.64 | 44.5% | 42.9% | 1.6% | 173 |
| {Aluminium,Total chromium,Zinc} | {Manganese} | 11.56 | 91.9% | 17.5% | 1.4% | 147 |
| {Aluminium,Nickel} | {Zinc} | 11.50 | 76.9% | 24.5% | 1.6% | 173 |
| {Aluminium,Total chromium} | {Manganese} | 11.50 | 91.4% | 21.5% | 1.7% | 180 |
| {Aluminium,Titanium} | {Copper} | 11.49 | 70.9% | 18.0% | 1.1% | 117 |
| {Copper,Total chromium} | {Manganese} | 11.44 | 90.9% | 32.2% | 2.6% | 270 |
| {Copper,Hexavalent chromium,Zinc} | {Manganese} | 11.40 | 90.6% | 13.7% | 1.1% | 115 |
| {Copper,Titanium,Zinc} | {Manganese} | 11.38 | 90.4% | 15.8% | 1.3% | 132 |
| {Copper,Hexavalent chromium} | {Manganese} | 11.36 | 90.3% | 18.9% | 1.5% | 158 |
| {Nickel,Titanium,Zinc} | {Manganese} | 11.33 | 90.0% | 14.0% | 1.1% | 117 |
| {Nickel,Total chromium} | {Copper} | 11.27 | 69.6% | 39.0% | 2.4% | 254 |
| {Aluminium,Copper,Nickel,Zinc} | {Manganese} | 11.26 | 89.5% | 16.2% | 1.3% | 136 |
| {Titanium} | {Zinc} | 11.23 | 75.1% | 29.1% | 1.9% | 205 |
| {Total chromium,Zinc} | {Manganese} | 11.23 | 89.2% | 30.5% | 2.4% | 256 |
| {Aluminium,Copper,Nickel} | {Manganese} | 11.22 | 89.1% | 19.6% | 1.6% | 164 |
| {Titanium,Total chromium} | {Manganese} | 11.22 | 89.1% | 15.6% | 1.2% | 131 |
| {Copper,Titanium} | {Manganese} | 11.20 | 89.0% | 17.4% | 1.4% | 146 |
| {Hexavalent chromium,Nickel,Total chromium} | {Manganese} | 11.18 | 88.8% | 14.2% | 1.1% | 119 |
| {Copper,Nickel,Zinc} | {Manganese} | 11.09 | 88.1% | 28.3% | 2.2% | 237 |
| {Aluminium,Nickel,Zinc} | {Manganese} | 11.06 | 87.9% | 18.1% | 1.4% | 152 |
| {Hexavalent chromium,Nickel} | {Manganese} | 10.93 | 86.9% | 20.5% | 1.6% | 172 |
| {Copper,Nickel} | {Manganese} | 10.87 | 86.4% | 40.1% | 3.2% | 336 |
| {Aluminium,Titanium,Zinc} | {Manganese} | 10.87 | 86.4% | 13.6% | 1.1% | 114 |
| {Aluminium,Nickel} | {Manganese} | 10.85 | 86.2% | 23.2% | 1.8% | 194 |
| {Nickel,Total chromium} | {Manganese} | 10.72 | 85.2% | 37.1% | 2.9% | 311 |
| {Nickel,Titanium} | {Manganese} | 10.66 | 84.7% | 15.9% | 1.3% | 133 |
| {Nickel,Zinc} | {Manganese} | 10.55 | 83.8% | 32.7% | 2.6% | 274 |
| {Aluminium,Copper,Zinc} | {Manganese} | 10.48 | 83.3% | 22.6% | 1.8% | 189 |
| {Nickel} | {Copper} | 10.45 | 64.5% | 59.8% | 3.7% | 389 |
| {Hexavalent chromium,Zinc} | {Manganese} | 10.45 | 83.0% | 15.8% | 1.3% | 132 |
| {Copper,Nickel} | {Zinc} | 10.35 | 69.2% | 38.2% | 2.6% | 269 |
| {Aluminium,Copper} | {Manganese} | 10.18 | 80.9% | 27.3% | 2.2% | 229 |
| {Total chromium} | {Copper} | 10.17 | 62.8% | 45.6% | 2.8% | 297 |
| {Methyl methacrylate} | {Styrene} | 10.13 | 49.1% | 21.1% | 1.0% | 108 |
| {Titanium} | {Nickel} | 10.06 | 57.5% | 26.0% | 1.5% | 157 |
| {Copper,Zinc} | {Manganese} | 10.05 | 79.8% | 41.1% | 3.3% | 344 |
| {Welding fumes} | {Manganese} | 10.03 | 79.7% | 12.6% | 1.0% | 106 |
| {Hexavalent chromium,Total chromium} | {Manganese} | 9.99 | 79.4% | 14.7% | 1.2% | 123 |
| {Aluminium,Titanium} | {Manganese} | 9.92 | 78.8% | 15.5% | 1.2% | 130 |
| {Copper} | {Zinc} | 9.90 | 66.2% | 61.1% | 4.1% | 431 |
| {Aluminium} | {Zinc} | 9.87 | 66.0% | 44.3% | 3.0% | 312 |
| {Titanium,Zinc} | {Manganese} | 9.76 | 77.6% | 19.0% | 1.5% | 159 |
| {Titanium} | {Copper} | 9.73 | 60.1% | 25.2% | 1.6% | 164 |
| {Copper} | {Manganese} | 9.71 | 77.1% | 59.9% | 4.8% | 502 |
| {Aluminium} | {Copper} | 9.69 | 59.8% | 43.5% | 2.7% | 283 |
| {Total chromium} | {Manganese} | 9.66 | 76.7% | 43.3% | 3.4% | 363 |
| {Nickel,Total chromium} | {Zinc} | 9.63 | 64.4% | 33.3% | 2.2% | 235 |
| {Nickel} | {Manganese} | 9.52 | 75.6% | 54.4% | 4.3% | 456 |
| {Benzene,Pentane} | {N-hexane} | 9.50 | 84.6% | 12.2% | 1.1% | 115 |
| {Aluminium,Zinc} | {Manganese} | 9.40 | 74.7% | 27.8% | 2.2% | 233 |
| {Aluminium} | {Total chromium} | 9.29 | 41.6% | 41.6% | 1.9% | 197 |
| {Aluminium,Copper,Titanium} | {Iron} | 9.28 | 100.0% | 10.3% | 1.1% | 117 |
| {Aluminium,Manganese,Titanium} | {Iron} | 9.28 | 100.0% | 11.4% | 1.2% | 130 |
| {Manganese,Titanium,Total chromium} | {Iron} | 9.28 | 100.0% | 11.5% | 1.2% | 131 |
| {Manganese,Titanium,Zinc} | {Iron} | 9.28 | 100.0% | 14.0% | 1.5% | 159 |
| {Nickel,Total chromium,Zinc} | {Iron} | 9.28 | 100.0% | 20.7% | 2.2% | 235 |
| {Hexavalent chromium,Total chromium,Zinc} | {Iron} | 9.28 | 100.0% | 9.6% | 1.0% | 109 |
| {Manganese,Total chromium,Zinc} | {Iron} | 9.28 | 100.0% | 22.5% | 2.4% | 256 |
| {Aluminium,Copper,Titanium,Zinc} | {Iron} | 9.28 | 100.0% | 9.5% | 1.0% | 108 |
| {Aluminium,Manganese,Titanium,Zinc} | {Iron} | 9.28 | 100.0% | 10.0% | 1.1% | 114 |
| {Copper,Nickel,Titanium,Total chromium} | {Iron} | 9.28 | 100.0% | 9.5% | 1.0% | 108 |
| {Manganese,Nickel,Titanium,Total chromium} | {Iron} | 9.28 | 100.0% | 9.9% | 1.1% | 112 |
| {Copper,Manganese,Titanium,Total chromium} | {Iron} | 9.28 | 100.0% | 10.2% | 1.1% | 116 |
| {Manganese,Titanium,Total chromium,Zinc} | {Iron} | 9.28 | 100.0% | 10.2% | 1.1% | 116 |
| {Copper,Nickel,Titanium,Zinc} | {Iron} | 9.28 | 100.0% | 9.9% | 1.1% | 113 |
| {Manganese,Nickel,Titanium,Zinc} | {Iron} | 9.28 | 100.0% | 10.3% | 1.1% | 117 |
| {Copper,Manganese,Titanium,Zinc} | {Iron} | 9.28 | 100.0% | 11.6% | 1.3% | 132 |
| {Aluminium,Nickel,Total chromium,Zinc} | {Iron} | 9.28 | 100.0% | 11.6% | 1.3% | 132 |
| {Aluminium,Manganese,Total chromium,Zinc} | {Iron} | 9.28 | 100.0% | 12.9% | 1.4% | 147 |
| {Copper,Nickel,Total chromium,Zinc} | {Iron} | 9.28 | 100.0% | 18.1% | 2.0% | 206 |
| {Manganese,Nickel,Total chromium,Zinc} | {Iron} | 9.28 | 100.0% | 19.5% | 2.1% | 222 |
| {Copper,Manganese,Total chromium,Zinc} | {Iron} | 9.28 | 100.0% | 19.5% | 2.1% | 221 |
| {Copper,Manganese,Nickel,Titanium,Zinc} | {Iron} | 9.28 | 100.0% | 9.4% | 1.0% | 107 |
| {Aluminium,Copper,Nickel,Total chromium,Zinc} | {Iron} | 9.28 | 100.0% | 10.7% | 1.2% | 122 |
| {Aluminium,Manganese,Nickel,Total chromium,Zinc} | {Iron} | 9.28 | 100.0% | 11.2% | 1.2% | 127 |
| {Aluminium,Copper,Manganese,Total chromium,Zinc} | {Iron} | 9.28 | 100.0% | 11.5% | 1.2% | 131 |
| {Copper,Manganese,Nickel,Total chromium,Zinc} | {Iron} | 9.28 | 100.0% | 17.3% | 1.9% | 196 |
| {Aluminium,Copper,Manganese,Nickel,Total chromium,Zinc} | {Iron} | 9.28 | 100.0% | 10.3% | 1.1% | 117 |
| {Copper,Manganese,Nickel,Total chromium} | {Iron} | 9.25 | 99.6% | 20.8% | 2.2% | 236 |
| {Aluminium,Nickel,Zinc} | {Iron} | 9.23 | 99.4% | 15.1% | 1.6% | 172 |
| {Aluminium,Total chromium,Zinc} | {Iron} | 9.23 | 99.4% | 14.0% | 1.5% | 159 |
| {Aluminium,Copper,Nickel,Zinc} | {Iron} | 9.22 | 99.3% | 13.3% | 1.4% | 151 |
| {Aluminium,Manganese,Nickel,Zinc} | {Iron} | 9.22 | 99.3% | 13.3% | 1.4% | 151 |
| {Copper,Titanium,Zinc} | {Iron} | 9.22 | 99.3% | 12.8% | 1.4% | 145 |
| {Copper,Manganese,Titanium} | {Iron} | 9.22 | 99.3% | 12.8% | 1.4% | 145 |
| {Total chromium,Zinc} | {Iron} | 9.22 | 99.3% | 25.1% | 2.7% | 285 |
| {Aluminium,Copper,Total chromium,Zinc} | {Iron} | 9.22 | 99.3% | 12.2% | 1.3% | 139 |
| {Aluminium,Copper,Manganese,Nickel,Zinc} | {Iron} | 9.22 | 99.3% | 11.9% | 1.3% | 135 |
| {Copper,Manganese,Total chromium} | {Iron} | 9.22 | 99.3% | 23.6% | 2.5% | 268 |
| {Aluminium,Copper,Manganese,Nickel,Total chromium} | {Iron} | 9.22 | 99.3% | 11.7% | 1.3% | 133 |
| {Aluminium,Titanium,Zinc} | {Iron} | 9.21 | 99.2% | 11.5% | 1.2% | 131 |
| {Nickel,Titanium,Zinc} | {Iron} | 9.21 | 99.2% | 11.4% | 1.2% | 129 |
| {Copper,Nickel,Titanium} | {Iron} | 9.21 | 99.2% | 11.0% | 1.2% | 125 |
| {Titanium,Total chromium,Zinc} | {Iron} | 9.21 | 99.2% | 10.9% | 1.2% | 124 |
| {Copper,Titanium,Total chromium} | {Iron} | 9.21 | 99.2% | 10.7% | 1.2% | 122 |
| {Nickel,Titanium,Total chromium} | {Iron} | 9.21 | 99.2% | 10.4% | 1.1% | 118 |
| {Copper,Total chromium,Zinc} | {Iron} | 9.21 | 99.2% | 20.7% | 2.2% | 235 |
| {Copper,Manganese,Nickel,Titanium} | {Iron} | 9.21 | 99.2% | 10.3% | 1.1% | 117 |
| {Aluminium,Manganese,Zinc} | {Iron} | 9.20 | 99.1% | 20.3% | 2.2% | 231 |
| {Lead,Total chromium,Zinc} | {Iron} | 9.20 | 99.1% | 9.9% | 1.1% | 112 |
| {Copper,Titanium,Total chromium,Zinc} | {Iron} | 9.20 | 99.1% | 9.5% | 1.0% | 108 |
| {Aluminium,Copper,Manganese,Zinc} | {Iron} | 9.19 | 98.9% | 16.5% | 1.8% | 187 |
| {Copper,Titanium} | {Iron} | 9.17 | 98.8% | 14.3% | 1.5% | 162 |
| {Aluminium,Manganese,Nickel,Total chromium} | {Iron} | 9.16 | 98.7% | 13.2% | 1.4% | 150 |
| {Aluminium,Copper,Manganese,Total chromium} | {Iron} | 9.16 | 98.7% | 12.9% | 1.4% | 147 |
| {Aluminium,Copper,Nickel,Total chromium} | {Iron} | 9.15 | 98.6% | 12.1% | 1.3% | 138 |
| {Manganese,Nickel,Zinc} | {Iron} | 9.15 | 98.5% | 23.8% | 2.6% | 270 |
| {Lead,Manganese,Zinc} | {Iron} | 9.15 | 98.5% | 11.6% | 1.3% | 132 |
| {Manganese,Nickel,Titanium} | {Iron} | 9.14 | 98.5% | 11.5% | 1.2% | 131 |
| {Hexavalent chromium,Manganese,Zinc} | {Iron} | 9.14 | 98.5% | 11.4% | 1.2% | 130 |
| {Copper,Nickel,Total chromium} | {Iron} | 9.14 | 98.4% | 22.0% | 2.4% | 250 |
| {Hexavalent chromium,Manganese,Total chromium} | {Iron} | 9.13 | 98.4% | 10.7% | 1.1% | 121 |
| {Lead,Manganese,Total chromium} | {Iron} | 9.13 | 98.3% | 10.4% | 1.1% | 118 |
| {Copper,Lead,Manganese,Zinc} | {Iron} | 9.13 | 98.3% | 10.4% | 1.1% | 118 |
| {Hexavalent chromium,Manganese,Nickel,Total chromium} | {Iron} | 9.13 | 98.3% | 10.3% | 1.1% | 117 |
| {Copper,Manganese,Nickel,Zinc} | {Iron} | 9.13 | 98.3% | 20.5% | 2.2% | 233 |
| {Copper,Hexavalent chromium,Manganese,Zinc} | {Iron} | 9.12 | 98.3% | 9.9% | 1.1% | 113 |
| {Lead,Manganese,Nickel,Total chromium} | {Iron} | 9.12 | 98.2% | 9.6% | 1.0% | 109 |
| {Hexavalent chromium,Manganese,Nickel,Zinc} | {Iron} | 9.12 | 98.2% | 9.6% | 1.0% | 109 |
| {Aluminium,Nickel,Total chromium} | {Iron} | 9.11 | 98.1% | 13.6% | 1.5% | 155 |
| {Copper,Manganese,Zinc} | {Iron} | 9.10 | 98.0% | 29.7% | 3.2% | 337 |
| {Manganese,Titanium} | {Iron} | 9.09 | 97.9% | 16.4% | 1.8% | 186 |
| {Copper,Lead,Manganese} | {Iron} | 9.08 | 97.8% | 11.7% | 1.3% | 133 |
| {Total chromium} | {Zinc} | 9.08 | 60.7% | 40.7% | 2.7% | 287 |
| {Manganese,Zinc} | {Iron} | 9.07 | 97.7% | 40.7% | 4.4% | 462 |
| {Copper,Hexavalent chromium,Zinc} | {Iron} | 9.07 | 97.6% | 10.9% | 1.2% | 124 |
| {Aluminium,Lead,Zinc} | {Iron} | 9.06 | 97.6% | 10.7% | 1.1% | 121 |
| {Aluminium,Copper,Total chromium} | {Iron} | 9.05 | 97.5% | 13.7% | 1.5% | 156 |
| {Hexavalent chromium,Nickel,Zinc} | {Iron} | 9.05 | 97.5% | 10.1% | 1.1% | 115 |
| {Copper,Nickel,Zinc} | {Iron} | 9.04 | 97.4% | 23.1% | 2.5% | 262 |
| {Aluminium,Copper,Zinc} | {Iron} | 9.04 | 97.4% | 19.5% | 2.1% | 221 |
| {Copper,Lead,Manganese,Nickel} | {Iron} | 9.03 | 97.3% | 9.5% | 1.0% | 108 |
| {Titanium,Total chromium} | {Iron} | 9.03 | 97.3% | 12.6% | 1.4% | 143 |
| {Copper,Lead,Total chromium} | {Iron} | 9.03 | 97.2% | 9.3% | 1.0% | 106 |
| {Aluminium,Manganese,Total chromium} | {Iron} | 9.03 | 97.2% | 15.4% | 1.7% | 175 |
| {Manganese,Nickel,Total chromium} | {Iron} | 9.02 | 97.1% | 26.6% | 2.9% | 302 |
| {Titanium,Zinc} | {Iron} | 9.01 | 97.1% | 17.5% | 1.9% | 199 |
| {Lead,Manganese} | {Iron} | 9.00 | 97.0% | 14.2% | 1.5% | 161 |
| {Aluminium,Copper,Manganese,Nickel} | {Iron} | 9.00 | 97.0% | 14.0% | 1.5% | 159 |
| {Aluminium,Copper,Manganese} | {Iron} | 9.00 | 96.9% | 19.5% | 2.1% | 222 |
| {Aluminium,Zinc} | {Iron} | 8.99 | 96.8% | 26.6% | 2.9% | 302 |
| {Manganese,Total chromium} | {Iron} | 8.98 | 96.7% | 30.9% | 3.3% | 351 |
| {Nickel,Zinc} | {Iron} | 8.97 | 96.6% | 27.8% | 3.0% | 316 |
| {Copper,Total chromium} | {Iron} | 8.97 | 96.6% | 25.3% | 2.7% | 287 |
| {Copper,Lead,Nickel,Zinc} | {Iron} | 8.97 | 96.6% | 9.9% | 1.1% | 113 |
| {Copper,Hexavalent chromium,Total chromium} | {Iron} | 8.95 | 96.4% | 9.5% | 1.0% | 108 |
| {Aluminium,Manganese,Nickel} | {Iron} | 8.95 | 96.4% | 16.5% | 1.8% | 187 |
| {Aluminium,Copper,Nickel} | {Iron} | 8.93 | 96.2% | 15.6% | 1.7% | 177 |
| {Lead,Nickel,Zinc} | {Iron} | 8.92 | 96.1% | 10.9% | 1.2% | 124 |
| {Lead,Manganese,Nickel} | {Iron} | 8.92 | 96.1% | 10.9% | 1.2% | 124 |
| {Aluminium,Nickel} | {Iron} | 8.91 | 96.0% | 19.0% | 2.0% | 216 |
| {Aluminium,Total chromium} | {Iron} | 8.91 | 95.9% | 16.6% | 1.8% | 189 |
| {Aluminium,Manganese} | {Iron} | 8.90 | 95.8% | 26.4% | 2.8% | 300 |
| {Aluminium,Titanium} | {Iron} | 8.89 | 95.8% | 13.9% | 1.5% | 158 |
| {Hexavalent chromium,Zinc} | {Iron} | 8.88 | 95.6% | 13.4% | 1.4% | 152 |
| {Nickel,Titanium} | {Iron} | 8.87 | 95.5% | 13.2% | 1.4% | 150 |
| {Copper,Lead,Nickel} | {Iron} | 8.80 | 94.7% | 11.1% | 1.2% | 126 |
| {Copper,Zinc} | {Iron} | 8.79 | 94.7% | 35.9% | 3.9% | 408 |
| {Titanium} | {Manganese} | 8.76 | 69.6% | 22.7% | 1.8% | 190 |
| {Lead,Nickel,Total chromium} | {Iron} | 8.74 | 94.2% | 9.9% | 1.1% | 113 |
| {Zinc} | {Iron} | 8.64 | 93.0% | 57.7% | 6.2% | 656 |
| {Copper,Lead,Zinc} | {Iron} | 8.63 | 93.0% | 14.0% | 1.5% | 159 |
| {Nickel,Total chromium} | {Iron} | 8.60 | 92.6% | 29.8% | 3.2% | 338 |
| {Aluminium,Copper} | {Iron} | 8.60 | 92.6% | 23.1% | 2.5% | 262 |
| {Hexavalent chromium,Nickel,Total chromium} | {Iron} | 8.59 | 92.5% | 10.9% | 1.2% | 124 |
| {Aluminium,Copper,Lead} | {Iron} | 8.59 | 92.5% | 9.8% | 1.1% | 111 |
| {Lead,Total chromium} | {Iron} | 8.57 | 92.3% | 11.5% | 1.2% | 131 |
| {Copper,Manganese} | {Iron} | 8.56 | 92.2% | 40.8% | 4.4% | 463 |
| {Lead,Zinc} | {Iron} | 8.53 | 91.9% | 18.0% | 1.9% | 205 |
| {Copper,Hexavalent chromium,Manganese,Nickel} | {Iron} | 8.53 | 91.9% | 11.0% | 1.2% | 125 |
| {Copper,Hexavalent chromium,Manganese} | {Iron} | 8.52 | 91.8% | 12.8% | 1.4% | 145 |
| {Copper,Manganese,Nickel} | {Iron} | 8.51 | 91.7% | 27.1% | 2.9% | 308 |
| {Manganese} | {Iron} | 8.50 | 91.5% | 67.5% | 7.3% | 767 |
| {Hexavalent chromium,Manganese} | {Iron} | 8.45 | 91.0% | 16.9% | 1.8% | 192 |
| {Zinc} | {Manganese} | 8.44 | 67.1% | 56.4% | 4.5% | 473 |
| {Aluminium,Lead} | {Iron} | 8.39 | 90.3% | 12.3% | 1.3% | 140 |
| {Manganese,Nickel} | {Iron} | 8.37 | 90.1% | 36.2% | 3.9% | 411 |
| {Hexavalent chromium,Manganese,Nickel} | {Iron} | 8.37 | 90.1% | 13.6% | 1.5% | 155 |
| {Titanium} | {Iron} | 8.37 | 90.1% | 21.7% | 2.3% | 246 |
| {Aluminium} | {Manganese} | 8.33 | 66.2% | 37.4% | 3.0% | 313 |
| {Total chromium} | {Iron} | 8.32 | 89.6% | 37.3% | 4.0% | 424 |
| {Aluminium} | {Nickel} | 8.32 | 47.6% | 37.3% | 2.1% | 225 |
| {Copper,Nickel} | {Iron} | 8.31 | 89.5% | 30.6% | 3.3% | 348 |
| {Copper,Hexavalent chromium} | {Iron} | 8.28 | 89.1% | 13.7% | 1.5% | 156 |
| {Copper,Hexavalent chromium,Nickel} | {Iron} | 8.27 | 89.0% | 11.4% | 1.2% | 130 |
| {Aluminium} | {Iron} | 8.22 | 88.6% | 36.9% | 4.0% | 419 |
| {Lead,Nickel} | {Iron} | 8.22 | 88.6% | 12.9% | 1.4% | 147 |
| {Hexavalent chromium,Total chromium} | {Iron} | 8.21 | 88.4% | 12.1% | 1.3% | 137 |
| {Copper,Lead} | {Iron} | 8.16 | 87.9% | 15.9% | 1.7% | 181 |
| {Nickel} | {Zinc} | 8.11 | 54.2% | 46.4% | 3.1% | 327 |
| {Copper} | {Iron} | 8.04 | 86.6% | 49.6% | 5.3% | 564 |
| {Aliphatic and alicyclic hydrocarbons} | {Ethyl acetate} | 8.01 | 30.6% | 31.8% | 1.2% | 128 |
| {Hexavalent chromium,Nickel} | {Iron} | 7.83 | 84.3% | 14.7% | 1.6% | 167 |
| {Nickel} | {Iron} | 7.82 | 84.2% | 44.7% | 4.8% | 508 |
| {1-Methoxypropan-2-ol} | {2-Methoxy-1-methylethyl acetate} | 7.76 | 47.6% | 50.1% | 3.1% | 324 |
| {Chromium (metal and inorganic Cr(II)/Cr(III) compounds),Manganese,Nickel} | {Iron} | 7.43 | 80.0% | 10.9% | 1.2% | 124 |
| {Cyclohexane} | {Heptane} | 7.41 | 42.9% | 40.5% | 2.3% | 247 |
| {Chromium (metal and inorganic Cr(II)/Cr(III) compounds),Manganese} | {Iron} | 7.32 | 78.8% | 12.8% | 1.4% | 145 |
| {Chromium (metal and inorganic Cr(II)/Cr(III) compounds),Nickel} | {Iron} | 7.28 | 78.4% | 11.2% | 1.2% | 127 |
| {N-butyl acetate} | {4-Methylpentan-2-one} | 7.13 | 35.4% | 21.0% | 1.0% | 110 |
| {Chromium (metal and inorganic Cr(II)/Cr(III) compounds),Copper} | {Iron} | 7.03 | 75.7% | 9.3% | 1.0% | 106 |
| {N-butyl acetate} | {2-Methoxy-1-methylethyl acetate} | 6.92 | 42.4% | 20.4% | 1.3% | 132 |
| {Cyclohexane,Heptane} | {N-hexane} | 6.82 | 60.7% | 16.0% | 1.4% | 150 |
| {Cobalt} | {Iron} | 6.43 | 69.2% | 10.3% | 1.1% | 117 |
| {4-Methylpentan-2-one} | {2-Methoxy-1-methylethyl acetate} | 6.14 | 37.7% | 30.4% | 1.9% | 197 |
| {Pentane} | {N-hexane} | 5.78 | 51.5% | 23.9% | 2.1% | 224 |
| {1,2,4-Trimethylbenzene,Butanone} | {Ethylbenzene} | 5.49 | 85.2% | 7.4% | 1.1% | 121 |
| {1,2,4-Trimethylbenzene,N-hexane} | {Ethylbenzene} | 5.48 | 85.1% | 8.4% | 1.3% | 137 |
| {Cyclohexane} | {N-hexane} | 5.44 | 48.4% | 29.7% | 2.6% | 279 |
| {1,2,4-Trimethylbenzene,Heptane} | {Ethylbenzene} | 5.38 | 83.6% | 7.4% | 1.2% | 122 |
| {Ethyl acetate,N-butyl acetate} | {Butanone} | 5.35 | 75.8% | 7.6% | 1.1% | 113 |
| {2-Methoxy-1-methylethyl acetate,4-Methylpentan-2-one,Butanone} | {Ethylbenzene} | 5.27 | 81.9% | 6.9% | 1.1% | 113 |
| {2-Methoxy-1-methylethyl acetate,4-Methylpentan-2-one} | {Ethylbenzene} | 5.26 | 81.7% | 9.8% | 1.5% | 161 |
| {2-Methoxy-1-methylethyl acetate,N-butyl acetate} | {Ethylbenzene} | 5.17 | 80.3% | 6.5% | 1.0% | 106 |
| {Pentane} | {Heptane} | 5.17 | 29.9% | 21.3% | 1.2% | 130 |
| {Chromium (metal and inorganic Cr(II)/Cr(III) compounds)} | {Hexavalent chromium} | 5.13 | 57.1% | 34.8% | 3.9% | 408 |
| {1,2,4-Trimethylbenzene,2-Methoxy-1-methylethyl acetate} | {Ethylbenzene} | 5.07 | 78.7% | 6.5% | 1.0% | 107 |
| {1,2,4-Trimethylbenzene,Acetone} | {Ethylbenzene} | 5.06 | 78.6% | 9.2% | 1.4% | 151 |
| {1,2,4-Trimethylbenzene,Benzene} | {Ethylbenzene} | 4.95 | 76.9% | 7.3% | 1.1% | 120 |
| {2-Methoxy-1-methylethyl acetate,4-Methylpentan-2-one} | {Butanone} | 4.94 | 70.1% | 9.2% | 1.3% | 138 |
| {1-Methoxypropan-2-ol,4-Methylpentan-2-one} | {Ethylbenzene} | 4.93 | 76.6% | 6.8% | 1.1% | 111 |
| {4-Methylpentan-2-one,Acetone,Butanone} | {Ethylbenzene} | 4.73 | 73.4% | 7.1% | 1.1% | 116 |
| {Butanone,N-butyl acetate} | {Ethylbenzene} | 4.66 | 72.4% | 8.2% | 1.3% | 134 |
| {Mesitylene} | {Ethylbenzene} | 4.65 | 72.2% | 13.6% | 2.1% | 223 |
| {Heptane} | {N-hexane} | 4.62 | 41.1% | 26.7% | 2.4% | 251 |
| {Ethylbenzene,N-butyl acetate} | {Xylene} | 4.62 | 99.5% | 9.0% | 1.9% | 204 |
| {Butanone,Ethylbenzene,N-butyl acetate} | {Xylene} | 4.61 | 99.3% | 5.9% | 1.3% | 133 |
| {1,2,4-Trimethylbenzene,Mesitylene} | {Ethylbenzene} | 4.61 | 71.6% | 11.8% | 1.8% | 194 |
| {Ethyl acetate,Ethylbenzene} | {Xylene} | 4.61 | 99.2% | 5.8% | 1.3% | 132 |
| {Acetone,Ethylbenzene,N-butyl acetate} | {Xylene} | 4.61 | 99.2% | 5.4% | 1.2% | 123 |
| {4-Methylpentan-2-one,Acetone,Butanone,Ethylbenzene} | {Xylene} | 4.60 | 99.1% | 5.1% | 1.1% | 115 |
| {2-Methoxy-1-methylethyl acetate,4-Methylpentan-2-one,Butanone,Ethylbenzene} | {Xylene} | 4.60 | 99.1% | 4.9% | 1.1% | 112 |
| {2-Methoxy-1-methylethyl acetate,4-Methylpentan-2-one,Ethylbenzene} | {Xylene} | 4.59 | 98.8% | 7.0% | 1.5% | 159 |
| {Cyclohexane,Ethylbenzene,Heptane} | {Xylene} | 4.56 | 98.2% | 4.9% | 1.1% | 111 |
| {1-Methoxypropan-2-ol,4-Methylpentan-2-one,Ethylbenzene} | {Xylene} | 4.56 | 98.2% | 4.8% | 1.0% | 109 |
| {4-Methylpentan-2-one,Acetone,Ethylbenzene} | {Xylene} | 4.56 | 98.1% | 6.9% | 1.5% | 156 |
| {Benzene,Ethylbenzene,N-hexane} | {Xylene} | 4.55 | 98.0% | 6.5% | 1.4% | 147 |
| {Pentane} | {Cyclohexane} | 4.55 | 24.8% | 18.8% | 1.0% | 108 |
| {1,2,4-Trimethylbenzene,Ethylbenzene,N-hexane} | {Xylene} | 4.54 | 97.8% | 5.9% | 1.3% | 134 |
| {Acetone,Ethylbenzene,N-hexane} | {Xylene} | 4.54 | 97.8% | 5.8% | 1.2% | 131 |
| {Aliphatic and alicyclic hydrocarbons,Ethylbenzene} | {Xylene} | 4.53 | 97.6% | 5.3% | 1.1% | 120 |
| {4-Methylpentan-2-one,Butanone,Ethylbenzene} | {Xylene} | 4.53 | 97.5% | 8.5% | 1.8% | 194 |
| {1,2,3-Trimethylbenzene,1,2,4-Trimethylbenzene,Ethylbenzene} | {Xylene} | 4.52 | 97.4% | 4.9% | 1.1% | 111 |
| {1,2,4-Trimethylbenzene,Acetone,Ethylbenzene} | {Xylene} | 4.52 | 97.4% | 6.5% | 1.4% | 147 |
| {4-Methylpentan-2-one,Ethylbenzene} | {Xylene} | 4.52 | 97.2% | 13.9% | 3.0% | 316 |
| {2-Methoxy-1-methylethyl acetate,Acetone,Ethylbenzene} | {Xylene} | 4.51 | 97.1% | 6.0% | 1.3% | 136 |
| {Butanone,Styrene} | {Acetone} | 4.51 | 84.9% | 5.4% | 1.0% | 107 |
| {Cyclohexane,Ethylbenzene} | {Xylene} | 4.50 | 96.9% | 8.3% | 1.8% | 189 |
| {Ethylbenzene,N-hexane} | {Xylene} | 4.50 | 96.9% | 12.5% | 2.7% | 283 |
| {1,2,4-Trimethylbenzene,Ethylbenzene,Mesitylene} | {Xylene} | 4.50 | 96.9% | 8.3% | 1.8% | 188 |
| {1,2,4-Trimethylbenzene,Ethylbenzene,Heptane} | {Xylene} | 4.49 | 96.7% | 5.2% | 1.1% | 118 |
| {Acetone,Butanone,Ethylbenzene} | {Xylene} | 4.49 | 96.7% | 10.4% | 2.2% | 236 |
| {4-Methylpentan-2-one,Acetone} | {Ethylbenzene} | 4.49 | 69.7% | 9.7% | 1.5% | 159 |
| {Ethylbenzene,N-hexane,Pentane} | {Xylene} | 4.48 | 96.5% | 4.8% | 1.0% | 110 |
| {1,2,4-Trimethylbenzene,Ethylbenzene} | {Xylene} | 4.46 | 96.1% | 18.2% | 3.9% | 414 |
| {1,2,4-Trimethylbenzene,Benzene,Ethylbenzene} | {Xylene} | 4.45 | 95.8% | 5.1% | 1.1% | 115 |
| {Butanone,Ethylbenzene} | {Xylene} | 4.44 | 95.6% | 20.0% | 4.3% | 454 |
| {Acetone,Cyclohexane,Ethylbenzene} | {Xylene} | 4.44 | 95.5% | 4.7% | 1.0% | 107 |
| {Ethylbenzene,Mesitylene} | {Xylene} | 4.44 | 95.5% | 9.4% | 2.0% | 213 |
| {Acetone,N-butyl acetate} | {Ethylbenzene} | 4.44 | 68.9% | 7.6% | 1.2% | 124 |
| {1,2,3-Trimethylbenzene,Ethylbenzene} | {Xylene} | 4.42 | 95.2% | 5.3% | 1.1% | 120 |
| {2-Methoxy-1-methylethyl acetate,Ethylbenzene} | {Xylene} | 4.42 | 95.1% | 13.7% | 2.9% | 311 |
| {2-Methoxy-1-methylethyl acetate,Butanone,Ethylbenzene} | {Xylene} | 4.42 | 95.1% | 7.7% | 1.6% | 174 |
| {1,2,4-Trimethylbenzene,Butanone,Ethylbenzene} | {Xylene} | 4.41 | 95.0% | 5.1% | 1.1% | 115 |
| {Acetone,Ethylbenzene} | {Xylene} | 4.41 | 95.0% | 20.9% | 4.5% | 475 |
| {Benzene,Ethylbenzene} | {Xylene} | 4.41 | 94.9% | 13.1% | 2.8% | 298 |
| {Ethylbenzene,Heptane} | {Xylene} | 4.40 | 94.7% | 8.6% | 1.8% | 195 |
| {Ethylbenzene} | {Xylene} | 4.39 | 94.6% | 68.3% | 14.7% | 1550 |
| {1-Methoxypropan-2-ol,Ethylbenzene} | {Xylene} | 4.39 | 94.6% | 10.8% | 2.3% | 246 |
| {Benzene,Heptane,Xylene} | {Toluene} | 4.39 | 98.2% | 4.7% | 1.0% | 110 |
| {1-Methoxypropan-2-ol,2-Methoxy-1-methylethyl acetate,Ethylbenzene} | {Xylene} | 4.37 | 94.0% | 6.2% | 1.3% | 141 |
| {1-Methoxypropan-2-ol,Butanone,Ethylbenzene} | {Xylene} | 4.35 | 93.8% | 5.9% | 1.3% | 135 |
| {Benzene,Ethylbenzene,N-hexane,Xylene} | {Toluene} | 4.35 | 97.3% | 6.1% | 1.4% | 143 |
| {Ethylbenzene,N-hexane,Pentane,Xylene} | {Toluene} | 4.35 | 97.3% | 4.5% | 1.0% | 107 |
| {2-Methoxy-1-methylethyl acetate,4-Methylpentan-2-one,Butanone} | {Xylene} | 4.34 | 93.5% | 5.7% | 1.2% | 129 |
| {Cristobalite} | {Quartz} | 4.33 | 97.5% | 59.7% | 13.4% | 1417 |
| {1,2,4-Trimethylbenzene,N-hexane} | {Xylene} | 4.33 | 93.2% | 6.6% | 1.4% | 150 |
| {1,2,4-Trimethylbenzene,4-Methylpentan-2-one} | {Xylene} | 4.32 | 93.1% | 4.8% | 1.0% | 108 |
| {1,2,4-Trimethylbenzene,N-hexane,Xylene} | {Toluene} | 4.32 | 96.7% | 6.1% | 1.4% | 145 |
| {1,2,4-Trimethylbenzene} | {Ethylbenzene} | 4.32 | 67.0% | 26.3% | 4.1% | 431 |
| {1,2,4-Trimethylbenzene,Ethylbenzene,N-hexane,Xylene} | {Toluene} | 4.30 | 96.3% | 5.5% | 1.2% | 129 |
| {4-Methylpentan-2-one} | {1-Methoxypropan-2-ol} | 4.29 | 27.7% | 21.3% | 1.4% | 145 |
| {Benzene,Ethylbenzene,N-hexane} | {Toluene} | 4.29 | 96.0% | 6.1% | 1.4% | 144 |
| {1,2,4-Trimethylbenzene,Benzene,Ethylbenzene,Xylene} | {Toluene} | 4.27 | 95.7% | 4.7% | 1.0% | 110 |
| {Benzene} | {N-hexane} | 4.27 | 38.0% | 32.2% | 2.9% | 302 |
| {1,2,4-Trimethylbenzene,Ethylbenzene,N-hexane} | {Toluene} | 4.27 | 95.6% | 5.5% | 1.2% | 131 |
| {Ethylbenzene,N-hexane,Pentane} | {Toluene} | 4.27 | 95.6% | 4.6% | 1.0% | 109 |
| {Benzene,N-hexane,Xylene} | {Toluene} | 4.26 | 95.4% | 7.9% | 1.8% | 186 |
| {Cristobalite,Tridymite} | {Quartz} | 4.25 | 95.5% | 7.2% | 1.6% | 171 |
| {N-butyl acetate} | {Ethylbenzene} | 4.24 | 65.9% | 12.5% | 1.9% | 205 |
| {2-Methoxy-1-methylethyl acetate,4-Methylpentan-2-one} | {Xylene} | 4.24 | 91.4% | 7.9% | 1.7% | 180 |
| {4-Methylpentan-2-one,Butanone} | {Ethylbenzene} | 4.24 | 65.9% | 12.1% | 1.9% | 199 |
| {N-hexane,Pentane,Xylene} | {Toluene} | 4.24 | 94.9% | 5.5% | 1.2% | 129 |
| {1,2,4-Trimethylbenzene,Benzene,Xylene} | {Toluene} | 4.23 | 94.8% | 5.4% | 1.2% | 127 |
| {Cyclohexane,Ethylbenzene,Heptane} | {Toluene} | 4.23 | 94.7% | 4.5% | 1.0% | 107 |
| {Benzene,Ethylbenzene,Xylene} | {Toluene} | 4.23 | 94.6% | 11.9% | 2.7% | 282 |
| {1,2,4-Trimethylbenzene,Butanone} | {Xylene} | 4.22 | 90.8% | 5.7% | 1.2% | 129 |
| {Ethylbenzene,Pentane} | {Xylene} | 4.20 | 90.5% | 5.9% | 1.3% | 134 |
| {1,2,4-Trimethylbenzene,Heptane} | {Xylene} | 4.20 | 90.4% | 5.8% | 1.3% | 132 |
| {Benzene,Cyclohexane} | {Toluene} | 4.20 | 94.0% | 4.6% | 1.0% | 109 |
| {Benzene,N-hexane,Pentane} | {Toluene} | 4.20 | 93.9% | 4.6% | 1.0% | 108 |
| {N-butyl acetate} | {Butanone} | 4.19 | 59.5% | 12.4% | 1.8% | 185 |
| {1,2,4-Trimethylbenzene,N-hexane} | {Toluene} | 4.19 | 93.8% | 6.4% | 1.4% | 151 |
| {1,2,4-Trimethylbenzene,Benzene,Ethylbenzene} | {Toluene} | 4.17 | 93.3% | 4.7% | 1.1% | 112 |
| {Ethylbenzene,Pentane,Xylene} | {Toluene} | 4.17 | 93.3% | 5.3% | 1.2% | 125 |
| {1-Methoxypropan-2-ol,4-Methylpentan-2-one} | {Xylene} | 4.16 | 89.7% | 5.7% | 1.2% | 130 |
| {Pentane} | {Benzene} | 4.15 | 31.3% | 17.1% | 1.3% | 136 |
| {1,2,3-Trimethylbenzene,1,2,4-Trimethylbenzene} | {Ethylbenzene} | 4.15 | 64.4% | 7.0% | 1.1% | 114 |
| {1,2,4-Trimethylbenzene,Heptane,Xylene} | {Toluene} | 4.13 | 92.4% | 5.2% | 1.2% | 122 |
| {1,2,4-Trimethylbenzene,Ethylbenzene,Heptane,Xylene} | {Toluene} | 4.13 | 92.4% | 4.6% | 1.0% | 109 |
| {Benzene,Ethylbenzene} | {Toluene} | 4.13 | 92.4% | 12.3% | 2.7% | 290 |
| {4-Methylpentan-2-one,Acetone,Butanone,Ethylbenzene} | {Toluene} | 4.12 | 92.2% | 4.5% | 1.0% | 107 |
| {1,2,3-Trimethylbenzene} | {Ethylbenzene} | 4.12 | 64.0% | 7.7% | 1.2% | 126 |
| {4-Methylpentan-2-one,Acetone,Butanone,Ethylbenzene,Xylene} | {Toluene} | 4.12 | 92.2% | 4.5% | 1.0% | 106 |
| {2-Methoxy-1-methylethyl acetate,N-butyl acetate} | {Xylene} | 4.12 | 88.6% | 5.2% | 1.1% | 117 |
| {4-Methylpentan-2-one,Acetone,Butanone} | {Xylene} | 4.12 | 88.6% | 6.2% | 1.3% | 140 |
| {Benzene,Xylene} | {Toluene} | 4.12 | 92.1% | 15.8% | 3.5% | 374 |
| {1,2,4-Trimethylbenzene,Ethylbenzene,Heptane} | {Toluene} | 4.10 | 91.8% | 4.7% | 1.1% | 112 |
| {Acetone,Ethylbenzene,N-hexane} | {Toluene} | 4.10 | 91.8% | 5.2% | 1.2% | 123 |
| {1,2,4-Trimethylbenzene,Benzene} | {Toluene} | 4.09 | 91.7% | 6.1% | 1.4% | 143 |
| {Heptane,N-hexane,Xylene} | {Toluene} | 4.09 | 91.7% | 5.1% | 1.1% | 121 |
| {Acetone,Ethylbenzene,N-hexane,Xylene} | {Toluene} | 4.09 | 91.6% | 5.1% | 1.1% | 120 |
| {4-Methylpentan-2-one} | {Butanone} | 4.07 | 57.7% | 20.2% | 2.9% | 302 |
| {Benzene,Heptane} | {Toluene} | 4.06 | 90.8% | 5.5% | 1.2% | 129 |
| {Tridymite} | {Quartz} | 4.05 | 91.1% | 14.2% | 3.2% | 338 |
| {4-Methylpentan-2-one,Acetone,Ethylbenzene} | {Toluene} | 4.05 | 90.6% | 6.1% | 1.4% | 144 |
| {Benzene,Pentane} | {Toluene} | 4.04 | 90.4% | 5.2% | 1.2% | 123 |
| {4-Methylpentan-2-one,Acetone,Ethylbenzene,Xylene} | {Toluene} | 4.04 | 90.4% | 6.0% | 1.3% | 141 |
| {Ethylbenzene,Heptane} | {Toluene} | 4.03 | 90.3% | 7.9% | 1.8% | 186 |
| {Ethylbenzene,Heptane,Xylene} | {Toluene} | 4.03 | 90.3% | 7.5% | 1.7% | 176 |
| {Ethyl acetate,Ethylbenzene} | {Toluene} | 4.03 | 90.2% | 5.1% | 1.1% | 120 |
| {Ethyl acetate,Ethylbenzene,Xylene} | {Toluene} | 4.03 | 90.2% | 5.0% | 1.1% | 119 |
| {Ethyl acetate} | {Butanone} | 4.02 | 57.1% | 15.4% | 2.2% | 230 |
| {Butanone,N-butyl acetate} | {Xylene} | 4.02 | 86.5% | 7.0% | 1.5% | 160 |
| {Ethyl acetate,N-butyl acetate,Xylene} | {Toluene} | 4.01 | 89.8% | 4.5% | 1.0% | 106 |
| {Ethylbenzene,N-hexane,Xylene} | {Toluene} | 4.01 | 89.8% | 10.8% | 2.4% | 254 |
| {Acetone,Butanone,N-butyl acetate} | {Xylene} | 4.01 | 86.3% | 5.0% | 1.1% | 113 |
| {4-Methylpentan-2-one} | {Ethylbenzene} | 4.00 | 62.1% | 19.8% | 3.1% | 325 |
| {Ethylbenzene,N-hexane} | {Toluene} | 3.99 | 89.4% | 11.1% | 2.5% | 261 |
| {1,2,4-Trimethylbenzene,Benzene} | {Xylene} | 3.99 | 85.9% | 5.9% | 1.3% | 134 |
| {2-Methoxy-1-methylethyl acetate,Acetone} | {Ethylbenzene} | 3.97 | 61.7% | 8.5% | 1.3% | 140 |
| {Chromium (metal and inorganic Cr(II)/Cr(III) compounds)} | {Nickel} | 3.97 | 22.7% | 26.9% | 1.5% | 162 |
| {Cyclohexane,Heptane,Xylene} | {Toluene} | 3.97 | 88.8% | 5.4% | 1.2% | 127 |
| {1,2,4-Trimethylbenzene,Acetone} | {Xylene} | 3.97 | 85.4% | 7.2% | 1.6% | 164 |
| {1,2,4-Trimethylbenzene,2-Methoxy-1-methylethyl acetate} | {Xylene} | 3.96 | 85.3% | 5.1% | 1.1% | 116 |
| {Styrene} | {Acetone} | 3.96 | 74.6% | 19.2% | 3.6% | 381 |
| {Ethyl acetate,N-butyl acetate} | {Acetone} | 3.96 | 74.5% | 5.6% | 1.1% | 111 |
| {Heptane} | {1,2,4-Trimethylbenzene} | 3.93 | 23.9% | 22.7% | 1.4% | 146 |
| {4-Methylpentan-2-one,Acetone,Butanone,Xylene} | {Toluene} | 3.92 | 87.9% | 5.2% | 1.2% | 123 |
| {Pentane,Xylene} | {Toluene} | 3.90 | 87.4% | 6.7% | 1.5% | 159 |
| {Acetone,N-hexane,Xylene} | {Toluene} | 3.90 | 87.3% | 5.8% | 1.3% | 138 |
| {Acetone,N-butyl acetate} | {Xylene} | 3.90 | 83.9% | 6.6% | 1.4% | 151 |
| {Ethylbenzene,Pentane} | {Toluene} | 3.89 | 87.2% | 5.5% | 1.2% | 129 |
| {Benzene,N-hexane} | {Toluene} | 3.88 | 86.8% | 11.1% | 2.5% | 262 |
| {2-Methoxy-1-methylethyl acetate,Butanone} | {Ethylbenzene} | 3.84 | 59.6% | 11.2% | 1.7% | 183 |
| {Butanone,Ethylbenzene,N-butyl acetate} | {Toluene} | 3.83 | 85.8% | 4.9% | 1.1% | 115 |
| {4-Methylpentan-2-one,Acetone} | {Xylene} | 3.83 | 82.5% | 8.3% | 1.8% | 188 |
| {Acetone,Heptane,Xylene} | {Toluene} | 3.83 | 85.7% | 4.8% | 1.1% | 114 |
| {Butanone,Ethylbenzene,N-butyl acetate,Xylene} | {Toluene} | 3.83 | 85.7% | 4.8% | 1.1% | 114 |
| {1,2,4-Trimethylbenzene,Acetone,Ethylbenzene,Xylene} | {Toluene} | 3.83 | 85.7% | 5.3% | 1.2% | 126 |
| {1,2,4-Trimethylbenzene,Heptane} | {Toluene} | 3.82 | 85.6% | 5.3% | 1.2% | 125 |
| {Copper,Nickel,Total chromium} | {Hexavalent chromium} | 3.82 | 42.5% | 9.2% | 1.0% | 108 |
| {4-Methylpentan-2-one,Acetone,Xylene} | {Toluene} | 3.80 | 85.1% | 6.8% | 1.5% | 160 |
| {N-hexane,Xylene} | {Toluene} | 3.80 | 85.1% | 14.0% | 3.1% | 330 |
| {1,2,4-Trimethylbenzene,Acetone,Ethylbenzene} | {Toluene} | 3.79 | 84.8% | 5.4% | 1.2% | 128 |
| {1,2,4-Trimethylbenzene,Acetone,Xylene} | {Toluene} | 3.79 | 84.8% | 5.9% | 1.3% | 139 |
| {Butanone,Ethyl acetate,Xylene} | {Toluene} | 3.77 | 84.3% | 4.8% | 1.1% | 113 |
| {Acetone,Ethyl acetate,Xylene} | {Toluene} | 3.77 | 84.3% | 4.8% | 1.1% | 113 |
| {Butanone,N-butyl acetate} | {Acetone} | 3.76 | 70.8% | 6.6% | 1.2% | 131 |
| {4-Methylpentan-2-one,Butanone,Ethylbenzene,Xylene} | {Toluene} | 3.75 | 84.0% | 6.9% | 1.5% | 163 |
| {4-Methylpentan-2-one,Butanone,Ethylbenzene} | {Toluene} | 3.75 | 83.9% | 7.1% | 1.6% | 167 |
| {2-Methoxy-1-methylethyl acetate,4-Methylpentan-2-one,Butanone,Xylene} | {Toluene} | 3.74 | 83.7% | 4.6% | 1.0% | 108 |
| {Cyclohexane,Ethylbenzene,Xylene} | {Toluene} | 3.71 | 83.1% | 6.6% | 1.5% | 157 |
| {2-Methoxy-1-methylethyl acetate,Acetone,Butanone} | {Xylene} | 3.70 | 79.7% | 5.0% | 1.1% | 114 |
| {1,2,4-Trimethylbenzene,Mesitylene} | {Xylene} | 3.70 | 79.7% | 9.5% | 2.0% | 216 |
| {Acetone,Benzene} | {Toluene} | 3.69 | 82.6% | 4.8% | 1.1% | 114 |
| {Cyclohexane,Ethylbenzene} | {Toluene} | 3.69 | 82.6% | 6.8% | 1.5% | 161 |
| {Acetone,Butanone,N-butyl acetate} | {Toluene} | 3.68 | 82.4% | 4.6% | 1.0% | 108 |
| {2-Methoxy-1-methylethyl acetate,Acetone,Ethylbenzene,Xylene} | {Toluene} | 3.68 | 82.4% | 4.7% | 1.1% | 112 |
| {Ethyl acetate,N-butyl acetate} | {Xylene} | 3.68 | 79.2% | 5.2% | 1.1% | 118 |
| {4-Methylpentan-2-one,Acetone,Butanone} | {Toluene} | 3.68 | 82.3% | 5.5% | 1.2% | 130 |
| {N-butyl acetate} | {Xylene} | 3.67 | 79.1% | 10.8% | 2.3% | 246 |
| {Benzene,Heptane} | {Xylene} | 3.66 | 78.9% | 4.9% | 1.1% | 112 |
| {2-Methoxy-1-methylethyl acetate,4-Methylpentan-2-one,Ethylbenzene} | {Toluene} | 3.66 | 82.0% | 5.6% | 1.3% | 132 |
| {Heptane,Xylene} | {Toluene} | 3.66 | 81.9% | 9.4% | 2.1% | 221 |
| {2-Methoxy-1-methylethyl acetate,4-Methylpentan-2-one,Ethylbenzene,Xylene} | {Toluene} | 3.65 | 81.8% | 5.5% | 1.2% | 130 |
| {Mesitylene} | {Xylene} | 3.65 | 78.6% | 10.7% | 2.3% | 243 |
| {Methyl methacrylate} | {Acetone} | 3.65 | 68.6% | 7.6% | 1.4% | 151 |
| {Acetone,Cyclohexane,Xylene} | {Toluene} | 3.64 | 81.5% | 4.7% | 1.0% | 110 |
| {4-Methylpentan-2-one} | {1,2,4-Trimethylbenzene} | 3.64 | 22.2% | 18.0% | 1.1% | 116 |
| {2-Methoxy-1-methylethyl acetate,Acetone,Ethylbenzene} | {Toluene} | 3.64 | 81.4% | 4.8% | 1.1% | 114 |
| {4-Methylpentan-2-one,Butanone} | {Xylene} | 3.63 | 78.1% | 10.4% | 2.2% | 236 |
| {Cyclohexane,N-hexane,Xylene} | {Toluene} | 3.62 | 81.1% | 4.5% | 1.0% | 107 |
| {Butanone,N-butyl acetate,Xylene} | {Toluene} | 3.60 | 80.6% | 5.5% | 1.2% | 129 |
| {Ethyl acetate,N-butyl acetate} | {Toluene} | 3.60 | 80.5% | 5.1% | 1.1% | 120 |
| {2-Methoxy-1-methylethyl acetate,4-Methylpentan-2-one,Butanone} | {Toluene} | 3.59 | 80.4% | 4.7% | 1.1% | 111 |
| {Cyclohexanone} | {Butanone} | 3.57 | 50.7% | 7.3% | 1.0% | 109 |
| {Ethyl acetate,Xylene} | {Toluene} | 3.57 | 79.9% | 6.1% | 1.4% | 143 |
| {4-Methylpentan-2-one,Butanone,Xylene} | {Toluene} | 3.56 | 79.7% | 8.0% | 1.8% | 188 |
| {Cumene} | {Xylene} | 3.56 | 76.6% | 4.8% | 1.0% | 108 |
| {1,2,4-Trimethylbenzene} | {Xylene} | 3.55 | 76.5% | 21.7% | 4.7% | 492 |
| {Copper,Nickel,Zinc} | {Hexavalent chromium} | 3.54 | 39.4% | 9.0% | 1.0% | 106 |
| {1-Methoxypropan-2-ol,2-Methoxy-1-methylethyl acetate} | {Butanone} | 3.53 | 50.0% | 10.8% | 1.5% | 162 |
| {2-Methoxy-1-methylethyl acetate,4-Methylpentan-2-one,Xylene} | {Toluene} | 3.52 | 78.9% | 6.0% | 1.3% | 142 |
| {2-Methoxy-1-methylethyl acetate,Butanone,Ethylbenzene,Xylene} | {Toluene} | 3.52 | 78.7% | 5.8% | 1.3% | 137 |
| {Butanone,Cyclohexane} | {Acetone} | 3.51 | 66.1% | 5.9% | 1.1% | 117 |
| {Acetone,Butanone,Ethylbenzene,Xylene} | {Toluene} | 3.50 | 78.4% | 7.8% | 1.8% | 185 |
| {Tridymite} | {Cristobalite} | 3.50 | 48.2% | 12.3% | 1.7% | 179 |
| {Butanone,Ethyl acetate} | {Acetone} | 3.49 | 65.7% | 7.6% | 1.4% | 151 |
| {4-Methylpentan-2-one,Ethylbenzene} | {Toluene} | 3.48 | 77.8% | 10.7% | 2.4% | 253 |
| {Butanone,N-butyl acetate} | {Toluene} | 3.48 | 77.8% | 6.1% | 1.4% | 144 |
| {1,2,4-Trimethylbenzene,Acetone} | {Toluene} | 3.47 | 77.6% | 6.3% | 1.4% | 149 |
| {4-Methylpentan-2-one,Ethylbenzene,Xylene} | {Toluene} | 3.46 | 77.5% | 10.4% | 2.3% | 245 |
| {Acetone,N-butyl acetate,Xylene} | {Toluene} | 3.46 | 77.5% | 5.0% | 1.1% | 117 |
| {Acetone,Butanone,Ethylbenzene} | {Toluene} | 3.46 | 77.5% | 8.0% | 1.8% | 189 |
| {Cyclohexane,Xylene} | {Toluene} | 3.45 | 77.3% | 8.3% | 1.9% | 197 |
| {4-Methylpentan-2-one,Acetone} | {Toluene} | 3.45 | 77.2% | 7.5% | 1.7% | 176 |
| {2-Methoxy-1-methylethyl acetate} | {1,2,4-Trimethylbenzene} | 3.45 | 21.0% | 21.2% | 1.3% | 136 |
| {4-Methylpentan-2-one} | {Xylene} | 3.45 | 74.2% | 17.1% | 3.7% | 388 |
| {2-Methoxy-1-methylethyl acetate,Acetone,Xylene} | {Toluene} | 3.44 | 77.1% | 5.4% | 1.2% | 128 |
| {2-Methoxy-1-methylethyl acetate,Butanone,Ethylbenzene} | {Toluene} | 3.44 | 77.0% | 6.0% | 1.3% | 141 |
| {Total chromium,Zinc} | {Hexavalent chromium} | 3.41 | 38.0% | 9.3% | 1.0% | 109 |
| {2-Methoxy-1-methylethyl acetate,4-Methylpentan-2-one} | {Toluene} | 3.40 | 76.1% | 6.4% | 1.4% | 150 |
| {Ethylbenzene,N-butyl acetate} | {Toluene} | 3.40 | 76.1% | 6.6% | 1.5% | 156 |
| {Copper,Nickel,Zinc} | {Lead} | 3.40 | 43.5% | 8.7% | 1.1% | 117 |
| {2-Methoxy-1-methylethyl acetate,Acetone} | {Xylene} | 3.40 | 73.1% | 7.3% | 1.6% | 166 |
| {Ethylbenzene,N-butyl acetate,Xylene} | {Toluene} | 3.39 | 76.0% | 6.6% | 1.5% | 155 |
| {Copper,Total chromium} | {Hexavalent chromium} | 3.39 | 37.7% | 9.5% | 1.1% | 112 |
| {Acetone,Ethylbenzene,Xylene} | {Toluene} | 3.39 | 75.8% | 15.2% | 3.4% | 360 |
| {1,2,3-Trimethylbenzene,1,2,4-Trimethylbenzene} | {Xylene} | 3.38 | 72.9% | 5.7% | 1.2% | 129 |
| {Acetone,N-butyl acetate} | {Toluene} | 3.38 | 75.6% | 5.8% | 1.3% | 136 |
| {Copper,Nickel} | {Hexavalent chromium} | 3.37 | 37.5% | 12.4% | 1.4% | 146 |
| {1,2,3-Trimethylbenzene} | {Xylene} | 3.37 | 72.6% | 6.3% | 1.4% | 143 |
| {Acetone,Ethylbenzene} | {Toluene} | 3.35 | 75.0% | 15.9% | 3.6% | 375 |
| {2-Methoxy-1-methylethyl acetate} | {Butanone} | 3.35 | 47.4% | 20.5% | 2.9% | 307 |
| {Aliphatic and alicyclic hydrocarbons,Xylene} | {Toluene} | 3.33 | 74.5% | 5.2% | 1.2% | 123 |
| {Aluminium,Copper} | {Lead} | 3.31 | 42.4% | 8.9% | 1.1% | 120 |
| {Nickel,Total chromium} | {Hexavalent chromium} | 3.30 | 36.7% | 11.4% | 1.3% | 134 |
| {N-hexane,Pentane} | {Toluene} | 3.29 | 73.7% | 7.0% | 1.6% | 165 |
| {2-Methoxy-1-methylethyl acetate,Butanone,Xylene} | {Toluene} | 3.29 | 73.6% | 6.7% | 1.5% | 159 |
| {Acetone,Butanone,Ethyl acetate} | {Toluene} | 3.28 | 73.5% | 4.7% | 1.1% | 111 |
| {N-hexane,Pentane} | {Ethylbenzene} | 3.28 | 50.9% | 7.0% | 1.1% | 114 |

## FIM and association rules by industry

### 081 - Quarrying of stone, sand and clay

275 work situations with coexposures; 35 different chemical agents involved.

#### Prevalence of exposure by agent

#### Frequent itemset mining

4 identified. The 4 most frequent itemsets are listed below

| Itemset | N WS | % Support |
| --- | --- | --- |
| {Cristobalite,Quartz} | 243 | 88.4% |
| {Quartz,Tridymite} | 53 | 19.3% |
| {Cristobalite,Quartz,Tridymite} | 38 | 13.8% |
| {Cristobalite,Tridymite} | 38 | 13.8% |

#### Association rules mining

##### Association rules with the largest confidence among WS exposed to a minimum of 2 agents. Minimum level of support = 0.1%, minimum confidence 0.1.

| Antecedent | Consequent | % Confidence | % Support | N WS |
| --- | --- | --- | --- | --- |
| {Tridymite} | {Quartz} | 100.0% | 19.3% | 53 |
| {Cristobalite} | {Quartz} | 100.0% | 88.4% | 243 |
| {Cristobalite,Tridymite} | {Quartz} | 100.0% | 13.8% | 38 |
| {Quartz} | {Cristobalite} | 90.0% | 88.4% | 243 |
| {Tridymite} | {Cristobalite} | 71.7% | 13.8% | 38 |
| {Quartz,Tridymite} | {Cristobalite} | 71.7% | 13.8% | 38 |
| {Quartz} | {Tridymite} | 19.6% | 19.3% | 53 |
| {Cristobalite} | {Tridymite} | 15.6% | 13.8% | 38 |
| {Cristobalite,Quartz} | {Tridymite} | 15.6% | 13.8% | 38 |

##### Association rules with the largest lift among WS exposed to a minimum of 2 agents. Minimum level of support = 0.1 %, minimum confidence 0.1.

| Antecedent | Consequent | Lift | % Conf (A\(\rightarrow\)C) | % Conf (C\(\rightarrow\)A) | % Support | N WS |
| --- | --- | --- | --- | --- | --- | --- |
| {Tridymite} | {Quartz} | 101.9 | 100.0% | 19.6% | 19.3% | 53 |
| {Cristobalite} | {Quartz} | 101.9 | 100.0% | 90.0% | 88.4% | 243 |
| {Cristobalite,Tridymite} | {Quartz} | 101.9 | 100.0% | 14.1% | 13.8% | 38 |
| {Tridymite} | {Cristobalite} | 81.1 | 71.7% | 15.6% | 13.8% | 38 |

### 162 - Manufacture of products of wood, cork, straw and plaiting materials

101 work situations with coexposures; 54 different chemical agents involved.

#### Prevalence of exposure by agent

#### Frequent itemset mining

123 identified. The 123 most frequent itemsets are listed below

| Itemset | N WS | % Support |
| --- | --- | --- |
| {Lead,Wood dust} | 27 | 26.7% |
| {Formaldehyde,Wood dust} | 19 | 18.8% |
| {Benz[a]anthracene,Benzo(e)acephenanthrylene,Benzo(k)fluoranthene,Benzo[def]chrysene,Benzo[ghi]perylene,Indeno[1,2,3-cd]pyrene} | 15 | 14.9% |
| {Benz[a]anthracene,Benzo(e)acephenanthrylene,Benzo(k)fluoranthene,Benzo[def]chrysene,Indeno[1,2,3-cd]pyrene} | 15 | 14.9% |
| {Benz[a]anthracene,Benzo(e)acephenanthrylene,Benzo(k)fluoranthene,Benzo[def]chrysene,Benzo[ghi]perylene} | 15 | 14.9% |
| {Benz[a]anthracene,Benzo(e)acephenanthrylene,Benzo(k)fluoranthene,Benzo[ghi]perylene,Indeno[1,2,3-cd]pyrene} | 15 | 14.9% |
| {Benz[a]anthracene,Benzo(e)acephenanthrylene,Benzo(k)fluoranthene,Indeno[1,2,3-cd]pyrene} | 15 | 14.9% |
| {Benz[a]anthracene,Benzo(e)acephenanthrylene,Benzo(k)fluoranthene,Benzo[ghi]perylene} | 15 | 14.9% |
| {Benz[a]anthracene,Benzo(e)acephenanthrylene,Benzo(k)fluoranthene,Benzo[def]chrysene} | 15 | 14.9% |
| {Benz[a]anthracene,Benzo(e)acephenanthrylene,Benzo[def]chrysene,Benzo[ghi]perylene,Indeno[1,2,3-cd]pyrene} | 15 | 14.9% |
| {Benz[a]anthracene,Benzo(e)acephenanthrylene,Benzo[def]chrysene,Indeno[1,2,3-cd]pyrene} | 15 | 14.9% |
| {Benz[a]anthracene,Benzo(e)acephenanthrylene,Benzo[def]chrysene,Benzo[ghi]perylene} | 15 | 14.9% |
| {Benz[a]anthracene,Benzo(e)acephenanthrylene,Benzo[ghi]perylene,Indeno[1,2,3-cd]pyrene} | 15 | 14.9% |
| {Benz[a]anthracene,Benzo(e)acephenanthrylene,Indeno[1,2,3-cd]pyrene} | 15 | 14.9% |
| {Benz[a]anthracene,Benzo(e)acephenanthrylene,Benzo[ghi]perylene} | 15 | 14.9% |
| {Benz[a]anthracene,Benzo(e)acephenanthrylene,Benzo[def]chrysene} | 15 | 14.9% |
| {Benz[a]anthracene,Benzo(e)acephenanthrylene,Benzo(k)fluoranthene} | 15 | 14.9% |
| {Benz[a]anthracene,Benzo(k)fluoranthene,Benzo[def]chrysene,Benzo[ghi]perylene,Indeno[1,2,3-cd]pyrene} | 15 | 14.9% |
| {Benz[a]anthracene,Benzo(k)fluoranthene,Benzo[def]chrysene,Indeno[1,2,3-cd]pyrene} | 15 | 14.9% |
| {Benz[a]anthracene,Benzo(k)fluoranthene,Benzo[def]chrysene,Benzo[ghi]perylene} | 15 | 14.9% |
| {Benz[a]anthracene,Benzo(k)fluoranthene,Benzo[ghi]perylene,Indeno[1,2,3-cd]pyrene} | 15 | 14.9% |
| {Benz[a]anthracene,Benzo(k)fluoranthene,Indeno[1,2,3-cd]pyrene} | 15 | 14.9% |
| {Benz[a]anthracene,Benzo(k)fluoranthene,Benzo[ghi]perylene} | 15 | 14.9% |
| {Benz[a]anthracene,Benzo(k)fluoranthene,Benzo[def]chrysene} | 15 | 14.9% |
| {Benz[a]anthracene,Benzo[def]chrysene,Benzo[ghi]perylene,Indeno[1,2,3-cd]pyrene} | 15 | 14.9% |
| {Benz[a]anthracene,Benzo[def]chrysene,Indeno[1,2,3-cd]pyrene} | 15 | 14.9% |
| {Benz[a]anthracene,Benzo[def]chrysene,Benzo[ghi]perylene} | 15 | 14.9% |
| {Benz[a]anthracene,Benzo[ghi]perylene,Indeno[1,2,3-cd]pyrene} | 15 | 14.9% |
| {Benz[a]anthracene,Indeno[1,2,3-cd]pyrene} | 15 | 14.9% |
| {Benz[a]anthracene,Benzo[ghi]perylene} | 15 | 14.9% |
| {Benz[a]anthracene,Benzo[def]chrysene} | 15 | 14.9% |
| {Benz[a]anthracene,Benzo(k)fluoranthene} | 15 | 14.9% |
| {Benz[a]anthracene,Benzo(e)acephenanthrylene} | 15 | 14.9% |
| {Benzo(e)acephenanthrylene,Benzo(k)fluoranthene,Benzo[def]chrysene,Benzo[ghi]perylene,Indeno[1,2,3-cd]pyrene} | 15 | 14.9% |
| {Benzo(e)acephenanthrylene,Benzo(k)fluoranthene,Benzo[def]chrysene,Indeno[1,2,3-cd]pyrene} | 15 | 14.9% |
| {Benzo(e)acephenanthrylene,Benzo(k)fluoranthene,Benzo[def]chrysene,Benzo[ghi]perylene} | 15 | 14.9% |
| {Benzo(e)acephenanthrylene,Benzo(k)fluoranthene,Benzo[ghi]perylene,Indeno[1,2,3-cd]pyrene} | 15 | 14.9% |
| {Benzo(e)acephenanthrylene,Benzo(k)fluoranthene,Indeno[1,2,3-cd]pyrene} | 15 | 14.9% |
| {Benzo(e)acephenanthrylene,Benzo(k)fluoranthene,Benzo[ghi]perylene} | 15 | 14.9% |
| {Benzo(e)acephenanthrylene,Benzo(k)fluoranthene,Benzo[def]chrysene} | 15 | 14.9% |
| {Benzo(e)acephenanthrylene,Benzo[def]chrysene,Benzo[ghi]perylene,Indeno[1,2,3-cd]pyrene} | 15 | 14.9% |
| {Benzo(e)acephenanthrylene,Benzo[def]chrysene,Indeno[1,2,3-cd]pyrene} | 15 | 14.9% |
| {Benzo(e)acephenanthrylene,Benzo[def]chrysene,Benzo[ghi]perylene} | 15 | 14.9% |
| {Benzo(e)acephenanthrylene,Benzo[ghi]perylene,Indeno[1,2,3-cd]pyrene} | 15 | 14.9% |
| {Benzo(e)acephenanthrylene,Indeno[1,2,3-cd]pyrene} | 15 | 14.9% |
| {Benzo(e)acephenanthrylene,Benzo[ghi]perylene} | 15 | 14.9% |
| {Benzo(e)acephenanthrylene,Benzo[def]chrysene} | 15 | 14.9% |
| {Benzo(e)acephenanthrylene,Benzo(k)fluoranthene} | 15 | 14.9% |
| {Benzo(k)fluoranthene,Benzo[def]chrysene,Benzo[ghi]perylene,Indeno[1,2,3-cd]pyrene} | 15 | 14.9% |
| {Benzo(k)fluoranthene,Benzo[def]chrysene,Indeno[1,2,3-cd]pyrene} | 15 | 14.9% |
| {Benzo(k)fluoranthene,Benzo[def]chrysene,Benzo[ghi]perylene} | 15 | 14.9% |
| {Benzo(k)fluoranthene,Benzo[ghi]perylene,Indeno[1,2,3-cd]pyrene} | 15 | 14.9% |
| {Benzo(k)fluoranthene,Indeno[1,2,3-cd]pyrene} | 15 | 14.9% |
| {Benzo(k)fluoranthene,Benzo[ghi]perylene} | 15 | 14.9% |
| {Benzo(k)fluoranthene,Benzo[def]chrysene} | 15 | 14.9% |
| {Benzo[def]chrysene,Benzo[ghi]perylene,Indeno[1,2,3-cd]pyrene} | 15 | 14.9% |
| {Benzo[def]chrysene,Indeno[1,2,3-cd]pyrene} | 15 | 14.9% |
| {Benzo[def]chrysene,Benzo[ghi]perylene} | 15 | 14.9% |
| {Benzo[ghi]perylene,Indeno[1,2,3-cd]pyrene} | 15 | 14.9% |
| {Toluene,Wood dust} | 13 | 12.9% |
| {Benz[a]anthracene,Benzo(e)acephenanthrylene,Benzo(k)fluoranthene,Benzo[def]chrysene,Benzo[ghi]perylene,Indeno[1,2,3-cd]pyrene,Wood dust} | 11 | 10.9% |
| {Benz[a]anthracene,Benzo(e)acephenanthrylene,Benzo(k)fluoranthene,Benzo[def]chrysene,Benzo[ghi]perylene,Wood dust} | 11 | 10.9% |
| {Benz[a]anthracene,Benzo(e)acephenanthrylene,Benzo(k)fluoranthene,Benzo[def]chrysene,Indeno[1,2,3-cd]pyrene,Wood dust} | 11 | 10.9% |
| {Benz[a]anthracene,Benzo(e)acephenanthrylene,Benzo(k)fluoranthene,Benzo[def]chrysene,Wood dust} | 11 | 10.9% |
| {Benz[a]anthracene,Benzo(e)acephenanthrylene,Benzo(k)fluoranthene,Benzo[ghi]perylene,Indeno[1,2,3-cd]pyrene,Wood dust} | 11 | 10.9% |
| {Benz[a]anthracene,Benzo(e)acephenanthrylene,Benzo(k)fluoranthene,Benzo[ghi]perylene,Wood dust} | 11 | 10.9% |
| {Benz[a]anthracene,Benzo(e)acephenanthrylene,Benzo(k)fluoranthene,Indeno[1,2,3-cd]pyrene,Wood dust} | 11 | 10.9% |
| {Benz[a]anthracene,Benzo(e)acephenanthrylene,Benzo(k)fluoranthene,Wood dust} | 11 | 10.9% |
| {Benz[a]anthracene,Benzo(e)acephenanthrylene,Benzo[def]chrysene,Benzo[ghi]perylene,Indeno[1,2,3-cd]pyrene,Wood dust} | 11 | 10.9% |
| {Benz[a]anthracene,Benzo(e)acephenanthrylene,Benzo[def]chrysene,Benzo[ghi]perylene,Wood dust} | 11 | 10.9% |
| {Benz[a]anthracene,Benzo(e)acephenanthrylene,Benzo[def]chrysene,Indeno[1,2,3-cd]pyrene,Wood dust} | 11 | 10.9% |
| {Benz[a]anthracene,Benzo(e)acephenanthrylene,Benzo[def]chrysene,Wood dust} | 11 | 10.9% |
| {Benz[a]anthracene,Benzo(e)acephenanthrylene,Benzo[ghi]perylene,Indeno[1,2,3-cd]pyrene,Wood dust} | 11 | 10.9% |
| {Benz[a]anthracene,Benzo(e)acephenanthrylene,Benzo[ghi]perylene,Wood dust} | 11 | 10.9% |
| {Benz[a]anthracene,Benzo(e)acephenanthrylene,Indeno[1,2,3-cd]pyrene,Wood dust} | 11 | 10.9% |
| {Benz[a]anthracene,Benzo(e)acephenanthrylene,Wood dust} | 11 | 10.9% |
| {Benz[a]anthracene,Benzo(k)fluoranthene,Benzo[def]chrysene,Benzo[ghi]perylene,Indeno[1,2,3-cd]pyrene,Wood dust} | 11 | 10.9% |
| {Benz[a]anthracene,Benzo(k)fluoranthene,Benzo[def]chrysene,Benzo[ghi]perylene,Wood dust} | 11 | 10.9% |
| {Benz[a]anthracene,Benzo(k)fluoranthene,Benzo[def]chrysene,Indeno[1,2,3-cd]pyrene,Wood dust} | 11 | 10.9% |
| {Benz[a]anthracene,Benzo(k)fluoranthene,Benzo[def]chrysene,Wood dust} | 11 | 10.9% |
| {Benz[a]anthracene,Benzo(k)fluoranthene,Benzo[ghi]perylene,Indeno[1,2,3-cd]pyrene,Wood dust} | 11 | 10.9% |
| {Benz[a]anthracene,Benzo(k)fluoranthene,Benzo[ghi]perylene,Wood dust} | 11 | 10.9% |
| {Benz[a]anthracene,Benzo(k)fluoranthene,Indeno[1,2,3-cd]pyrene,Wood dust} | 11 | 10.9% |
| {Benz[a]anthracene,Benzo(k)fluoranthene,Wood dust} | 11 | 10.9% |
| {Benz[a]anthracene,Benzo[def]chrysene,Benzo[ghi]perylene,Indeno[1,2,3-cd]pyrene,Wood dust} | 11 | 10.9% |
| {Benz[a]anthracene,Benzo[def]chrysene,Benzo[ghi]perylene,Wood dust} | 11 | 10.9% |
| {Benz[a]anthracene,Benzo[def]chrysene,Indeno[1,2,3-cd]pyrene,Wood dust} | 11 | 10.9% |
| {Benz[a]anthracene,Benzo[def]chrysene,Wood dust} | 11 | 10.9% |
| {Benz[a]anthracene,Benzo[ghi]perylene,Indeno[1,2,3-cd]pyrene,Wood dust} | 11 | 10.9% |
| {Benz[a]anthracene,Benzo[ghi]perylene,Wood dust} | 11 | 10.9% |
| {Benz[a]anthracene,Indeno[1,2,3-cd]pyrene,Wood dust} | 11 | 10.9% |
| {Benz[a]anthracene,Wood dust} | 11 | 10.9% |
| {Benzo(e)acephenanthrylene,Benzo(k)fluoranthene,Benzo[def]chrysene,Benzo[ghi]perylene,Indeno[1,2,3-cd]pyrene,Wood dust} | 11 | 10.9% |
| {Benzo(e)acephenanthrylene,Benzo(k)fluoranthene,Benzo[def]chrysene,Benzo[ghi]perylene,Wood dust} | 11 | 10.9% |
| {Benzo(e)acephenanthrylene,Benzo(k)fluoranthene,Benzo[def]chrysene,Indeno[1,2,3-cd]pyrene,Wood dust} | 11 | 10.9% |
| {Benzo(e)acephenanthrylene,Benzo(k)fluoranthene,Benzo[def]chrysene,Wood dust} | 11 | 10.9% |
| {Benzo(e)acephenanthrylene,Benzo(k)fluoranthene,Benzo[ghi]perylene,Indeno[1,2,3-cd]pyrene,Wood dust} | 11 | 10.9% |
| {Benzo(e)acephenanthrylene,Benzo(k)fluoranthene,Benzo[ghi]perylene,Wood dust} | 11 | 10.9% |
| {Benzo(e)acephenanthrylene,Benzo(k)fluoranthene,Indeno[1,2,3-cd]pyrene,Wood dust} | 11 | 10.9% |
| {Benzo(e)acephenanthrylene,Benzo(k)fluoranthene,Wood dust} | 11 | 10.9% |
| {Benzo(e)acephenanthrylene,Benzo[def]chrysene,Benzo[ghi]perylene,Indeno[1,2,3-cd]pyrene,Wood dust} | 11 | 10.9% |
| {Benzo(e)acephenanthrylene,Benzo[def]chrysene,Benzo[ghi]perylene,Wood dust} | 11 | 10.9% |
| {Benzo(e)acephenanthrylene,Benzo[def]chrysene,Indeno[1,2,3-cd]pyrene,Wood dust} | 11 | 10.9% |
| {Benzo(e)acephenanthrylene,Benzo[def]chrysene,Wood dust} | 11 | 10.9% |
| {Benzo(e)acephenanthrylene,Benzo[ghi]perylene,Indeno[1,2,3-cd]pyrene,Wood dust} | 11 | 10.9% |
| {Benzo(e)acephenanthrylene,Benzo[ghi]perylene,Wood dust} | 11 | 10.9% |
| {Benzo(e)acephenanthrylene,Indeno[1,2,3-cd]pyrene,Wood dust} | 11 | 10.9% |
| {Benzo(e)acephenanthrylene,Wood dust} | 11 | 10.9% |
| {Benzo(k)fluoranthene,Benzo[def]chrysene,Benzo[ghi]perylene,Indeno[1,2,3-cd]pyrene,Wood dust} | 11 | 10.9% |
| {Benzo(k)fluoranthene,Benzo[def]chrysene,Benzo[ghi]perylene,Wood dust} | 11 | 10.9% |
| {Benzo(k)fluoranthene,Benzo[def]chrysene,Indeno[1,2,3-cd]pyrene,Wood dust} | 11 | 10.9% |
| {Benzo(k)fluoranthene,Benzo[def]chrysene,Wood dust} | 11 | 10.9% |
| {Benzo(k)fluoranthene,Benzo[ghi]perylene,Indeno[1,2,3-cd]pyrene,Wood dust} | 11 | 10.9% |
| {Benzo(k)fluoranthene,Benzo[ghi]perylene,Wood dust} | 11 | 10.9% |
| {Benzo(k)fluoranthene,Indeno[1,2,3-cd]pyrene,Wood dust} | 11 | 10.9% |
| {Benzo(k)fluoranthene,Wood dust} | 11 | 10.9% |
| {Benzo[def]chrysene,Benzo[ghi]perylene,Indeno[1,2,3-cd]pyrene,Wood dust} | 11 | 10.9% |
| {Benzo[def]chrysene,Benzo[ghi]perylene,Wood dust} | 11 | 10.9% |
| {Benzo[def]chrysene,Indeno[1,2,3-cd]pyrene,Wood dust} | 11 | 10.9% |
| {Benzo[def]chrysene,Wood dust} | 11 | 10.9% |
| {Benzo[ghi]perylene,Indeno[1,2,3-cd]pyrene,Wood dust} | 11 | 10.9% |
| {Benzo[ghi]perylene,Wood dust} | 11 | 10.9% |
| {Indeno[1,2,3-cd]pyrene,Wood dust} | 11 | 10.9% |

#### Association rules mining

##### Association rules with the largest confidence among WS exposed to a minimum of 2 agents. Minimum level of support = 0.1%, minimum confidence 0.1.

| Antecedent | Consequent | % Confidence | % Support | N WS |
| --- | --- | --- | --- | --- |
| {Indeno[1,2,3-cd]pyrene} | {Benzo[ghi]perylene} | 100.0% | 14.9% | 15 |
| {Benzo[ghi]perylene} | {Indeno[1,2,3-cd]pyrene} | 100.0% | 14.9% | 15 |
| {Indeno[1,2,3-cd]pyrene} | {Benzo[def]chrysene} | 100.0% | 14.9% | 15 |
| {Benzo[def]chrysene} | {Indeno[1,2,3-cd]pyrene} | 100.0% | 14.9% | 15 |
| {Indeno[1,2,3-cd]pyrene} | {Benzo(k)fluoranthene} | 100.0% | 14.9% | 15 |
| {Benzo(k)fluoranthene} | {Indeno[1,2,3-cd]pyrene} | 100.0% | 14.9% | 15 |
| {Indeno[1,2,3-cd]pyrene} | {Benzo(e)acephenanthrylene} | 100.0% | 14.9% | 15 |
| {Benzo(e)acephenanthrylene} | {Indeno[1,2,3-cd]pyrene} | 100.0% | 14.9% | 15 |
| {Indeno[1,2,3-cd]pyrene} | {Benz[a]anthracene} | 100.0% | 14.9% | 15 |
| {Benz[a]anthracene} | {Indeno[1,2,3-cd]pyrene} | 100.0% | 14.9% | 15 |
| {Benzo[ghi]perylene} | {Benzo[def]chrysene} | 100.0% | 14.9% | 15 |
| {Benzo[def]chrysene} | {Benzo[ghi]perylene} | 100.0% | 14.9% | 15 |
| {Benzo[ghi]perylene} | {Benzo(k)fluoranthene} | 100.0% | 14.9% | 15 |
| {Benzo(k)fluoranthene} | {Benzo[ghi]perylene} | 100.0% | 14.9% | 15 |
| {Benzo[ghi]perylene} | {Benzo(e)acephenanthrylene} | 100.0% | 14.9% | 15 |
| {Benzo(e)acephenanthrylene} | {Benzo[ghi]perylene} | 100.0% | 14.9% | 15 |
| {Benzo[ghi]perylene} | {Benz[a]anthracene} | 100.0% | 14.9% | 15 |
| {Benz[a]anthracene} | {Benzo[ghi]perylene} | 100.0% | 14.9% | 15 |
| {Benzo[def]chrysene} | {Benzo(k)fluoranthene} | 100.0% | 14.9% | 15 |
| {Benzo(k)fluoranthene} | {Benzo[def]chrysene} | 100.0% | 14.9% | 15 |
| {Benzo[def]chrysene} | {Benzo(e)acephenanthrylene} | 100.0% | 14.9% | 15 |
| {Benzo(e)acephenanthrylene} | {Benzo[def]chrysene} | 100.0% | 14.9% | 15 |
| {Benzo[def]chrysene} | {Benz[a]anthracene} | 100.0% | 14.9% | 15 |
| {Benz[a]anthracene} | {Benzo[def]chrysene} | 100.0% | 14.9% | 15 |
| {Benzo(k)fluoranthene} | {Benzo(e)acephenanthrylene} | 100.0% | 14.9% | 15 |
| {Benzo(e)acephenanthrylene} | {Benzo(k)fluoranthene} | 100.0% | 14.9% | 15 |
| {Benzo(k)fluoranthene} | {Benz[a]anthracene} | 100.0% | 14.9% | 15 |
| {Benz[a]anthracene} | {Benzo(k)fluoranthene} | 100.0% | 14.9% | 15 |
| {Benzo(e)acephenanthrylene} | {Benz[a]anthracene} | 100.0% | 14.9% | 15 |
| {Benz[a]anthracene} | {Benzo(e)acephenanthrylene} | 100.0% | 14.9% | 15 |
| {Benzo[ghi]perylene,Indeno[1,2,3-cd]pyrene} | {Benzo[def]chrysene} | 100.0% | 14.9% | 15 |
| {Benzo[def]chrysene,Indeno[1,2,3-cd]pyrene} | {Benzo[ghi]perylene} | 100.0% | 14.9% | 15 |
| {Benzo[def]chrysene,Benzo[ghi]perylene} | {Indeno[1,2,3-cd]pyrene} | 100.0% | 14.9% | 15 |
| {Benzo[ghi]perylene,Indeno[1,2,3-cd]pyrene} | {Benzo(k)fluoranthene} | 100.0% | 14.9% | 15 |
| {Benzo(k)fluoranthene,Indeno[1,2,3-cd]pyrene} | {Benzo[ghi]perylene} | 100.0% | 14.9% | 15 |
| {Benzo(k)fluoranthene,Benzo[ghi]perylene} | {Indeno[1,2,3-cd]pyrene} | 100.0% | 14.9% | 15 |
| {Benzo[ghi]perylene,Indeno[1,2,3-cd]pyrene} | {Benzo(e)acephenanthrylene} | 100.0% | 14.9% | 15 |
| {Benzo(e)acephenanthrylene,Indeno[1,2,3-cd]pyrene} | {Benzo[ghi]perylene} | 100.0% | 14.9% | 15 |
| {Benzo(e)acephenanthrylene,Benzo[ghi]perylene} | {Indeno[1,2,3-cd]pyrene} | 100.0% | 14.9% | 15 |
| {Benzo[ghi]perylene,Indeno[1,2,3-cd]pyrene} | {Benz[a]anthracene} | 100.0% | 14.9% | 15 |
| {Benz[a]anthracene,Indeno[1,2,3-cd]pyrene} | {Benzo[ghi]perylene} | 100.0% | 14.9% | 15 |
| {Benz[a]anthracene,Benzo[ghi]perylene} | {Indeno[1,2,3-cd]pyrene} | 100.0% | 14.9% | 15 |
| {Indeno[1,2,3-cd]pyrene,Wood dust} | {Benzo[ghi]perylene} | 100.0% | 10.9% | 11 |
| {Benzo[ghi]perylene,Wood dust} | {Indeno[1,2,3-cd]pyrene} | 100.0% | 10.9% | 11 |
| {Benzo[def]chrysene,Indeno[1,2,3-cd]pyrene} | {Benzo(k)fluoranthene} | 100.0% | 14.9% | 15 |
| {Benzo(k)fluoranthene,Indeno[1,2,3-cd]pyrene} | {Benzo[def]chrysene} | 100.0% | 14.9% | 15 |
| {Benzo(k)fluoranthene,Benzo[def]chrysene} | {Indeno[1,2,3-cd]pyrene} | 100.0% | 14.9% | 15 |
| {Benzo[def]chrysene,Indeno[1,2,3-cd]pyrene} | {Benzo(e)acephenanthrylene} | 100.0% | 14.9% | 15 |
| {Benzo(e)acephenanthrylene,Indeno[1,2,3-cd]pyrene} | {Benzo[def]chrysene} | 100.0% | 14.9% | 15 |
| {Benzo(e)acephenanthrylene,Benzo[def]chrysene} | {Indeno[1,2,3-cd]pyrene} | 100.0% | 14.9% | 15 |
| {Benzo[def]chrysene,Indeno[1,2,3-cd]pyrene} | {Benz[a]anthracene} | 100.0% | 14.9% | 15 |
| {Benz[a]anthracene,Indeno[1,2,3-cd]pyrene} | {Benzo[def]chrysene} | 100.0% | 14.9% | 15 |
| {Benz[a]anthracene,Benzo[def]chrysene} | {Indeno[1,2,3-cd]pyrene} | 100.0% | 14.9% | 15 |
| {Indeno[1,2,3-cd]pyrene,Wood dust} | {Benzo[def]chrysene} | 100.0% | 10.9% | 11 |
| {Benzo[def]chrysene,Wood dust} | {Indeno[1,2,3-cd]pyrene} | 100.0% | 10.9% | 11 |
| {Benzo(k)fluoranthene,Indeno[1,2,3-cd]pyrene} | {Benzo(e)acephenanthrylene} | 100.0% | 14.9% | 15 |
| {Benzo(e)acephenanthrylene,Indeno[1,2,3-cd]pyrene} | {Benzo(k)fluoranthene} | 100.0% | 14.9% | 15 |
| {Benzo(e)acephenanthrylene,Benzo(k)fluoranthene} | {Indeno[1,2,3-cd]pyrene} | 100.0% | 14.9% | 15 |
| {Benzo(k)fluoranthene,Indeno[1,2,3-cd]pyrene} | {Benz[a]anthracene} | 100.0% | 14.9% | 15 |
| {Benz[a]anthracene,Indeno[1,2,3-cd]pyrene} | {Benzo(k)fluoranthene} | 100.0% | 14.9% | 15 |
| {Benz[a]anthracene,Benzo(k)fluoranthene} | {Indeno[1,2,3-cd]pyrene} | 100.0% | 14.9% | 15 |
| {Indeno[1,2,3-cd]pyrene,Wood dust} | {Benzo(k)fluoranthene} | 100.0% | 10.9% | 11 |
| {Benzo(k)fluoranthene,Wood dust} | {Indeno[1,2,3-cd]pyrene} | 100.0% | 10.9% | 11 |
| {Benzo(e)acephenanthrylene,Indeno[1,2,3-cd]pyrene} | {Benz[a]anthracene} | 100.0% | 14.9% | 15 |
| {Benz[a]anthracene,Indeno[1,2,3-cd]pyrene} | {Benzo(e)acephenanthrylene} | 100.0% | 14.9% | 15 |
| {Benz[a]anthracene,Benzo(e)acephenanthrylene} | {Indeno[1,2,3-cd]pyrene} | 100.0% | 14.9% | 15 |
| {Indeno[1,2,3-cd]pyrene,Wood dust} | {Benzo(e)acephenanthrylene} | 100.0% | 10.9% | 11 |
| {Benzo(e)acephenanthrylene,Wood dust} | {Indeno[1,2,3-cd]pyrene} | 100.0% | 10.9% | 11 |
| {Indeno[1,2,3-cd]pyrene,Wood dust} | {Benz[a]anthracene} | 100.0% | 10.9% | 11 |
| {Benz[a]anthracene,Wood dust} | {Indeno[1,2,3-cd]pyrene} | 100.0% | 10.9% | 11 |
| {Benzo[def]chrysene,Benzo[ghi]perylene} | {Benzo(k)fluoranthene} | 100.0% | 14.9% | 15 |
| {Benzo(k)fluoranthene,Benzo[ghi]perylene} | {Benzo[def]chrysene} | 100.0% | 14.9% | 15 |
| {Benzo(k)fluoranthene,Benzo[def]chrysene} | {Benzo[ghi]perylene} | 100.0% | 14.9% | 15 |
| {Benzo[def]chrysene,Benzo[ghi]perylene} | {Benzo(e)acephenanthrylene} | 100.0% | 14.9% | 15 |
| {Benzo(e)acephenanthrylene,Benzo[ghi]perylene} | {Benzo[def]chrysene} | 100.0% | 14.9% | 15 |
| {Benzo(e)acephenanthrylene,Benzo[def]chrysene} | {Benzo[ghi]perylene} | 100.0% | 14.9% | 15 |
| {Benzo[def]chrysene,Benzo[ghi]perylene} | {Benz[a]anthracene} | 100.0% | 14.9% | 15 |
| {Benz[a]anthracene,Benzo[ghi]perylene} | {Benzo[def]chrysene} | 100.0% | 14.9% | 15 |
| {Benz[a]anthracene,Benzo[def]chrysene} | {Benzo[ghi]perylene} | 100.0% | 14.9% | 15 |
| {Benzo[ghi]perylene,Wood dust} | {Benzo[def]chrysene} | 100.0% | 10.9% | 11 |
| {Benzo[def]chrysene,Wood dust} | {Benzo[ghi]perylene} | 100.0% | 10.9% | 11 |
| {Benzo(k)fluoranthene,Benzo[ghi]perylene} | {Benzo(e)acephenanthrylene} | 100.0% | 14.9% | 15 |
| {Benzo(e)acephenanthrylene,Benzo[ghi]perylene} | {Benzo(k)fluoranthene} | 100.0% | 14.9% | 15 |
| {Benzo(e)acephenanthrylene,Benzo(k)fluoranthene} | {Benzo[ghi]perylene} | 100.0% | 14.9% | 15 |
| {Benzo(k)fluoranthene,Benzo[ghi]perylene} | {Benz[a]anthracene} | 100.0% | 14.9% | 15 |
| {Benz[a]anthracene,Benzo[ghi]perylene} | {Benzo(k)fluoranthene} | 100.0% | 14.9% | 15 |
| {Benz[a]anthracene,Benzo(k)fluoranthene} | {Benzo[ghi]perylene} | 100.0% | 14.9% | 15 |
| {Benzo[ghi]perylene,Wood dust} | {Benzo(k)fluoranthene} | 100.0% | 10.9% | 11 |
| {Benzo(k)fluoranthene,Wood dust} | {Benzo[ghi]perylene} | 100.0% | 10.9% | 11 |
| {Benzo(e)acephenanthrylene,Benzo[ghi]perylene} | {Benz[a]anthracene} | 100.0% | 14.9% | 15 |
| {Benz[a]anthracene,Benzo[ghi]perylene} | {Benzo(e)acephenanthrylene} | 100.0% | 14.9% | 15 |
| {Benz[a]anthracene,Benzo(e)acephenanthrylene} | {Benzo[ghi]perylene} | 100.0% | 14.9% | 15 |
| {Benzo[ghi]perylene,Wood dust} | {Benzo(e)acephenanthrylene} | 100.0% | 10.9% | 11 |
| {Benzo(e)acephenanthrylene,Wood dust} | {Benzo[ghi]perylene} | 100.0% | 10.9% | 11 |
| {Benzo[ghi]perylene,Wood dust} | {Benz[a]anthracene} | 100.0% | 10.9% | 11 |
| {Benz[a]anthracene,Wood dust} | {Benzo[ghi]perylene} | 100.0% | 10.9% | 11 |
| {Benzo(k)fluoranthene,Benzo[def]chrysene} | {Benzo(e)acephenanthrylene} | 100.0% | 14.9% | 15 |
| {Benzo(e)acephenanthrylene,Benzo[def]chrysene} | {Benzo(k)fluoranthene} | 100.0% | 14.9% | 15 |
| {Benzo(e)acephenanthrylene,Benzo(k)fluoranthene} | {Benzo[def]chrysene} | 100.0% | 14.9% | 15 |
| {Benzo(k)fluoranthene,Benzo[def]chrysene} | {Benz[a]anthracene} | 100.0% | 14.9% | 15 |
| {Benz[a]anthracene,Benzo[def]chrysene} | {Benzo(k)fluoranthene} | 100.0% | 14.9% | 15 |
| {Benz[a]anthracene,Benzo(k)fluoranthene} | {Benzo[def]chrysene} | 100.0% | 14.9% | 15 |
| {Benzo[def]chrysene,Wood dust} | {Benzo(k)fluoranthene} | 100.0% | 10.9% | 11 |
| {Benzo(k)fluoranthene,Wood dust} | {Benzo[def]chrysene} | 100.0% | 10.9% | 11 |
| {Benzo(e)acephenanthrylene,Benzo[def]chrysene} | {Benz[a]anthracene} | 100.0% | 14.9% | 15 |
| {Benz[a]anthracene,Benzo[def]chrysene} | {Benzo(e)acephenanthrylene} | 100.0% | 14.9% | 15 |
| {Benz[a]anthracene,Benzo(e)acephenanthrylene} | {Benzo[def]chrysene} | 100.0% | 14.9% | 15 |
| {Benzo[def]chrysene,Wood dust} | {Benzo(e)acephenanthrylene} | 100.0% | 10.9% | 11 |
| {Benzo(e)acephenanthrylene,Wood dust} | {Benzo[def]chrysene} | 100.0% | 10.9% | 11 |
| {Benzo[def]chrysene,Wood dust} | {Benz[a]anthracene} | 100.0% | 10.9% | 11 |
| {Benz[a]anthracene,Wood dust} | {Benzo[def]chrysene} | 100.0% | 10.9% | 11 |
| {Benzo(e)acephenanthrylene,Benzo(k)fluoranthene} | {Benz[a]anthracene} | 100.0% | 14.9% | 15 |
| {Benz[a]anthracene,Benzo(k)fluoranthene} | {Benzo(e)acephenanthrylene} | 100.0% | 14.9% | 15 |
| {Benz[a]anthracene,Benzo(e)acephenanthrylene} | {Benzo(k)fluoranthene} | 100.0% | 14.9% | 15 |
| {Benzo(k)fluoranthene,Wood dust} | {Benzo(e)acephenanthrylene} | 100.0% | 10.9% | 11 |
| {Benzo(e)acephenanthrylene,Wood dust} | {Benzo(k)fluoranthene} | 100.0% | 10.9% | 11 |
| {Benzo(k)fluoranthene,Wood dust} | {Benz[a]anthracene} | 100.0% | 10.9% | 11 |
| {Benz[a]anthracene,Wood dust} | {Benzo(k)fluoranthene} | 100.0% | 10.9% | 11 |
| {Benzo(e)acephenanthrylene,Wood dust} | {Benz[a]anthracene} | 100.0% | 10.9% | 11 |
| {Benz[a]anthracene,Wood dust} | {Benzo(e)acephenanthrylene} | 100.0% | 10.9% | 11 |
| {Benzo[def]chrysene,Benzo[ghi]perylene,Indeno[1,2,3-cd]pyrene} | {Benzo(k)fluoranthene} | 100.0% | 14.9% | 15 |
| {Benzo(k)fluoranthene,Benzo[ghi]perylene,Indeno[1,2,3-cd]pyrene} | {Benzo[def]chrysene} | 100.0% | 14.9% | 15 |
| {Benzo(k)fluoranthene,Benzo[def]chrysene,Indeno[1,2,3-cd]pyrene} | {Benzo[ghi]perylene} | 100.0% | 14.9% | 15 |
| {Benzo(k)fluoranthene,Benzo[def]chrysene,Benzo[ghi]perylene} | {Indeno[1,2,3-cd]pyrene} | 100.0% | 14.9% | 15 |
| {Benzo[def]chrysene,Benzo[ghi]perylene,Indeno[1,2,3-cd]pyrene} | {Benzo(e)acephenanthrylene} | 100.0% | 14.9% | 15 |
| {Benzo(e)acephenanthrylene,Benzo[ghi]perylene,Indeno[1,2,3-cd]pyrene} | {Benzo[def]chrysene} | 100.0% | 14.9% | 15 |
| {Benzo(e)acephenanthrylene,Benzo[def]chrysene,Indeno[1,2,3-cd]pyrene} | {Benzo[ghi]perylene} | 100.0% | 14.9% | 15 |
| {Benzo(e)acephenanthrylene,Benzo[def]chrysene,Benzo[ghi]perylene} | {Indeno[1,2,3-cd]pyrene} | 100.0% | 14.9% | 15 |
| {Benzo[def]chrysene,Benzo[ghi]perylene,Indeno[1,2,3-cd]pyrene} | {Benz[a]anthracene} | 100.0% | 14.9% | 15 |
| {Benz[a]anthracene,Benzo[ghi]perylene,Indeno[1,2,3-cd]pyrene} | {Benzo[def]chrysene} | 100.0% | 14.9% | 15 |
| {Benz[a]anthracene,Benzo[def]chrysene,Indeno[1,2,3-cd]pyrene} | {Benzo[ghi]perylene} | 100.0% | 14.9% | 15 |
| {Benz[a]anthracene,Benzo[def]chrysene,Benzo[ghi]perylene} | {Indeno[1,2,3-cd]pyrene} | 100.0% | 14.9% | 15 |
| {Benzo[ghi]perylene,Indeno[1,2,3-cd]pyrene,Wood dust} | {Benzo[def]chrysene} | 100.0% | 10.9% | 11 |
| {Benzo[def]chrysene,Indeno[1,2,3-cd]pyrene,Wood dust} | {Benzo[ghi]perylene} | 100.0% | 10.9% | 11 |
| {Benzo[def]chrysene,Benzo[ghi]perylene,Wood dust} | {Indeno[1,2,3-cd]pyrene} | 100.0% | 10.9% | 11 |
| {Benzo(k)fluoranthene,Benzo[ghi]perylene,Indeno[1,2,3-cd]pyrene} | {Benzo(e)acephenanthrylene} | 100.0% | 14.9% | 15 |
| {Benzo(e)acephenanthrylene,Benzo[ghi]perylene,Indeno[1,2,3-cd]pyrene} | {Benzo(k)fluoranthene} | 100.0% | 14.9% | 15 |
| {Benzo(e)acephenanthrylene,Benzo(k)fluoranthene,Indeno[1,2,3-cd]pyrene} | {Benzo[ghi]perylene} | 100.0% | 14.9% | 15 |
| {Benzo(e)acephenanthrylene,Benzo(k)fluoranthene,Benzo[ghi]perylene} | {Indeno[1,2,3-cd]pyrene} | 100.0% | 14.9% | 15 |
| {Benzo(k)fluoranthene,Benzo[ghi]perylene,Indeno[1,2,3-cd]pyrene} | {Benz[a]anthracene} | 100.0% | 14.9% | 15 |
| {Benz[a]anthracene,Benzo[ghi]perylene,Indeno[1,2,3-cd]pyrene} | {Benzo(k)fluoranthene} | 100.0% | 14.9% | 15 |
| {Benz[a]anthracene,Benzo(k)fluoranthene,Indeno[1,2,3-cd]pyrene} | {Benzo[ghi]perylene} | 100.0% | 14.9% | 15 |
| {Benz[a]anthracene,Benzo(k)fluoranthene,Benzo[ghi]perylene} | {Indeno[1,2,3-cd]pyrene} | 100.0% | 14.9% | 15 |
| {Benzo[ghi]perylene,Indeno[1,2,3-cd]pyrene,Wood dust} | {Benzo(k)fluoranthene} | 100.0% | 10.9% | 11 |
| {Benzo(k)fluoranthene,Indeno[1,2,3-cd]pyrene,Wood dust} | {Benzo[ghi]perylene} | 100.0% | 10.9% | 11 |
| {Benzo(k)fluoranthene,Benzo[ghi]perylene,Wood dust} | {Indeno[1,2,3-cd]pyrene} | 100.0% | 10.9% | 11 |
| {Benzo(e)acephenanthrylene,Benzo[ghi]perylene,Indeno[1,2,3-cd]pyrene} | {Benz[a]anthracene} | 100.0% | 14.9% | 15 |
| {Benz[a]anthracene,Benzo[ghi]perylene,Indeno[1,2,3-cd]pyrene} | {Benzo(e)acephenanthrylene} | 100.0% | 14.9% | 15 |
| {Benz[a]anthracene,Benzo(e)acephenanthrylene,Indeno[1,2,3-cd]pyrene} | {Benzo[ghi]perylene} | 100.0% | 14.9% | 15 |
| {Benz[a]anthracene,Benzo(e)acephenanthrylene,Benzo[ghi]perylene} | {Indeno[1,2,3-cd]pyrene} | 100.0% | 14.9% | 15 |
| {Benzo[ghi]perylene,Indeno[1,2,3-cd]pyrene,Wood dust} | {Benzo(e)acephenanthrylene} | 100.0% | 10.9% | 11 |
| {Benzo(e)acephenanthrylene,Indeno[1,2,3-cd]pyrene,Wood dust} | {Benzo[ghi]perylene} | 100.0% | 10.9% | 11 |
| {Benzo(e)acephenanthrylene,Benzo[ghi]perylene,Wood dust} | {Indeno[1,2,3-cd]pyrene} | 100.0% | 10.9% | 11 |
| {Benzo[ghi]perylene,Indeno[1,2,3-cd]pyrene,Wood dust} | {Benz[a]anthracene} | 100.0% | 10.9% | 11 |
| {Benz[a]anthracene,Indeno[1,2,3-cd]pyrene,Wood dust} | {Benzo[ghi]perylene} | 100.0% | 10.9% | 11 |
| {Benz[a]anthracene,Benzo[ghi]perylene,Wood dust} | {Indeno[1,2,3-cd]pyrene} | 100.0% | 10.9% | 11 |
| {Benzo(k)fluoranthene,Benzo[def]chrysene,Indeno[1,2,3-cd]pyrene} | {Benzo(e)acephenanthrylene} | 100.0% | 14.9% | 15 |
| {Benzo(e)acephenanthrylene,Benzo[def]chrysene,Indeno[1,2,3-cd]pyrene} | {Benzo(k)fluoranthene} | 100.0% | 14.9% | 15 |
| {Benzo(e)acephenanthrylene,Benzo(k)fluoranthene,Indeno[1,2,3-cd]pyrene} | {Benzo[def]chrysene} | 100.0% | 14.9% | 15 |
| {Benzo(e)acephenanthrylene,Benzo(k)fluoranthene,Benzo[def]chrysene} | {Indeno[1,2,3-cd]pyrene} | 100.0% | 14.9% | 15 |
| {Benzo(k)fluoranthene,Benzo[def]chrysene,Indeno[1,2,3-cd]pyrene} | {Benz[a]anthracene} | 100.0% | 14.9% | 15 |
| {Benz[a]anthracene,Benzo[def]chrysene,Indeno[1,2,3-cd]pyrene} | {Benzo(k)fluoranthene} | 100.0% | 14.9% | 15 |
| {Benz[a]anthracene,Benzo(k)fluoranthene,Indeno[1,2,3-cd]pyrene} | {Benzo[def]chrysene} | 100.0% | 14.9% | 15 |
| {Benz[a]anthracene,Benzo(k)fluoranthene,Benzo[def]chrysene} | {Indeno[1,2,3-cd]pyrene} | 100.0% | 14.9% | 15 |
| {Benzo[def]chrysene,Indeno[1,2,3-cd]pyrene,Wood dust} | {Benzo(k)fluoranthene} | 100.0% | 10.9% | 11 |
| {Benzo(k)fluoranthene,Indeno[1,2,3-cd]pyrene,Wood dust} | {Benzo[def]chrysene} | 100.0% | 10.9% | 11 |
| {Benzo(k)fluoranthene,Benzo[def]chrysene,Wood dust} | {Indeno[1,2,3-cd]pyrene} | 100.0% | 10.9% | 11 |
| {Benzo(e)acephenanthrylene,Benzo[def]chrysene,Indeno[1,2,3-cd]pyrene} | {Benz[a]anthracene} | 100.0% | 14.9% | 15 |
| {Benz[a]anthracene,Benzo[def]chrysene,Indeno[1,2,3-cd]pyrene} | {Benzo(e)acephenanthrylene} | 100.0% | 14.9% | 15 |
| {Benz[a]anthracene,Benzo(e)acephenanthrylene,Indeno[1,2,3-cd]pyrene} | {Benzo[def]chrysene} | 100.0% | 14.9% | 15 |
| {Benz[a]anthracene,Benzo(e)acephenanthrylene,Benzo[def]chrysene} | {Indeno[1,2,3-cd]pyrene} | 100.0% | 14.9% | 15 |
| {Benzo[def]chrysene,Indeno[1,2,3-cd]pyrene,Wood dust} | {Benzo(e)acephenanthrylene} | 100.0% | 10.9% | 11 |
| {Benzo(e)acephenanthrylene,Indeno[1,2,3-cd]pyrene,Wood dust} | {Benzo[def]chrysene} | 100.0% | 10.9% | 11 |
| {Benzo(e)acephenanthrylene,Benzo[def]chrysene,Wood dust} | {Indeno[1,2,3-cd]pyrene} | 100.0% | 10.9% | 11 |
| {Benzo[def]chrysene,Indeno[1,2,3-cd]pyrene,Wood dust} | {Benz[a]anthracene} | 100.0% | 10.9% | 11 |
| {Benz[a]anthracene,Indeno[1,2,3-cd]pyrene,Wood dust} | {Benzo[def]chrysene} | 100.0% | 10.9% | 11 |
| {Benz[a]anthracene,Benzo[def]chrysene,Wood dust} | {Indeno[1,2,3-cd]pyrene} | 100.0% | 10.9% | 11 |
| {Benzo(e)acephenanthrylene,Benzo(k)fluoranthene,Indeno[1,2,3-cd]pyrene} | {Benz[a]anthracene} | 100.0% | 14.9% | 15 |
| {Benz[a]anthracene,Benzo(k)fluoranthene,Indeno[1,2,3-cd]pyrene} | {Benzo(e)acephenanthrylene} | 100.0% | 14.9% | 15 |
| {Benz[a]anthracene,Benzo(e)acephenanthrylene,Indeno[1,2,3-cd]pyrene} | {Benzo(k)fluoranthene} | 100.0% | 14.9% | 15 |
| {Benz[a]anthracene,Benzo(e)acephenanthrylene,Benzo(k)fluoranthene} | {Indeno[1,2,3-cd]pyrene} | 100.0% | 14.9% | 15 |
| {Benzo(k)fluoranthene,Indeno[1,2,3-cd]pyrene,Wood dust} | {Benzo(e)acephenanthrylene} | 100.0% | 10.9% | 11 |
| {Benzo(e)acephenanthrylene,Indeno[1,2,3-cd]pyrene,Wood dust} | {Benzo(k)fluoranthene} | 100.0% | 10.9% | 11 |
| {Benzo(e)acephenanthrylene,Benzo(k)fluoranthene,Wood dust} | {Indeno[1,2,3-cd]pyrene} | 100.0% | 10.9% | 11 |
| {Benzo(k)fluoranthene,Indeno[1,2,3-cd]pyrene,Wood dust} | {Benz[a]anthracene} | 100.0% | 10.9% | 11 |
| {Benz[a]anthracene,Indeno[1,2,3-cd]pyrene,Wood dust} | {Benzo(k)fluoranthene} | 100.0% | 10.9% | 11 |
| {Benz[a]anthracene,Benzo(k)fluoranthene,Wood dust} | {Indeno[1,2,3-cd]pyrene} | 100.0% | 10.9% | 11 |
| {Benzo(e)acephenanthrylene,Indeno[1,2,3-cd]pyrene,Wood dust} | {Benz[a]anthracene} | 100.0% | 10.9% | 11 |
| {Benz[a]anthracene,Indeno[1,2,3-cd]pyrene,Wood dust} | {Benzo(e)acephenanthrylene} | 100.0% | 10.9% | 11 |
| {Benz[a]anthracene,Benzo(e)acephenanthrylene,Wood dust} | {Indeno[1,2,3-cd]pyrene} | 100.0% | 10.9% | 11 |
| {Benzo(k)fluoranthene,Benzo[def]chrysene,Benzo[ghi]perylene} | {Benzo(e)acephenanthrylene} | 100.0% | 14.9% | 15 |
| {Benzo(e)acephenanthrylene,Benzo[def]chrysene,Benzo[ghi]perylene} | {Benzo(k)fluoranthene} | 100.0% | 14.9% | 15 |
| {Benzo(e)acephenanthrylene,Benzo(k)fluoranthene,Benzo[ghi]perylene} | {Benzo[def]chrysene} | 100.0% | 14.9% | 15 |
| {Benzo(e)acephenanthrylene,Benzo(k)fluoranthene,Benzo[def]chrysene} | {Benzo[ghi]perylene} | 100.0% | 14.9% | 15 |
| {Benzo(k)fluoranthene,Benzo[def]chrysene,Benzo[ghi]perylene} | {Benz[a]anthracene} | 100.0% | 14.9% | 15 |
| {Benz[a]anthracene,Benzo[def]chrysene,Benzo[ghi]perylene} | {Benzo(k)fluoranthene} | 100.0% | 14.9% | 15 |
| {Benz[a]anthracene,Benzo(k)fluoranthene,Benzo[ghi]perylene} | {Benzo[def]chrysene} | 100.0% | 14.9% | 15 |
| {Benz[a]anthracene,Benzo(k)fluoranthene,Benzo[def]chrysene} | {Benzo[ghi]perylene} | 100.0% | 14.9% | 15 |
| {Benzo[def]chrysene,Benzo[ghi]perylene,Wood dust} | {Benzo(k)fluoranthene} | 100.0% | 10.9% | 11 |
| {Benzo(k)fluoranthene,Benzo[ghi]perylene,Wood dust} | {Benzo[def]chrysene} | 100.0% | 10.9% | 11 |
| {Benzo(k)fluoranthene,Benzo[def]chrysene,Wood dust} | {Benzo[ghi]perylene} | 100.0% | 10.9% | 11 |
| {Benzo(e)acephenanthrylene,Benzo[def]chrysene,Benzo[ghi]perylene} | {Benz[a]anthracene} | 100.0% | 14.9% | 15 |
| {Benz[a]anthracene,Benzo[def]chrysene,Benzo[ghi]perylene} | {Benzo(e)acephenanthrylene} | 100.0% | 14.9% | 15 |
| {Benz[a]anthracene,Benzo(e)acephenanthrylene,Benzo[ghi]perylene} | {Benzo[def]chrysene} | 100.0% | 14.9% | 15 |
| {Benz[a]anthracene,Benzo(e)acephenanthrylene,Benzo[def]chrysene} | {Benzo[ghi]perylene} | 100.0% | 14.9% | 15 |
| {Benzo[def]chrysene,Benzo[ghi]perylene,Wood dust} | {Benzo(e)acephenanthrylene} | 100.0% | 10.9% | 11 |
| {Benzo(e)acephenanthrylene,Benzo[ghi]perylene,Wood dust} | {Benzo[def]chrysene} | 100.0% | 10.9% | 11 |
| {Benzo(e)acephenanthrylene,Benzo[def]chrysene,Wood dust} | {Benzo[ghi]perylene} | 100.0% | 10.9% | 11 |
| {Benzo[def]chrysene,Benzo[ghi]perylene,Wood dust} | {Benz[a]anthracene} | 100.0% | 10.9% | 11 |
| {Benz[a]anthracene,Benzo[ghi]perylene,Wood dust} | {Benzo[def]chrysene} | 100.0% | 10.9% | 11 |
| {Benz[a]anthracene,Benzo[def]chrysene,Wood dust} | {Benzo[ghi]perylene} | 100.0% | 10.9% | 11 |
| {Benzo(e)acephenanthrylene,Benzo(k)fluoranthene,Benzo[ghi]perylene} | {Benz[a]anthracene} | 100.0% | 14.9% | 15 |
| {Benz[a]anthracene,Benzo(k)fluoranthene,Benzo[ghi]perylene} | {Benzo(e)acephenanthrylene} | 100.0% | 14.9% | 15 |
| {Benz[a]anthracene,Benzo(e)acephenanthrylene,Benzo[ghi]perylene} | {Benzo(k)fluoranthene} | 100.0% | 14.9% | 15 |
| {Benz[a]anthracene,Benzo(e)acephenanthrylene,Benzo(k)fluoranthene} | {Benzo[ghi]perylene} | 100.0% | 14.9% | 15 |
| {Benzo(k)fluoranthene,Benzo[ghi]perylene,Wood dust} | {Benzo(e)acephenanthrylene} | 100.0% | 10.9% | 11 |
| {Benzo(e)acephenanthrylene,Benzo[ghi]perylene,Wood dust} | {Benzo(k)fluoranthene} | 100.0% | 10.9% | 11 |
| {Benzo(e)acephenanthrylene,Benzo(k)fluoranthene,Wood dust} | {Benzo[ghi]perylene} | 100.0% | 10.9% | 11 |
| {Benzo(k)fluoranthene,Benzo[ghi]perylene,Wood dust} | {Benz[a]anthracene} | 100.0% | 10.9% | 11 |
| {Benz[a]anthracene,Benzo[ghi]perylene,Wood dust} | {Benzo(k)fluoranthene} | 100.0% | 10.9% | 11 |
| {Benz[a]anthracene,Benzo(k)fluoranthene,Wood dust} | {Benzo[ghi]perylene} | 100.0% | 10.9% | 11 |
| {Benzo(e)acephenanthrylene,Benzo[ghi]perylene,Wood dust} | {Benz[a]anthracene} | 100.0% | 10.9% | 11 |
| {Benz[a]anthracene,Benzo[ghi]perylene,Wood dust} | {Benzo(e)acephenanthrylene} | 100.0% | 10.9% | 11 |
| {Benz[a]anthracene,Benzo(e)acephenanthrylene,Wood dust} | {Benzo[ghi]perylene} | 100.0% | 10.9% | 11 |
| {Benzo(e)acephenanthrylene,Benzo(k)fluoranthene,Benzo[def]chrysene} | {Benz[a]anthracene} | 100.0% | 14.9% | 15 |
| {Benz[a]anthracene,Benzo(k)fluoranthene,Benzo[def]chrysene} | {Benzo(e)acephenanthrylene} | 100.0% | 14.9% | 15 |
| {Benz[a]anthracene,Benzo(e)acephenanthrylene,Benzo[def]chrysene} | {Benzo(k)fluoranthene} | 100.0% | 14.9% | 15 |
| {Benz[a]anthracene,Benzo(e)acephenanthrylene,Benzo(k)fluoranthene} | {Benzo[def]chrysene} | 100.0% | 14.9% | 15 |
| {Benzo(k)fluoranthene,Benzo[def]chrysene,Wood dust} | {Benzo(e)acephenanthrylene} | 100.0% | 10.9% | 11 |
| {Benzo(e)acephenanthrylene,Benzo[def]chrysene,Wood dust} | {Benzo(k)fluoranthene} | 100.0% | 10.9% | 11 |
| {Benzo(e)acephenanthrylene,Benzo(k)fluoranthene,Wood dust} | {Benzo[def]chrysene} | 100.0% | 10.9% | 11 |
| {Benzo(k)fluoranthene,Benzo[def]chrysene,Wood dust} | {Benz[a]anthracene} | 100.0% | 10.9% | 11 |
| {Benz[a]anthracene,Benzo[def]chrysene,Wood dust} | {Benzo(k)fluoranthene} | 100.0% | 10.9% | 11 |
| {Benz[a]anthracene,Benzo(k)fluoranthene,Wood dust} | {Benzo[def]chrysene} | 100.0% | 10.9% | 11 |
| {Benzo(e)acephenanthrylene,Benzo[def]chrysene,Wood dust} | {Benz[a]anthracene} | 100.0% | 10.9% | 11 |
| {Benz[a]anthracene,Benzo[def]chrysene,Wood dust} | {Benzo(e)acephenanthrylene} | 100.0% | 10.9% | 11 |
| {Benz[a]anthracene,Benzo(e)acephenanthrylene,Wood dust} | {Benzo[def]chrysene} | 100.0% | 10.9% | 11 |
| {Benzo(e)acephenanthrylene,Benzo(k)fluoranthene,Wood dust} | {Benz[a]anthracene} | 100.0% | 10.9% | 11 |
| {Benz[a]anthracene,Benzo(k)fluoranthene,Wood dust} | {Benzo(e)acephenanthrylene} | 100.0% | 10.9% | 11 |
| {Benz[a]anthracene,Benzo(e)acephenanthrylene,Wood dust} | {Benzo(k)fluoranthene} | 100.0% | 10.9% | 11 |
| {Benzo(k)fluoranthene,Benzo[def]chrysene,Benzo[ghi]perylene,Indeno[1,2,3-cd]pyrene} | {Benzo(e)acephenanthrylene} | 100.0% | 14.9% | 15 |
| {Benzo(e)acephenanthrylene,Benzo[def]chrysene,Benzo[ghi]perylene,Indeno[1,2,3-cd]pyrene} | {Benzo(k)fluoranthene} | 100.0% | 14.9% | 15 |
| {Benzo(e)acephenanthrylene,Benzo(k)fluoranthene,Benzo[ghi]perylene,Indeno[1,2,3-cd]pyrene} | {Benzo[def]chrysene} | 100.0% | 14.9% | 15 |
| {Benzo(e)acephenanthrylene,Benzo(k)fluoranthene,Benzo[def]chrysene,Indeno[1,2,3-cd]pyrene} | {Benzo[ghi]perylene} | 100.0% | 14.9% | 15 |
| {Benzo(e)acephenanthrylene,Benzo(k)fluoranthene,Benzo[def]chrysene,Benzo[ghi]perylene} | {Indeno[1,2,3-cd]pyrene} | 100.0% | 14.9% | 15 |
| {Benzo(k)fluoranthene,Benzo[def]chrysene,Benzo[ghi]perylene,Indeno[1,2,3-cd]pyrene} | {Benz[a]anthracene} | 100.0% | 14.9% | 15 |
| {Benz[a]anthracene,Benzo[def]chrysene,Benzo[ghi]perylene,Indeno[1,2,3-cd]pyrene} | {Benzo(k)fluoranthene} | 100.0% | 14.9% | 15 |
| {Benz[a]anthracene,Benzo(k)fluoranthene,Benzo[ghi]perylene,Indeno[1,2,3-cd]pyrene} | {Benzo[def]chrysene} | 100.0% | 14.9% | 15 |
| {Benz[a]anthracene,Benzo(k)fluoranthene,Benzo[def]chrysene,Indeno[1,2,3-cd]pyrene} | {Benzo[ghi]perylene} | 100.0% | 14.9% | 15 |
| {Benz[a]anthracene,Benzo(k)fluoranthene,Benzo[def]chrysene,Benzo[ghi]perylene} | {Indeno[1,2,3-cd]pyrene} | 100.0% | 14.9% | 15 |
| {Benzo[def]chrysene,Benzo[ghi]perylene,Indeno[1,2,3-cd]pyrene,Wood dust} | {Benzo(k)fluoranthene} | 100.0% | 10.9% | 11 |
| {Benzo(k)fluoranthene,Benzo[ghi]perylene,Indeno[1,2,3-cd]pyrene,Wood dust} | {Benzo[def]chrysene} | 100.0% | 10.9% | 11 |
| {Benzo(k)fluoranthene,Benzo[def]chrysene,Indeno[1,2,3-cd]pyrene,Wood dust} | {Benzo[ghi]perylene} | 100.0% | 10.9% | 11 |
| {Benzo(k)fluoranthene,Benzo[def]chrysene,Benzo[ghi]perylene,Wood dust} | {Indeno[1,2,3-cd]pyrene} | 100.0% | 10.9% | 11 |
| {Benzo(e)acephenanthrylene,Benzo[def]chrysene,Benzo[ghi]perylene,Indeno[1,2,3-cd]pyrene} | {Benz[a]anthracene} | 100.0% | 14.9% | 15 |
| {Benz[a]anthracene,Benzo[def]chrysene,Benzo[ghi]perylene,Indeno[1,2,3-cd]pyrene} | {Benzo(e)acephenanthrylene} | 100.0% | 14.9% | 15 |
| {Benz[a]anthracene,Benzo(e)acephenanthrylene,Benzo[ghi]perylene,Indeno[1,2,3-cd]pyrene} | {Benzo[def]chrysene} | 100.0% | 14.9% | 15 |
| {Benz[a]anthracene,Benzo(e)acephenanthrylene,Benzo[def]chrysene,Indeno[1,2,3-cd]pyrene} | {Benzo[ghi]perylene} | 100.0% | 14.9% | 15 |
| {Benz[a]anthracene,Benzo(e)acephenanthrylene,Benzo[def]chrysene,Benzo[ghi]perylene} | {Indeno[1,2,3-cd]pyrene} | 100.0% | 14.9% | 15 |
| {Benzo[def]chrysene,Benzo[ghi]perylene,Indeno[1,2,3-cd]pyrene,Wood dust} | {Benzo(e)acephenanthrylene} | 100.0% | 10.9% | 11 |
| {Benzo(e)acephenanthrylene,Benzo[ghi]perylene,Indeno[1,2,3-cd]pyrene,Wood dust} | {Benzo[def]chrysene} | 100.0% | 10.9% | 11 |
| {Benzo(e)acephenanthrylene,Benzo[def]chrysene,Indeno[1,2,3-cd]pyrene,Wood dust} | {Benzo[ghi]perylene} | 100.0% | 10.9% | 11 |
| {Benzo(e)acephenanthrylene,Benzo[def]chrysene,Benzo[ghi]perylene,Wood dust} | {Indeno[1,2,3-cd]pyrene} | 100.0% | 10.9% | 11 |
| {Benzo[def]chrysene,Benzo[ghi]perylene,Indeno[1,2,3-cd]pyrene,Wood dust} | {Benz[a]anthracene} | 100.0% | 10.9% | 11 |
| {Benz[a]anthracene,Benzo[ghi]perylene,Indeno[1,2,3-cd]pyrene,Wood dust} | {Benzo[def]chrysene} | 100.0% | 10.9% | 11 |
| {Benz[a]anthracene,Benzo[def]chrysene,Indeno[1,2,3-cd]pyrene,Wood dust} | {Benzo[ghi]perylene} | 100.0% | 10.9% | 11 |
| {Benz[a]anthracene,Benzo[def]chrysene,Benzo[ghi]perylene,Wood dust} | {Indeno[1,2,3-cd]pyrene} | 100.0% | 10.9% | 11 |
| {Benzo(e)acephenanthrylene,Benzo(k)fluoranthene,Benzo[ghi]perylene,Indeno[1,2,3-cd]pyrene} | {Benz[a]anthracene} | 100.0% | 14.9% | 15 |
| {Benz[a]anthracene,Benzo(k)fluoranthene,Benzo[ghi]perylene,Indeno[1,2,3-cd]pyrene} | {Benzo(e)acephenanthrylene} | 100.0% | 14.9% | 15 |
| {Benz[a]anthracene,Benzo(e)acephenanthrylene,Benzo[ghi]perylene,Indeno[1,2,3-cd]pyrene} | {Benzo(k)fluoranthene} | 100.0% | 14.9% | 15 |
| {Benz[a]anthracene,Benzo(e)acephenanthrylene,Benzo(k)fluoranthene,Indeno[1,2,3-cd]pyrene} | {Benzo[ghi]perylene} | 100.0% | 14.9% | 15 |
| {Benz[a]anthracene,Benzo(e)acephenanthrylene,Benzo(k)fluoranthene,Benzo[ghi]perylene} | {Indeno[1,2,3-cd]pyrene} | 100.0% | 14.9% | 15 |
| {Benzo(k)fluoranthene,Benzo[ghi]perylene,Indeno[1,2,3-cd]pyrene,Wood dust} | {Benzo(e)acephenanthrylene} | 100.0% | 10.9% | 11 |
| {Benzo(e)acephenanthrylene,Benzo[ghi]perylene,Indeno[1,2,3-cd]pyrene,Wood dust} | {Benzo(k)fluoranthene} | 100.0% | 10.9% | 11 |
| {Benzo(e)acephenanthrylene,Benzo(k)fluoranthene,Indeno[1,2,3-cd]pyrene,Wood dust} | {Benzo[ghi]perylene} | 100.0% | 10.9% | 11 |
| {Benzo(e)acephenanthrylene,Benzo(k)fluoranthene,Benzo[ghi]perylene,Wood dust} | {Indeno[1,2,3-cd]pyrene} | 100.0% | 10.9% | 11 |
| {Benzo(k)fluoranthene,Benzo[ghi]perylene,Indeno[1,2,3-cd]pyrene,Wood dust} | {Benz[a]anthracene} | 100.0% | 10.9% | 11 |
| {Benz[a]anthracene,Benzo[ghi]perylene,Indeno[1,2,3-cd]pyrene,Wood dust} | {Benzo(k)fluoranthene} | 100.0% | 10.9% | 11 |
| {Benz[a]anthracene,Benzo(k)fluoranthene,Indeno[1,2,3-cd]pyrene,Wood dust} | {Benzo[ghi]perylene} | 100.0% | 10.9% | 11 |
| {Benz[a]anthracene,Benzo(k)fluoranthene,Benzo[ghi]perylene,Wood dust} | {Indeno[1,2,3-cd]pyrene} | 100.0% | 10.9% | 11 |
| {Benzo(e)acephenanthrylene,Benzo[ghi]perylene,Indeno[1,2,3-cd]pyrene,Wood dust} | {Benz[a]anthracene} | 100.0% | 10.9% | 11 |
| {Benz[a]anthracene,Benzo[ghi]perylene,Indeno[1,2,3-cd]pyrene,Wood dust} | {Benzo(e)acephenanthrylene} | 100.0% | 10.9% | 11 |
| {Benz[a]anthracene,Benzo(e)acephenanthrylene,Indeno[1,2,3-cd]pyrene,Wood dust} | {Benzo[ghi]perylene} | 100.0% | 10.9% | 11 |
| {Benz[a]anthracene,Benzo(e)acephenanthrylene,Benzo[ghi]perylene,Wood dust} | {Indeno[1,2,3-cd]pyrene} | 100.0% | 10.9% | 11 |
| {Benzo(e)acephenanthrylene,Benzo(k)fluoranthene,Benzo[def]chrysene,Indeno[1,2,3-cd]pyrene} | {Benz[a]anthracene} | 100.0% | 14.9% | 15 |
| {Benz[a]anthracene,Benzo(k)fluoranthene,Benzo[def]chrysene,Indeno[1,2,3-cd]pyrene} | {Benzo(e)acephenanthrylene} | 100.0% | 14.9% | 15 |
| {Benz[a]anthracene,Benzo(e)acephenanthrylene,Benzo[def]chrysene,Indeno[1,2,3-cd]pyrene} | {Benzo(k)fluoranthene} | 100.0% | 14.9% | 15 |
| {Benz[a]anthracene,Benzo(e)acephenanthrylene,Benzo(k)fluoranthene,Indeno[1,2,3-cd]pyrene} | {Benzo[def]chrysene} | 100.0% | 14.9% | 15 |
| {Benz[a]anthracene,Benzo(e)acephenanthrylene,Benzo(k)fluoranthene,Benzo[def]chrysene} | {Indeno[1,2,3-cd]pyrene} | 100.0% | 14.9% | 15 |
| {Benzo(k)fluoranthene,Benzo[def]chrysene,Indeno[1,2,3-cd]pyrene,Wood dust} | {Benzo(e)acephenanthrylene} | 100.0% | 10.9% | 11 |
| {Benzo(e)acephenanthrylene,Benzo[def]chrysene,Indeno[1,2,3-cd]pyrene,Wood dust} | {Benzo(k)fluoranthene} | 100.0% | 10.9% | 11 |
| {Benzo(e)acephenanthrylene,Benzo(k)fluoranthene,Indeno[1,2,3-cd]pyrene,Wood dust} | {Benzo[def]chrysene} | 100.0% | 10.9% | 11 |
| {Benzo(e)acephenanthrylene,Benzo(k)fluoranthene,Benzo[def]chrysene,Wood dust} | {Indeno[1,2,3-cd]pyrene} | 100.0% | 10.9% | 11 |
| {Benzo(k)fluoranthene,Benzo[def]chrysene,Indeno[1,2,3-cd]pyrene,Wood dust} | {Benz[a]anthracene} | 100.0% | 10.9% | 11 |
| {Benz[a]anthracene,Benzo[def]chrysene,Indeno[1,2,3-cd]pyrene,Wood dust} | {Benzo(k)fluoranthene} | 100.0% | 10.9% | 11 |
| {Benz[a]anthracene,Benzo(k)fluoranthene,Indeno[1,2,3-cd]pyrene,Wood dust} | {Benzo[def]chrysene} | 100.0% | 10.9% | 11 |
| {Benz[a]anthracene,Benzo(k)fluoranthene,Benzo[def]chrysene,Wood dust} | {Indeno[1,2,3-cd]pyrene} | 100.0% | 10.9% | 11 |
| {Benzo(e)acephenanthrylene,Benzo[def]chrysene,Indeno[1,2,3-cd]pyrene,Wood dust} | {Benz[a]anthracene} | 100.0% | 10.9% | 11 |
| {Benz[a]anthracene,Benzo[def]chrysene,Indeno[1,2,3-cd]pyrene,Wood dust} | {Benzo(e)acephenanthrylene} | 100.0% | 10.9% | 11 |
| {Benz[a]anthracene,Benzo(e)acephenanthrylene,Indeno[1,2,3-cd]pyrene,Wood dust} | {Benzo[def]chrysene} | 100.0% | 10.9% | 11 |
| {Benz[a]anthracene,Benzo(e)acephenanthrylene,Benzo[def]chrysene,Wood dust} | {Indeno[1,2,3-cd]pyrene} | 100.0% | 10.9% | 11 |
| {Benzo(e)acephenanthrylene,Benzo(k)fluoranthene,Indeno[1,2,3-cd]pyrene,Wood dust} | {Benz[a]anthracene} | 100.0% | 10.9% | 11 |
| {Benz[a]anthracene,Benzo(k)fluoranthene,Indeno[1,2,3-cd]pyrene,Wood dust} | {Benzo(e)acephenanthrylene} | 100.0% | 10.9% | 11 |
| {Benz[a]anthracene,Benzo(e)acephenanthrylene,Indeno[1,2,3-cd]pyrene,Wood dust} | {Benzo(k)fluoranthene} | 100.0% | 10.9% | 11 |
| {Benz[a]anthracene,Benzo(e)acephenanthrylene,Benzo(k)fluoranthene,Wood dust} | {Indeno[1,2,3-cd]pyrene} | 100.0% | 10.9% | 11 |
| {Benzo(e)acephenanthrylene,Benzo(k)fluoranthene,Benzo[def]chrysene,Benzo[ghi]perylene} | {Benz[a]anthracene} | 100.0% | 14.9% | 15 |
| {Benz[a]anthracene,Benzo(k)fluoranthene,Benzo[def]chrysene,Benzo[ghi]perylene} | {Benzo(e)acephenanthrylene} | 100.0% | 14.9% | 15 |
| {Benz[a]anthracene,Benzo(e)acephenanthrylene,Benzo[def]chrysene,Benzo[ghi]perylene} | {Benzo(k)fluoranthene} | 100.0% | 14.9% | 15 |
| {Benz[a]anthracene,Benzo(e)acephenanthrylene,Benzo(k)fluoranthene,Benzo[ghi]perylene} | {Benzo[def]chrysene} | 100.0% | 14.9% | 15 |
| {Benz[a]anthracene,Benzo(e)acephenanthrylene,Benzo(k)fluoranthene,Benzo[def]chrysene} | {Benzo[ghi]perylene} | 100.0% | 14.9% | 15 |
| {Benzo(k)fluoranthene,Benzo[def]chrysene,Benzo[ghi]perylene,Wood dust} | {Benzo(e)acephenanthrylene} | 100.0% | 10.9% | 11 |
| {Benzo(e)acephenanthrylene,Benzo[def]chrysene,Benzo[ghi]perylene,Wood dust} | {Benzo(k)fluoranthene} | 100.0% | 10.9% | 11 |
| {Benzo(e)acephenanthrylene,Benzo(k)fluoranthene,Benzo[ghi]perylene,Wood dust} | {Benzo[def]chrysene} | 100.0% | 10.9% | 11 |
| {Benzo(e)acephenanthrylene,Benzo(k)fluoranthene,Benzo[def]chrysene,Wood dust} | {Benzo[ghi]perylene} | 100.0% | 10.9% | 11 |
| {Benzo(k)fluoranthene,Benzo[def]chrysene,Benzo[ghi]perylene,Wood dust} | {Benz[a]anthracene} | 100.0% | 10.9% | 11 |
| {Benz[a]anthracene,Benzo[def]chrysene,Benzo[ghi]perylene,Wood dust} | {Benzo(k)fluoranthene} | 100.0% | 10.9% | 11 |
| {Benz[a]anthracene,Benzo(k)fluoranthene,Benzo[ghi]perylene,Wood dust} | {Benzo[def]chrysene} | 100.0% | 10.9% | 11 |
| {Benz[a]anthracene,Benzo(k)fluoranthene,Benzo[def]chrysene,Wood dust} | {Benzo[ghi]perylene} | 100.0% | 10.9% | 11 |
| {Benzo(e)acephenanthrylene,Benzo[def]chrysene,Benzo[ghi]perylene,Wood dust} | {Benz[a]anthracene} | 100.0% | 10.9% | 11 |
| {Benz[a]anthracene,Benzo[def]chrysene,Benzo[ghi]perylene,Wood dust} | {Benzo(e)acephenanthrylene} | 100.0% | 10.9% | 11 |
| {Benz[a]anthracene,Benzo(e)acephenanthrylene,Benzo[ghi]perylene,Wood dust} | {Benzo[def]chrysene} | 100.0% | 10.9% | 11 |
| {Benz[a]anthracene,Benzo(e)acephenanthrylene,Benzo[def]chrysene,Wood dust} | {Benzo[ghi]perylene} | 100.0% | 10.9% | 11 |
| {Benzo(e)acephenanthrylene,Benzo(k)fluoranthene,Benzo[ghi]perylene,Wood dust} | {Benz[a]anthracene} | 100.0% | 10.9% | 11 |
| {Benz[a]anthracene,Benzo(k)fluoranthene,Benzo[ghi]perylene,Wood dust} | {Benzo(e)acephenanthrylene} | 100.0% | 10.9% | 11 |
| {Benz[a]anthracene,Benzo(e)acephenanthrylene,Benzo[ghi]perylene,Wood dust} | {Benzo(k)fluoranthene} | 100.0% | 10.9% | 11 |
| {Benz[a]anthracene,Benzo(e)acephenanthrylene,Benzo(k)fluoranthene,Wood dust} | {Benzo[ghi]perylene} | 100.0% | 10.9% | 11 |
| {Benzo(e)acephenanthrylene,Benzo(k)fluoranthene,Benzo[def]chrysene,Wood dust} | {Benz[a]anthracene} | 100.0% | 10.9% | 11 |
| {Benz[a]anthracene,Benzo(k)fluoranthene,Benzo[def]chrysene,Wood dust} | {Benzo(e)acephenanthrylene} | 100.0% | 10.9% | 11 |
| {Benz[a]anthracene,Benzo(e)acephenanthrylene,Benzo[def]chrysene,Wood dust} | {Benzo(k)fluoranthene} | 100.0% | 10.9% | 11 |
| {Benz[a]anthracene,Benzo(e)acephenanthrylene,Benzo(k)fluoranthene,Wood dust} | {Benzo[def]chrysene} | 100.0% | 10.9% | 11 |
| {Benzo(e)acephenanthrylene,Benzo(k)fluoranthene,Benzo[def]chrysene,Benzo[ghi]perylene,Indeno[1,2,3-cd]pyrene} | {Benz[a]anthracene} | 100.0% | 14.9% | 15 |
| {Benz[a]anthracene,Benzo(k)fluoranthene,Benzo[def]chrysene,Benzo[ghi]perylene,Indeno[1,2,3-cd]pyrene} | {Benzo(e)acephenanthrylene} | 100.0% | 14.9% | 15 |
| {Benz[a]anthracene,Benzo(e)acephenanthrylene,Benzo[def]chrysene,Benzo[ghi]perylene,Indeno[1,2,3-cd]pyrene} | {Benzo(k)fluoranthene} | 100.0% | 14.9% | 15 |
| {Benz[a]anthracene,Benzo(e)acephenanthrylene,Benzo(k)fluoranthene,Benzo[ghi]perylene,Indeno[1,2,3-cd]pyrene} | {Benzo[def]chrysene} | 100.0% | 14.9% | 15 |
| {Benz[a]anthracene,Benzo(e)acephenanthrylene,Benzo(k)fluoranthene,Benzo[def]chrysene,Indeno[1,2,3-cd]pyrene} | {Benzo[ghi]perylene} | 100.0% | 14.9% | 15 |
| {Benz[a]anthracene,Benzo(e)acephenanthrylene,Benzo(k)fluoranthene,Benzo[def]chrysene,Benzo[ghi]perylene} | {Indeno[1,2,3-cd]pyrene} | 100.0% | 14.9% | 15 |
| {Benzo(k)fluoranthene,Benzo[def]chrysene,Benzo[ghi]perylene,Indeno[1,2,3-cd]pyrene,Wood dust} | {Benzo(e)acephenanthrylene} | 100.0% | 10.9% | 11 |
| {Benzo(e)acephenanthrylene,Benzo[def]chrysene,Benzo[ghi]perylene,Indeno[1,2,3-cd]pyrene,Wood dust} | {Benzo(k)fluoranthene} | 100.0% | 10.9% | 11 |
| {Benzo(e)acephenanthrylene,Benzo(k)fluoranthene,Benzo[ghi]perylene,Indeno[1,2,3-cd]pyrene,Wood dust} | {Benzo[def]chrysene} | 100.0% | 10.9% | 11 |
| {Benzo(e)acephenanthrylene,Benzo(k)fluoranthene,Benzo[def]chrysene,Indeno[1,2,3-cd]pyrene,Wood dust} | {Benzo[ghi]perylene} | 100.0% | 10.9% | 11 |
| {Benzo(e)acephenanthrylene,Benzo(k)fluoranthene,Benzo[def]chrysene,Benzo[ghi]perylene,Wood dust} | {Indeno[1,2,3-cd]pyrene} | 100.0% | 10.9% | 11 |
| {Benzo(k)fluoranthene,Benzo[def]chrysene,Benzo[ghi]perylene,Indeno[1,2,3-cd]pyrene,Wood dust} | {Benz[a]anthracene} | 100.0% | 10.9% | 11 |
| {Benz[a]anthracene,Benzo[def]chrysene,Benzo[ghi]perylene,Indeno[1,2,3-cd]pyrene,Wood dust} | {Benzo(k)fluoranthene} | 100.0% | 10.9% | 11 |
| {Benz[a]anthracene,Benzo(k)fluoranthene,Benzo[ghi]perylene,Indeno[1,2,3-cd]pyrene,Wood dust} | {Benzo[def]chrysene} | 100.0% | 10.9% | 11 |
| {Benz[a]anthracene,Benzo(k)fluoranthene,Benzo[def]chrysene,Indeno[1,2,3-cd]pyrene,Wood dust} | {Benzo[ghi]perylene} | 100.0% | 10.9% | 11 |
| {Benz[a]anthracene,Benzo(k)fluoranthene,Benzo[def]chrysene,Benzo[ghi]perylene,Wood dust} | {Indeno[1,2,3-cd]pyrene} | 100.0% | 10.9% | 11 |
| {Benzo(e)acephenanthrylene,Benzo[def]chrysene,Benzo[ghi]perylene,Indeno[1,2,3-cd]pyrene,Wood dust} | {Benz[a]anthracene} | 100.0% | 10.9% | 11 |
| {Benz[a]anthracene,Benzo[def]chrysene,Benzo[ghi]perylene,Indeno[1,2,3-cd]pyrene,Wood dust} | {Benzo(e)acephenanthrylene} | 100.0% | 10.9% | 11 |
| {Benz[a]anthracene,Benzo(e)acephenanthrylene,Benzo[ghi]perylene,Indeno[1,2,3-cd]pyrene,Wood dust} | {Benzo[def]chrysene} | 100.0% | 10.9% | 11 |
| {Benz[a]anthracene,Benzo(e)acephenanthrylene,Benzo[def]chrysene,Indeno[1,2,3-cd]pyrene,Wood dust} | {Benzo[ghi]perylene} | 100.0% | 10.9% | 11 |
| {Benz[a]anthracene,Benzo(e)acephenanthrylene,Benzo[def]chrysene,Benzo[ghi]perylene,Wood dust} | {Indeno[1,2,3-cd]pyrene} | 100.0% | 10.9% | 11 |
| {Benzo(e)acephenanthrylene,Benzo(k)fluoranthene,Benzo[ghi]perylene,Indeno[1,2,3-cd]pyrene,Wood dust} | {Benz[a]anthracene} | 100.0% | 10.9% | 11 |
| {Benz[a]anthracene,Benzo(k)fluoranthene,Benzo[ghi]perylene,Indeno[1,2,3-cd]pyrene,Wood dust} | {Benzo(e)acephenanthrylene} | 100.0% | 10.9% | 11 |
| {Benz[a]anthracene,Benzo(e)acephenanthrylene,Benzo[ghi]perylene,Indeno[1,2,3-cd]pyrene,Wood dust} | {Benzo(k)fluoranthene} | 100.0% | 10.9% | 11 |
| {Benz[a]anthracene,Benzo(e)acephenanthrylene,Benzo(k)fluoranthene,Indeno[1,2,3-cd]pyrene,Wood dust} | {Benzo[ghi]perylene} | 100.0% | 10.9% | 11 |
| {Benz[a]anthracene,Benzo(e)acephenanthrylene,Benzo(k)fluoranthene,Benzo[ghi]perylene,Wood dust} | {Indeno[1,2,3-cd]pyrene} | 100.0% | 10.9% | 11 |
| {Benzo(e)acephenanthrylene,Benzo(k)fluoranthene,Benzo[def]chrysene,Indeno[1,2,3-cd]pyrene,Wood dust} | {Benz[a]anthracene} | 100.0% | 10.9% | 11 |
| {Benz[a]anthracene,Benzo(k)fluoranthene,Benzo[def]chrysene,Indeno[1,2,3-cd]pyrene,Wood dust} | {Benzo(e)acephenanthrylene} | 100.0% | 10.9% | 11 |
| {Benz[a]anthracene,Benzo(e)acephenanthrylene,Benzo[def]chrysene,Indeno[1,2,3-cd]pyrene,Wood dust} | {Benzo(k)fluoranthene} | 100.0% | 10.9% | 11 |
| {Benz[a]anthracene,Benzo(e)acephenanthrylene,Benzo(k)fluoranthene,Indeno[1,2,3-cd]pyrene,Wood dust} | {Benzo[def]chrysene} | 100.0% | 10.9% | 11 |
| {Benz[a]anthracene,Benzo(e)acephenanthrylene,Benzo(k)fluoranthene,Benzo[def]chrysene,Wood dust} | {Indeno[1,2,3-cd]pyrene} | 100.0% | 10.9% | 11 |
| {Benzo(e)acephenanthrylene,Benzo(k)fluoranthene,Benzo[def]chrysene,Benzo[ghi]perylene,Wood dust} | {Benz[a]anthracene} | 100.0% | 10.9% | 11 |
| {Benz[a]anthracene,Benzo(k)fluoranthene,Benzo[def]chrysene,Benzo[ghi]perylene,Wood dust} | {Benzo(e)acephenanthrylene} | 100.0% | 10.9% | 11 |
| {Benz[a]anthracene,Benzo(e)acephenanthrylene,Benzo[def]chrysene,Benzo[ghi]perylene,Wood dust} | {Benzo(k)fluoranthene} | 100.0% | 10.9% | 11 |
| {Benz[a]anthracene,Benzo(e)acephenanthrylene,Benzo(k)fluoranthene,Benzo[ghi]perylene,Wood dust} | {Benzo[def]chrysene} | 100.0% | 10.9% | 11 |
| {Benz[a]anthracene,Benzo(e)acephenanthrylene,Benzo(k)fluoranthene,Benzo[def]chrysene,Wood dust} | {Benzo[ghi]perylene} | 100.0% | 10.9% | 11 |
| {Benzo(e)acephenanthrylene,Benzo(k)fluoranthene,Benzo[def]chrysene,Benzo[ghi]perylene,Indeno[1,2,3-cd]pyrene,Wood dust} | {Benz[a]anthracene} | 100.0% | 10.9% | 11 |
| {Benz[a]anthracene,Benzo(k)fluoranthene,Benzo[def]chrysene,Benzo[ghi]perylene,Indeno[1,2,3-cd]pyrene,Wood dust} | {Benzo(e)acephenanthrylene} | 100.0% | 10.9% | 11 |
| {Benz[a]anthracene,Benzo(e)acephenanthrylene,Benzo[def]chrysene,Benzo[ghi]perylene,Indeno[1,2,3-cd]pyrene,Wood dust} | {Benzo(k)fluoranthene} | 100.0% | 10.9% | 11 |
| {Benz[a]anthracene,Benzo(e)acephenanthrylene,Benzo(k)fluoranthene,Benzo[ghi]perylene,Indeno[1,2,3-cd]pyrene,Wood dust} | {Benzo[def]chrysene} | 100.0% | 10.9% | 11 |
| {Benz[a]anthracene,Benzo(e)acephenanthrylene,Benzo(k)fluoranthene,Benzo[def]chrysene,Indeno[1,2,3-cd]pyrene,Wood dust} | {Benzo[ghi]perylene} | 100.0% | 10.9% | 11 |
| {Benz[a]anthracene,Benzo(e)acephenanthrylene,Benzo(k)fluoranthene,Benzo[def]chrysene,Benzo[ghi]perylene,Wood dust} | {Indeno[1,2,3-cd]pyrene} | 100.0% | 10.9% | 11 |
| {Lead} | {Wood dust} | 96.4% | 26.7% | 27 |
| {Formaldehyde} | {Wood dust} | 90.5% | 18.8% | 19 |
| {Toluene} | {Wood dust} | 81.2% | 12.9% | 13 |
| {Indeno[1,2,3-cd]pyrene} | {Wood dust} | 73.3% | 10.9% | 11 |
| {Benzo[ghi]perylene} | {Wood dust} | 73.3% | 10.9% | 11 |
| {Benzo[def]chrysene} | {Wood dust} | 73.3% | 10.9% | 11 |
| {Benzo(k)fluoranthene} | {Wood dust} | 73.3% | 10.9% | 11 |
| {Benzo(e)acephenanthrylene} | {Wood dust} | 73.3% | 10.9% | 11 |
| {Benz[a]anthracene} | {Wood dust} | 73.3% | 10.9% | 11 |
| {Benzo[ghi]perylene,Indeno[1,2,3-cd]pyrene} | {Wood dust} | 73.3% | 10.9% | 11 |
| {Benzo[def]chrysene,Indeno[1,2,3-cd]pyrene} | {Wood dust} | 73.3% | 10.9% | 11 |
| {Benzo(k)fluoranthene,Indeno[1,2,3-cd]pyrene} | {Wood dust} | 73.3% | 10.9% | 11 |
| {Benzo(e)acephenanthrylene,Indeno[1,2,3-cd]pyrene} | {Wood dust} | 73.3% | 10.9% | 11 |
| {Benz[a]anthracene,Indeno[1,2,3-cd]pyrene} | {Wood dust} | 73.3% | 10.9% | 11 |
| {Benzo[def]chrysene,Benzo[ghi]perylene} | {Wood dust} | 73.3% | 10.9% | 11 |
| {Benzo(k)fluoranthene,Benzo[ghi]perylene} | {Wood dust} | 73.3% | 10.9% | 11 |
| {Benzo(e)acephenanthrylene,Benzo[ghi]perylene} | {Wood dust} | 73.3% | 10.9% | 11 |
| {Benz[a]anthracene,Benzo[ghi]perylene} | {Wood dust} | 73.3% | 10.9% | 11 |
| {Benzo(k)fluoranthene,Benzo[def]chrysene} | {Wood dust} | 73.3% | 10.9% | 11 |
| {Benzo(e)acephenanthrylene,Benzo[def]chrysene} | {Wood dust} | 73.3% | 10.9% | 11 |
| {Benz[a]anthracene,Benzo[def]chrysene} | {Wood dust} | 73.3% | 10.9% | 11 |
| {Benzo(e)acephenanthrylene,Benzo(k)fluoranthene} | {Wood dust} | 73.3% | 10.9% | 11 |
| {Benz[a]anthracene,Benzo(k)fluoranthene} | {Wood dust} | 73.3% | 10.9% | 11 |
| {Benz[a]anthracene,Benzo(e)acephenanthrylene} | {Wood dust} | 73.3% | 10.9% | 11 |
| {Benzo[def]chrysene,Benzo[ghi]perylene,Indeno[1,2,3-cd]pyrene} | {Wood dust} | 73.3% | 10.9% | 11 |
| {Benzo(k)fluoranthene,Benzo[ghi]perylene,Indeno[1,2,3-cd]pyrene} | {Wood dust} | 73.3% | 10.9% | 11 |
| {Benzo(e)acephenanthrylene,Benzo[ghi]perylene,Indeno[1,2,3-cd]pyrene} | {Wood dust} | 73.3% | 10.9% | 11 |
| {Benz[a]anthracene,Benzo[ghi]perylene,Indeno[1,2,3-cd]pyrene} | {Wood dust} | 73.3% | 10.9% | 11 |
| {Benzo(k)fluoranthene,Benzo[def]chrysene,Indeno[1,2,3-cd]pyrene} | {Wood dust} | 73.3% | 10.9% | 11 |
| {Benzo(e)acephenanthrylene,Benzo[def]chrysene,Indeno[1,2,3-cd]pyrene} | {Wood dust} | 73.3% | 10.9% | 11 |
| {Benz[a]anthracene,Benzo[def]chrysene,Indeno[1,2,3-cd]pyrene} | {Wood dust} | 73.3% | 10.9% | 11 |
| {Benzo(e)acephenanthrylene,Benzo(k)fluoranthene,Indeno[1,2,3-cd]pyrene} | {Wood dust} | 73.3% | 10.9% | 11 |
| {Benz[a]anthracene,Benzo(k)fluoranthene,Indeno[1,2,3-cd]pyrene} | {Wood dust} | 73.3% | 10.9% | 11 |
| {Benz[a]anthracene,Benzo(e)acephenanthrylene,Indeno[1,2,3-cd]pyrene} | {Wood dust} | 73.3% | 10.9% | 11 |
| {Benzo(k)fluoranthene,Benzo[def]chrysene,Benzo[ghi]perylene} | {Wood dust} | 73.3% | 10.9% | 11 |
| {Benzo(e)acephenanthrylene,Benzo[def]chrysene,Benzo[ghi]perylene} | {Wood dust} | 73.3% | 10.9% | 11 |
| {Benz[a]anthracene,Benzo[def]chrysene,Benzo[ghi]perylene} | {Wood dust} | 73.3% | 10.9% | 11 |
| {Benzo(e)acephenanthrylene,Benzo(k)fluoranthene,Benzo[ghi]perylene} | {Wood dust} | 73.3% | 10.9% | 11 |
| {Benz[a]anthracene,Benzo(k)fluoranthene,Benzo[ghi]perylene} | {Wood dust} | 73.3% | 10.9% | 11 |
| {Benz[a]anthracene,Benzo(e)acephenanthrylene,Benzo[ghi]perylene} | {Wood dust} | 73.3% | 10.9% | 11 |
| {Benzo(e)acephenanthrylene,Benzo(k)fluoranthene,Benzo[def]chrysene} | {Wood dust} | 73.3% | 10.9% | 11 |
| {Benz[a]anthracene,Benzo(k)fluoranthene,Benzo[def]chrysene} | {Wood dust} | 73.3% | 10.9% | 11 |
| {Benz[a]anthracene,Benzo(e)acephenanthrylene,Benzo[def]chrysene} | {Wood dust} | 73.3% | 10.9% | 11 |
| {Benz[a]anthracene,Benzo(e)acephenanthrylene,Benzo(k)fluoranthene} | {Wood dust} | 73.3% | 10.9% | 11 |
| {Benzo(k)fluoranthene,Benzo[def]chrysene,Benzo[ghi]perylene,Indeno[1,2,3-cd]pyrene} | {Wood dust} | 73.3% | 10.9% | 11 |
| {Benzo(e)acephenanthrylene,Benzo[def]chrysene,Benzo[ghi]perylene,Indeno[1,2,3-cd]pyrene} | {Wood dust} | 73.3% | 10.9% | 11 |
| {Benz[a]anthracene,Benzo[def]chrysene,Benzo[ghi]perylene,Indeno[1,2,3-cd]pyrene} | {Wood dust} | 73.3% | 10.9% | 11 |
| {Benzo(e)acephenanthrylene,Benzo(k)fluoranthene,Benzo[ghi]perylene,Indeno[1,2,3-cd]pyrene} | {Wood dust} | 73.3% | 10.9% | 11 |
| {Benz[a]anthracene,Benzo(k)fluoranthene,Benzo[ghi]perylene,Indeno[1,2,3-cd]pyrene} | {Wood dust} | 73.3% | 10.9% | 11 |
| {Benz[a]anthracene,Benzo(e)acephenanthrylene,Benzo[ghi]perylene,Indeno[1,2,3-cd]pyrene} | {Wood dust} | 73.3% | 10.9% | 11 |
| {Benzo(e)acephenanthrylene,Benzo(k)fluoranthene,Benzo[def]chrysene,Indeno[1,2,3-cd]pyrene} | {Wood dust} | 73.3% | 10.9% | 11 |
| {Benz[a]anthracene,Benzo(k)fluoranthene,Benzo[def]chrysene,Indeno[1,2,3-cd]pyrene} | {Wood dust} | 73.3% | 10.9% | 11 |
| {Benz[a]anthracene,Benzo(e)acephenanthrylene,Benzo[def]chrysene,Indeno[1,2,3-cd]pyrene} | {Wood dust} | 73.3% | 10.9% | 11 |
| {Benz[a]anthracene,Benzo(e)acephenanthrylene,Benzo(k)fluoranthene,Indeno[1,2,3-cd]pyrene} | {Wood dust} | 73.3% | 10.9% | 11 |
| {Benzo(e)acephenanthrylene,Benzo(k)fluoranthene,Benzo[def]chrysene,Benzo[ghi]perylene} | {Wood dust} | 73.3% | 10.9% | 11 |
| {Benz[a]anthracene,Benzo(k)fluoranthene,Benzo[def]chrysene,Benzo[ghi]perylene} | {Wood dust} | 73.3% | 10.9% | 11 |
| {Benz[a]anthracene,Benzo(e)acephenanthrylene,Benzo[def]chrysene,Benzo[ghi]perylene} | {Wood dust} | 73.3% | 10.9% | 11 |
| {Benz[a]anthracene,Benzo(e)acephenanthrylene,Benzo(k)fluoranthene,Benzo[ghi]perylene} | {Wood dust} | 73.3% | 10.9% | 11 |
| {Benz[a]anthracene,Benzo(e)acephenanthrylene,Benzo(k)fluoranthene,Benzo[def]chrysene} | {Wood dust} | 73.3% | 10.9% | 11 |
| {Benzo(e)acephenanthrylene,Benzo(k)fluoranthene,Benzo[def]chrysene,Benzo[ghi]perylene,Indeno[1,2,3-cd]pyrene} | {Wood dust} | 73.3% | 10.9% | 11 |
| {Benz[a]anthracene,Benzo(k)fluoranthene,Benzo[def]chrysene,Benzo[ghi]perylene,Indeno[1,2,3-cd]pyrene} | {Wood dust} | 73.3% | 10.9% | 11 |
| {Benz[a]anthracene,Benzo(e)acephenanthrylene,Benzo[def]chrysene,Benzo[ghi]perylene,Indeno[1,2,3-cd]pyrene} | {Wood dust} | 73.3% | 10.9% | 11 |
| {Benz[a]anthracene,Benzo(e)acephenanthrylene,Benzo(k)fluoranthene,Benzo[ghi]perylene,Indeno[1,2,3-cd]pyrene} | {Wood dust} | 73.3% | 10.9% | 11 |
| {Benz[a]anthracene,Benzo(e)acephenanthrylene,Benzo(k)fluoranthene,Benzo[def]chrysene,Indeno[1,2,3-cd]pyrene} | {Wood dust} | 73.3% | 10.9% | 11 |
| {Benz[a]anthracene,Benzo(e)acephenanthrylene,Benzo(k)fluoranthene,Benzo[def]chrysene,Benzo[ghi]perylene} | {Wood dust} | 73.3% | 10.9% | 11 |
| {Benz[a]anthracene,Benzo(e)acephenanthrylene,Benzo(k)fluoranthene,Benzo[def]chrysene,Benzo[ghi]perylene,Indeno[1,2,3-cd]pyrene} | {Wood dust} | 73.3% | 10.9% | 11 |
| {Wood dust} | {Lead} | 32.5% | 26.7% | 27 |
| {Wood dust} | {Formaldehyde} | 22.9% | 18.8% | 19 |
| {Wood dust} | {Toluene} | 15.7% | 12.9% | 13 |
| {Wood dust} | {Indeno[1,2,3-cd]pyrene} | 13.3% | 10.9% | 11 |
| {Wood dust} | {Benzo[ghi]perylene} | 13.3% | 10.9% | 11 |
| {Wood dust} | {Benzo[def]chrysene} | 13.3% | 10.9% | 11 |
| {Wood dust} | {Benzo(k)fluoranthene} | 13.3% | 10.9% | 11 |
| {Wood dust} | {Benzo(e)acephenanthrylene} | 13.3% | 10.9% | 11 |
| {Wood dust} | {Benz[a]anthracene} | 13.3% | 10.9% | 11 |

##### Association rules with the largest lift among WS exposed to a minimum of 2 agents. Minimum level of support = 0.1 %, minimum confidence 0.1.

| Antecedent | Consequent | Lift | % Conf (A\(\rightarrow\)C) | % Conf (C\(\rightarrow\)A) | % Support | N WS |
| --- | --- | --- | --- | --- | --- | --- |
| {Indeno[1,2,3-cd]pyrene} | {Benzo[ghi]perylene} | 673.3 | 100.0% | 100.0% | 14.9% | 15 |
| {Indeno[1,2,3-cd]pyrene} | {Benzo[def]chrysene} | 673.3 | 100.0% | 100.0% | 14.9% | 15 |
| {Indeno[1,2,3-cd]pyrene} | {Benzo(k)fluoranthene} | 673.3 | 100.0% | 100.0% | 14.9% | 15 |
| {Indeno[1,2,3-cd]pyrene} | {Benzo(e)acephenanthrylene} | 673.3 | 100.0% | 100.0% | 14.9% | 15 |
| {Indeno[1,2,3-cd]pyrene} | {Benz[a]anthracene} | 673.3 | 100.0% | 100.0% | 14.9% | 15 |
| {Benzo[ghi]perylene} | {Benzo[def]chrysene} | 673.3 | 100.0% | 100.0% | 14.9% | 15 |
| {Benzo[ghi]perylene} | {Benzo(k)fluoranthene} | 673.3 | 100.0% | 100.0% | 14.9% | 15 |
| {Benzo[ghi]perylene} | {Benzo(e)acephenanthrylene} | 673.3 | 100.0% | 100.0% | 14.9% | 15 |
| {Benzo[ghi]perylene} | {Benz[a]anthracene} | 673.3 | 100.0% | 100.0% | 14.9% | 15 |
| {Benzo[def]chrysene} | {Benzo(k)fluoranthene} | 673.3 | 100.0% | 100.0% | 14.9% | 15 |
| {Benzo[def]chrysene} | {Benzo(e)acephenanthrylene} | 673.3 | 100.0% | 100.0% | 14.9% | 15 |
| {Benzo[def]chrysene} | {Benz[a]anthracene} | 673.3 | 100.0% | 100.0% | 14.9% | 15 |
| {Benzo(k)fluoranthene} | {Benzo(e)acephenanthrylene} | 673.3 | 100.0% | 100.0% | 14.9% | 15 |
| {Benzo(k)fluoranthene} | {Benz[a]anthracene} | 673.3 | 100.0% | 100.0% | 14.9% | 15 |
| {Benzo(e)acephenanthrylene} | {Benz[a]anthracene} | 673.3 | 100.0% | 100.0% | 14.9% | 15 |
| {Benzo[ghi]perylene,Indeno[1,2,3-cd]pyrene} | {Benzo[def]chrysene} | 673.3 | 100.0% | 100.0% | 14.9% | 15 |
| {Benzo[ghi]perylene,Indeno[1,2,3-cd]pyrene} | {Benzo(k)fluoranthene} | 673.3 | 100.0% | 100.0% | 14.9% | 15 |
| {Benzo[ghi]perylene,Indeno[1,2,3-cd]pyrene} | {Benzo(e)acephenanthrylene} | 673.3 | 100.0% | 100.0% | 14.9% | 15 |
| {Benzo[ghi]perylene,Indeno[1,2,3-cd]pyrene} | {Benz[a]anthracene} | 673.3 | 100.0% | 100.0% | 14.9% | 15 |
| {Benzo[def]chrysene,Indeno[1,2,3-cd]pyrene} | {Benzo(k)fluoranthene} | 673.3 | 100.0% | 100.0% | 14.9% | 15 |
| {Benzo[def]chrysene,Indeno[1,2,3-cd]pyrene} | {Benzo(e)acephenanthrylene} | 673.3 | 100.0% | 100.0% | 14.9% | 15 |
| {Benzo[def]chrysene,Indeno[1,2,3-cd]pyrene} | {Benz[a]anthracene} | 673.3 | 100.0% | 100.0% | 14.9% | 15 |
| {Benzo(k)fluoranthene,Indeno[1,2,3-cd]pyrene} | {Benzo(e)acephenanthrylene} | 673.3 | 100.0% | 100.0% | 14.9% | 15 |
| {Benzo(k)fluoranthene,Indeno[1,2,3-cd]pyrene} | {Benz[a]anthracene} | 673.3 | 100.0% | 100.0% | 14.9% | 15 |
| {Benzo(e)acephenanthrylene,Indeno[1,2,3-cd]pyrene} | {Benz[a]anthracene} | 673.3 | 100.0% | 100.0% | 14.9% | 15 |
| {Benzo[def]chrysene,Benzo[ghi]perylene} | {Benzo(k)fluoranthene} | 673.3 | 100.0% | 100.0% | 14.9% | 15 |
| {Benzo[def]chrysene,Benzo[ghi]perylene} | {Benzo(e)acephenanthrylene} | 673.3 | 100.0% | 100.0% | 14.9% | 15 |
| {Benzo[def]chrysene,Benzo[ghi]perylene} | {Benz[a]anthracene} | 673.3 | 100.0% | 100.0% | 14.9% | 15 |
| {Benzo(k)fluoranthene,Benzo[ghi]perylene} | {Benzo(e)acephenanthrylene} | 673.3 | 100.0% | 100.0% | 14.9% | 15 |
| {Benzo(k)fluoranthene,Benzo[ghi]perylene} | {Benz[a]anthracene} | 673.3 | 100.0% | 100.0% | 14.9% | 15 |
| {Benzo(e)acephenanthrylene,Benzo[ghi]perylene} | {Benz[a]anthracene} | 673.3 | 100.0% | 100.0% | 14.9% | 15 |
| {Benzo(k)fluoranthene,Benzo[def]chrysene} | {Benzo(e)acephenanthrylene} | 673.3 | 100.0% | 100.0% | 14.9% | 15 |
| {Benzo(k)fluoranthene,Benzo[def]chrysene} | {Benz[a]anthracene} | 673.3 | 100.0% | 100.0% | 14.9% | 15 |
| {Benzo(e)acephenanthrylene,Benzo[def]chrysene} | {Benz[a]anthracene} | 673.3 | 100.0% | 100.0% | 14.9% | 15 |
| {Benzo(e)acephenanthrylene,Benzo(k)fluoranthene} | {Benz[a]anthracene} | 673.3 | 100.0% | 100.0% | 14.9% | 15 |
| {Benzo[def]chrysene,Benzo[ghi]perylene,Indeno[1,2,3-cd]pyrene} | {Benzo(k)fluoranthene} | 673.3 | 100.0% | 100.0% | 14.9% | 15 |
| {Benzo[def]chrysene,Benzo[ghi]perylene,Indeno[1,2,3-cd]pyrene} | {Benzo(e)acephenanthrylene} | 673.3 | 100.0% | 100.0% | 14.9% | 15 |
| {Benzo[def]chrysene,Benzo[ghi]perylene,Indeno[1,2,3-cd]pyrene} | {Benz[a]anthracene} | 673.3 | 100.0% | 100.0% | 14.9% | 15 |
| {Benzo(k)fluoranthene,Benzo[ghi]perylene,Indeno[1,2,3-cd]pyrene} | {Benzo(e)acephenanthrylene} | 673.3 | 100.0% | 100.0% | 14.9% | 15 |
| {Benzo(k)fluoranthene,Benzo[ghi]perylene,Indeno[1,2,3-cd]pyrene} | {Benz[a]anthracene} | 673.3 | 100.0% | 100.0% | 14.9% | 15 |
| {Benzo(e)acephenanthrylene,Benzo[ghi]perylene,Indeno[1,2,3-cd]pyrene} | {Benz[a]anthracene} | 673.3 | 100.0% | 100.0% | 14.9% | 15 |
| {Benzo(k)fluoranthene,Benzo[def]chrysene,Indeno[1,2,3-cd]pyrene} | {Benzo(e)acephenanthrylene} | 673.3 | 100.0% | 100.0% | 14.9% | 15 |
| {Benzo(k)fluoranthene,Benzo[def]chrysene,Indeno[1,2,3-cd]pyrene} | {Benz[a]anthracene} | 673.3 | 100.0% | 100.0% | 14.9% | 15 |
| {Benzo(e)acephenanthrylene,Benzo[def]chrysene,Indeno[1,2,3-cd]pyrene} | {Benz[a]anthracene} | 673.3 | 100.0% | 100.0% | 14.9% | 15 |
| {Benzo(e)acephenanthrylene,Benzo(k)fluoranthene,Indeno[1,2,3-cd]pyrene} | {Benz[a]anthracene} | 673.3 | 100.0% | 100.0% | 14.9% | 15 |
| {Benzo(k)fluoranthene,Benzo[def]chrysene,Benzo[ghi]perylene} | {Benzo(e)acephenanthrylene} | 673.3 | 100.0% | 100.0% | 14.9% | 15 |
| {Benzo(k)fluoranthene,Benzo[def]chrysene,Benzo[ghi]perylene} | {Benz[a]anthracene} | 673.3 | 100.0% | 100.0% | 14.9% | 15 |
| {Benzo(e)acephenanthrylene,Benzo[def]chrysene,Benzo[ghi]perylene} | {Benz[a]anthracene} | 673.3 | 100.0% | 100.0% | 14.9% | 15 |
| {Benzo(e)acephenanthrylene,Benzo(k)fluoranthene,Benzo[ghi]perylene} | {Benz[a]anthracene} | 673.3 | 100.0% | 100.0% | 14.9% | 15 |
| {Benzo(e)acephenanthrylene,Benzo(k)fluoranthene,Benzo[def]chrysene} | {Benz[a]anthracene} | 673.3 | 100.0% | 100.0% | 14.9% | 15 |
| {Benzo(k)fluoranthene,Benzo[def]chrysene,Benzo[ghi]perylene,Indeno[1,2,3-cd]pyrene} | {Benzo(e)acephenanthrylene} | 673.3 | 100.0% | 100.0% | 14.9% | 15 |
| {Benzo(k)fluoranthene,Benzo[def]chrysene,Benzo[ghi]perylene,Indeno[1,2,3-cd]pyrene} | {Benz[a]anthracene} | 673.3 | 100.0% | 100.0% | 14.9% | 15 |
| {Benzo(e)acephenanthrylene,Benzo[def]chrysene,Benzo[ghi]perylene,Indeno[1,2,3-cd]pyrene} | {Benz[a]anthracene} | 673.3 | 100.0% | 100.0% | 14.9% | 15 |
| {Benzo(e)acephenanthrylene,Benzo(k)fluoranthene,Benzo[ghi]perylene,Indeno[1,2,3-cd]pyrene} | {Benz[a]anthracene} | 673.3 | 100.0% | 100.0% | 14.9% | 15 |
| {Benzo(e)acephenanthrylene,Benzo(k)fluoranthene,Benzo[def]chrysene,Indeno[1,2,3-cd]pyrene} | {Benz[a]anthracene} | 673.3 | 100.0% | 100.0% | 14.9% | 15 |
| {Benzo(e)acephenanthrylene,Benzo(k)fluoranthene,Benzo[def]chrysene,Benzo[ghi]perylene} | {Benz[a]anthracene} | 673.3 | 100.0% | 100.0% | 14.9% | 15 |
| {Benzo(e)acephenanthrylene,Benzo(k)fluoranthene,Benzo[def]chrysene,Benzo[ghi]perylene,Indeno[1,2,3-cd]pyrene} | {Benz[a]anthracene} | 673.3 | 100.0% | 100.0% | 14.9% | 15 |
| {Lead} | {Wood dust} | 117.3 | 96.4% | 32.5% | 26.7% | 27 |
| {Formaldehyde} | {Wood dust} | 110.1 | 90.5% | 22.9% | 18.8% | 19 |
| {Toluene} | {Wood dust} | 98.9 | 81.2% | 15.7% | 12.9% | 13 |
| {Indeno[1,2,3-cd]pyrene} | {Wood dust} | 89.2 | 73.3% | 13.3% | 10.9% | 11 |
| {Benzo[ghi]perylene} | {Wood dust} | 89.2 | 73.3% | 13.3% | 10.9% | 11 |
| {Benzo[def]chrysene} | {Wood dust} | 89.2 | 73.3% | 13.3% | 10.9% | 11 |
| {Benzo(k)fluoranthene} | {Wood dust} | 89.2 | 73.3% | 13.3% | 10.9% | 11 |
| {Benzo(e)acephenanthrylene} | {Wood dust} | 89.2 | 73.3% | 13.3% | 10.9% | 11 |
| {Benz[a]anthracene} | {Wood dust} | 89.2 | 73.3% | 13.3% | 10.9% | 11 |
| {Benzo[ghi]perylene,Indeno[1,2,3-cd]pyrene} | {Wood dust} | 89.2 | 73.3% | 13.3% | 10.9% | 11 |
| {Benzo[def]chrysene,Indeno[1,2,3-cd]pyrene} | {Wood dust} | 89.2 | 73.3% | 13.3% | 10.9% | 11 |
| {Benzo(k)fluoranthene,Indeno[1,2,3-cd]pyrene} | {Wood dust} | 89.2 | 73.3% | 13.3% | 10.9% | 11 |
| {Benzo(e)acephenanthrylene,Indeno[1,2,3-cd]pyrene} | {Wood dust} | 89.2 | 73.3% | 13.3% | 10.9% | 11 |
| {Benz[a]anthracene,Indeno[1,2,3-cd]pyrene} | {Wood dust} | 89.2 | 73.3% | 13.3% | 10.9% | 11 |
| {Benzo[def]chrysene,Benzo[ghi]perylene} | {Wood dust} | 89.2 | 73.3% | 13.3% | 10.9% | 11 |
| {Benzo(k)fluoranthene,Benzo[ghi]perylene} | {Wood dust} | 89.2 | 73.3% | 13.3% | 10.9% | 11 |
| {Benzo(e)acephenanthrylene,Benzo[ghi]perylene} | {Wood dust} | 89.2 | 73.3% | 13.3% | 10.9% | 11 |
| {Benz[a]anthracene,Benzo[ghi]perylene} | {Wood dust} | 89.2 | 73.3% | 13.3% | 10.9% | 11 |
| {Benzo(k)fluoranthene,Benzo[def]chrysene} | {Wood dust} | 89.2 | 73.3% | 13.3% | 10.9% | 11 |
| {Benzo(e)acephenanthrylene,Benzo[def]chrysene} | {Wood dust} | 89.2 | 73.3% | 13.3% | 10.9% | 11 |
| {Benz[a]anthracene,Benzo[def]chrysene} | {Wood dust} | 89.2 | 73.3% | 13.3% | 10.9% | 11 |
| {Benzo(e)acephenanthrylene,Benzo(k)fluoranthene} | {Wood dust} | 89.2 | 73.3% | 13.3% | 10.9% | 11 |
| {Benz[a]anthracene,Benzo(k)fluoranthene} | {Wood dust} | 89.2 | 73.3% | 13.3% | 10.9% | 11 |
| {Benz[a]anthracene,Benzo(e)acephenanthrylene} | {Wood dust} | 89.2 | 73.3% | 13.3% | 10.9% | 11 |
| {Benzo[def]chrysene,Benzo[ghi]perylene,Indeno[1,2,3-cd]pyrene} | {Wood dust} | 89.2 | 73.3% | 13.3% | 10.9% | 11 |
| {Benzo(k)fluoranthene,Benzo[ghi]perylene,Indeno[1,2,3-cd]pyrene} | {Wood dust} | 89.2 | 73.3% | 13.3% | 10.9% | 11 |
| {Benzo(e)acephenanthrylene,Benzo[ghi]perylene,Indeno[1,2,3-cd]pyrene} | {Wood dust} | 89.2 | 73.3% | 13.3% | 10.9% | 11 |
| {Benz[a]anthracene,Benzo[ghi]perylene,Indeno[1,2,3-cd]pyrene} | {Wood dust} | 89.2 | 73.3% | 13.3% | 10.9% | 11 |
| {Benzo(k)fluoranthene,Benzo[def]chrysene,Indeno[1,2,3-cd]pyrene} | {Wood dust} | 89.2 | 73.3% | 13.3% | 10.9% | 11 |
| {Benzo(e)acephenanthrylene,Benzo[def]chrysene,Indeno[1,2,3-cd]pyrene} | {Wood dust} | 89.2 | 73.3% | 13.3% | 10.9% | 11 |
| {Benz[a]anthracene,Benzo[def]chrysene,Indeno[1,2,3-cd]pyrene} | {Wood dust} | 89.2 | 73.3% | 13.3% | 10.9% | 11 |
| {Benzo(e)acephenanthrylene,Benzo(k)fluoranthene,Indeno[1,2,3-cd]pyrene} | {Wood dust} | 89.2 | 73.3% | 13.3% | 10.9% | 11 |
| {Benz[a]anthracene,Benzo(k)fluoranthene,Indeno[1,2,3-cd]pyrene} | {Wood dust} | 89.2 | 73.3% | 13.3% | 10.9% | 11 |
| {Benz[a]anthracene,Benzo(e)acephenanthrylene,Indeno[1,2,3-cd]pyrene} | {Wood dust} | 89.2 | 73.3% | 13.3% | 10.9% | 11 |
| {Benzo(k)fluoranthene,Benzo[def]chrysene,Benzo[ghi]perylene} | {Wood dust} | 89.2 | 73.3% | 13.3% | 10.9% | 11 |
| {Benzo(e)acephenanthrylene,Benzo[def]chrysene,Benzo[ghi]perylene} | {Wood dust} | 89.2 | 73.3% | 13.3% | 10.9% | 11 |
| {Benz[a]anthracene,Benzo[def]chrysene,Benzo[ghi]perylene} | {Wood dust} | 89.2 | 73.3% | 13.3% | 10.9% | 11 |
| {Benzo(e)acephenanthrylene,Benzo(k)fluoranthene,Benzo[ghi]perylene} | {Wood dust} | 89.2 | 73.3% | 13.3% | 10.9% | 11 |
| {Benz[a]anthracene,Benzo(k)fluoranthene,Benzo[ghi]perylene} | {Wood dust} | 89.2 | 73.3% | 13.3% | 10.9% | 11 |
| {Benz[a]anthracene,Benzo(e)acephenanthrylene,Benzo[ghi]perylene} | {Wood dust} | 89.2 | 73.3% | 13.3% | 10.9% | 11 |
| {Benzo(e)acephenanthrylene,Benzo(k)fluoranthene,Benzo[def]chrysene} | {Wood dust} | 89.2 | 73.3% | 13.3% | 10.9% | 11 |
| {Benz[a]anthracene,Benzo(k)fluoranthene,Benzo[def]chrysene} | {Wood dust} | 89.2 | 73.3% | 13.3% | 10.9% | 11 |
| {Benz[a]anthracene,Benzo(e)acephenanthrylene,Benzo[def]chrysene} | {Wood dust} | 89.2 | 73.3% | 13.3% | 10.9% | 11 |
| {Benz[a]anthracene,Benzo(e)acephenanthrylene,Benzo(k)fluoranthene} | {Wood dust} | 89.2 | 73.3% | 13.3% | 10.9% | 11 |
| {Benzo(k)fluoranthene,Benzo[def]chrysene,Benzo[ghi]perylene,Indeno[1,2,3-cd]pyrene} | {Wood dust} | 89.2 | 73.3% | 13.3% | 10.9% | 11 |
| {Benzo(e)acephenanthrylene,Benzo[def]chrysene,Benzo[ghi]perylene,Indeno[1,2,3-cd]pyrene} | {Wood dust} | 89.2 | 73.3% | 13.3% | 10.9% | 11 |
| {Benz[a]anthracene,Benzo[def]chrysene,Benzo[ghi]perylene,Indeno[1,2,3-cd]pyrene} | {Wood dust} | 89.2 | 73.3% | 13.3% | 10.9% | 11 |
| {Benzo(e)acephenanthrylene,Benzo(k)fluoranthene,Benzo[ghi]perylene,Indeno[1,2,3-cd]pyrene} | {Wood dust} | 89.2 | 73.3% | 13.3% | 10.9% | 11 |
| {Benz[a]anthracene,Benzo(k)fluoranthene,Benzo[ghi]perylene,Indeno[1,2,3-cd]pyrene} | {Wood dust} | 89.2 | 73.3% | 13.3% | 10.9% | 11 |
| {Benz[a]anthracene,Benzo(e)acephenanthrylene,Benzo[ghi]perylene,Indeno[1,2,3-cd]pyrene} | {Wood dust} | 89.2 | 73.3% | 13.3% | 10.9% | 11 |
| {Benzo(e)acephenanthrylene,Benzo(k)fluoranthene,Benzo[def]chrysene,Indeno[1,2,3-cd]pyrene} | {Wood dust} | 89.2 | 73.3% | 13.3% | 10.9% | 11 |
| {Benz[a]anthracene,Benzo(k)fluoranthene,Benzo[def]chrysene,Indeno[1,2,3-cd]pyrene} | {Wood dust} | 89.2 | 73.3% | 13.3% | 10.9% | 11 |
| {Benz[a]anthracene,Benzo(e)acephenanthrylene,Benzo[def]chrysene,Indeno[1,2,3-cd]pyrene} | {Wood dust} | 89.2 | 73.3% | 13.3% | 10.9% | 11 |
| {Benz[a]anthracene,Benzo(e)acephenanthrylene,Benzo(k)fluoranthene,Indeno[1,2,3-cd]pyrene} | {Wood dust} | 89.2 | 73.3% | 13.3% | 10.9% | 11 |
| {Benzo(e)acephenanthrylene,Benzo(k)fluoranthene,Benzo[def]chrysene,Benzo[ghi]perylene} | {Wood dust} | 89.2 | 73.3% | 13.3% | 10.9% | 11 |
| {Benz[a]anthracene,Benzo(k)fluoranthene,Benzo[def]chrysene,Benzo[ghi]perylene} | {Wood dust} | 89.2 | 73.3% | 13.3% | 10.9% | 11 |
| {Benz[a]anthracene,Benzo(e)acephenanthrylene,Benzo[def]chrysene,Benzo[ghi]perylene} | {Wood dust} | 89.2 | 73.3% | 13.3% | 10.9% | 11 |
| {Benz[a]anthracene,Benzo(e)acephenanthrylene,Benzo(k)fluoranthene,Benzo[ghi]perylene} | {Wood dust} | 89.2 | 73.3% | 13.3% | 10.9% | 11 |
| {Benz[a]anthracene,Benzo(e)acephenanthrylene,Benzo(k)fluoranthene,Benzo[def]chrysene} | {Wood dust} | 89.2 | 73.3% | 13.3% | 10.9% | 11 |
| {Benzo(e)acephenanthrylene,Benzo(k)fluoranthene,Benzo[def]chrysene,Benzo[ghi]perylene,Indeno[1,2,3-cd]pyrene} | {Wood dust} | 89.2 | 73.3% | 13.3% | 10.9% | 11 |
| {Benz[a]anthracene,Benzo(k)fluoranthene,Benzo[def]chrysene,Benzo[ghi]perylene,Indeno[1,2,3-cd]pyrene} | {Wood dust} | 89.2 | 73.3% | 13.3% | 10.9% | 11 |
| {Benz[a]anthracene,Benzo(e)acephenanthrylene,Benzo[def]chrysene,Benzo[ghi]perylene,Indeno[1,2,3-cd]pyrene} | {Wood dust} | 89.2 | 73.3% | 13.3% | 10.9% | 11 |
| {Benz[a]anthracene,Benzo(e)acephenanthrylene,Benzo(k)fluoranthene,Benzo[ghi]perylene,Indeno[1,2,3-cd]pyrene} | {Wood dust} | 89.2 | 73.3% | 13.3% | 10.9% | 11 |
| {Benz[a]anthracene,Benzo(e)acephenanthrylene,Benzo(k)fluoranthene,Benzo[def]chrysene,Indeno[1,2,3-cd]pyrene} | {Wood dust} | 89.2 | 73.3% | 13.3% | 10.9% | 11 |
| {Benz[a]anthracene,Benzo(e)acephenanthrylene,Benzo(k)fluoranthene,Benzo[def]chrysene,Benzo[ghi]perylene} | {Wood dust} | 89.2 | 73.3% | 13.3% | 10.9% | 11 |
| {Benz[a]anthracene,Benzo(e)acephenanthrylene,Benzo(k)fluoranthene,Benzo[def]chrysene,Benzo[ghi]perylene,Indeno[1,2,3-cd]pyrene} | {Wood dust} | 89.2 | 73.3% | 13.3% | 10.9% | 11 |

### 192 - Manufacture of refined petroleum products

224 work situations with coexposures; 52 different chemical agents involved.

#### Prevalence of exposure by agent

#### Frequent itemset mining

953 identified. The 500 most frequent itemsets are listed below

| Itemset | N WS | % Support |
| --- | --- | --- |
| {Benzene,Toluene} | 142 | 63.4% |
| {Toluene,Xylene} | 115 | 51.3% |
| {Benzene,Xylene} | 113 | 50.4% |
| {Benzene,Toluene,Xylene} | 106 | 47.3% |
| {Ethylbenzene,Xylene} | 89 | 39.7% |
| {Benzene,Ethylbenzene} | 85 | 37.9% |
| {Ethylbenzene,Toluene} | 85 | 37.9% |
| {Ethylbenzene,Toluene,Xylene} | 84 | 37.5% |
| {Benzene,Ethylbenzene,Toluene} | 82 | 36.6% |
| {Benzene,Ethylbenzene,Toluene,Xylene} | 81 | 36.2% |
| {Benzene,Ethylbenzene,Xylene} | 81 | 36.2% |
| {Benzene,N-hexane} | 66 | 29.5% |
| {N-hexane,Toluene} | 65 | 29.0% |
| {Benzene,N-hexane,Toluene} | 60 | 26.8% |
| {N-hexane,Xylene} | 48 | 21.4% |
| {Pentane,Toluene} | 47 | 21.0% |
| {Heptane,Toluene} | 47 | 21.0% |
| {Benzene,N-hexane,Xylene} | 45 | 20.1% |
| {Cyclohexane,N-hexane} | 43 | 19.2% |
| {Benzene,N-hexane,Toluene,Xylene} | 43 | 19.2% |
| {N-hexane,Toluene,Xylene} | 43 | 19.2% |
| {Benzene,Pentane} | 42 | 18.8% |
| {N-hexane,Pentane} | 41 | 18.3% |
| {Benzene,Cyclohexane} | 40 | 17.9% |
| {Benzene,Heptane,Toluene} | 40 | 17.9% |
| {Benzene,Heptane} | 40 | 17.9% |
| {Heptane,Xylene} | 40 | 17.9% |
| {Benzene,Pentane,Toluene} | 39 | 17.4% |
| {Heptane,N-hexane} | 39 | 17.4% |
| {Benzene,Cyclohexane,N-hexane} | 38 | 17.0% |
| {Cyclohexane,Toluene} | 38 | 17.0% |
| {Benzene,Cyclohexane,Toluene} | 37 | 16.5% |
| {N-hexane,Pentane,Toluene} | 37 | 16.5% |
| {Heptane,Toluene,Xylene} | 37 | 16.5% |
| {1,2,4-Trimethylbenzene,Benzene} | 36 | 16.1% |
| {Cyclohexane,Xylene} | 36 | 16.1% |
| {Benzene,N-hexane,Pentane} | 36 | 16.1% |
| {Ethylbenzene,N-hexane} | 36 | 16.1% |
| {Benzene,Cyclohexane,N-hexane,Toluene} | 35 | 15.6% |
| {Cyclohexane,N-hexane,Toluene} | 35 | 15.6% |
| {Benzene,N-hexane,Pentane,Toluene} | 35 | 15.6% |
| {Benzene,Heptane,N-hexane,Toluene} | 35 | 15.6% |
| {Benzene,Heptane,N-hexane} | 35 | 15.6% |
| {Heptane,N-hexane,Toluene} | 35 | 15.6% |
| {Benzene,Heptane,Toluene,Xylene} | 35 | 15.6% |
| {Benzene,Heptane,Xylene} | 35 | 15.6% |
| {1,2,4-Trimethylbenzene,Ethylbenzene} | 34 | 15.2% |
| {Cyclohexane,N-hexane,Xylene} | 34 | 15.2% |
| {Benzene,Cyclohexane,Xylene} | 34 | 15.2% |
| {Benzene,Ethylbenzene,N-hexane} | 34 | 15.2% |
| {Ethylbenzene,N-hexane,Xylene} | 34 | 15.2% |
| {1,2,4-Trimethylbenzene,Toluene} | 33 | 14.7% |
| {Benzene,Cyclohexane,N-hexane,Xylene} | 33 | 14.7% |
| {Cyclohexane,Toluene,Xylene} | 33 | 14.7% |
| {Benzene,Ethylbenzene,N-hexane,Toluene} | 33 | 14.7% |
| {Ethylbenzene,N-hexane,Toluene} | 33 | 14.7% |
| {1,2,4-Trimethylbenzene,Benzene,Ethylbenzene} | 32 | 14.3% |
| {1,2,4-Trimethylbenzene,Benzene,Toluene} | 32 | 14.3% |
| {Benzene,Cyclohexane,Toluene,Xylene} | 32 | 14.3% |
| {Pentane,Xylene} | 32 | 14.3% |
| {Heptane,N-hexane,Xylene} | 32 | 14.3% |
| {Ethylbenzene,Heptane} | 32 | 14.3% |
| {Benzene,Ethylbenzene,N-hexane,Toluene,Xylene} | 32 | 14.3% |
| {Benzene,Ethylbenzene,N-hexane,Xylene} | 32 | 14.3% |
| {Ethylbenzene,N-hexane,Toluene,Xylene} | 32 | 14.3% |
| {1,2,4-Trimethylbenzene,Xylene} | 31 | 13.8% |
| {Benzene,Cyclohexane,N-hexane,Toluene,Xylene} | 31 | 13.8% |
| {Cyclohexane,N-hexane,Toluene,Xylene} | 31 | 13.8% |
| {Pentane,Toluene,Xylene} | 31 | 13.8% |
| {Benzene,Heptane,N-hexane,Toluene,Xylene} | 31 | 13.8% |
| {Benzene,Heptane,N-hexane,Xylene} | 31 | 13.8% |
| {Heptane,N-hexane,Toluene,Xylene} | 31 | 13.8% |
| {Benzene,Ethylbenzene,Heptane,Toluene} | 31 | 13.8% |
| {Benzene,Ethylbenzene,Heptane} | 31 | 13.8% |
| {Ethylbenzene,Heptane,Toluene} | 31 | 13.8% |
| {Ethylbenzene,Heptane,Xylene} | 31 | 13.8% |
| {1,2,4-Trimethylbenzene,Ethylbenzene,Toluene} | 30 | 13.4% |
| {1,2,4-Trimethylbenzene,Ethylbenzene,Xylene} | 30 | 13.4% |
| {1,2,4-Trimethylbenzene,Toluene,Xylene} | 30 | 13.4% |
| {Benzene,Pentane,Toluene,Xylene} | 30 | 13.4% |
| {Benzene,Pentane,Xylene} | 30 | 13.4% |
| {Heptane,Pentane} | 30 | 13.4% |
| {Benzene,Ethylbenzene,Heptane,Toluene,Xylene} | 30 | 13.4% |
| {Benzene,Ethylbenzene,Heptane,Xylene} | 30 | 13.4% |
| {Ethylbenzene,Heptane,Toluene,Xylene} | 30 | 13.4% |
| {1,2,4-Trimethylbenzene,Ethylbenzene,Toluene,Xylene} | 29 | 12.9% |
| {1,2,4-Trimethylbenzene,Benzene,Ethylbenzene,Toluene} | 29 | 12.9% |
| {1,2,4-Trimethylbenzene,Benzene,Toluene,Xylene} | 29 | 12.9% |
| {1,2,4-Trimethylbenzene,Benzene,Xylene} | 29 | 12.9% |
| {Cyclohexane,Ethylbenzene,Xylene} | 29 | 12.9% |
| {Cyclohexane,Ethylbenzene} | 29 | 12.9% |
| {Cyclohexane,Heptane} | 29 | 12.9% |
| {N-hexane,Pentane,Xylene} | 29 | 12.9% |
| {Ethylbenzene,Pentane} | 29 | 12.9% |
| {1,2,4-Trimethylbenzene,Benzene,Ethylbenzene,Toluene,Xylene} | 28 | 12.5% |
| {1,2,4-Trimethylbenzene,Benzene,Ethylbenzene,Xylene} | 28 | 12.5% |
| {Cyclohexane,Heptane,N-hexane} | 28 | 12.5% |
| {Cyclohexane,Ethylbenzene,Toluene,Xylene} | 28 | 12.5% |
| {Cyclohexane,Ethylbenzene,Toluene} | 28 | 12.5% |
| {Benzene,Heptane,Pentane,Toluene} | 28 | 12.5% |
| {Benzene,Heptane,Pentane} | 28 | 12.5% |
| {Heptane,Pentane,Toluene} | 28 | 12.5% |
| {Heptane,N-hexane,Pentane} | 28 | 12.5% |
| {Benzene,N-hexane,Pentane,Toluene,Xylene} | 28 | 12.5% |
| {Benzene,N-hexane,Pentane,Xylene} | 28 | 12.5% |
| {N-hexane,Pentane,Toluene,Xylene} | 28 | 12.5% |
| {Benzene,Ethylbenzene,Pentane} | 28 | 12.5% |
| {Benzene,Cyclohexane,Heptane,Toluene} | 27 | 12.1% |
| {Benzene,Cyclohexane,Heptane} | 27 | 12.1% |
| {Cyclohexane,Heptane,Toluene} | 27 | 12.1% |
| {Cyclohexane,Ethylbenzene,N-hexane,Xylene} | 27 | 12.1% |
| {Cyclohexane,Ethylbenzene,N-hexane} | 27 | 12.1% |
| {Benzene,Cyclohexane,Ethylbenzene,Toluene,Xylene} | 27 | 12.1% |
| {Benzene,Cyclohexane,Ethylbenzene,Xylene} | 27 | 12.1% |
| {Benzene,Cyclohexane,Ethylbenzene,Toluene} | 27 | 12.1% |
| {Benzene,Cyclohexane,Ethylbenzene} | 27 | 12.1% |
| {Benzene,Heptane,N-hexane,Pentane,Toluene} | 27 | 12.1% |
| {Benzene,Heptane,N-hexane,Pentane} | 27 | 12.1% |
| {Heptane,N-hexane,Pentane,Toluene} | 27 | 12.1% |
| {Heptane,Pentane,Xylene} | 27 | 12.1% |
| {Benzene,Ethylbenzene,Heptane,N-hexane,Toluene} | 27 | 12.1% |
| {Benzene,Ethylbenzene,Heptane,N-hexane} | 27 | 12.1% |
| {Ethylbenzene,Heptane,N-hexane,Toluene} | 27 | 12.1% |
| {Ethylbenzene,Heptane,N-hexane} | 27 | 12.1% |
| {1,2,4-Trimethylbenzene,Ethylbenzene,Heptane} | 26 | 11.6% |
| {1,2,4-Trimethylbenzene,Benzene,N-hexane} | 26 | 11.6% |
| {1,2,4-Trimethylbenzene,N-hexane} | 26 | 11.6% |
| {1,2,4-Trimethylbenzene,Heptane} | 26 | 11.6% |
| {Benzene,Cyclohexane,Heptane,N-hexane,Toluene} | 26 | 11.6% |
| {Benzene,Cyclohexane,Heptane,N-hexane} | 26 | 11.6% |
| {Cyclohexane,Heptane,N-hexane,Toluene} | 26 | 11.6% |
| {Benzene,Cyclohexane,Ethylbenzene,N-hexane,Toluene,Xylene} | 26 | 11.6% |
| {Benzene,Cyclohexane,Ethylbenzene,N-hexane,Xylene} | 26 | 11.6% |
| {Cyclohexane,Ethylbenzene,N-hexane,Toluene,Xylene} | 26 | 11.6% |
| {Benzene,Cyclohexane,Ethylbenzene,N-hexane,Toluene} | 26 | 11.6% |
| {Benzene,Cyclohexane,Ethylbenzene,N-hexane} | 26 | 11.6% |
| {Cyclohexane,Ethylbenzene,N-hexane,Toluene} | 26 | 11.6% |
| {Heptane,N-hexane,Pentane,Xylene} | 26 | 11.6% |
| {Benzene,Heptane,Pentane,Toluene,Xylene} | 26 | 11.6% |
| {Benzene,Heptane,Pentane,Xylene} | 26 | 11.6% |
| {Heptane,Pentane,Toluene,Xylene} | 26 | 11.6% |
| {Ethylbenzene,Pentane,Toluene,Xylene} | 26 | 11.6% |
| {Ethylbenzene,Pentane,Toluene} | 26 | 11.6% |
| {Ethylbenzene,Pentane,Xylene} | 26 | 11.6% |
| {Benzene,Ethylbenzene,Heptane,N-hexane,Toluene,Xylene} | 26 | 11.6% |
| {Benzene,Ethylbenzene,Heptane,N-hexane,Xylene} | 26 | 11.6% |
| {Ethylbenzene,Heptane,N-hexane,Toluene,Xylene} | 26 | 11.6% |
| {Ethylbenzene,Heptane,N-hexane,Xylene} | 26 | 11.6% |
| {Benzene,Chloroethylene} | 25 | 11.2% |
| {1,2,4-Trimethylbenzene,Benzene,Ethylbenzene,Heptane,Toluene} | 25 | 11.2% |
| {1,2,4-Trimethylbenzene,Benzene,Ethylbenzene,Heptane} | 25 | 11.2% |
| {1,2,4-Trimethylbenzene,Ethylbenzene,Heptane,Toluene} | 25 | 11.2% |
| {1,2,4-Trimethylbenzene,Ethylbenzene,Heptane,Xylene} | 25 | 11.2% |
| {1,2,4-Trimethylbenzene,Benzene,Heptane,Toluene} | 25 | 11.2% |
| {1,2,4-Trimethylbenzene,Benzene,Heptane} | 25 | 11.2% |
| {1,2,4-Trimethylbenzene,Heptane,Toluene} | 25 | 11.2% |
| {1,2,4-Trimethylbenzene,Heptane,Xylene} | 25 | 11.2% |
| {1,2,4-Trimethylbenzene,Benzene,Ethylbenzene,N-hexane} | 25 | 11.2% |
| {1,2,4-Trimethylbenzene,Benzene,N-hexane,Toluene} | 25 | 11.2% |
| {1,2,4-Trimethylbenzene,N-hexane,Toluene} | 25 | 11.2% |
| {1,2,4-Trimethylbenzene,Ethylbenzene,N-hexane} | 25 | 11.2% |
| {Benzene,Cyclohexane,Heptane,Toluene,Xylene} | 25 | 11.2% |
| {Benzene,Cyclohexane,Heptane,Xylene} | 25 | 11.2% |
| {Cyclohexane,Heptane,Toluene,Xylene} | 25 | 11.2% |
| {Cyclohexane,Heptane,Xylene} | 25 | 11.2% |
| {Benzene,Heptane,N-hexane,Pentane,Toluene,Xylene} | 25 | 11.2% |
| {Benzene,Heptane,N-hexane,Pentane,Xylene} | 25 | 11.2% |
| {Heptane,N-hexane,Pentane,Toluene,Xylene} | 25 | 11.2% |
| {Benzene,Ethylbenzene,Pentane,Toluene,Xylene} | 25 | 11.2% |
| {Benzene,Ethylbenzene,Pentane,Xylene} | 25 | 11.2% |
| {Benzene,Ethylbenzene,Pentane,Toluene} | 25 | 11.2% |
| {1,2,4-Trimethylbenzene,Benzene,Ethylbenzene,Heptane,Toluene,Xylene} | 24 | 10.7% |
| {1,2,4-Trimethylbenzene,Benzene,Ethylbenzene,Heptane,Xylene} | 24 | 10.7% |
| {1,2,4-Trimethylbenzene,Ethylbenzene,Heptane,Toluene,Xylene} | 24 | 10.7% |
| {1,2,4-Trimethylbenzene,Benzene,Heptane,Toluene,Xylene} | 24 | 10.7% |
| {1,2,4-Trimethylbenzene,Benzene,Heptane,Xylene} | 24 | 10.7% |
| {1,2,4-Trimethylbenzene,Heptane,Toluene,Xylene} | 24 | 10.7% |
| {1,2,4-Trimethylbenzene,Benzene,Ethylbenzene,N-hexane,Toluene} | 24 | 10.7% |
| {1,2,4-Trimethylbenzene,Ethylbenzene,N-hexane,Toluene} | 24 | 10.7% |
| {1,2,4-Trimethylbenzene,Benzene,N-hexane,Toluene,Xylene} | 24 | 10.7% |
| {1,2,4-Trimethylbenzene,Benzene,N-hexane,Xylene} | 24 | 10.7% |
| {1,2,4-Trimethylbenzene,N-hexane,Toluene,Xylene} | 24 | 10.7% |
| {1,2,4-Trimethylbenzene,N-hexane,Xylene} | 24 | 10.7% |
| {Cyclohexane,Pentane,Toluene} | 24 | 10.7% |
| {Benzene,Cyclohexane,Heptane,N-hexane,Toluene,Xylene} | 24 | 10.7% |
| {Benzene,Cyclohexane,Heptane,N-hexane,Xylene} | 24 | 10.7% |
| {Cyclohexane,Heptane,N-hexane,Toluene,Xylene} | 24 | 10.7% |
| {Cyclohexane,Heptane,N-hexane,Xylene} | 24 | 10.7% |
| {Cyclohexane,Pentane} | 24 | 10.7% |
| {Benzene,Ethylbenzene,N-hexane,Pentane} | 24 | 10.7% |
| {Ethylbenzene,N-hexane,Pentane} | 24 | 10.7% |
| {1,2,4-Trimethylbenzene,Benzene,Pentane} | 23 | 10.3% |
| {1,2,4-Trimethylbenzene,Benzene,Ethylbenzene,N-hexane,Toluene,Xylene} | 23 | 10.3% |
| {1,2,4-Trimethylbenzene,Benzene,Ethylbenzene,N-hexane,Xylene} | 23 | 10.3% |
| {1,2,4-Trimethylbenzene,Ethylbenzene,N-hexane,Toluene,Xylene} | 23 | 10.3% |
| {1,2,4-Trimethylbenzene,Ethylbenzene,N-hexane,Xylene} | 23 | 10.3% |
| {1,2,4-Trimethylbenzene,Pentane} | 23 | 10.3% |
| {Benzene,Cyclohexane,Pentane,Toluene} | 23 | 10.3% |
| {Benzene,Cyclohexane,Pentane} | 23 | 10.3% |
| {Benzene,Cyclohexane,Ethylbenzene,Heptane,Toluene,Xylene} | 23 | 10.3% |
| {Benzene,Cyclohexane,Ethylbenzene,Heptane,Xylene} | 23 | 10.3% |
| {Cyclohexane,Ethylbenzene,Heptane,Toluene,Xylene} | 23 | 10.3% |
| {Benzene,Cyclohexane,Ethylbenzene,Heptane,Toluene} | 23 | 10.3% |
| {Benzene,Cyclohexane,Ethylbenzene,Heptane} | 23 | 10.3% |
| {Cyclohexane,Ethylbenzene,Heptane,Toluene} | 23 | 10.3% |
| {Cyclohexane,Ethylbenzene,Heptane,Xylene} | 23 | 10.3% |
| {Cyclohexane,Ethylbenzene,Heptane} | 23 | 10.3% |
| {Benzene,Ethylbenzene,N-hexane,Pentane,Toluene,Xylene} | 23 | 10.3% |
| {Benzene,Ethylbenzene,N-hexane,Pentane,Xylene} | 23 | 10.3% |
| {Ethylbenzene,N-hexane,Pentane,Toluene,Xylene} | 23 | 10.3% |
| {Benzene,Ethylbenzene,N-hexane,Pentane,Toluene} | 23 | 10.3% |
| {Ethylbenzene,N-hexane,Pentane,Toluene} | 23 | 10.3% |
| {Ethylbenzene,N-hexane,Pentane,Xylene} | 23 | 10.3% |
| {1,2,4-Trimethylbenzene,Benzene,Ethylbenzene,Pentane} | 22 | 9.8% |
| {1,2,4-Trimethylbenzene,Ethylbenzene,Pentane} | 22 | 9.8% |
| {1,2,4-Trimethylbenzene,Benzene,Ethylbenzene,Heptane,N-hexane,Toluene} | 22 | 9.8% |
| {1,2,4-Trimethylbenzene,Benzene,Ethylbenzene,Heptane,N-hexane} | 22 | 9.8% |
| {1,2,4-Trimethylbenzene,Ethylbenzene,Heptane,N-hexane,Toluene} | 22 | 9.8% |
| {1,2,4-Trimethylbenzene,Benzene,Heptane,N-hexane,Toluene} | 22 | 9.8% |
| {1,2,4-Trimethylbenzene,Benzene,Heptane,N-hexane} | 22 | 9.8% |
| {1,2,4-Trimethylbenzene,Heptane,N-hexane,Toluene} | 22 | 9.8% |
| {1,2,4-Trimethylbenzene,Ethylbenzene,Heptane,N-hexane} | 22 | 9.8% |
| {1,2,4-Trimethylbenzene,Heptane,N-hexane} | 22 | 9.8% |
| {Cyclohexane,Pentane,Toluene,Xylene} | 22 | 9.8% |
| {Cyclohexane,Pentane,Xylene} | 22 | 9.8% |
| {Benzene,Cyclohexane,Ethylbenzene,Heptane,N-hexane,Toluene,Xylene} | 22 | 9.8% |
| {Benzene,Cyclohexane,Ethylbenzene,Heptane,N-hexane,Xylene} | 22 | 9.8% |
| {Cyclohexane,Ethylbenzene,Heptane,N-hexane,Toluene,Xylene} | 22 | 9.8% |
| {Benzene,Cyclohexane,Ethylbenzene,Heptane,N-hexane,Toluene} | 22 | 9.8% |
| {Benzene,Cyclohexane,Ethylbenzene,Heptane,N-hexane} | 22 | 9.8% |
| {Cyclohexane,Ethylbenzene,Heptane,N-hexane,Toluene} | 22 | 9.8% |
| {Cyclohexane,Ethylbenzene,Heptane,N-hexane,Xylene} | 22 | 9.8% |
| {Cyclohexane,Ethylbenzene,Heptane,N-hexane} | 22 | 9.8% |
| {Benzene,Ethylbenzene,Heptane,Pentane,Toluene,Xylene} | 22 | 9.8% |
| {Benzene,Ethylbenzene,Heptane,Pentane,Xylene} | 22 | 9.8% |
| {Ethylbenzene,Heptane,Pentane,Toluene,Xylene} | 22 | 9.8% |
| {Benzene,Ethylbenzene,Heptane,Pentane,Toluene} | 22 | 9.8% |
| {Benzene,Ethylbenzene,Heptane,Pentane} | 22 | 9.8% |
| {Ethylbenzene,Heptane,Pentane,Toluene} | 22 | 9.8% |
| {Ethylbenzene,Heptane,Pentane,Xylene} | 22 | 9.8% |
| {Ethylbenzene,Heptane,Pentane} | 22 | 9.8% |
| {Chloroethylene,Xylene} | 21 | 9.4% |
| {1,2,4-Trimethylbenzene,Benzene,Ethylbenzene,Heptane,N-hexane,Toluene,Xylene} | 21 | 9.4% |
| {1,2,4-Trimethylbenzene,Benzene,Ethylbenzene,Heptane,N-hexane,Xylene} | 21 | 9.4% |
| {1,2,4-Trimethylbenzene,Ethylbenzene,Heptane,N-hexane,Toluene,Xylene} | 21 | 9.4% |
| {1,2,4-Trimethylbenzene,Ethylbenzene,Heptane,N-hexane,Xylene} | 21 | 9.4% |
| {1,2,4-Trimethylbenzene,Benzene,Heptane,N-hexane,Toluene,Xylene} | 21 | 9.4% |
| {1,2,4-Trimethylbenzene,Benzene,Heptane,N-hexane,Xylene} | 21 | 9.4% |
| {1,2,4-Trimethylbenzene,Heptane,N-hexane,Toluene,Xylene} | 21 | 9.4% |
| {1,2,4-Trimethylbenzene,Heptane,N-hexane,Xylene} | 21 | 9.4% |
| {Benzene,Cyclohexane,Heptane,Pentane,Toluene} | 21 | 9.4% |
| {Benzene,Cyclohexane,Heptane,Pentane} | 21 | 9.4% |
| {Cyclohexane,Heptane,Pentane,Toluene} | 21 | 9.4% |
| {Benzene,Cyclohexane,N-hexane,Pentane,Toluene} | 21 | 9.4% |
| {Benzene,Cyclohexane,N-hexane,Pentane} | 21 | 9.4% |
| {Cyclohexane,N-hexane,Pentane,Toluene} | 21 | 9.4% |
| {Cyclohexane,Ethylbenzene,Pentane,Toluene,Xylene} | 21 | 9.4% |
| {Cyclohexane,Ethylbenzene,Pentane,Toluene} | 21 | 9.4% |
| {Cyclohexane,Ethylbenzene,Pentane,Xylene} | 21 | 9.4% |
| {Benzene,Cyclohexane,Pentane,Toluene,Xylene} | 21 | 9.4% |
| {Benzene,Cyclohexane,Pentane,Xylene} | 21 | 9.4% |
| {Cyclohexane,Ethylbenzene,Pentane} | 21 | 9.4% |
| {Cyclohexane,N-hexane,Pentane} | 21 | 9.4% |
| {Cyclohexane,Heptane,Pentane} | 21 | 9.4% |
| {Benzene,Ethylbenzene,Heptane,N-hexane,Pentane,Toluene,Xylene} | 21 | 9.4% |
| {Benzene,Ethylbenzene,Heptane,N-hexane,Pentane,Xylene} | 21 | 9.4% |
| {Ethylbenzene,Heptane,N-hexane,Pentane,Toluene,Xylene} | 21 | 9.4% |
| {Benzene,Ethylbenzene,Heptane,N-hexane,Pentane,Toluene} | 21 | 9.4% |
| {Benzene,Ethylbenzene,Heptane,N-hexane,Pentane} | 21 | 9.4% |
| {Ethylbenzene,Heptane,N-hexane,Pentane,Toluene} | 21 | 9.4% |
| {Ethylbenzene,Heptane,N-hexane,Pentane,Xylene} | 21 | 9.4% |
| {Ethylbenzene,Heptane,N-hexane,Pentane} | 21 | 9.4% |
| {1,2,4-Trimethylbenzene,Benzene,Pentane,Toluene} | 20 | 8.9% |
| {1,2,4-Trimethylbenzene,Pentane,Toluene} | 20 | 8.9% |
| {Benzene,Cyclohexane,Heptane,N-hexane,Pentane,Toluene} | 20 | 8.9% |
| {Benzene,Cyclohexane,Heptane,N-hexane,Pentane} | 20 | 8.9% |
| {Cyclohexane,Heptane,N-hexane,Pentane,Toluene} | 20 | 8.9% |
| {Benzene,Cyclohexane,Heptane,Pentane,Toluene,Xylene} | 20 | 8.9% |
| {Benzene,Cyclohexane,Heptane,Pentane,Xylene} | 20 | 8.9% |
| {Cyclohexane,Heptane,Pentane,Toluene,Xylene} | 20 | 8.9% |
| {Cyclohexane,Heptane,Pentane,Xylene} | 20 | 8.9% |
| {Cyclohexane,Heptane,N-hexane,Pentane} | 20 | 8.9% |
| {Benzene,Cyclohexane,N-hexane,Pentane,Toluene,Xylene} | 20 | 8.9% |
| {Benzene,Cyclohexane,N-hexane,Pentane,Xylene} | 20 | 8.9% |
| {Cyclohexane,N-hexane,Pentane,Toluene,Xylene} | 20 | 8.9% |
| {Cyclohexane,N-hexane,Pentane,Xylene} | 20 | 8.9% |
| {Benzene,Cyclohexane,Ethylbenzene,Pentane,Toluene,Xylene} | 20 | 8.9% |
| {Benzene,Cyclohexane,Ethylbenzene,Pentane,Xylene} | 20 | 8.9% |
| {Benzene,Cyclohexane,Ethylbenzene,Pentane,Toluene} | 20 | 8.9% |
| {Benzene,Cyclohexane,Ethylbenzene,Pentane} | 20 | 8.9% |
| {Benzene,Styrene} | 19 | 8.5% |
| {Benzene,Chloroethylene,Xylene} | 19 | 8.5% |
| {1,2,4-Trimethylbenzene,Benzene,Ethylbenzene,N-hexane,Pentane} | 19 | 8.5% |
| {1,2,4-Trimethylbenzene,Benzene,N-hexane,Pentane} | 19 | 8.5% |
| {1,2,4-Trimethylbenzene,Ethylbenzene,N-hexane,Pentane} | 19 | 8.5% |
| {1,2,4-Trimethylbenzene,Benzene,Ethylbenzene,Pentane,Toluene,Xylene} | 19 | 8.5% |
| {1,2,4-Trimethylbenzene,Benzene,Ethylbenzene,Pentane,Xylene} | 19 | 8.5% |
| {1,2,4-Trimethylbenzene,Ethylbenzene,Pentane,Toluene,Xylene} | 19 | 8.5% |
| {1,2,4-Trimethylbenzene,Benzene,Ethylbenzene,Pentane,Toluene} | 19 | 8.5% |
| {1,2,4-Trimethylbenzene,Ethylbenzene,Pentane,Toluene} | 19 | 8.5% |
| {1,2,4-Trimethylbenzene,Ethylbenzene,Pentane,Xylene} | 19 | 8.5% |
| {1,2,4-Trimethylbenzene,Benzene,Pentane,Toluene,Xylene} | 19 | 8.5% |
| {1,2,4-Trimethylbenzene,Benzene,Pentane,Xylene} | 19 | 8.5% |
| {1,2,4-Trimethylbenzene,Pentane,Toluene,Xylene} | 19 | 8.5% |
| {1,2,4-Trimethylbenzene,Pentane,Xylene} | 19 | 8.5% |
| {1,2,4-Trimethylbenzene,N-hexane,Pentane} | 19 | 8.5% |
| {Benzene,Cyclohexane,Heptane,N-hexane,Pentane,Toluene,Xylene} | 19 | 8.5% |
| {Benzene,Cyclohexane,Heptane,N-hexane,Pentane,Xylene} | 19 | 8.5% |
| {Cyclohexane,Heptane,N-hexane,Pentane,Toluene,Xylene} | 19 | 8.5% |
| {Cyclohexane,Heptane,N-hexane,Pentane,Xylene} | 19 | 8.5% |
| {Benzene,Cyclohexane,Ethylbenzene,Heptane,Pentane,Toluene,Xylene} | 19 | 8.5% |
| {Benzene,Cyclohexane,Ethylbenzene,Heptane,Pentane,Xylene} | 19 | 8.5% |
| {Cyclohexane,Ethylbenzene,Heptane,Pentane,Toluene,Xylene} | 19 | 8.5% |
| {Benzene,Cyclohexane,Ethylbenzene,Heptane,Pentane,Toluene} | 19 | 8.5% |
| {Benzene,Cyclohexane,Ethylbenzene,Heptane,Pentane} | 19 | 8.5% |
| {Cyclohexane,Ethylbenzene,Heptane,Pentane,Toluene} | 19 | 8.5% |
| {Cyclohexane,Ethylbenzene,Heptane,Pentane,Xylene} | 19 | 8.5% |
| {Cyclohexane,Ethylbenzene,Heptane,Pentane} | 19 | 8.5% |
| {Benzene,Cyclohexane,Ethylbenzene,N-hexane,Pentane,Toluene,Xylene} | 19 | 8.5% |
| {Benzene,Cyclohexane,Ethylbenzene,N-hexane,Pentane,Xylene} | 19 | 8.5% |
| {Cyclohexane,Ethylbenzene,N-hexane,Pentane,Toluene,Xylene} | 19 | 8.5% |
| {Benzene,Cyclohexane,Ethylbenzene,N-hexane,Pentane,Toluene} | 19 | 8.5% |
| {Benzene,Cyclohexane,Ethylbenzene,N-hexane,Pentane} | 19 | 8.5% |
| {Cyclohexane,Ethylbenzene,N-hexane,Pentane,Toluene} | 19 | 8.5% |
| {Cyclohexane,Ethylbenzene,N-hexane,Pentane,Xylene} | 19 | 8.5% |
| {Cyclohexane,Ethylbenzene,N-hexane,Pentane} | 19 | 8.5% |
| {1,2,3-Trimethylbenzene,Benzene} | 18 | 8.0% |
| {1,2,3-Trimethylbenzene,1,2,4-Trimethylbenzene} | 18 | 8.0% |
| {1,2,4-Trimethylbenzene,Benzene,Cyclohexane,Ethylbenzene,Heptane,Toluene,Xylene} | 18 | 8.0% |
| {1,2,4-Trimethylbenzene,Benzene,Cyclohexane,Ethylbenzene,Heptane,Xylene} | 18 | 8.0% |
| {1,2,4-Trimethylbenzene,Cyclohexane,Ethylbenzene,Heptane,Toluene,Xylene} | 18 | 8.0% |
| {1,2,4-Trimethylbenzene,Benzene,Cyclohexane,Ethylbenzene,Heptane,Toluene} | 18 | 8.0% |
| {1,2,4-Trimethylbenzene,Benzene,Cyclohexane,Ethylbenzene,Heptane} | 18 | 8.0% |
| {1,2,4-Trimethylbenzene,Cyclohexane,Ethylbenzene,Heptane,Toluene} | 18 | 8.0% |
| {1,2,4-Trimethylbenzene,Cyclohexane,Ethylbenzene,Heptane,Xylene} | 18 | 8.0% |
| {1,2,4-Trimethylbenzene,Benzene,Cyclohexane,Heptane,Toluene,Xylene} | 18 | 8.0% |
| {1,2,4-Trimethylbenzene,Benzene,Cyclohexane,Heptane,Xylene} | 18 | 8.0% |
| {1,2,4-Trimethylbenzene,Cyclohexane,Heptane,Toluene,Xylene} | 18 | 8.0% |
| {1,2,4-Trimethylbenzene,Benzene,Cyclohexane,Heptane,Toluene} | 18 | 8.0% |
| {1,2,4-Trimethylbenzene,Benzene,Cyclohexane,Heptane} | 18 | 8.0% |
| {1,2,4-Trimethylbenzene,Cyclohexane,Heptane,Toluene} | 18 | 8.0% |
| {1,2,4-Trimethylbenzene,Cyclohexane,Heptane,Xylene} | 18 | 8.0% |
| {1,2,4-Trimethylbenzene,Cyclohexane,Ethylbenzene,Heptane} | 18 | 8.0% |
| {1,2,4-Trimethylbenzene,Benzene,Cyclohexane,Ethylbenzene,Toluene,Xylene} | 18 | 8.0% |
| {1,2,4-Trimethylbenzene,Benzene,Cyclohexane,Ethylbenzene,Xylene} | 18 | 8.0% |
| {1,2,4-Trimethylbenzene,Cyclohexane,Ethylbenzene,Toluene,Xylene} | 18 | 8.0% |
| {1,2,4-Trimethylbenzene,Benzene,Cyclohexane,Ethylbenzene,Toluene} | 18 | 8.0% |
| {1,2,4-Trimethylbenzene,Benzene,Cyclohexane,Ethylbenzene} | 18 | 8.0% |
| {1,2,4-Trimethylbenzene,Cyclohexane,Ethylbenzene,Toluene} | 18 | 8.0% |
| {1,2,4-Trimethylbenzene,Cyclohexane,Ethylbenzene,Xylene} | 18 | 8.0% |
| {1,2,4-Trimethylbenzene,Benzene,Cyclohexane,Toluene,Xylene} | 18 | 8.0% |
| {1,2,4-Trimethylbenzene,Benzene,Cyclohexane,Xylene} | 18 | 8.0% |
| {1,2,4-Trimethylbenzene,Cyclohexane,Toluene,Xylene} | 18 | 8.0% |
| {1,2,4-Trimethylbenzene,Benzene,Cyclohexane,Toluene} | 18 | 8.0% |
| {1,2,4-Trimethylbenzene,Benzene,Cyclohexane} | 18 | 8.0% |
| {1,2,4-Trimethylbenzene,Cyclohexane,Toluene} | 18 | 8.0% |
| {1,2,4-Trimethylbenzene,Cyclohexane,Xylene} | 18 | 8.0% |
| {1,2,4-Trimethylbenzene,Cyclohexane,Ethylbenzene} | 18 | 8.0% |
| {1,2,4-Trimethylbenzene,Cyclohexane,Heptane} | 18 | 8.0% |
| {1,2,4-Trimethylbenzene,Benzene,Ethylbenzene,Heptane,Pentane,Toluene,Xylene} | 18 | 8.0% |
| {1,2,4-Trimethylbenzene,Benzene,Ethylbenzene,Heptane,Pentane,Xylene} | 18 | 8.0% |
| {1,2,4-Trimethylbenzene,Ethylbenzene,Heptane,Pentane,Toluene,Xylene} | 18 | 8.0% |
| {1,2,4-Trimethylbenzene,Benzene,Ethylbenzene,Heptane,Pentane,Toluene} | 18 | 8.0% |
| {1,2,4-Trimethylbenzene,Benzene,Ethylbenzene,Heptane,Pentane} | 18 | 8.0% |
| {1,2,4-Trimethylbenzene,Ethylbenzene,Heptane,Pentane,Toluene} | 18 | 8.0% |
| {1,2,4-Trimethylbenzene,Ethylbenzene,Heptane,Pentane,Xylene} | 18 | 8.0% |
| {1,2,4-Trimethylbenzene,Benzene,Heptane,Pentane,Toluene,Xylene} | 18 | 8.0% |
| {1,2,4-Trimethylbenzene,Benzene,Heptane,Pentane,Xylene} | 18 | 8.0% |
| {1,2,4-Trimethylbenzene,Heptane,Pentane,Toluene,Xylene} | 18 | 8.0% |
| {1,2,4-Trimethylbenzene,Benzene,Heptane,Pentane,Toluene} | 18 | 8.0% |
| {1,2,4-Trimethylbenzene,Benzene,Heptane,Pentane} | 18 | 8.0% |
| {1,2,4-Trimethylbenzene,Heptane,Pentane,Toluene} | 18 | 8.0% |
| {1,2,4-Trimethylbenzene,Heptane,Pentane,Xylene} | 18 | 8.0% |
| {1,2,4-Trimethylbenzene,Ethylbenzene,Heptane,Pentane} | 18 | 8.0% |
| {1,2,4-Trimethylbenzene,Benzene,Ethylbenzene,N-hexane,Pentane,Toluene,Xylene} | 18 | 8.0% |
| {1,2,4-Trimethylbenzene,Benzene,Ethylbenzene,N-hexane,Pentane,Xylene} | 18 | 8.0% |
| {1,2,4-Trimethylbenzene,Ethylbenzene,N-hexane,Pentane,Toluene,Xylene} | 18 | 8.0% |
| {1,2,4-Trimethylbenzene,Benzene,Ethylbenzene,N-hexane,Pentane,Toluene} | 18 | 8.0% |
| {1,2,4-Trimethylbenzene,Ethylbenzene,N-hexane,Pentane,Toluene} | 18 | 8.0% |
| {1,2,4-Trimethylbenzene,Ethylbenzene,N-hexane,Pentane,Xylene} | 18 | 8.0% |
| {1,2,4-Trimethylbenzene,Benzene,N-hexane,Pentane,Toluene,Xylene} | 18 | 8.0% |
| {1,2,4-Trimethylbenzene,Benzene,N-hexane,Pentane,Xylene} | 18 | 8.0% |
| {1,2,4-Trimethylbenzene,N-hexane,Pentane,Toluene,Xylene} | 18 | 8.0% |
| {1,2,4-Trimethylbenzene,Benzene,N-hexane,Pentane,Toluene} | 18 | 8.0% |
| {1,2,4-Trimethylbenzene,N-hexane,Pentane,Toluene} | 18 | 8.0% |
| {1,2,4-Trimethylbenzene,N-hexane,Pentane,Xylene} | 18 | 8.0% |
| {1,2,4-Trimethylbenzene,Heptane,Pentane} | 18 | 8.0% |
| {1,2,4-Trimethylbenzene,Cyclohexane} | 18 | 8.0% |
| {Benzene,Cyclohexane,Ethylbenzene,Heptane,N-hexane,Pentane,Toluene,Xylene} | 18 | 8.0% |
| {Benzene,Cyclohexane,Ethylbenzene,Heptane,N-hexane,Pentane,Xylene} | 18 | 8.0% |
| {Cyclohexane,Ethylbenzene,Heptane,N-hexane,Pentane,Toluene,Xylene} | 18 | 8.0% |
| {Benzene,Cyclohexane,Ethylbenzene,Heptane,N-hexane,Pentane,Toluene} | 18 | 8.0% |
| {Benzene,Cyclohexane,Ethylbenzene,Heptane,N-hexane,Pentane} | 18 | 8.0% |
| {Cyclohexane,Ethylbenzene,Heptane,N-hexane,Pentane,Toluene} | 18 | 8.0% |
| {Cyclohexane,Ethylbenzene,Heptane,N-hexane,Pentane,Xylene} | 18 | 8.0% |
| {Cyclohexane,Ethylbenzene,Heptane,N-hexane,Pentane} | 18 | 8.0% |
| {Benzene,Chloroethylene,Toluene} | 17 | 7.6% |
| {Chloroethylene,Toluene} | 17 | 7.6% |
| {1,2,4-Trimethylbenzene,Mesitylene} | 17 | 7.6% |
| {1,2,3-Trimethylbenzene,1,2,4-Trimethylbenzene,Benzene} | 17 | 7.6% |
| {1,2,3-Trimethylbenzene,1,2,4-Trimethylbenzene,Ethylbenzene} | 17 | 7.6% |
| {1,2,3-Trimethylbenzene,Ethylbenzene} | 17 | 7.6% |
| {1,2,4-Trimethylbenzene,Benzene,Cyclohexane,Ethylbenzene,Heptane,N-hexane,Toluene,Xylene} | 17 | 7.6% |
| {1,2,4-Trimethylbenzene,Benzene,Cyclohexane,Ethylbenzene,Heptane,N-hexane,Xylene} | 17 | 7.6% |
| {1,2,4-Trimethylbenzene,Cyclohexane,Ethylbenzene,Heptane,N-hexane,Toluene,Xylene} | 17 | 7.6% |
| {1,2,4-Trimethylbenzene,Benzene,Cyclohexane,Ethylbenzene,Heptane,N-hexane,Toluene} | 17 | 7.6% |
| {1,2,4-Trimethylbenzene,Benzene,Cyclohexane,Ethylbenzene,Heptane,N-hexane} | 17 | 7.6% |
| {1,2,4-Trimethylbenzene,Cyclohexane,Ethylbenzene,Heptane,N-hexane,Toluene} | 17 | 7.6% |
| {1,2,4-Trimethylbenzene,Cyclohexane,Ethylbenzene,Heptane,N-hexane,Xylene} | 17 | 7.6% |
| {1,2,4-Trimethylbenzene,Benzene,Cyclohexane,Heptane,N-hexane,Toluene,Xylene} | 17 | 7.6% |
| {1,2,4-Trimethylbenzene,Benzene,Cyclohexane,Heptane,N-hexane,Xylene} | 17 | 7.6% |
| {1,2,4-Trimethylbenzene,Cyclohexane,Heptane,N-hexane,Toluene,Xylene} | 17 | 7.6% |
| {1,2,4-Trimethylbenzene,Benzene,Cyclohexane,Heptane,N-hexane,Toluene} | 17 | 7.6% |
| {1,2,4-Trimethylbenzene,Benzene,Cyclohexane,Heptane,N-hexane} | 17 | 7.6% |
| {1,2,4-Trimethylbenzene,Cyclohexane,Heptane,N-hexane,Toluene} | 17 | 7.6% |
| {1,2,4-Trimethylbenzene,Cyclohexane,Heptane,N-hexane,Xylene} | 17 | 7.6% |
| {1,2,4-Trimethylbenzene,Cyclohexane,Ethylbenzene,Heptane,N-hexane} | 17 | 7.6% |
| {1,2,4-Trimethylbenzene,Cyclohexane,Heptane,N-hexane} | 17 | 7.6% |
| {1,2,4-Trimethylbenzene,Benzene,Cyclohexane,Ethylbenzene,N-hexane,Toluene,Xylene} | 17 | 7.6% |
| {1,2,4-Trimethylbenzene,Benzene,Cyclohexane,Ethylbenzene,N-hexane,Xylene} | 17 | 7.6% |
| {1,2,4-Trimethylbenzene,Cyclohexane,Ethylbenzene,N-hexane,Toluene,Xylene} | 17 | 7.6% |
| {1,2,4-Trimethylbenzene,Benzene,Cyclohexane,Ethylbenzene,N-hexane,Toluene} | 17 | 7.6% |
| {1,2,4-Trimethylbenzene,Benzene,Cyclohexane,Ethylbenzene,N-hexane} | 17 | 7.6% |
| {1,2,4-Trimethylbenzene,Cyclohexane,Ethylbenzene,N-hexane,Toluene} | 17 | 7.6% |
| {1,2,4-Trimethylbenzene,Cyclohexane,Ethylbenzene,N-hexane,Xylene} | 17 | 7.6% |
| {1,2,4-Trimethylbenzene,Benzene,Cyclohexane,N-hexane,Toluene,Xylene} | 17 | 7.6% |
| {1,2,4-Trimethylbenzene,Benzene,Cyclohexane,N-hexane,Xylene} | 17 | 7.6% |
| {1,2,4-Trimethylbenzene,Cyclohexane,N-hexane,Toluene,Xylene} | 17 | 7.6% |
| {1,2,4-Trimethylbenzene,Benzene,Cyclohexane,N-hexane,Toluene} | 17 | 7.6% |
| {1,2,4-Trimethylbenzene,Benzene,Cyclohexane,N-hexane} | 17 | 7.6% |
| {1,2,4-Trimethylbenzene,Cyclohexane,N-hexane,Toluene} | 17 | 7.6% |
| {1,2,4-Trimethylbenzene,Cyclohexane,N-hexane,Xylene} | 17 | 7.6% |
| {1,2,4-Trimethylbenzene,Cyclohexane,Ethylbenzene,N-hexane} | 17 | 7.6% |
| {1,2,4-Trimethylbenzene,Cyclohexane,N-hexane} | 17 | 7.6% |
| {1,2,4-Trimethylbenzene,Benzene,Ethylbenzene,Heptane,N-hexane,Pentane,Toluene,Xylene} | 17 | 7.6% |
| {1,2,4-Trimethylbenzene,Benzene,Ethylbenzene,Heptane,N-hexane,Pentane,Xylene} | 17 | 7.6% |
| {1,2,4-Trimethylbenzene,Ethylbenzene,Heptane,N-hexane,Pentane,Toluene,Xylene} | 17 | 7.6% |
| {1,2,4-Trimethylbenzene,Benzene,Ethylbenzene,Heptane,N-hexane,Pentane,Toluene} | 17 | 7.6% |
| {1,2,4-Trimethylbenzene,Benzene,Ethylbenzene,Heptane,N-hexane,Pentane} | 17 | 7.6% |
| {1,2,4-Trimethylbenzene,Ethylbenzene,Heptane,N-hexane,Pentane,Toluene} | 17 | 7.6% |
| {1,2,4-Trimethylbenzene,Ethylbenzene,Heptane,N-hexane,Pentane,Xylene} | 17 | 7.6% |
| {1,2,4-Trimethylbenzene,Benzene,Heptane,N-hexane,Pentane,Toluene,Xylene} | 17 | 7.6% |
| {1,2,4-Trimethylbenzene,Benzene,Heptane,N-hexane,Pentane,Xylene} | 17 | 7.6% |
| {1,2,4-Trimethylbenzene,Heptane,N-hexane,Pentane,Toluene,Xylene} | 17 | 7.6% |
| {1,2,4-Trimethylbenzene,Benzene,Heptane,N-hexane,Pentane,Toluene} | 17 | 7.6% |
| {1,2,4-Trimethylbenzene,Benzene,Heptane,N-hexane,Pentane} | 17 | 7.6% |
| {1,2,4-Trimethylbenzene,Heptane,N-hexane,Pentane,Toluene} | 17 | 7.6% |
| {1,2,4-Trimethylbenzene,Heptane,N-hexane,Pentane,Xylene} | 17 | 7.6% |
| {1,2,4-Trimethylbenzene,Ethylbenzene,Heptane,N-hexane,Pentane} | 17 | 7.6% |
| {1,2,4-Trimethylbenzene,Heptane,N-hexane,Pentane} | 17 | 7.6% |
| {Styrene,Xylene} | 16 | 7.1% |
| {1,2,4-Trimethylbenzene,Benzene,Mesitylene} | 16 | 7.1% |
| {1,2,4-Trimethylbenzene,Ethylbenzene,Mesitylene} | 16 | 7.1% |
| {Benzene,Mesitylene} | 16 | 7.1% |
| {Ethylbenzene,Mesitylene} | 16 | 7.1% |
| {1,2,3-Trimethylbenzene,1,2,4-Trimethylbenzene,Benzene,Ethylbenzene} | 16 | 7.1% |
| {1,2,3-Trimethylbenzene,Benzene,Ethylbenzene} | 16 | 7.1% |
| {1,2,3-Trimethylbenzene,Benzene,Toluene} | 16 | 7.1% |
| {1,2,3-Trimethylbenzene,Toluene} | 16 | 7.1% |
| {Acetonitrile,Benzene} | 15 | 6.7% |
| {Benzene,Styrene,Xylene} | 15 | 6.7% |
| {Chloroethylene,Styrene} | 15 | 6.7% |
| {2-Methylbutane,Toluene} | 15 | 6.7% |
| {Acetone,Benzene} | 15 | 6.7% |
| {Chloroethylene,Ethylbenzene,Xylene} | 15 | 6.7% |
| {Benzene,Chloroethylene,Toluene,Xylene} | 15 | 6.7% |
| {Chloroethylene,Toluene,Xylene} | 15 | 6.7% |
| {Chloroethylene,Ethylbenzene} | 15 | 6.7% |
| {1,2,4-Trimethylbenzene,Benzene,Ethylbenzene,Mesitylene} | 15 | 6.7% |
| {Benzene,Ethylbenzene,Mesitylene} | 15 | 6.7% |
| {1,2,3-Trimethylbenzene,1,2,4-Trimethylbenzene,Benzene,Toluene} | 15 | 6.7% |
| {1,2,3-Trimethylbenzene,1,2,4-Trimethylbenzene,Toluene} | 15 | 6.7% |
| {1,2,3-Trimethylbenzene,Xylene} | 15 | 6.7% |
| {1,2,4-Trimethylbenzene,Benzene,Cyclohexane,Ethylbenzene,Heptane,Pentane,Toluene,Xylene} | 15 | 6.7% |
| {1,2,4-Trimethylbenzene,Benzene,Cyclohexane,Ethylbenzene,Heptane,Pentane,Xylene} | 15 | 6.7% |
| {1,2,4-Trimethylbenzene,Cyclohexane,Ethylbenzene,Heptane,Pentane,Toluene,Xylene} | 15 | 6.7% |
| {1,2,4-Trimethylbenzene,Benzene,Cyclohexane,Ethylbenzene,Heptane,Pentane,Toluene} | 15 | 6.7% |
| {1,2,4-Trimethylbenzene,Benzene,Cyclohexane,Ethylbenzene,Heptane,Pentane} | 15 | 6.7% |
| {1,2,4-Trimethylbenzene,Cyclohexane,Ethylbenzene,Heptane,Pentane,Toluene} | 15 | 6.7% |
| {1,2,4-Trimethylbenzene,Cyclohexane,Ethylbenzene,Heptane,Pentane,Xylene} | 15 | 6.7% |
| {1,2,4-Trimethylbenzene,Benzene,Cyclohexane,Heptane,Pentane,Toluene,Xylene} | 15 | 6.7% |
| {1,2,4-Trimethylbenzene,Benzene,Cyclohexane,Heptane,Pentane,Xylene} | 15 | 6.7% |
| {1,2,4-Trimethylbenzene,Cyclohexane,Heptane,Pentane,Toluene,Xylene} | 15 | 6.7% |
| {1,2,4-Trimethylbenzene,Benzene,Cyclohexane,Heptane,Pentane,Toluene} | 15 | 6.7% |
| {1,2,4-Trimethylbenzene,Benzene,Cyclohexane,Heptane,Pentane} | 15 | 6.7% |
| {1,2,4-Trimethylbenzene,Cyclohexane,Heptane,Pentane,Toluene} | 15 | 6.7% |
| {1,2,4-Trimethylbenzene,Cyclohexane,Heptane,Pentane,Xylene} | 15 | 6.7% |
| {1,2,4-Trimethylbenzene,Cyclohexane,Ethylbenzene,Heptane,Pentane} | 15 | 6.7% |
| {1,2,4-Trimethylbenzene,Benzene,Cyclohexane,Ethylbenzene,Pentane,Toluene,Xylene} | 15 | 6.7% |
| {1,2,4-Trimethylbenzene,Benzene,Cyclohexane,Ethylbenzene,Pentane,Xylene} | 15 | 6.7% |
| {1,2,4-Trimethylbenzene,Cyclohexane,Ethylbenzene,Pentane,Toluene,Xylene} | 15 | 6.7% |
| {1,2,4-Trimethylbenzene,Benzene,Cyclohexane,Ethylbenzene,Pentane,Toluene} | 15 | 6.7% |
| {1,2,4-Trimethylbenzene,Benzene,Cyclohexane,Ethylbenzene,Pentane} | 15 | 6.7% |
| {1,2,4-Trimethylbenzene,Cyclohexane,Ethylbenzene,Pentane,Toluene} | 15 | 6.7% |
| {1,2,4-Trimethylbenzene,Cyclohexane,Ethylbenzene,Pentane,Xylene} | 15 | 6.7% |
| {1,2,4-Trimethylbenzene,Benzene,Cyclohexane,Pentane,Toluene,Xylene} | 15 | 6.7% |
| {1,2,4-Trimethylbenzene,Benzene,Cyclohexane,Pentane,Xylene} | 15 | 6.7% |
| {1,2,4-Trimethylbenzene,Cyclohexane,Pentane,Toluene,Xylene} | 15 | 6.7% |
| {1,2,4-Trimethylbenzene,Benzene,Cyclohexane,Pentane,Toluene} | 15 | 6.7% |
| {1,2,4-Trimethylbenzene,Benzene,Cyclohexane,Pentane} | 15 | 6.7% |

#### Association rules mining

##### Association rules with the largest confidence among WS exposed to a minimum of 2 agents. Minimum level of support = 0.1%, minimum confidence 0.1.

| Antecedent | Consequent | % Confidence | % Support | N WS |
| --- | --- | --- | --- | --- |
| {Phenol} | {Xylene} | 100.0% | 5.4% | 12 |
| {Acetonitrile} | {Benzene} | 100.0% | 6.7% | 15 |
| {2-Methylbutane} | {Toluene} | 100.0% | 6.7% | 15 |
| {Mesitylene} | {1,2,4-Trimethylbenzene} | 100.0% | 7.6% | 17 |
| {Styrene,Toluene} | {Benzene} | 100.0% | 5.4% | 12 |
| {1,2,4-Trimethylbenzene,2-Methylbutane} | {Ethylbenzene} | 100.0% | 5.4% | 12 |
| {2-Methylbutane,Ethylbenzene} | {1,2,4-Trimethylbenzene} | 100.0% | 5.4% | 12 |
| {1,2,4-Trimethylbenzene,2-Methylbutane} | {Toluene} | 100.0% | 5.4% | 12 |
| {2-Methylbutane,Ethylbenzene} | {Toluene} | 100.0% | 5.4% | 12 |
| {2-Methylbutane,Benzene} | {Toluene} | 100.0% | 5.4% | 12 |
| {Acetone,N-hexane} | {Toluene} | 100.0% | 5.4% | 12 |
| {Acetone,N-hexane} | {Benzene} | 100.0% | 5.4% | 12 |
| {Acetone,Xylene} | {Toluene} | 100.0% | 5.4% | 12 |
| {Acetone,Xylene} | {Benzene} | 100.0% | 5.4% | 12 |
| {Acetone,Toluene} | {Benzene} | 100.0% | 5.8% | 13 |
| {Chloroethylene,Ethylbenzene} | {Xylene} | 100.0% | 6.7% | 15 |
| {Chloroethylene,Toluene} | {Benzene} | 100.0% | 7.6% | 17 |
| {1,2,3-Trimethylbenzene,Mesitylene} | {1,2,4-Trimethylbenzene} | 100.0% | 6.2% | 14 |
| {1,2,3-Trimethylbenzene,Mesitylene} | {Benzene} | 100.0% | 6.2% | 14 |
| {Ethylbenzene,Mesitylene} | {1,2,4-Trimethylbenzene} | 100.0% | 7.1% | 16 |
| {Mesitylene,Xylene} | {1,2,4-Trimethylbenzene} | 100.0% | 5.4% | 12 |
| {Mesitylene,Toluene} | {1,2,4-Trimethylbenzene} | 100.0% | 6.2% | 14 |
| {Benzene,Mesitylene} | {1,2,4-Trimethylbenzene} | 100.0% | 7.1% | 16 |
| {Mesitylene,Xylene} | {Ethylbenzene} | 100.0% | 5.4% | 12 |
| {Mesitylene,Xylene} | {Toluene} | 100.0% | 5.4% | 12 |
| {1,2,3-Trimethylbenzene,Heptane} | {1,2,4-Trimethylbenzene} | 100.0% | 6.2% | 14 |
| {1,2,3-Trimethylbenzene,N-hexane} | {1,2,4-Trimethylbenzene} | 100.0% | 5.8% | 13 |
| {1,2,3-Trimethylbenzene,Ethylbenzene} | {1,2,4-Trimethylbenzene} | 100.0% | 7.6% | 17 |
| {1,2,3-Trimethylbenzene,Heptane} | {Ethylbenzene} | 100.0% | 6.2% | 14 |
| {1,2,3-Trimethylbenzene,N-hexane} | {Ethylbenzene} | 100.0% | 5.8% | 13 |
| {1,2,3-Trimethylbenzene,N-hexane} | {Benzene} | 100.0% | 5.8% | 13 |
| {1,2,3-Trimethylbenzene,Toluene} | {Benzene} | 100.0% | 7.1% | 16 |
| {1,2,4-Trimethylbenzene,Cyclohexane} | {Heptane} | 100.0% | 8.0% | 18 |
| {1,2,4-Trimethylbenzene,Cyclohexane} | {Ethylbenzene} | 100.0% | 8.0% | 18 |
| {1,2,4-Trimethylbenzene,Cyclohexane} | {Xylene} | 100.0% | 8.0% | 18 |
| {1,2,4-Trimethylbenzene,Cyclohexane} | {Toluene} | 100.0% | 8.0% | 18 |
| {1,2,4-Trimethylbenzene,Cyclohexane} | {Benzene} | 100.0% | 8.0% | 18 |
| {1,2,4-Trimethylbenzene,Pentane} | {Benzene} | 100.0% | 10.3% | 23 |
| {1,2,4-Trimethylbenzene,Heptane} | {Ethylbenzene} | 100.0% | 11.6% | 26 |
| {1,2,4-Trimethylbenzene,N-hexane} | {Benzene} | 100.0% | 11.6% | 26 |
| {Cyclohexane,Pentane} | {Toluene} | 100.0% | 10.7% | 24 |
| {Cyclohexane,Ethylbenzene} | {Xylene} | 100.0% | 12.9% | 29 |
| {Benzene,Heptane} | {Toluene} | 100.0% | 17.9% | 40 |
| {1,2,4-Trimethylbenzene,2-Methylbutane,Ethylbenzene} | {Toluene} | 100.0% | 5.4% | 12 |
| {1,2,4-Trimethylbenzene,2-Methylbutane,Toluene} | {Ethylbenzene} | 100.0% | 5.4% | 12 |
| {2-Methylbutane,Ethylbenzene,Toluene} | {1,2,4-Trimethylbenzene} | 100.0% | 5.4% | 12 |
| {Acetone,N-hexane,Toluene} | {Benzene} | 100.0% | 5.4% | 12 |
| {Acetone,Benzene,N-hexane} | {Toluene} | 100.0% | 5.4% | 12 |
| {Acetone,Toluene,Xylene} | {Benzene} | 100.0% | 5.4% | 12 |
| {Acetone,Benzene,Xylene} | {Toluene} | 100.0% | 5.4% | 12 |
| {Chloroethylene,Ethylbenzene,Toluene} | {Xylene} | 100.0% | 6.2% | 14 |
| {Benzene,Chloroethylene,Ethylbenzene} | {Xylene} | 100.0% | 6.2% | 14 |
| {Chloroethylene,Ethylbenzene,Toluene} | {Benzene} | 100.0% | 6.2% | 14 |
| {Benzene,Chloroethylene,Ethylbenzene} | {Toluene} | 100.0% | 6.2% | 14 |
| {Chloroethylene,Toluene,Xylene} | {Benzene} | 100.0% | 6.7% | 15 |
| {1,2,3-Trimethylbenzene,Ethylbenzene,Mesitylene} | {1,2,4-Trimethylbenzene} | 100.0% | 5.8% | 13 |
| {1,2,3-Trimethylbenzene,Mesitylene,Toluene} | {1,2,4-Trimethylbenzene} | 100.0% | 5.4% | 12 |
| {1,2,3-Trimethylbenzene,1,2,4-Trimethylbenzene,Mesitylene} | {Benzene} | 100.0% | 6.2% | 14 |
| {1,2,3-Trimethylbenzene,Benzene,Mesitylene} | {1,2,4-Trimethylbenzene} | 100.0% | 6.2% | 14 |
| {1,2,3-Trimethylbenzene,Ethylbenzene,Mesitylene} | {Benzene} | 100.0% | 5.8% | 13 |
| {1,2,3-Trimethylbenzene,Mesitylene,Toluene} | {Benzene} | 100.0% | 5.4% | 12 |
| {1,2,4-Trimethylbenzene,Mesitylene,Xylene} | {Ethylbenzene} | 100.0% | 5.4% | 12 |
| {Ethylbenzene,Mesitylene,Xylene} | {1,2,4-Trimethylbenzene} | 100.0% | 5.4% | 12 |
| {Ethylbenzene,Mesitylene,Toluene} | {1,2,4-Trimethylbenzene} | 100.0% | 5.8% | 13 |
| {Benzene,Ethylbenzene,Mesitylene} | {1,2,4-Trimethylbenzene} | 100.0% | 6.7% | 15 |
| {1,2,4-Trimethylbenzene,Mesitylene,Xylene} | {Toluene} | 100.0% | 5.4% | 12 |
| {Mesitylene,Toluene,Xylene} | {1,2,4-Trimethylbenzene} | 100.0% | 5.4% | 12 |
| {Benzene,Mesitylene,Toluene} | {1,2,4-Trimethylbenzene} | 100.0% | 5.8% | 13 |
| {Ethylbenzene,Mesitylene,Xylene} | {Toluene} | 100.0% | 5.4% | 12 |
| {Mesitylene,Toluene,Xylene} | {Ethylbenzene} | 100.0% | 5.4% | 12 |
| {1,2,3-Trimethylbenzene,Heptane,N-hexane} | {1,2,4-Trimethylbenzene} | 100.0% | 5.4% | 12 |
| {1,2,3-Trimethylbenzene,1,2,4-Trimethylbenzene,Heptane} | {Ethylbenzene} | 100.0% | 6.2% | 14 |
| {1,2,3-Trimethylbenzene,Ethylbenzene,Heptane} | {1,2,4-Trimethylbenzene} | 100.0% | 6.2% | 14 |
| {1,2,3-Trimethylbenzene,Heptane,Xylene} | {1,2,4-Trimethylbenzene} | 100.0% | 5.8% | 13 |
| {1,2,3-Trimethylbenzene,Heptane,Toluene} | {1,2,4-Trimethylbenzene} | 100.0% | 5.8% | 13 |
| {1,2,3-Trimethylbenzene,Benzene,Heptane} | {1,2,4-Trimethylbenzene} | 100.0% | 5.8% | 13 |
| {1,2,3-Trimethylbenzene,1,2,4-Trimethylbenzene,N-hexane} | {Ethylbenzene} | 100.0% | 5.8% | 13 |
| {1,2,3-Trimethylbenzene,Ethylbenzene,N-hexane} | {1,2,4-Trimethylbenzene} | 100.0% | 5.8% | 13 |
| {1,2,3-Trimethylbenzene,N-hexane,Toluene} | {1,2,4-Trimethylbenzene} | 100.0% | 5.4% | 12 |
| {1,2,3-Trimethylbenzene,1,2,4-Trimethylbenzene,N-hexane} | {Benzene} | 100.0% | 5.8% | 13 |
| {1,2,3-Trimethylbenzene,Benzene,N-hexane} | {1,2,4-Trimethylbenzene} | 100.0% | 5.8% | 13 |
| {1,2,3-Trimethylbenzene,1,2,4-Trimethylbenzene,Xylene} | {Ethylbenzene} | 100.0% | 6.2% | 14 |
| {1,2,3-Trimethylbenzene,Ethylbenzene,Xylene} | {1,2,4-Trimethylbenzene} | 100.0% | 6.2% | 14 |
| {1,2,3-Trimethylbenzene,Ethylbenzene,Toluene} | {1,2,4-Trimethylbenzene} | 100.0% | 6.2% | 14 |
| {1,2,3-Trimethylbenzene,Benzene,Ethylbenzene} | {1,2,4-Trimethylbenzene} | 100.0% | 7.1% | 16 |
| {1,2,3-Trimethylbenzene,1,2,4-Trimethylbenzene,Toluene} | {Benzene} | 100.0% | 6.7% | 15 |
| {1,2,3-Trimethylbenzene,Heptane,N-hexane} | {Ethylbenzene} | 100.0% | 5.4% | 12 |
| {1,2,3-Trimethylbenzene,Heptane,N-hexane} | {Toluene} | 100.0% | 5.4% | 12 |
| {1,2,3-Trimethylbenzene,N-hexane,Toluene} | {Heptane} | 100.0% | 5.4% | 12 |
| {1,2,3-Trimethylbenzene,Heptane,N-hexane} | {Benzene} | 100.0% | 5.4% | 12 |
| {1,2,3-Trimethylbenzene,Heptane,Xylene} | {Ethylbenzene} | 100.0% | 5.8% | 13 |
| {1,2,3-Trimethylbenzene,Heptane,Toluene} | {Ethylbenzene} | 100.0% | 5.8% | 13 |
| {1,2,3-Trimethylbenzene,Benzene,Heptane} | {Ethylbenzene} | 100.0% | 5.8% | 13 |
| {1,2,3-Trimethylbenzene,Heptane,Toluene} | {Benzene} | 100.0% | 5.8% | 13 |
| {1,2,3-Trimethylbenzene,Benzene,Heptane} | {Toluene} | 100.0% | 5.8% | 13 |
| {1,2,3-Trimethylbenzene,N-hexane,Toluene} | {Ethylbenzene} | 100.0% | 5.4% | 12 |
| {1,2,3-Trimethylbenzene,Ethylbenzene,N-hexane} | {Benzene} | 100.0% | 5.8% | 13 |
| {1,2,3-Trimethylbenzene,Benzene,N-hexane} | {Ethylbenzene} | 100.0% | 5.8% | 13 |
| {1,2,3-Trimethylbenzene,N-hexane,Toluene} | {Benzene} | 100.0% | 5.4% | 12 |
| {1,2,3-Trimethylbenzene,Ethylbenzene,Toluene} | {Benzene} | 100.0% | 6.2% | 14 |
| {1,2,3-Trimethylbenzene,Toluene,Xylene} | {Benzene} | 100.0% | 6.2% | 14 |
| {1,2,3-Trimethylbenzene,Benzene,Xylene} | {Toluene} | 100.0% | 6.2% | 14 |
| {1,2,4-Trimethylbenzene,Cyclohexane,Pentane} | {Heptane} | 100.0% | 6.7% | 15 |
| {1,2,4-Trimethylbenzene,Cyclohexane,Pentane} | {Ethylbenzene} | 100.0% | 6.7% | 15 |
| {1,2,4-Trimethylbenzene,Cyclohexane,Pentane} | {Xylene} | 100.0% | 6.7% | 15 |
| {1,2,4-Trimethylbenzene,Cyclohexane,Pentane} | {Toluene} | 100.0% | 6.7% | 15 |
| {1,2,4-Trimethylbenzene,Cyclohexane,Pentane} | {Benzene} | 100.0% | 6.7% | 15 |
| {1,2,4-Trimethylbenzene,Cyclohexane,N-hexane} | {Heptane} | 100.0% | 7.6% | 17 |
| {1,2,4-Trimethylbenzene,Cyclohexane,Heptane} | {Ethylbenzene} | 100.0% | 8.0% | 18 |
| {1,2,4-Trimethylbenzene,Cyclohexane,Ethylbenzene} | {Heptane} | 100.0% | 8.0% | 18 |
| {1,2,4-Trimethylbenzene,Cyclohexane,Heptane} | {Xylene} | 100.0% | 8.0% | 18 |
| {1,2,4-Trimethylbenzene,Cyclohexane,Xylene} | {Heptane} | 100.0% | 8.0% | 18 |
| {1,2,4-Trimethylbenzene,Cyclohexane,Heptane} | {Toluene} | 100.0% | 8.0% | 18 |
| {1,2,4-Trimethylbenzene,Cyclohexane,Toluene} | {Heptane} | 100.0% | 8.0% | 18 |
| {1,2,4-Trimethylbenzene,Cyclohexane,Heptane} | {Benzene} | 100.0% | 8.0% | 18 |
| {1,2,4-Trimethylbenzene,Benzene,Cyclohexane} | {Heptane} | 100.0% | 8.0% | 18 |
| {1,2,4-Trimethylbenzene,Cyclohexane,N-hexane} | {Ethylbenzene} | 100.0% | 7.6% | 17 |
| {1,2,4-Trimethylbenzene,Cyclohexane,N-hexane} | {Xylene} | 100.0% | 7.6% | 17 |
| {1,2,4-Trimethylbenzene,Cyclohexane,N-hexane} | {Toluene} | 100.0% | 7.6% | 17 |
| {1,2,4-Trimethylbenzene,Cyclohexane,N-hexane} | {Benzene} | 100.0% | 7.6% | 17 |
| {1,2,4-Trimethylbenzene,Cyclohexane,Ethylbenzene} | {Xylene} | 100.0% | 8.0% | 18 |
| {1,2,4-Trimethylbenzene,Cyclohexane,Xylene} | {Ethylbenzene} | 100.0% | 8.0% | 18 |
| {1,2,4-Trimethylbenzene,Cyclohexane,Ethylbenzene} | {Toluene} | 100.0% | 8.0% | 18 |
| {1,2,4-Trimethylbenzene,Cyclohexane,Toluene} | {Ethylbenzene} | 100.0% | 8.0% | 18 |
| {1,2,4-Trimethylbenzene,Cyclohexane,Ethylbenzene} | {Benzene} | 100.0% | 8.0% | 18 |
| {1,2,4-Trimethylbenzene,Benzene,Cyclohexane} | {Ethylbenzene} | 100.0% | 8.0% | 18 |
| {1,2,4-Trimethylbenzene,Cyclohexane,Xylene} | {Toluene} | 100.0% | 8.0% | 18 |
| {1,2,4-Trimethylbenzene,Cyclohexane,Toluene} | {Xylene} | 100.0% | 8.0% | 18 |
| {1,2,4-Trimethylbenzene,Cyclohexane,Xylene} | {Benzene} | 100.0% | 8.0% | 18 |
| {1,2,4-Trimethylbenzene,Benzene,Cyclohexane} | {Xylene} | 100.0% | 8.0% | 18 |
| {1,2,4-Trimethylbenzene,Cyclohexane,Toluene} | {Benzene} | 100.0% | 8.0% | 18 |
| {1,2,4-Trimethylbenzene,Benzene,Cyclohexane} | {Toluene} | 100.0% | 8.0% | 18 |
| {1,2,4-Trimethylbenzene,Heptane,Pentane} | {Ethylbenzene} | 100.0% | 8.0% | 18 |
| {1,2,4-Trimethylbenzene,Heptane,Pentane} | {Xylene} | 100.0% | 8.0% | 18 |
| {1,2,4-Trimethylbenzene,Heptane,Pentane} | {Toluene} | 100.0% | 8.0% | 18 |
| {1,2,4-Trimethylbenzene,Heptane,Pentane} | {Benzene} | 100.0% | 8.0% | 18 |
| {1,2,4-Trimethylbenzene,N-hexane,Pentane} | {Ethylbenzene} | 100.0% | 8.5% | 19 |
| {1,2,4-Trimethylbenzene,N-hexane,Pentane} | {Benzene} | 100.0% | 8.5% | 19 |
| {1,2,4-Trimethylbenzene,Pentane,Xylene} | {Ethylbenzene} | 100.0% | 8.5% | 19 |
| {1,2,4-Trimethylbenzene,Ethylbenzene,Pentane} | {Benzene} | 100.0% | 9.8% | 22 |
| {1,2,4-Trimethylbenzene,Pentane,Xylene} | {Toluene} | 100.0% | 8.5% | 19 |
| {1,2,4-Trimethylbenzene,Pentane,Xylene} | {Benzene} | 100.0% | 8.5% | 19 |
| {1,2,4-Trimethylbenzene,Pentane,Toluene} | {Benzene} | 100.0% | 8.9% | 20 |
| {1,2,4-Trimethylbenzene,Heptane,N-hexane} | {Ethylbenzene} | 100.0% | 9.8% | 22 |
| {1,2,4-Trimethylbenzene,Heptane,N-hexane} | {Toluene} | 100.0% | 9.8% | 22 |
| {1,2,4-Trimethylbenzene,Heptane,N-hexane} | {Benzene} | 100.0% | 9.8% | 22 |
| {1,2,4-Trimethylbenzene,Heptane,Xylene} | {Ethylbenzene} | 100.0% | 11.2% | 25 |
| {1,2,4-Trimethylbenzene,Heptane,Toluene} | {Ethylbenzene} | 100.0% | 11.2% | 25 |
| {1,2,4-Trimethylbenzene,Benzene,Heptane} | {Ethylbenzene} | 100.0% | 11.2% | 25 |
| {1,2,4-Trimethylbenzene,Heptane,Toluene} | {Benzene} | 100.0% | 11.2% | 25 |
| {1,2,4-Trimethylbenzene,Benzene,Heptane} | {Toluene} | 100.0% | 11.2% | 25 |
| {1,2,4-Trimethylbenzene,Ethylbenzene,N-hexane} | {Benzene} | 100.0% | 11.2% | 25 |
| {1,2,4-Trimethylbenzene,N-hexane,Xylene} | {Toluene} | 100.0% | 10.7% | 24 |
| {1,2,4-Trimethylbenzene,N-hexane,Xylene} | {Benzene} | 100.0% | 10.7% | 24 |
| {1,2,4-Trimethylbenzene,N-hexane,Toluene} | {Benzene} | 100.0% | 11.2% | 25 |
| {1,2,4-Trimethylbenzene,Benzene,Xylene} | {Toluene} | 100.0% | 12.9% | 29 |
| {Cyclohexane,Heptane,Pentane} | {Toluene} | 100.0% | 9.4% | 21 |
| {Cyclohexane,Heptane,Pentane} | {Benzene} | 100.0% | 9.4% | 21 |
| {Cyclohexane,N-hexane,Pentane} | {Toluene} | 100.0% | 9.4% | 21 |
| {Cyclohexane,N-hexane,Pentane} | {Benzene} | 100.0% | 9.4% | 21 |
| {Cyclohexane,Ethylbenzene,Pentane} | {Xylene} | 100.0% | 9.4% | 21 |
| {Cyclohexane,Ethylbenzene,Pentane} | {Toluene} | 100.0% | 9.4% | 21 |
| {Cyclohexane,Pentane,Xylene} | {Toluene} | 100.0% | 9.8% | 22 |
| {Benzene,Cyclohexane,Pentane} | {Toluene} | 100.0% | 10.3% | 23 |
| {Cyclohexane,Ethylbenzene,Heptane} | {Xylene} | 100.0% | 10.3% | 23 |
| {Cyclohexane,Ethylbenzene,Heptane} | {Toluene} | 100.0% | 10.3% | 23 |
| {Cyclohexane,Ethylbenzene,Heptane} | {Benzene} | 100.0% | 10.3% | 23 |
| {Cyclohexane,Heptane,Xylene} | {Toluene} | 100.0% | 11.2% | 25 |
| {Cyclohexane,Heptane,Xylene} | {Benzene} | 100.0% | 11.2% | 25 |
| {Cyclohexane,Heptane,Toluene} | {Benzene} | 100.0% | 12.1% | 27 |
| {Benzene,Cyclohexane,Heptane} | {Toluene} | 100.0% | 12.1% | 27 |
| {Cyclohexane,Ethylbenzene,N-hexane} | {Xylene} | 100.0% | 12.1% | 27 |
| {Cyclohexane,N-hexane,Toluene} | {Benzene} | 100.0% | 15.6% | 35 |
| {Cyclohexane,Ethylbenzene,Toluene} | {Xylene} | 100.0% | 12.5% | 28 |
| {Benzene,Cyclohexane,Ethylbenzene} | {Xylene} | 100.0% | 12.1% | 27 |
| {Benzene,Cyclohexane,Ethylbenzene} | {Toluene} | 100.0% | 12.1% | 27 |
| {Ethylbenzene,Heptane,Pentane} | {Xylene} | 100.0% | 9.8% | 22 |
| {Ethylbenzene,Heptane,Pentane} | {Toluene} | 100.0% | 9.8% | 22 |
| {Ethylbenzene,Heptane,Pentane} | {Benzene} | 100.0% | 9.8% | 22 |
| {Heptane,Pentane,Toluene} | {Benzene} | 100.0% | 12.5% | 28 |
| {Benzene,Heptane,Pentane} | {Toluene} | 100.0% | 12.5% | 28 |
| {Ethylbenzene,N-hexane,Pentane} | {Benzene} | 100.0% | 10.7% | 24 |
| {Ethylbenzene,Pentane,Xylene} | {Toluene} | 100.0% | 11.6% | 26 |
| {Ethylbenzene,Pentane,Toluene} | {Xylene} | 100.0% | 11.6% | 26 |
| {Benzene,Pentane,Xylene} | {Toluene} | 100.0% | 13.4% | 30 |
| {Ethylbenzene,Heptane,N-hexane} | {Toluene} | 100.0% | 12.1% | 27 |
| {Ethylbenzene,Heptane,N-hexane} | {Benzene} | 100.0% | 12.1% | 27 |
| {Heptane,N-hexane,Toluene} | {Benzene} | 100.0% | 15.6% | 35 |
| {Benzene,Heptane,N-hexane} | {Toluene} | 100.0% | 15.6% | 35 |
| {Ethylbenzene,Heptane,Toluene} | {Benzene} | 100.0% | 13.8% | 31 |
| {Benzene,Ethylbenzene,Heptane} | {Toluene} | 100.0% | 13.8% | 31 |
| {Benzene,Heptane,Xylene} | {Toluene} | 100.0% | 15.6% | 35 |
| {Ethylbenzene,N-hexane,Toluene} | {Benzene} | 100.0% | 14.7% | 33 |
| {N-hexane,Toluene,Xylene} | {Benzene} | 100.0% | 19.2% | 43 |
| {Benzene,Ethylbenzene,Xylene} | {Toluene} | 100.0% | 36.2% | 81 |
| {Chloroethylene,Ethylbenzene,Toluene,Xylene} | {Benzene} | 100.0% | 6.2% | 14 |
| {Benzene,Chloroethylene,Ethylbenzene,Xylene} | {Toluene} | 100.0% | 6.2% | 14 |
| {Benzene,Chloroethylene,Ethylbenzene,Toluene} | {Xylene} | 100.0% | 6.2% | 14 |
| {1,2,3-Trimethylbenzene,1,2,4-Trimethylbenzene,Ethylbenzene,Mesitylene} | {Benzene} | 100.0% | 5.8% | 13 |
| {1,2,3-Trimethylbenzene,Benzene,Ethylbenzene,Mesitylene} | {1,2,4-Trimethylbenzene} | 100.0% | 5.8% | 13 |
| {1,2,3-Trimethylbenzene,1,2,4-Trimethylbenzene,Mesitylene,Toluene} | {Benzene} | 100.0% | 5.4% | 12 |
| {1,2,3-Trimethylbenzene,Benzene,Mesitylene,Toluene} | {1,2,4-Trimethylbenzene} | 100.0% | 5.4% | 12 |
| {1,2,4-Trimethylbenzene,Ethylbenzene,Mesitylene,Xylene} | {Toluene} | 100.0% | 5.4% | 12 |
| {1,2,4-Trimethylbenzene,Mesitylene,Toluene,Xylene} | {Ethylbenzene} | 100.0% | 5.4% | 12 |
| {Ethylbenzene,Mesitylene,Toluene,Xylene} | {1,2,4-Trimethylbenzene} | 100.0% | 5.4% | 12 |
| {Benzene,Ethylbenzene,Mesitylene,Toluene} | {1,2,4-Trimethylbenzene} | 100.0% | 5.4% | 12 |
| {1,2,3-Trimethylbenzene,1,2,4-Trimethylbenzene,Heptane,N-hexane} | {Ethylbenzene} | 100.0% | 5.4% | 12 |
| {1,2,3-Trimethylbenzene,Ethylbenzene,Heptane,N-hexane} | {1,2,4-Trimethylbenzene} | 100.0% | 5.4% | 12 |
| {1,2,3-Trimethylbenzene,1,2,4-Trimethylbenzene,Heptane,N-hexane} | {Toluene} | 100.0% | 5.4% | 12 |
| {1,2,3-Trimethylbenzene,1,2,4-Trimethylbenzene,N-hexane,Toluene} | {Heptane} | 100.0% | 5.4% | 12 |
| {1,2,3-Trimethylbenzene,Heptane,N-hexane,Toluene} | {1,2,4-Trimethylbenzene} | 100.0% | 5.4% | 12 |
| {1,2,3-Trimethylbenzene,1,2,4-Trimethylbenzene,Heptane,N-hexane} | {Benzene} | 100.0% | 5.4% | 12 |
| {1,2,3-Trimethylbenzene,Benzene,Heptane,N-hexane} | {1,2,4-Trimethylbenzene} | 100.0% | 5.4% | 12 |
| {1,2,3-Trimethylbenzene,1,2,4-Trimethylbenzene,Heptane,Xylene} | {Ethylbenzene} | 100.0% | 5.8% | 13 |
| {1,2,3-Trimethylbenzene,Ethylbenzene,Heptane,Xylene} | {1,2,4-Trimethylbenzene} | 100.0% | 5.8% | 13 |
| {1,2,3-Trimethylbenzene,1,2,4-Trimethylbenzene,Heptane,Toluene} | {Ethylbenzene} | 100.0% | 5.8% | 13 |
| {1,2,3-Trimethylbenzene,Ethylbenzene,Heptane,Toluene} | {1,2,4-Trimethylbenzene} | 100.0% | 5.8% | 13 |
| {1,2,3-Trimethylbenzene,1,2,4-Trimethylbenzene,Benzene,Heptane} | {Ethylbenzene} | 100.0% | 5.8% | 13 |
| {1,2,3-Trimethylbenzene,Benzene,Ethylbenzene,Heptane} | {1,2,4-Trimethylbenzene} | 100.0% | 5.8% | 13 |
| {1,2,3-Trimethylbenzene,Heptane,Toluene,Xylene} | {1,2,4-Trimethylbenzene} | 100.0% | 5.4% | 12 |
| {1,2,3-Trimethylbenzene,Benzene,Heptane,Xylene} | {1,2,4-Trimethylbenzene} | 100.0% | 5.4% | 12 |
| {1,2,3-Trimethylbenzene,1,2,4-Trimethylbenzene,Heptane,Toluene} | {Benzene} | 100.0% | 5.8% | 13 |
| {1,2,3-Trimethylbenzene,1,2,4-Trimethylbenzene,Benzene,Heptane} | {Toluene} | 100.0% | 5.8% | 13 |
| {1,2,3-Trimethylbenzene,Benzene,Heptane,Toluene} | {1,2,4-Trimethylbenzene} | 100.0% | 5.8% | 13 |
| {1,2,3-Trimethylbenzene,1,2,4-Trimethylbenzene,N-hexane,Toluene} | {Ethylbenzene} | 100.0% | 5.4% | 12 |
| {1,2,3-Trimethylbenzene,Ethylbenzene,N-hexane,Toluene} | {1,2,4-Trimethylbenzene} | 100.0% | 5.4% | 12 |
| {1,2,3-Trimethylbenzene,1,2,4-Trimethylbenzene,Ethylbenzene,N-hexane} | {Benzene} | 100.0% | 5.8% | 13 |
| {1,2,3-Trimethylbenzene,1,2,4-Trimethylbenzene,Benzene,N-hexane} | {Ethylbenzene} | 100.0% | 5.8% | 13 |
| {1,2,3-Trimethylbenzene,Benzene,Ethylbenzene,N-hexane} | {1,2,4-Trimethylbenzene} | 100.0% | 5.8% | 13 |
| {1,2,3-Trimethylbenzene,1,2,4-Trimethylbenzene,N-hexane,Toluene} | {Benzene} | 100.0% | 5.4% | 12 |
| {1,2,3-Trimethylbenzene,Benzene,N-hexane,Toluene} | {1,2,4-Trimethylbenzene} | 100.0% | 5.4% | 12 |
| {1,2,3-Trimethylbenzene,1,2,4-Trimethylbenzene,Toluene,Xylene} | {Ethylbenzene} | 100.0% | 5.8% | 13 |
| {1,2,3-Trimethylbenzene,Ethylbenzene,Toluene,Xylene} | {1,2,4-Trimethylbenzene} | 100.0% | 5.8% | 13 |
| {1,2,3-Trimethylbenzene,1,2,4-Trimethylbenzene,Benzene,Xylene} | {Ethylbenzene} | 100.0% | 5.8% | 13 |
| {1,2,3-Trimethylbenzene,Benzene,Ethylbenzene,Xylene} | {1,2,4-Trimethylbenzene} | 100.0% | 5.8% | 13 |
| {1,2,3-Trimethylbenzene,1,2,4-Trimethylbenzene,Ethylbenzene,Toluene} | {Benzene} | 100.0% | 6.2% | 14 |
| {1,2,3-Trimethylbenzene,Benzene,Ethylbenzene,Toluene} | {1,2,4-Trimethylbenzene} | 100.0% | 6.2% | 14 |
| {1,2,3-Trimethylbenzene,1,2,4-Trimethylbenzene,Toluene,Xylene} | {Benzene} | 100.0% | 5.8% | 13 |
| {1,2,3-Trimethylbenzene,1,2,4-Trimethylbenzene,Benzene,Xylene} | {Toluene} | 100.0% | 5.8% | 13 |
| {1,2,3-Trimethylbenzene,Ethylbenzene,Heptane,N-hexane} | {Toluene} | 100.0% | 5.4% | 12 |
| {1,2,3-Trimethylbenzene,Heptane,N-hexane,Toluene} | {Ethylbenzene} | 100.0% | 5.4% | 12 |
| {1,2,3-Trimethylbenzene,Ethylbenzene,N-hexane,Toluene} | {Heptane} | 100.0% | 5.4% | 12 |
| {1,2,3-Trimethylbenzene,Ethylbenzene,Heptane,N-hexane} | {Benzene} | 100.0% | 5.4% | 12 |
| {1,2,3-Trimethylbenzene,Benzene,Heptane,N-hexane} | {Ethylbenzene} | 100.0% | 5.4% | 12 |
| {1,2,3-Trimethylbenzene,Heptane,N-hexane,Toluene} | {Benzene} | 100.0% | 5.4% | 12 |
| {1,2,3-Trimethylbenzene,Benzene,Heptane,N-hexane} | {Toluene} | 100.0% | 5.4% | 12 |
| {1,2,3-Trimethylbenzene,Benzene,N-hexane,Toluene} | {Heptane} | 100.0% | 5.4% | 12 |
| {1,2,3-Trimethylbenzene,Heptane,Toluene,Xylene} | {Ethylbenzene} | 100.0% | 5.4% | 12 |
| {1,2,3-Trimethylbenzene,Benzene,Heptane,Xylene} | {Ethylbenzene} | 100.0% | 5.4% | 12 |
| {1,2,3-Trimethylbenzene,Ethylbenzene,Heptane,Toluene} | {Benzene} | 100.0% | 5.8% | 13 |
| {1,2,3-Trimethylbenzene,Benzene,Ethylbenzene,Heptane} | {Toluene} | 100.0% | 5.8% | 13 |
| {1,2,3-Trimethylbenzene,Benzene,Heptane,Toluene} | {Ethylbenzene} | 100.0% | 5.8% | 13 |
| {1,2,3-Trimethylbenzene,Heptane,Toluene,Xylene} | {Benzene} | 100.0% | 5.4% | 12 |
| {1,2,3-Trimethylbenzene,Benzene,Heptane,Xylene} | {Toluene} | 100.0% | 5.4% | 12 |
| {1,2,3-Trimethylbenzene,Ethylbenzene,N-hexane,Toluene} | {Benzene} | 100.0% | 5.4% | 12 |
| {1,2,3-Trimethylbenzene,Benzene,N-hexane,Toluene} | {Ethylbenzene} | 100.0% | 5.4% | 12 |
| {1,2,3-Trimethylbenzene,Ethylbenzene,Toluene,Xylene} | {Benzene} | 100.0% | 5.8% | 13 |
| {1,2,3-Trimethylbenzene,Benzene,Ethylbenzene,Xylene} | {Toluene} | 100.0% | 5.8% | 13 |
| {1,2,4-Trimethylbenzene,Cyclohexane,N-hexane,Pentane} | {Heptane} | 100.0% | 6.2% | 14 |
| {1,2,4-Trimethylbenzene,Cyclohexane,Heptane,Pentane} | {Ethylbenzene} | 100.0% | 6.7% | 15 |
| {1,2,4-Trimethylbenzene,Cyclohexane,Ethylbenzene,Pentane} | {Heptane} | 100.0% | 6.7% | 15 |
| {1,2,4-Trimethylbenzene,Cyclohexane,Heptane,Pentane} | {Xylene} | 100.0% | 6.7% | 15 |
| {1,2,4-Trimethylbenzene,Cyclohexane,Pentane,Xylene} | {Heptane} | 100.0% | 6.7% | 15 |
| {1,2,4-Trimethylbenzene,Cyclohexane,Heptane,Pentane} | {Toluene} | 100.0% | 6.7% | 15 |
| {1,2,4-Trimethylbenzene,Cyclohexane,Pentane,Toluene} | {Heptane} | 100.0% | 6.7% | 15 |
| {1,2,4-Trimethylbenzene,Cyclohexane,Heptane,Pentane} | {Benzene} | 100.0% | 6.7% | 15 |
| {1,2,4-Trimethylbenzene,Benzene,Cyclohexane,Pentane} | {Heptane} | 100.0% | 6.7% | 15 |
| {1,2,4-Trimethylbenzene,Cyclohexane,N-hexane,Pentane} | {Ethylbenzene} | 100.0% | 6.2% | 14 |
| {1,2,4-Trimethylbenzene,Cyclohexane,N-hexane,Pentane} | {Xylene} | 100.0% | 6.2% | 14 |
| {1,2,4-Trimethylbenzene,Cyclohexane,N-hexane,Pentane} | {Toluene} | 100.0% | 6.2% | 14 |
| {1,2,4-Trimethylbenzene,Cyclohexane,N-hexane,Pentane} | {Benzene} | 100.0% | 6.2% | 14 |
| {1,2,4-Trimethylbenzene,Cyclohexane,Ethylbenzene,Pentane} | {Xylene} | 100.0% | 6.7% | 15 |
| {1,2,4-Trimethylbenzene,Cyclohexane,Pentane,Xylene} | {Ethylbenzene} | 100.0% | 6.7% | 15 |
| {1,2,4-Trimethylbenzene,Cyclohexane,Ethylbenzene,Pentane} | {Toluene} | 100.0% | 6.7% | 15 |
| {1,2,4-Trimethylbenzene,Cyclohexane,Pentane,Toluene} | {Ethylbenzene} | 100.0% | 6.7% | 15 |
| {1,2,4-Trimethylbenzene,Cyclohexane,Ethylbenzene,Pentane} | {Benzene} | 100.0% | 6.7% | 15 |
| {1,2,4-Trimethylbenzene,Benzene,Cyclohexane,Pentane} | {Ethylbenzene} | 100.0% | 6.7% | 15 |
| {1,2,4-Trimethylbenzene,Cyclohexane,Pentane,Xylene} | {Toluene} | 100.0% | 6.7% | 15 |
| {1,2,4-Trimethylbenzene,Cyclohexane,Pentane,Toluene} | {Xylene} | 100.0% | 6.7% | 15 |
| {1,2,4-Trimethylbenzene,Cyclohexane,Pentane,Xylene} | {Benzene} | 100.0% | 6.7% | 15 |
| {1,2,4-Trimethylbenzene,Benzene,Cyclohexane,Pentane} | {Xylene} | 100.0% | 6.7% | 15 |
| {1,2,4-Trimethylbenzene,Cyclohexane,Pentane,Toluene} | {Benzene} | 100.0% | 6.7% | 15 |
| {1,2,4-Trimethylbenzene,Benzene,Cyclohexane,Pentane} | {Toluene} | 100.0% | 6.7% | 15 |
| {1,2,4-Trimethylbenzene,Cyclohexane,Heptane,N-hexane} | {Ethylbenzene} | 100.0% | 7.6% | 17 |
| {1,2,4-Trimethylbenzene,Cyclohexane,Ethylbenzene,N-hexane} | {Heptane} | 100.0% | 7.6% | 17 |
| {1,2,4-Trimethylbenzene,Cyclohexane,Heptane,N-hexane} | {Xylene} | 100.0% | 7.6% | 17 |
| {1,2,4-Trimethylbenzene,Cyclohexane,N-hexane,Xylene} | {Heptane} | 100.0% | 7.6% | 17 |
| {1,2,4-Trimethylbenzene,Cyclohexane,Heptane,N-hexane} | {Toluene} | 100.0% | 7.6% | 17 |
| {1,2,4-Trimethylbenzene,Cyclohexane,N-hexane,Toluene} | {Heptane} | 100.0% | 7.6% | 17 |
| {1,2,4-Trimethylbenzene,Cyclohexane,Heptane,N-hexane} | {Benzene} | 100.0% | 7.6% | 17 |
| {1,2,4-Trimethylbenzene,Benzene,Cyclohexane,N-hexane} | {Heptane} | 100.0% | 7.6% | 17 |
| {1,2,4-Trimethylbenzene,Cyclohexane,Ethylbenzene,Heptane} | {Xylene} | 100.0% | 8.0% | 18 |
| {1,2,4-Trimethylbenzene,Cyclohexane,Heptane,Xylene} | {Ethylbenzene} | 100.0% | 8.0% | 18 |
| {1,2,4-Trimethylbenzene,Cyclohexane,Ethylbenzene,Xylene} | {Heptane} | 100.0% | 8.0% | 18 |
| {1,2,4-Trimethylbenzene,Cyclohexane,Ethylbenzene,Heptane} | {Toluene} | 100.0% | 8.0% | 18 |
| {1,2,4-Trimethylbenzene,Cyclohexane,Heptane,Toluene} | {Ethylbenzene} | 100.0% | 8.0% | 18 |
| {1,2,4-Trimethylbenzene,Cyclohexane,Ethylbenzene,Toluene} | {Heptane} | 100.0% | 8.0% | 18 |
| {1,2,4-Trimethylbenzene,Cyclohexane,Ethylbenzene,Heptane} | {Benzene} | 100.0% | 8.0% | 18 |
| {1,2,4-Trimethylbenzene,Benzene,Cyclohexane,Heptane} | {Ethylbenzene} | 100.0% | 8.0% | 18 |
| {1,2,4-Trimethylbenzene,Benzene,Cyclohexane,Ethylbenzene} | {Heptane} | 100.0% | 8.0% | 18 |
| {1,2,4-Trimethylbenzene,Cyclohexane,Heptane,Xylene} | {Toluene} | 100.0% | 8.0% | 18 |
| {1,2,4-Trimethylbenzene,Cyclohexane,Heptane,Toluene} | {Xylene} | 100.0% | 8.0% | 18 |
| {1,2,4-Trimethylbenzene,Cyclohexane,Toluene,Xylene} | {Heptane} | 100.0% | 8.0% | 18 |
| {1,2,4-Trimethylbenzene,Cyclohexane,Heptane,Xylene} | {Benzene} | 100.0% | 8.0% | 18 |
| {1,2,4-Trimethylbenzene,Benzene,Cyclohexane,Heptane} | {Xylene} | 100.0% | 8.0% | 18 |
| {1,2,4-Trimethylbenzene,Benzene,Cyclohexane,Xylene} | {Heptane} | 100.0% | 8.0% | 18 |
| {1,2,4-Trimethylbenzene,Cyclohexane,Heptane,Toluene} | {Benzene} | 100.0% | 8.0% | 18 |
| {1,2,4-Trimethylbenzene,Benzene,Cyclohexane,Heptane} | {Toluene} | 100.0% | 8.0% | 18 |
| {1,2,4-Trimethylbenzene,Benzene,Cyclohexane,Toluene} | {Heptane} | 100.0% | 8.0% | 18 |
| {1,2,4-Trimethylbenzene,Cyclohexane,Ethylbenzene,N-hexane} | {Xylene} | 100.0% | 7.6% | 17 |
| {1,2,4-Trimethylbenzene,Cyclohexane,N-hexane,Xylene} | {Ethylbenzene} | 100.0% | 7.6% | 17 |
| {1,2,4-Trimethylbenzene,Cyclohexane,Ethylbenzene,N-hexane} | {Toluene} | 100.0% | 7.6% | 17 |
| {1,2,4-Trimethylbenzene,Cyclohexane,N-hexane,Toluene} | {Ethylbenzene} | 100.0% | 7.6% | 17 |
| {1,2,4-Trimethylbenzene,Cyclohexane,Ethylbenzene,N-hexane} | {Benzene} | 100.0% | 7.6% | 17 |
| {1,2,4-Trimethylbenzene,Benzene,Cyclohexane,N-hexane} | {Ethylbenzene} | 100.0% | 7.6% | 17 |
| {1,2,4-Trimethylbenzene,Cyclohexane,N-hexane,Xylene} | {Toluene} | 100.0% | 7.6% | 17 |
| {1,2,4-Trimethylbenzene,Cyclohexane,N-hexane,Toluene} | {Xylene} | 100.0% | 7.6% | 17 |
| {1,2,4-Trimethylbenzene,Cyclohexane,N-hexane,Xylene} | {Benzene} | 100.0% | 7.6% | 17 |
| {1,2,4-Trimethylbenzene,Benzene,Cyclohexane,N-hexane} | {Xylene} | 100.0% | 7.6% | 17 |
| {1,2,4-Trimethylbenzene,Cyclohexane,N-hexane,Toluene} | {Benzene} | 100.0% | 7.6% | 17 |
| {1,2,4-Trimethylbenzene,Benzene,Cyclohexane,N-hexane} | {Toluene} | 100.0% | 7.6% | 17 |
| {1,2,4-Trimethylbenzene,Cyclohexane,Ethylbenzene,Xylene} | {Toluene} | 100.0% | 8.0% | 18 |
| {1,2,4-Trimethylbenzene,Cyclohexane,Ethylbenzene,Toluene} | {Xylene} | 100.0% | 8.0% | 18 |
| {1,2,4-Trimethylbenzene,Cyclohexane,Toluene,Xylene} | {Ethylbenzene} | 100.0% | 8.0% | 18 |
| {1,2,4-Trimethylbenzene,Cyclohexane,Ethylbenzene,Xylene} | {Benzene} | 100.0% | 8.0% | 18 |
| {1,2,4-Trimethylbenzene,Benzene,Cyclohexane,Ethylbenzene} | {Xylene} | 100.0% | 8.0% | 18 |
| {1,2,4-Trimethylbenzene,Benzene,Cyclohexane,Xylene} | {Ethylbenzene} | 100.0% | 8.0% | 18 |
| {1,2,4-Trimethylbenzene,Cyclohexane,Ethylbenzene,Toluene} | {Benzene} | 100.0% | 8.0% | 18 |
| {1,2,4-Trimethylbenzene,Benzene,Cyclohexane,Ethylbenzene} | {Toluene} | 100.0% | 8.0% | 18 |
| {1,2,4-Trimethylbenzene,Benzene,Cyclohexane,Toluene} | {Ethylbenzene} | 100.0% | 8.0% | 18 |
| {1,2,4-Trimethylbenzene,Cyclohexane,Toluene,Xylene} | {Benzene} | 100.0% | 8.0% | 18 |
| {1,2,4-Trimethylbenzene,Benzene,Cyclohexane,Xylene} | {Toluene} | 100.0% | 8.0% | 18 |
| {1,2,4-Trimethylbenzene,Benzene,Cyclohexane,Toluene} | {Xylene} | 100.0% | 8.0% | 18 |
| {1,2,4-Trimethylbenzene,Heptane,N-hexane,Pentane} | {Ethylbenzene} | 100.0% | 7.6% | 17 |
| {1,2,4-Trimethylbenzene,Heptane,N-hexane,Pentane} | {Xylene} | 100.0% | 7.6% | 17 |
| {1,2,4-Trimethylbenzene,Heptane,N-hexane,Pentane} | {Toluene} | 100.0% | 7.6% | 17 |
| {1,2,4-Trimethylbenzene,Heptane,N-hexane,Pentane} | {Benzene} | 100.0% | 7.6% | 17 |
| {1,2,4-Trimethylbenzene,Ethylbenzene,Heptane,Pentane} | {Xylene} | 100.0% | 8.0% | 18 |
| {1,2,4-Trimethylbenzene,Heptane,Pentane,Xylene} | {Ethylbenzene} | 100.0% | 8.0% | 18 |
| {1,2,4-Trimethylbenzene,Ethylbenzene,Heptane,Pentane} | {Toluene} | 100.0% | 8.0% | 18 |
| {1,2,4-Trimethylbenzene,Heptane,Pentane,Toluene} | {Ethylbenzene} | 100.0% | 8.0% | 18 |
| {1,2,4-Trimethylbenzene,Ethylbenzene,Heptane,Pentane} | {Benzene} | 100.0% | 8.0% | 18 |
| {1,2,4-Trimethylbenzene,Benzene,Heptane,Pentane} | {Ethylbenzene} | 100.0% | 8.0% | 18 |
| {1,2,4-Trimethylbenzene,Heptane,Pentane,Xylene} | {Toluene} | 100.0% | 8.0% | 18 |
| {1,2,4-Trimethylbenzene,Heptane,Pentane,Toluene} | {Xylene} | 100.0% | 8.0% | 18 |
| {1,2,4-Trimethylbenzene,Heptane,Pentane,Xylene} | {Benzene} | 100.0% | 8.0% | 18 |
| {1,2,4-Trimethylbenzene,Benzene,Heptane,Pentane} | {Xylene} | 100.0% | 8.0% | 18 |
| {1,2,4-Trimethylbenzene,Heptane,Pentane,Toluene} | {Benzene} | 100.0% | 8.0% | 18 |
| {1,2,4-Trimethylbenzene,Benzene,Heptane,Pentane} | {Toluene} | 100.0% | 8.0% | 18 |
| {1,2,4-Trimethylbenzene,N-hexane,Pentane,Xylene} | {Ethylbenzene} | 100.0% | 8.0% | 18 |
| {1,2,4-Trimethylbenzene,N-hexane,Pentane,Toluene} | {Ethylbenzene} | 100.0% | 8.0% | 18 |
| {1,2,4-Trimethylbenzene,Ethylbenzene,N-hexane,Pentane} | {Benzene} | 100.0% | 8.5% | 19 |
| {1,2,4-Trimethylbenzene,Benzene,N-hexane,Pentane} | {Ethylbenzene} | 100.0% | 8.5% | 19 |
| {1,2,4-Trimethylbenzene,N-hexane,Pentane,Xylene} | {Toluene} | 100.0% | 8.0% | 18 |
| {1,2,4-Trimethylbenzene,N-hexane,Pentane,Toluene} | {Xylene} | 100.0% | 8.0% | 18 |
| {1,2,4-Trimethylbenzene,N-hexane,Pentane,Xylene} | {Benzene} | 100.0% | 8.0% | 18 |
| {1,2,4-Trimethylbenzene,N-hexane,Pentane,Toluene} | {Benzene} | 100.0% | 8.0% | 18 |
| {1,2,4-Trimethylbenzene,Ethylbenzene,Pentane,Xylene} | {Toluene} | 100.0% | 8.5% | 19 |
| {1,2,4-Trimethylbenzene,Ethylbenzene,Pentane,Toluene} | {Xylene} | 100.0% | 8.5% | 19 |
| {1,2,4-Trimethylbenzene,Pentane,Toluene,Xylene} | {Ethylbenzene} | 100.0% | 8.5% | 19 |
| {1,2,4-Trimethylbenzene,Ethylbenzene,Pentane,Xylene} | {Benzene} | 100.0% | 8.5% | 19 |
| {1,2,4-Trimethylbenzene,Benzene,Pentane,Xylene} | {Ethylbenzene} | 100.0% | 8.5% | 19 |
| {1,2,4-Trimethylbenzene,Ethylbenzene,Pentane,Toluene} | {Benzene} | 100.0% | 8.5% | 19 |
| {1,2,4-Trimethylbenzene,Pentane,Toluene,Xylene} | {Benzene} | 100.0% | 8.5% | 19 |
| {1,2,4-Trimethylbenzene,Benzene,Pentane,Xylene} | {Toluene} | 100.0% | 8.5% | 19 |
| {1,2,4-Trimethylbenzene,Heptane,N-hexane,Xylene} | {Ethylbenzene} | 100.0% | 9.4% | 21 |
| {1,2,4-Trimethylbenzene,Ethylbenzene,Heptane,N-hexane} | {Toluene} | 100.0% | 9.8% | 22 |
| {1,2,4-Trimethylbenzene,Heptane,N-hexane,Toluene} | {Ethylbenzene} | 100.0% | 9.8% | 22 |
| {1,2,4-Trimethylbenzene,Ethylbenzene,Heptane,N-hexane} | {Benzene} | 100.0% | 9.8% | 22 |
| {1,2,4-Trimethylbenzene,Benzene,Heptane,N-hexane} | {Ethylbenzene} | 100.0% | 9.8% | 22 |
| {1,2,4-Trimethylbenzene,Heptane,N-hexane,Xylene} | {Toluene} | 100.0% | 9.4% | 21 |
| {1,2,4-Trimethylbenzene,Heptane,N-hexane,Xylene} | {Benzene} | 100.0% | 9.4% | 21 |
| {1,2,4-Trimethylbenzene,Heptane,N-hexane,Toluene} | {Benzene} | 100.0% | 9.8% | 22 |
| {1,2,4-Trimethylbenzene,Benzene,Heptane,N-hexane} | {Toluene} | 100.0% | 9.8% | 22 |
| {1,2,4-Trimethylbenzene,Heptane,Toluene,Xylene} | {Ethylbenzene} | 100.0% | 10.7% | 24 |
| {1,2,4-Trimethylbenzene,Benzene,Heptane,Xylene} | {Ethylbenzene} | 100.0% | 10.7% | 24 |
| {1,2,4-Trimethylbenzene,Ethylbenzene,Heptane,Toluene} | {Benzene} | 100.0% | 11.2% | 25 |
| {1,2,4-Trimethylbenzene,Benzene,Ethylbenzene,Heptane} | {Toluene} | 100.0% | 11.2% | 25 |
| {1,2,4-Trimethylbenzene,Benzene,Heptane,Toluene} | {Ethylbenzene} | 100.0% | 11.2% | 25 |
| {1,2,4-Trimethylbenzene,Heptane,Toluene,Xylene} | {Benzene} | 100.0% | 10.7% | 24 |
| {1,2,4-Trimethylbenzene,Benzene,Heptane,Xylene} | {Toluene} | 100.0% | 10.7% | 24 |
| {1,2,4-Trimethylbenzene,Ethylbenzene,N-hexane,Xylene} | {Toluene} | 100.0% | 10.3% | 23 |
| {1,2,4-Trimethylbenzene,Ethylbenzene,N-hexane,Xylene} | {Benzene} | 100.0% | 10.3% | 23 |
| {1,2,4-Trimethylbenzene,Ethylbenzene,N-hexane,Toluene} | {Benzene} | 100.0% | 10.7% | 24 |
| {1,2,4-Trimethylbenzene,N-hexane,Toluene,Xylene} | {Benzene} | 100.0% | 10.7% | 24 |
| {1,2,4-Trimethylbenzene,Benzene,N-hexane,Xylene} | {Toluene} | 100.0% | 10.7% | 24 |
| {1,2,4-Trimethylbenzene,Benzene,Ethylbenzene,Xylene} | {Toluene} | 100.0% | 12.5% | 28 |
| {Cyclohexane,Heptane,N-hexane,Pentane} | {Toluene} | 100.0% | 8.9% | 20 |
| {Cyclohexane,Heptane,N-hexane,Pentane} | {Benzene} | 100.0% | 8.9% | 20 |
| {Cyclohexane,Ethylbenzene,Heptane,Pentane} | {Xylene} | 100.0% | 8.5% | 19 |
| {Cyclohexane,Ethylbenzene,Heptane,Pentane} | {Toluene} | 100.0% | 8.5% | 19 |
| {Cyclohexane,Ethylbenzene,Heptane,Pentane} | {Benzene} | 100.0% | 8.5% | 19 |
| {Cyclohexane,Heptane,Pentane,Xylene} | {Toluene} | 100.0% | 8.9% | 20 |
| {Cyclohexane,Heptane,Pentane,Xylene} | {Benzene} | 100.0% | 8.9% | 20 |
| {Cyclohexane,Heptane,Pentane,Toluene} | {Benzene} | 100.0% | 9.4% | 21 |
| {Benzene,Cyclohexane,Heptane,Pentane} | {Toluene} | 100.0% | 9.4% | 21 |
| {Cyclohexane,Ethylbenzene,N-hexane,Pentane} | {Xylene} | 100.0% | 8.5% | 19 |
| {Cyclohexane,Ethylbenzene,N-hexane,Pentane} | {Toluene} | 100.0% | 8.5% | 19 |
| {Cyclohexane,Ethylbenzene,N-hexane,Pentane} | {Benzene} | 100.0% | 8.5% | 19 |
| {Cyclohexane,N-hexane,Pentane,Xylene} | {Toluene} | 100.0% | 8.9% | 20 |
| {Cyclohexane,N-hexane,Pentane,Xylene} | {Benzene} | 100.0% | 8.9% | 20 |
| {Cyclohexane,N-hexane,Pentane,Toluene} | {Benzene} | 100.0% | 9.4% | 21 |
| {Benzene,Cyclohexane,N-hexane,Pentane} | {Toluene} | 100.0% | 9.4% | 21 |
| {Cyclohexane,Ethylbenzene,Pentane,Xylene} | {Toluene} | 100.0% | 9.4% | 21 |
| {Cyclohexane,Ethylbenzene,Pentane,Toluene} | {Xylene} | 100.0% | 9.4% | 21 |
| {Benzene,Cyclohexane,Ethylbenzene,Pentane} | {Xylene} | 100.0% | 8.9% | 20 |
| {Benzene,Cyclohexane,Ethylbenzene,Pentane} | {Toluene} | 100.0% | 8.9% | 20 |
| {Benzene,Cyclohexane,Pentane,Xylene} | {Toluene} | 100.0% | 9.4% | 21 |
| {Cyclohexane,Ethylbenzene,Heptane,N-hexane} | {Xylene} | 100.0% | 9.8% | 22 |
| {Cyclohexane,Ethylbenzene,Heptane,N-hexane} | {Toluene} | 100.0% | 9.8% | 22 |
| {Cyclohexane,Ethylbenzene,Heptane,N-hexane} | {Benzene} | 100.0% | 9.8% | 22 |
| {Cyclohexane,Heptane,N-hexane,Xylene} | {Toluene} | 100.0% | 10.7% | 24 |
| {Cyclohexane,Heptane,N-hexane,Xylene} | {Benzene} | 100.0% | 10.7% | 24 |
| {Cyclohexane,Heptane,N-hexane,Toluene} | {Benzene} | 100.0% | 11.6% | 26 |
| {Benzene,Cyclohexane,Heptane,N-hexane} | {Toluene} | 100.0% | 11.6% | 26 |
| {Cyclohexane,Ethylbenzene,Heptane,Xylene} | {Toluene} | 100.0% | 10.3% | 23 |
| {Cyclohexane,Ethylbenzene,Heptane,Toluene} | {Xylene} | 100.0% | 10.3% | 23 |
| {Cyclohexane,Ethylbenzene,Heptane,Xylene} | {Benzene} | 100.0% | 10.3% | 23 |
| {Benzene,Cyclohexane,Ethylbenzene,Heptane} | {Xylene} | 100.0% | 10.3% | 23 |
| {Cyclohexane,Ethylbenzene,Heptane,Toluene} | {Benzene} | 100.0% | 10.3% | 23 |
| {Benzene,Cyclohexane,Ethylbenzene,Heptane} | {Toluene} | 100.0% | 10.3% | 23 |
| {Cyclohexane,Heptane,Toluene,Xylene} | {Benzene} | 100.0% | 11.2% | 25 |
| {Benzene,Cyclohexane,Heptane,Xylene} | {Toluene} | 100.0% | 11.2% | 25 |
| {Cyclohexane,Ethylbenzene,N-hexane,Toluene} | {Xylene} | 100.0% | 11.6% | 26 |
| {Benzene,Cyclohexane,Ethylbenzene,N-hexane} | {Xylene} | 100.0% | 11.6% | 26 |
| {Cyclohexane,Ethylbenzene,N-hexane,Toluene} | {Benzene} | 100.0% | 11.6% | 26 |
| {Benzene,Cyclohexane,Ethylbenzene,N-hexane} | {Toluene} | 100.0% | 11.6% | 26 |
| {Cyclohexane,N-hexane,Toluene,Xylene} | {Benzene} | 100.0% | 13.8% | 31 |
| {Benzene,Cyclohexane,Ethylbenzene,Xylene} | {Toluene} | 100.0% | 12.1% | 27 |
| {Benzene,Cyclohexane,Ethylbenzene,Toluene} | {Xylene} | 100.0% | 12.1% | 27 |
| {Ethylbenzene,Heptane,N-hexane,Pentane} | {Xylene} | 100.0% | 9.4% | 21 |
| {Ethylbenzene,Heptane,N-hexane,Pentane} | {Toluene} | 100.0% | 9.4% | 21 |
| {Ethylbenzene,Heptane,N-hexane,Pentane} | {Benzene} | 100.0% | 9.4% | 21 |
| {Heptane,N-hexane,Pentane,Toluene} | {Benzene} | 100.0% | 12.1% | 27 |
| {Benzene,Heptane,N-hexane,Pentane} | {Toluene} | 100.0% | 12.1% | 27 |
| {Ethylbenzene,Heptane,Pentane,Xylene} | {Toluene} | 100.0% | 9.8% | 22 |
| {Ethylbenzene,Heptane,Pentane,Toluene} | {Xylene} | 100.0% | 9.8% | 22 |
| {Ethylbenzene,Heptane,Pentane,Xylene} | {Benzene} | 100.0% | 9.8% | 22 |
| {Benzene,Ethylbenzene,Heptane,Pentane} | {Xylene} | 100.0% | 9.8% | 22 |
| {Ethylbenzene,Heptane,Pentane,Toluene} | {Benzene} | 100.0% | 9.8% | 22 |
| {Benzene,Ethylbenzene,Heptane,Pentane} | {Toluene} | 100.0% | 9.8% | 22 |
| {Heptane,Pentane,Toluene,Xylene} | {Benzene} | 100.0% | 11.6% | 26 |
| {Benzene,Heptane,Pentane,Xylene} | {Toluene} | 100.0% | 11.6% | 26 |
| {Ethylbenzene,N-hexane,Pentane,Xylene} | {Toluene} | 100.0% | 10.3% | 23 |
| {Ethylbenzene,N-hexane,Pentane,Toluene} | {Xylene} | 100.0% | 10.3% | 23 |
| {Ethylbenzene,N-hexane,Pentane,Xylene} | {Benzene} | 100.0% | 10.3% | 23 |
| {Ethylbenzene,N-hexane,Pentane,Toluene} | {Benzene} | 100.0% | 10.3% | 23 |
| {N-hexane,Pentane,Toluene,Xylene} | {Benzene} | 100.0% | 12.5% | 28 |
| {Benzene,N-hexane,Pentane,Xylene} | {Toluene} | 100.0% | 12.5% | 28 |
| {Benzene,Ethylbenzene,Pentane,Xylene} | {Toluene} | 100.0% | 11.2% | 25 |
| {Benzene,Ethylbenzene,Pentane,Toluene} | {Xylene} | 100.0% | 11.2% | 25 |
| {Ethylbenzene,Heptane,N-hexane,Xylene} | {Toluene} | 100.0% | 11.6% | 26 |
| {Ethylbenzene,Heptane,N-hexane,Xylene} | {Benzene} | 100.0% | 11.6% | 26 |
| {Ethylbenzene,Heptane,N-hexane,Toluene} | {Benzene} | 100.0% | 12.1% | 27 |
| {Benzene,Ethylbenzene,Heptane,N-hexane} | {Toluene} | 100.0% | 12.1% | 27 |
| {Heptane,N-hexane,Toluene,Xylene} | {Benzene} | 100.0% | 13.8% | 31 |
| {Benzene,Heptane,N-hexane,Xylene} | {Toluene} | 100.0% | 13.8% | 31 |
| {Ethylbenzene,Heptane,Toluene,Xylene} | {Benzene} | 100.0% | 13.4% | 30 |
| {Benzene,Ethylbenzene,Heptane,Xylene} | {Toluene} | 100.0% | 13.4% | 30 |
| {Ethylbenzene,N-hexane,Toluene,Xylene} | {Benzene} | 100.0% | 14.3% | 32 |
| {Benzene,Ethylbenzene,N-hexane,Xylene} | {Toluene} | 100.0% | 14.3% | 32 |
| {1,2,3-Trimethylbenzene,1,2,4-Trimethylbenzene,Ethylbenzene,Heptane,N-hexane} | {Toluene} | 100.0% | 5.4% | 12 |
| {1,2,3-Trimethylbenzene,1,2,4-Trimethylbenzene,Heptane,N-hexane,Toluene} | {Ethylbenzene} | 100.0% | 5.4% | 12 |
| {1,2,3-Trimethylbenzene,1,2,4-Trimethylbenzene,Ethylbenzene,N-hexane,Toluene} | {Heptane} | 100.0% | 5.4% | 12 |
| {1,2,3-Trimethylbenzene,Ethylbenzene,Heptane,N-hexane,Toluene} | {1,2,4-Trimethylbenzene} | 100.0% | 5.4% | 12 |
| {1,2,3-Trimethylbenzene,1,2,4-Trimethylbenzene,Ethylbenzene,Heptane,N-hexane} | {Benzene} | 100.0% | 5.4% | 12 |
| {1,2,3-Trimethylbenzene,1,2,4-Trimethylbenzene,Benzene,Heptane,N-hexane} | {Ethylbenzene} | 100.0% | 5.4% | 12 |
| {1,2,3-Trimethylbenzene,Benzene,Ethylbenzene,Heptane,N-hexane} | {1,2,4-Trimethylbenzene} | 100.0% | 5.4% | 12 |
| {1,2,3-Trimethylbenzene,1,2,4-Trimethylbenzene,Heptane,N-hexane,Toluene} | {Benzene} | 100.0% | 5.4% | 12 |
| {1,2,3-Trimethylbenzene,1,2,4-Trimethylbenzene,Benzene,Heptane,N-hexane} | {Toluene} | 100.0% | 5.4% | 12 |
| {1,2,3-Trimethylbenzene,1,2,4-Trimethylbenzene,Benzene,N-hexane,Toluene} | {Heptane} | 100.0% | 5.4% | 12 |
| {1,2,3-Trimethylbenzene,Benzene,Heptane,N-hexane,Toluene} | {1,2,4-Trimethylbenzene} | 100.0% | 5.4% | 12 |
| {1,2,3-Trimethylbenzene,1,2,4-Trimethylbenzene,Heptane,Toluene,Xylene} | {Ethylbenzene} | 100.0% | 5.4% | 12 |
| {1,2,3-Trimethylbenzene,Ethylbenzene,Heptane,Toluene,Xylene} | {1,2,4-Trimethylbenzene} | 100.0% | 5.4% | 12 |
| {1,2,3-Trimethylbenzene,1,2,4-Trimethylbenzene,Benzene,Heptane,Xylene} | {Ethylbenzene} | 100.0% | 5.4% | 12 |
| {1,2,3-Trimethylbenzene,Benzene,Ethylbenzene,Heptane,Xylene} | {1,2,4-Trimethylbenzene} | 100.0% | 5.4% | 12 |
| {1,2,3-Trimethylbenzene,1,2,4-Trimethylbenzene,Ethylbenzene,Heptane,Toluene} | {Benzene} | 100.0% | 5.8% | 13 |
| {1,2,3-Trimethylbenzene,1,2,4-Trimethylbenzene,Benzene,Ethylbenzene,Heptane} | {Toluene} | 100.0% | 5.8% | 13 |
| {1,2,3-Trimethylbenzene,1,2,4-Trimethylbenzene,Benzene,Heptane,Toluene} | {Ethylbenzene} | 100.0% | 5.8% | 13 |
| {1,2,3-Trimethylbenzene,Benzene,Ethylbenzene,Heptane,Toluene} | {1,2,4-Trimethylbenzene} | 100.0% | 5.8% | 13 |
| {1,2,3-Trimethylbenzene,1,2,4-Trimethylbenzene,Heptane,Toluene,Xylene} | {Benzene} | 100.0% | 5.4% | 12 |
| {1,2,3-Trimethylbenzene,1,2,4-Trimethylbenzene,Benzene,Heptane,Xylene} | {Toluene} | 100.0% | 5.4% | 12 |
| {1,2,3-Trimethylbenzene,Benzene,Heptane,Toluene,Xylene} | {1,2,4-Trimethylbenzene} | 100.0% | 5.4% | 12 |
| {1,2,3-Trimethylbenzene,1,2,4-Trimethylbenzene,Ethylbenzene,N-hexane,Toluene} | {Benzene} | 100.0% | 5.4% | 12 |
| {1,2,3-Trimethylbenzene,1,2,4-Trimethylbenzene,Benzene,N-hexane,Toluene} | {Ethylbenzene} | 100.0% | 5.4% | 12 |
| {1,2,3-Trimethylbenzene,Benzene,Ethylbenzene,N-hexane,Toluene} | {1,2,4-Trimethylbenzene} | 100.0% | 5.4% | 12 |
| {1,2,3-Trimethylbenzene,1,2,4-Trimethylbenzene,Ethylbenzene,Toluene,Xylene} | {Benzene} | 100.0% | 5.8% | 13 |
| {1,2,3-Trimethylbenzene,1,2,4-Trimethylbenzene,Benzene,Ethylbenzene,Xylene} | {Toluene} | 100.0% | 5.8% | 13 |
| {1,2,3-Trimethylbenzene,1,2,4-Trimethylbenzene,Benzene,Toluene,Xylene} | {Ethylbenzene} | 100.0% | 5.8% | 13 |
| {1,2,3-Trimethylbenzene,Benzene,Ethylbenzene,Toluene,Xylene} | {1,2,4-Trimethylbenzene} | 100.0% | 5.8% | 13 |
| {1,2,3-Trimethylbenzene,Ethylbenzene,Heptane,N-hexane,Toluene} | {Benzene} | 100.0% | 5.4% | 12 |
| {1,2,3-Trimethylbenzene,Benzene,Ethylbenzene,Heptane,N-hexane} | {Toluene} | 100.0% | 5.4% | 12 |
| {1,2,3-Trimethylbenzene,Benzene,Heptane,N-hexane,Toluene} | {Ethylbenzene} | 100.0% | 5.4% | 12 |
| {1,2,3-Trimethylbenzene,Benzene,Ethylbenzene,N-hexane,Toluene} | {Heptane} | 100.0% | 5.4% | 12 |
| {1,2,3-Trimethylbenzene,Ethylbenzene,Heptane,Toluene,Xylene} | {Benzene} | 100.0% | 5.4% | 12 |
| {1,2,3-Trimethylbenzene,Benzene,Ethylbenzene,Heptane,Xylene} | {Toluene} | 100.0% | 5.4% | 12 |
| {1,2,3-Trimethylbenzene,Benzene,Heptane,Toluene,Xylene} | {Ethylbenzene} | 100.0% | 5.4% | 12 |
| {1,2,4-Trimethylbenzene,Cyclohexane,Heptane,N-hexane,Pentane} | {Ethylbenzene} | 100.0% | 6.2% | 14 |
| {1,2,4-Trimethylbenzene,Cyclohexane,Ethylbenzene,N-hexane,Pentane} | {Heptane} | 100.0% | 6.2% | 14 |
| {1,2,4-Trimethylbenzene,Cyclohexane,Heptane,N-hexane,Pentane} | {Xylene} | 100.0% | 6.2% | 14 |

##### Association rules with the largest lift among WS exposed to a minimum of 2 agents. Minimum level of support = 0.1 %, minimum confidence 0.1.

| Antecedent | Consequent | Lift | % Conf (A\(\rightarrow\)C) | % Conf (C\(\rightarrow\)A) | % Support | N WS |
| --- | --- | --- | --- | --- | --- | --- |
| {Phenol} | {Methanol} | 1244.4 | 83.3% | 66.7% | 4.5% | 10 |
| {Cumene} | {1,2,3-Trimethylbenzene} | 1071.8 | 90.9% | 52.6% | 4.5% | 10 |
| {Acetonitrile} | {Methanol} | 995.6 | 66.7% | 66.7% | 4.5% | 10 |
| {Mesitylene} | {1,2,3-Trimethylbenzene} | 970.9 | 82.4% | 73.7% | 6.2% | 14 |
| {Styrene} | {Chloroethylene} | 600.0 | 75.0% | 53.6% | 6.7% | 15 |
| {Mesitylene} | {1,2,4-Trimethylbenzene} | 589.5 | 100.0% | 44.7% | 7.6% | 17 |
| {1,2,3-Trimethylbenzene,Cumene} | {1,2,4-Trimethylbenzene} | 589.5 | 100.0% | 26.3% | 4.5% | 10 |
| {1,2,3-Trimethylbenzene,Mesitylene} | {1,2,4-Trimethylbenzene} | 589.5 | 100.0% | 36.8% | 6.2% | 14 |
| {1,2,3-Trimethylbenzene} | {1,2,4-Trimethylbenzene} | 558.4 | 94.7% | 47.4% | 8.0% | 18 |
| {Cumene} | {1,2,4-Trimethylbenzene} | 535.9 | 90.9% | 26.3% | 4.5% | 10 |
| {2-Methylbutane} | {1,2,4-Trimethylbenzene} | 471.6 | 80.0% | 31.6% | 5.4% | 12 |
| {1,2,4-Trimethylbenzene,Cyclohexane} | {Heptane} | 393.0 | 100.0% | 31.6% | 8.0% | 18 |
| {1,2,4-Trimethylbenzene,Cyclohexane,Pentane} | {Heptane} | 393.0 | 100.0% | 26.3% | 6.7% | 15 |
| {Acetone,Cyclohexane} | {Heptane} | 357.3 | 90.9% | 17.5% | 4.5% | 10 |
| {1,2,4-Trimethylbenzene,Cyclohexane} | {Pentane} | 345.7 | 83.3% | 27.8% | 6.7% | 15 |
| {Cyclohexane,Pentane} | {Heptane} | 343.9 | 87.5% | 36.8% | 9.4% | 21 |
| {Acetone} | {Cyclohexane} | 327.7 | 68.8% | 23.4% | 4.9% | 11 |
| {1,2,4-Trimethylbenzene,Pentane} | {Heptane} | 307.6 | 78.3% | 31.6% | 8.0% | 18 |
| {1,2,3-Trimethylbenzene,1,2,4-Trimethylbenzene} | {Heptane} | 305.7 | 77.8% | 24.6% | 6.2% | 14 |
| {Acetone} | {Heptane} | 294.7 | 75.0% | 21.1% | 5.4% | 12 |
| {1,2,3-Trimethylbenzene} | {Heptane} | 289.6 | 73.7% | 24.6% | 6.2% | 14 |
| {1,2,3-Trimethylbenzene,Mesitylene} | {Heptane} | 280.7 | 71.4% | 17.5% | 4.5% | 10 |
| {1,2,3-Trimethylbenzene,1,2,4-Trimethylbenzene,Mesitylene} | {Heptane} | 280.7 | 71.4% | 17.5% | 4.5% | 10 |
| {2-Methylbutane} | {Pentane} | 276.5 | 66.7% | 18.5% | 4.5% | 10 |
| {1,2,4-Trimethylbenzene} | {Heptane} | 268.9 | 68.4% | 45.6% | 11.6% | 26 |
| {Cyclohexane,Heptane} | {N-hexane} | 267.0 | 96.6% | 34.6% | 12.5% | 28 |
| {Cyclohexane,Heptane,Pentane} | {N-hexane} | 263.4 | 95.2% | 24.7% | 8.9% | 20 |
| {1,2,4-Trimethylbenzene,Cyclohexane} | {N-hexane} | 261.2 | 94.4% | 21.0% | 7.6% | 17 |
| {1,2,4-Trimethylbenzene,Cyclohexane,Heptane} | {N-hexane} | 261.2 | 94.4% | 21.0% | 7.6% | 17 |
| {1,2,4-Trimethylbenzene,Heptane,Pentane} | {N-hexane} | 261.2 | 94.4% | 21.0% | 7.6% | 17 |
| {Heptane,Pentane} | {N-hexane} | 258.1 | 93.3% | 34.6% | 12.5% | 28 |
| {1,2,4-Trimethylbenzene,Cyclohexane,Pentane} | {N-hexane} | 258.1 | 93.3% | 17.3% | 6.2% | 14 |
| {1,2,4-Trimethylbenzene,Cyclohexane,Heptane,Pentane} | {N-hexane} | 258.1 | 93.3% | 17.3% | 6.2% | 14 |
| {Mesitylene} | {Heptane} | 254.3 | 64.7% | 19.3% | 4.9% | 11 |
| {1,2,4-Trimethylbenzene,Mesitylene} | {Heptane} | 254.3 | 64.7% | 19.3% | 4.9% | 11 |
| {1,2,3-Trimethylbenzene,1,2,4-Trimethylbenzene} | {Pentane} | 253.5 | 61.1% | 20.4% | 4.9% | 11 |
| {Cyclohexane} | {N-hexane} | 253.0 | 91.5% | 53.1% | 19.2% | 43 |
| {Acetone,Cyclohexane} | {N-hexane} | 251.4 | 90.9% | 12.3% | 4.5% | 10 |
| {Heptane,Mesitylene} | {N-hexane} | 251.4 | 90.9% | 12.3% | 4.5% | 10 |
| {1,2,3-Trimethylbenzene,Pentane} | {N-hexane} | 251.4 | 90.9% | 12.3% | 4.5% | 10 |
| {1,2,4-Trimethylbenzene,Heptane,Mesitylene} | {N-hexane} | 251.4 | 90.9% | 12.3% | 4.5% | 10 |
| {1,2,3-Trimethylbenzene,1,2,4-Trimethylbenzene,Pentane} | {N-hexane} | 251.4 | 90.9% | 12.3% | 4.5% | 10 |
| {1,2,4-Trimethylbenzene} | {Pentane} | 251.1 | 60.5% | 42.6% | 10.3% | 23 |
| {Cyclohexane} | {Heptane} | 242.5 | 61.7% | 50.9% | 12.9% | 29 |
| {Cyclohexane,Pentane} | {N-hexane} | 242.0 | 87.5% | 25.9% | 9.4% | 21 |
| {1,2,3-Trimethylbenzene,Cumene} | {Ethylbenzene} | 240.9 | 100.0% | 10.8% | 4.5% | 10 |
| {1,2,4-Trimethylbenzene,Cumene} | {Ethylbenzene} | 240.9 | 100.0% | 10.8% | 4.5% | 10 |
| {1,2,4-Trimethylbenzene,2-Methylbutane} | {Ethylbenzene} | 240.9 | 100.0% | 12.9% | 5.4% | 12 |
| {Heptane,Mesitylene} | {Ethylbenzene} | 240.9 | 100.0% | 11.8% | 4.9% | 11 |
| {Mesitylene,N-hexane} | {Ethylbenzene} | 240.9 | 100.0% | 11.8% | 4.9% | 11 |
| {1,2,3-Trimethylbenzene,Pentane} | {Ethylbenzene} | 240.9 | 100.0% | 11.8% | 4.9% | 11 |
| {1,2,3-Trimethylbenzene,Heptane} | {Ethylbenzene} | 240.9 | 100.0% | 15.1% | 6.2% | 14 |
| {1,2,3-Trimethylbenzene,N-hexane} | {Ethylbenzene} | 240.9 | 100.0% | 14.0% | 5.8% | 13 |
| {1,2,4-Trimethylbenzene,Cyclohexane} | {Ethylbenzene} | 240.9 | 100.0% | 19.4% | 8.0% | 18 |
| {1,2,4-Trimethylbenzene,Heptane} | {Ethylbenzene} | 240.9 | 100.0% | 28.0% | 11.6% | 26 |
| {1,2,3-Trimethylbenzene,1,2,4-Trimethylbenzene,Cumene} | {Ethylbenzene} | 240.9 | 100.0% | 10.8% | 4.5% | 10 |
| {1,2,4-Trimethylbenzene,2-Methylbutane,N-hexane} | {Ethylbenzene} | 240.9 | 100.0% | 10.8% | 4.5% | 10 |
| {1,2,3-Trimethylbenzene,Heptane,Mesitylene} | {Ethylbenzene} | 240.9 | 100.0% | 10.8% | 4.5% | 10 |
| {1,2,3-Trimethylbenzene,Mesitylene,N-hexane} | {Ethylbenzene} | 240.9 | 100.0% | 10.8% | 4.5% | 10 |
| {1,2,4-Trimethylbenzene,Heptane,Mesitylene} | {Ethylbenzene} | 240.9 | 100.0% | 11.8% | 4.9% | 11 |
| {1,2,4-Trimethylbenzene,Mesitylene,N-hexane} | {Ethylbenzene} | 240.9 | 100.0% | 11.8% | 4.9% | 11 |
| {Heptane,Mesitylene,N-hexane} | {Ethylbenzene} | 240.9 | 100.0% | 10.8% | 4.5% | 10 |
| {1,2,3-Trimethylbenzene,1,2,4-Trimethylbenzene,Pentane} | {Ethylbenzene} | 240.9 | 100.0% | 11.8% | 4.9% | 11 |
| {1,2,3-Trimethylbenzene,1,2,4-Trimethylbenzene,Heptane} | {Ethylbenzene} | 240.9 | 100.0% | 15.1% | 6.2% | 14 |
| {1,2,3-Trimethylbenzene,1,2,4-Trimethylbenzene,N-hexane} | {Ethylbenzene} | 240.9 | 100.0% | 14.0% | 5.8% | 13 |
| {1,2,3-Trimethylbenzene,N-hexane,Pentane} | {Ethylbenzene} | 240.9 | 100.0% | 10.8% | 4.5% | 10 |
| {1,2,3-Trimethylbenzene,Heptane,N-hexane} | {Ethylbenzene} | 240.9 | 100.0% | 12.9% | 5.4% | 12 |
| {1,2,4-Trimethylbenzene,Cyclohexane,Pentane} | {Ethylbenzene} | 240.9 | 100.0% | 16.1% | 6.7% | 15 |
| {1,2,4-Trimethylbenzene,Cyclohexane,Heptane} | {Ethylbenzene} | 240.9 | 100.0% | 19.4% | 8.0% | 18 |
| {1,2,4-Trimethylbenzene,Cyclohexane,N-hexane} | {Ethylbenzene} | 240.9 | 100.0% | 18.3% | 7.6% | 17 |
| {1,2,4-Trimethylbenzene,Heptane,Pentane} | {Ethylbenzene} | 240.9 | 100.0% | 19.4% | 8.0% | 18 |
| {1,2,4-Trimethylbenzene,N-hexane,Pentane} | {Ethylbenzene} | 240.9 | 100.0% | 20.4% | 8.5% | 19 |
| {1,2,4-Trimethylbenzene,Heptane,N-hexane} | {Ethylbenzene} | 240.9 | 100.0% | 23.7% | 9.8% | 22 |
| {1,2,3-Trimethylbenzene,1,2,4-Trimethylbenzene,Heptane,Mesitylene} | {Ethylbenzene} | 240.9 | 100.0% | 10.8% | 4.5% | 10 |
| {1,2,3-Trimethylbenzene,1,2,4-Trimethylbenzene,Mesitylene,N-hexane} | {Ethylbenzene} | 240.9 | 100.0% | 10.8% | 4.5% | 10 |
| {1,2,4-Trimethylbenzene,Heptane,Mesitylene,N-hexane} | {Ethylbenzene} | 240.9 | 100.0% | 10.8% | 4.5% | 10 |
| {1,2,3-Trimethylbenzene,1,2,4-Trimethylbenzene,N-hexane,Pentane} | {Ethylbenzene} | 240.9 | 100.0% | 10.8% | 4.5% | 10 |
| {1,2,3-Trimethylbenzene,1,2,4-Trimethylbenzene,Heptane,N-hexane} | {Ethylbenzene} | 240.9 | 100.0% | 12.9% | 5.4% | 12 |
| {1,2,4-Trimethylbenzene,Cyclohexane,Heptane,Pentane} | {Ethylbenzene} | 240.9 | 100.0% | 16.1% | 6.7% | 15 |
| {1,2,4-Trimethylbenzene,Cyclohexane,N-hexane,Pentane} | {Ethylbenzene} | 240.9 | 100.0% | 15.1% | 6.2% | 14 |
| {1,2,4-Trimethylbenzene,Cyclohexane,Heptane,N-hexane} | {Ethylbenzene} | 240.9 | 100.0% | 18.3% | 7.6% | 17 |
| {1,2,4-Trimethylbenzene,Heptane,N-hexane,Pentane} | {Ethylbenzene} | 240.9 | 100.0% | 18.3% | 7.6% | 17 |
| {1,2,4-Trimethylbenzene,Cyclohexane,Heptane,N-hexane,Pentane} | {Ethylbenzene} | 240.9 | 100.0% | 15.1% | 6.2% | 14 |
| {1,2,3-Trimethylbenzene} | {Pentane} | 240.2 | 57.9% | 20.4% | 4.9% | 11 |
| {1,2,3-Trimethylbenzene,Heptane} | {N-hexane} | 237.0 | 85.7% | 14.8% | 5.4% | 12 |
| {1,2,3-Trimethylbenzene,1,2,4-Trimethylbenzene,Heptane} | {N-hexane} | 237.0 | 85.7% | 14.8% | 5.4% | 12 |
| {1,2,4-Trimethylbenzene,Heptane} | {N-hexane} | 234.0 | 84.6% | 27.2% | 9.8% | 22 |
| {1,2,4-Trimethylbenzene,N-hexane} | {Ethylbenzene} | 231.6 | 96.2% | 26.9% | 11.2% | 25 |
| {1,2,4-Trimethylbenzene,2-Methylbutane} | {N-hexane} | 230.5 | 83.3% | 12.3% | 4.5% | 10 |
| {Acetone,Heptane} | {N-hexane} | 230.5 | 83.3% | 12.3% | 4.5% | 10 |
| {1,2,4-Trimethylbenzene,Pentane} | {Ethylbenzene} | 230.4 | 95.7% | 23.7% | 9.8% | 22 |
| {1,2,4-Trimethylbenzene,Pentane} | {N-hexane} | 228.4 | 82.6% | 23.5% | 8.5% | 19 |
| {1,2,3-Trimethylbenzene,1,2,4-Trimethylbenzene} | {Ethylbenzene} | 227.5 | 94.4% | 18.3% | 7.6% | 17 |
| {Mesitylene} | {Ethylbenzene} | 226.7 | 94.1% | 17.2% | 7.1% | 16 |
| {1,2,4-Trimethylbenzene,Mesitylene} | {Ethylbenzene} | 226.7 | 94.1% | 17.2% | 7.1% | 16 |
| {1,2,4-Trimethylbenzene} | {Cyclohexane} | 225.8 | 47.4% | 38.3% | 8.0% | 18 |
| {1,2,3-Trimethylbenzene,Mesitylene} | {Ethylbenzene} | 223.7 | 92.9% | 14.0% | 5.8% | 13 |
| {1,2,3-Trimethylbenzene,1,2,4-Trimethylbenzene,Mesitylene} | {Ethylbenzene} | 223.7 | 92.9% | 14.0% | 5.8% | 13 |
| {Cumene} | {Ethylbenzene} | 219.0 | 90.9% | 10.8% | 4.5% | 10 |
| {2-Methylbutane,N-hexane} | {Ethylbenzene} | 219.0 | 90.9% | 10.8% | 4.5% | 10 |
| {Pentane} | {Heptane} | 218.3 | 55.6% | 52.6% | 13.4% | 30 |
| {Cyclohexane,Heptane,Pentane} | {Ethylbenzene} | 217.9 | 90.5% | 20.4% | 8.5% | 19 |
| {Cyclohexane,N-hexane,Pentane} | {Ethylbenzene} | 217.9 | 90.5% | 20.4% | 8.5% | 19 |
| {Cyclohexane,Heptane,N-hexane,Pentane} | {Ethylbenzene} | 216.8 | 90.0% | 19.4% | 8.0% | 18 |
| {1,2,3-Trimethylbenzene} | {Ethylbenzene} | 215.5 | 89.5% | 18.3% | 7.6% | 17 |
| {1,2,4-Trimethylbenzene} | {Ethylbenzene} | 215.5 | 89.5% | 36.6% | 15.2% | 34 |
| {Cyclohexane} | {Pentane} | 211.8 | 51.1% | 44.4% | 10.7% | 24 |
| {Cyclohexane,Pentane} | {Ethylbenzene} | 210.8 | 87.5% | 22.6% | 9.4% | 21 |
| {Pentane} | {N-hexane} | 210.0 | 75.9% | 50.6% | 18.3% | 41 |
| {Acetone} | {N-hexane} | 207.4 | 75.0% | 14.8% | 5.4% | 12 |
| {2-Methylbutane} | {N-hexane} | 202.8 | 73.3% | 13.6% | 4.9% | 11 |
| {1,2,3-Trimethylbenzene,1,2,4-Trimethylbenzene} | {N-hexane} | 199.7 | 72.2% | 16.0% | 5.8% | 13 |
| {1,2,3-Trimethylbenzene,Mesitylene} | {N-hexane} | 197.5 | 71.4% | 12.3% | 4.5% | 10 |
| {1,2,3-Trimethylbenzene,1,2,4-Trimethylbenzene,Mesitylene} | {N-hexane} | 197.5 | 71.4% | 12.3% | 4.5% | 10 |
| {2-Methylbutane} | {Ethylbenzene} | 192.7 | 80.0% | 12.9% | 5.4% | 12 |
| {Cyclohexane,Heptane} | {Ethylbenzene} | 191.0 | 79.3% | 24.7% | 10.3% | 23 |
| {Cyclohexane,Heptane,N-hexane} | {Ethylbenzene} | 189.2 | 78.6% | 23.7% | 9.8% | 22 |
| {1,2,3-Trimethylbenzene} | {N-hexane} | 189.2 | 68.4% | 16.0% | 5.8% | 13 |
| {1,2,4-Trimethylbenzene} | {N-hexane} | 189.2 | 68.4% | 32.1% | 11.6% | 26 |
| {Heptane} | {N-hexane} | 189.2 | 68.4% | 48.1% | 17.4% | 39 |
| {Ammonia, anhydrous} | {N-hexane} | 184.4 | 66.7% | 12.3% | 4.5% | 10 |
| {Heptane,N-hexane,Pentane} | {Ethylbenzene} | 180.6 | 75.0% | 22.6% | 9.4% | 21 |
| {Mesitylene} | {N-hexane} | 178.9 | 64.7% | 13.6% | 4.9% | 11 |
| {1,2,4-Trimethylbenzene,Mesitylene} | {N-hexane} | 178.9 | 64.7% | 13.6% | 4.9% | 11 |
| {Heptane,Pentane} | {Ethylbenzene} | 176.6 | 73.3% | 23.7% | 9.8% | 22 |
| {Phenol} | {Xylene} | 171.0 | 100.0% | 9.2% | 5.4% | 12 |
| {Methanol,Phenol} | {Xylene} | 171.0 | 100.0% | 7.6% | 4.5% | 10 |
| {Acetonitrile,Methanol} | {Xylene} | 171.0 | 100.0% | 7.6% | 4.5% | 10 |
| {Ethylbenzene,Styrene} | {Xylene} | 171.0 | 100.0% | 7.6% | 4.5% | 10 |
| {Chloroethylene,Ethylbenzene} | {Xylene} | 171.0 | 100.0% | 11.5% | 6.7% | 15 |
| {1,2,4-Trimethylbenzene,Cyclohexane} | {Xylene} | 171.0 | 100.0% | 13.7% | 8.0% | 18 |
| {Cyclohexane,Ethylbenzene} | {Xylene} | 171.0 | 100.0% | 22.1% | 12.9% | 29 |
| {1,2,4-Trimethylbenzene,Cyclohexane,Pentane} | {Xylene} | 171.0 | 100.0% | 11.5% | 6.7% | 15 |
| {1,2,4-Trimethylbenzene,Cyclohexane,Heptane} | {Xylene} | 171.0 | 100.0% | 13.7% | 8.0% | 18 |
| {1,2,4-Trimethylbenzene,Cyclohexane,N-hexane} | {Xylene} | 171.0 | 100.0% | 13.0% | 7.6% | 17 |
| {1,2,4-Trimethylbenzene,Cyclohexane,Ethylbenzene} | {Xylene} | 171.0 | 100.0% | 13.7% | 8.0% | 18 |
| {1,2,4-Trimethylbenzene,Heptane,Pentane} | {Xylene} | 171.0 | 100.0% | 13.7% | 8.0% | 18 |
| {Cyclohexane,Ethylbenzene,Pentane} | {Xylene} | 171.0 | 100.0% | 16.0% | 9.4% | 21 |
| {Cyclohexane,Ethylbenzene,Heptane} | {Xylene} | 171.0 | 100.0% | 17.6% | 10.3% | 23 |
| {Cyclohexane,Ethylbenzene,N-hexane} | {Xylene} | 171.0 | 100.0% | 20.6% | 12.1% | 27 |
| {Ethylbenzene,Heptane,Pentane} | {Xylene} | 171.0 | 100.0% | 16.8% | 9.8% | 22 |
| {1,2,4-Trimethylbenzene,Cyclohexane,Heptane,Pentane} | {Xylene} | 171.0 | 100.0% | 11.5% | 6.7% | 15 |
| {1,2,4-Trimethylbenzene,Cyclohexane,N-hexane,Pentane} | {Xylene} | 171.0 | 100.0% | 10.7% | 6.2% | 14 |
| {1,2,4-Trimethylbenzene,Cyclohexane,Ethylbenzene,Pentane} | {Xylene} | 171.0 | 100.0% | 11.5% | 6.7% | 15 |
| {1,2,4-Trimethylbenzene,Cyclohexane,Heptane,N-hexane} | {Xylene} | 171.0 | 100.0% | 13.0% | 7.6% | 17 |
| {1,2,4-Trimethylbenzene,Cyclohexane,Ethylbenzene,Heptane} | {Xylene} | 171.0 | 100.0% | 13.7% | 8.0% | 18 |
| {1,2,4-Trimethylbenzene,Cyclohexane,Ethylbenzene,N-hexane} | {Xylene} | 171.0 | 100.0% | 13.0% | 7.6% | 17 |
| {1,2,4-Trimethylbenzene,Heptane,N-hexane,Pentane} | {Xylene} | 171.0 | 100.0% | 13.0% | 7.6% | 17 |
| {1,2,4-Trimethylbenzene,Ethylbenzene,Heptane,Pentane} | {Xylene} | 171.0 | 100.0% | 13.7% | 8.0% | 18 |
| {Cyclohexane,Ethylbenzene,Heptane,Pentane} | {Xylene} | 171.0 | 100.0% | 14.5% | 8.5% | 19 |
| {Cyclohexane,Ethylbenzene,N-hexane,Pentane} | {Xylene} | 171.0 | 100.0% | 14.5% | 8.5% | 19 |
| {Cyclohexane,Ethylbenzene,Heptane,N-hexane} | {Xylene} | 171.0 | 100.0% | 16.8% | 9.8% | 22 |
| {Ethylbenzene,Heptane,N-hexane,Pentane} | {Xylene} | 171.0 | 100.0% | 16.0% | 9.4% | 21 |
| {1,2,4-Trimethylbenzene,Cyclohexane,Heptane,N-hexane,Pentane} | {Xylene} | 171.0 | 100.0% | 10.7% | 6.2% | 14 |
| {1,2,4-Trimethylbenzene,Cyclohexane,Ethylbenzene,Heptane,Pentane} | {Xylene} | 171.0 | 100.0% | 11.5% | 6.7% | 15 |
| {1,2,4-Trimethylbenzene,Cyclohexane,Ethylbenzene,N-hexane,Pentane} | {Xylene} | 171.0 | 100.0% | 10.7% | 6.2% | 14 |
| {1,2,4-Trimethylbenzene,Cyclohexane,Ethylbenzene,Heptane,N-hexane} | {Xylene} | 171.0 | 100.0% | 13.0% | 7.6% | 17 |
| {1,2,4-Trimethylbenzene,Ethylbenzene,Heptane,N-hexane,Pentane} | {Xylene} | 171.0 | 100.0% | 13.0% | 7.6% | 17 |
| {Cyclohexane,Ethylbenzene,Heptane,N-hexane,Pentane} | {Xylene} | 171.0 | 100.0% | 13.7% | 8.0% | 18 |
| {1,2,4-Trimethylbenzene,Cyclohexane,Ethylbenzene,Heptane,N-hexane,Pentane} | {Xylene} | 171.0 | 100.0% | 10.7% | 6.2% | 14 |
| {Heptane,N-hexane} | {Ethylbenzene} | 166.7 | 69.2% | 29.0% | 12.1% | 27 |
| {Ethylbenzene,Heptane} | {Xylene} | 165.6 | 96.9% | 23.7% | 13.8% | 31 |
| {Ethylbenzene,Heptane,N-hexane} | {Xylene} | 164.7 | 96.3% | 19.8% | 11.6% | 26 |
| {1,2,4-Trimethylbenzene,Heptane} | {Xylene} | 164.4 | 96.2% | 19.1% | 11.2% | 25 |
| {1,2,4-Trimethylbenzene,Ethylbenzene,Heptane} | {Xylene} | 164.4 | 96.2% | 19.1% | 11.2% | 25 |
| {Ethylbenzene,N-hexane,Pentane} | {Xylene} | 163.9 | 95.8% | 17.6% | 10.3% | 23 |
| {Ethylbenzene} | {Xylene} | 163.6 | 95.7% | 67.9% | 39.7% | 89 |
| {1,2,4-Trimethylbenzene,Heptane,N-hexane} | {Xylene} | 163.2 | 95.5% | 16.0% | 9.4% | 21 |
| {1,2,4-Trimethylbenzene,Ethylbenzene,Heptane,N-hexane} | {Xylene} | 163.2 | 95.5% | 16.0% | 9.4% | 21 |
| {Cyclohexane,Heptane,Pentane} | {Xylene} | 162.8 | 95.2% | 15.3% | 8.9% | 20 |
| {Cyclohexane,N-hexane,Pentane} | {Xylene} | 162.8 | 95.2% | 15.3% | 8.9% | 20 |
| {Cyclohexane,Heptane,N-hexane,Pentane} | {Xylene} | 162.4 | 95.0% | 14.5% | 8.5% | 19 |
| {1,2,4-Trimethylbenzene,N-hexane,Pentane} | {Xylene} | 162.0 | 94.7% | 13.7% | 8.0% | 18 |
| {1,2,4-Trimethylbenzene,Ethylbenzene,N-hexane,Pentane} | {Xylene} | 162.0 | 94.7% | 13.7% | 8.0% | 18 |
| {Ethylbenzene,N-hexane} | {Xylene} | 161.5 | 94.4% | 26.0% | 15.2% | 34 |
| {Methanol} | {Xylene} | 159.6 | 93.3% | 10.7% | 6.2% | 14 |
| {Chloroethylene,Styrene} | {Xylene} | 159.6 | 93.3% | 10.7% | 6.2% | 14 |
| {1,2,3-Trimethylbenzene,Heptane} | {Xylene} | 158.8 | 92.9% | 9.9% | 5.8% | 13 |
| {1,2,3-Trimethylbenzene,1,2,4-Trimethylbenzene,Heptane} | {Xylene} | 158.8 | 92.9% | 9.9% | 5.8% | 13 |
| {1,2,3-Trimethylbenzene,Ethylbenzene,Heptane} | {Xylene} | 158.8 | 92.9% | 9.9% | 5.8% | 13 |
| {Heptane,N-hexane,Pentane} | {Xylene} | 158.8 | 92.9% | 19.8% | 11.6% | 26 |
| {1,2,3-Trimethylbenzene,1,2,4-Trimethylbenzene,Ethylbenzene,Heptane} | {Xylene} | 158.8 | 92.9% | 9.9% | 5.8% | 13 |
| {1,2,4-Trimethylbenzene,N-hexane} | {Xylene} | 157.8 | 92.3% | 18.3% | 10.7% | 24 |
| {1,2,4-Trimethylbenzene,Ethylbenzene,N-hexane} | {Xylene} | 157.3 | 92.0% | 17.6% | 10.3% | 23 |
| {1,2,4-Trimethylbenzene,2-Methylbutane} | {Xylene} | 156.7 | 91.7% | 8.4% | 4.9% | 11 |
| {2-Methylbutane,Ethylbenzene} | {Xylene} | 156.7 | 91.7% | 8.4% | 4.9% | 11 |
| {Acetone,N-hexane} | {Xylene} | 156.7 | 91.7% | 8.4% | 4.9% | 11 |
| {Cyclohexane,Pentane} | {Xylene} | 156.7 | 91.7% | 16.8% | 9.8% | 22 |
| {1,2,4-Trimethylbenzene,2-Methylbutane,Ethylbenzene} | {Xylene} | 156.7 | 91.7% | 8.4% | 4.9% | 11 |
| {1,2,3-Trimethylbenzene,Heptane,N-hexane} | {Xylene} | 156.7 | 91.7% | 8.4% | 4.9% | 11 |
| {1,2,3-Trimethylbenzene,1,2,4-Trimethylbenzene,Heptane,N-hexane} | {Xylene} | 156.7 | 91.7% | 8.4% | 4.9% | 11 |
| {1,2,3-Trimethylbenzene,Ethylbenzene,Heptane,N-hexane} | {Xylene} | 156.7 | 91.7% | 8.4% | 4.9% | 11 |
| {1,2,3-Trimethylbenzene,1,2,4-Trimethylbenzene,Ethylbenzene,Heptane,N-hexane} | {Xylene} | 156.7 | 91.7% | 8.4% | 4.9% | 11 |
| {Acetone,Cyclohexane} | {Xylene} | 155.4 | 90.9% | 7.6% | 4.5% | 10 |
| {Heptane,Mesitylene} | {Xylene} | 155.4 | 90.9% | 7.6% | 4.5% | 10 |
| {1,2,4-Trimethylbenzene,Heptane,Mesitylene} | {Xylene} | 155.4 | 90.9% | 7.6% | 4.5% | 10 |
| {Ethylbenzene,Heptane,Mesitylene} | {Xylene} | 155.4 | 90.9% | 7.6% | 4.5% | 10 |
| {1,2,4-Trimethylbenzene,Ethylbenzene,Heptane,Mesitylene} | {Xylene} | 155.4 | 90.9% | 7.6% | 4.5% | 10 |
| {Heptane,Pentane} | {Xylene} | 153.9 | 90.0% | 20.6% | 12.1% | 27 |
| {Ethylbenzene,Pentane} | {Xylene} | 153.3 | 89.7% | 19.8% | 11.6% | 26 |
| {Cyclohexane,N-hexane} | {Ethylbenzene} | 151.2 | 62.8% | 29.0% | 12.1% | 27 |
| {1,2,4-Trimethylbenzene,Ethylbenzene} | {Xylene} | 150.9 | 88.2% | 22.9% | 13.4% | 30 |
| {Cyclohexane} | {Ethylbenzene} | 148.6 | 61.7% | 31.2% | 12.9% | 29 |
| {1,2,4-Trimethylbenzene,Ethylbenzene,Pentane} | {Xylene} | 147.7 | 86.4% | 14.5% | 8.5% | 19 |
| {Cyclohexane,Heptane} | {Xylene} | 147.4 | 86.2% | 19.1% | 11.2% | 25 |
| {Cyclohexane,Heptane,N-hexane} | {Xylene} | 146.6 | 85.7% | 18.3% | 10.7% | 24 |
| {1,2,3-Trimethylbenzene,N-hexane} | {Xylene} | 144.7 | 84.6% | 8.4% | 4.9% | 11 |
| {1,2,3-Trimethylbenzene,1,2,4-Trimethylbenzene,N-hexane} | {Xylene} | 144.7 | 84.6% | 8.4% | 4.9% | 11 |
| {1,2,3-Trimethylbenzene,Ethylbenzene,N-hexane} | {Xylene} | 144.7 | 84.6% | 8.4% | 4.9% | 11 |
| {1,2,3-Trimethylbenzene,1,2,4-Trimethylbenzene,Ethylbenzene,N-hexane} | {Xylene} | 144.7 | 84.6% | 8.4% | 4.9% | 11 |
| {Acetone,Heptane} | {Xylene} | 142.5 | 83.3% | 7.6% | 4.5% | 10 |
| {1,2,4-Trimethylbenzene,Pentane} | {Xylene} | 141.3 | 82.6% | 14.5% | 8.5% | 19 |
| {N-hexane,Pentane} | {Ethylbenzene} | 141.0 | 58.5% | 25.8% | 10.7% | 24 |
| {1,2,3-Trimethylbenzene,Ethylbenzene} | {Xylene} | 140.8 | 82.4% | 10.7% | 6.2% | 14 |
| {1,2,3-Trimethylbenzene,1,2,4-Trimethylbenzene,Ethylbenzene} | {Xylene} | 140.8 | 82.4% | 10.7% | 6.2% | 14 |
| {Heptane,N-hexane} | {Xylene} | 140.3 | 82.1% | 24.4% | 14.3% | 32 |
| {1,2,4-Trimethylbenzene} | {Xylene} | 139.5 | 81.6% | 23.7% | 13.8% | 31 |
| {Styrene} | {Xylene} | 136.8 | 80.0% | 12.2% | 7.1% | 16 |
| {Heptane} | {Ethylbenzene} | 135.2 | 56.1% | 34.4% | 14.3% | 32 |
| {Cyclohexane,N-hexane} | {Xylene} | 135.2 | 79.1% | 26.0% | 15.2% | 34 |
| {1,2,3-Trimethylbenzene} | {Xylene} | 135.0 | 78.9% | 11.5% | 6.7% | 15 |
| {1,2,3-Trimethylbenzene,1,2,4-Trimethylbenzene} | {Xylene} | 133.0 | 77.8% | 10.7% | 6.2% | 14 |
| {Acetonitrile} | {Benzene} | 131.8 | 100.0% | 8.8% | 6.7% | 15 |
| {2-Methylbutane} | {Toluene} | 131.8 | 100.0% | 8.8% | 6.7% | 15 |
| {Acetonitrile,Methanol} | {Benzene} | 131.8 | 100.0% | 5.9% | 4.5% | 10 |
| {Acetonitrile,Xylene} | {Benzene} | 131.8 | 100.0% | 5.9% | 4.5% | 10 |
| {1,2,3-Trimethylbenzene,Cumene} | {Benzene} | 131.8 | 100.0% | 5.9% | 4.5% | 10 |
| {1,2,4-Trimethylbenzene,Cumene} | {Benzene} | 131.8 | 100.0% | 5.9% | 4.5% | 10 |
| {Cumene,Ethylbenzene} | {Benzene} | 131.8 | 100.0% | 5.9% | 4.5% | 10 |
| {Styrene,Toluene} | {Benzene} | 131.8 | 100.0% | 7.1% | 5.4% | 12 |
| {Ammonia, anhydrous,N-hexane} | {Toluene} | 131.8 | 100.0% | 5.9% | 4.5% | 10 |
| {1,2,4-Trimethylbenzene,2-Methylbutane} | {Toluene} | 131.8 | 100.0% | 7.1% | 5.4% | 12 |
| {2-Methylbutane,Pentane} | {Toluene} | 131.8 | 100.0% | 5.9% | 4.5% | 10 |
| {2-Methylbutane,N-hexane} | {Toluene} | 131.8 | 100.0% | 6.5% | 4.9% | 11 |
| {2-Methylbutane,N-hexane} | {Benzene} | 131.8 | 100.0% | 6.5% | 4.9% | 11 |
| {2-Methylbutane,Ethylbenzene} | {Toluene} | 131.8 | 100.0% | 7.1% | 5.4% | 12 |
| {2-Methylbutane,Xylene} | {Toluene} | 131.8 | 100.0% | 6.5% | 4.9% | 11 |
| {Acetone,Cyclohexane} | {Toluene} | 131.8 | 100.0% | 6.5% | 4.9% | 11 |
| {Acetone,Cyclohexane} | {Benzene} | 131.8 | 100.0% | 6.5% | 4.9% | 11 |
| {Acetone,N-hexane} | {Toluene} | 131.8 | 100.0% | 7.1% | 5.4% | 12 |
| {Acetone,N-hexane} | {Benzene} | 131.8 | 100.0% | 7.1% | 5.4% | 12 |
| {Acetone,Xylene} | {Toluene} | 131.8 | 100.0% | 7.1% | 5.4% | 12 |
| {Acetone,Xylene} | {Benzene} | 131.8 | 100.0% | 7.1% | 5.4% | 12 |
| {Acetone,Toluene} | {Benzene} | 131.8 | 100.0% | 7.6% | 5.8% | 13 |
| {Chloroethylene,Toluene} | {Benzene} | 131.8 | 100.0% | 10.0% | 7.6% | 17 |
| {1,2,3-Trimethylbenzene,Mesitylene} | {Benzene} | 131.8 | 100.0% | 8.2% | 6.2% | 14 |
| {Heptane,Mesitylene} | {Toluene} | 131.8 | 100.0% | 6.5% | 4.9% | 11 |
| {Heptane,Mesitylene} | {Benzene} | 131.8 | 100.0% | 6.5% | 4.9% | 11 |
| {Mesitylene,N-hexane} | {Benzene} | 131.8 | 100.0% | 6.5% | 4.9% | 11 |
| {Mesitylene,Xylene} | {Toluene} | 131.8 | 100.0% | 7.1% | 5.4% | 12 |
| {1,2,3-Trimethylbenzene,Pentane} | {Benzene} | 131.8 | 100.0% | 6.5% | 4.9% | 11 |
| {1,2,3-Trimethylbenzene,N-hexane} | {Benzene} | 131.8 | 100.0% | 7.6% | 5.8% | 13 |
| {1,2,3-Trimethylbenzene,Toluene} | {Benzene} | 131.8 | 100.0% | 9.4% | 7.1% | 16 |
| {1,2,4-Trimethylbenzene,Cyclohexane} | {Toluene} | 131.8 | 100.0% | 10.6% | 8.0% | 18 |
| {1,2,4-Trimethylbenzene,Cyclohexane} | {Benzene} | 131.8 | 100.0% | 10.6% | 8.0% | 18 |
| {1,2,4-Trimethylbenzene,Pentane} | {Benzene} | 131.8 | 100.0% | 13.5% | 10.3% | 23 |
| {1,2,4-Trimethylbenzene,N-hexane} | {Benzene} | 131.8 | 100.0% | 15.3% | 11.6% | 26 |
| {Cyclohexane,Pentane} | {Toluene} | 131.8 | 100.0% | 14.1% | 10.7% | 24 |
| {Acetonitrile,Methanol,Xylene} | {Benzene} | 131.8 | 100.0% | 5.9% | 4.5% | 10 |
| {1,2,3-Trimethylbenzene,1,2,4-Trimethylbenzene,Cumene} | {Benzene} | 131.8 | 100.0% | 5.9% | 4.5% | 10 |
| {1,2,3-Trimethylbenzene,Cumene,Ethylbenzene} | {Benzene} | 131.8 | 100.0% | 5.9% | 4.5% | 10 |
| {1,2,4-Trimethylbenzene,Cumene,Ethylbenzene} | {Benzene} | 131.8 | 100.0% | 5.9% | 4.5% | 10 |
| {Chloroethylene,Styrene,Toluene} | {Benzene} | 131.8 | 100.0% | 5.9% | 4.5% | 10 |
| {Styrene,Toluene,Xylene} | {Benzene} | 131.8 | 100.0% | 6.5% | 4.9% | 11 |
| {1,2,4-Trimethylbenzene,2-Methylbutane,N-hexane} | {Toluene} | 131.8 | 100.0% | 5.9% | 4.5% | 10 |
| {1,2,4-Trimethylbenzene,2-Methylbutane,N-hexane} | {Benzene} | 131.8 | 100.0% | 5.9% | 4.5% | 10 |
| {1,2,4-Trimethylbenzene,2-Methylbutane,Ethylbenzene} | {Toluene} | 131.8 | 100.0% | 7.1% | 5.4% | 12 |
| {1,2,4-Trimethylbenzene,2-Methylbutane,Xylene} | {Toluene} | 131.8 | 100.0% | 6.5% | 4.9% | 11 |
| {2-Methylbutane,Ethylbenzene,N-hexane} | {Toluene} | 131.8 | 100.0% | 5.9% | 4.5% | 10 |
| {2-Methylbutane,Ethylbenzene,N-hexane} | {Benzene} | 131.8 | 100.0% | 5.9% | 4.5% | 10 |
| {2-Methylbutane,N-hexane,Toluene} | {Benzene} | 131.8 | 100.0% | 6.5% | 4.9% | 11 |
| {2-Methylbutane,Ethylbenzene,Xylene} | {Toluene} | 131.8 | 100.0% | 6.5% | 4.9% | 11 |
| {Acetone,Cyclohexane,Heptane} | {Toluene} | 131.8 | 100.0% | 5.9% | 4.5% | 10 |
| {Acetone,Cyclohexane,Heptane} | {Benzene} | 131.8 | 100.0% | 5.9% | 4.5% | 10 |
| {Acetone,Cyclohexane,N-hexane} | {Toluene} | 131.8 | 100.0% | 5.9% | 4.5% | 10 |
| {Acetone,Cyclohexane,N-hexane} | {Benzene} | 131.8 | 100.0% | 5.9% | 4.5% | 10 |
| {Acetone,Cyclohexane,Xylene} | {Toluene} | 131.8 | 100.0% | 5.9% | 4.5% | 10 |
| {Acetone,Cyclohexane,Xylene} | {Benzene} | 131.8 | 100.0% | 5.9% | 4.5% | 10 |
| {Acetone,Cyclohexane,Toluene} | {Benzene} | 131.8 | 100.0% | 6.5% | 4.9% | 11 |
| {Acetone,Heptane,N-hexane} | {Toluene} | 131.8 | 100.0% | 5.9% | 4.5% | 10 |
| {Acetone,Heptane,N-hexane} | {Benzene} | 131.8 | 100.0% | 5.9% | 4.5% | 10 |
| {Acetone,Heptane,Xylene} | {Toluene} | 131.8 | 100.0% | 5.9% | 4.5% | 10 |
| {Acetone,Heptane,Xylene} | {Benzene} | 131.8 | 100.0% | 5.9% | 4.5% | 10 |
| {Acetone,Heptane,Toluene} | {Benzene} | 131.8 | 100.0% | 6.5% | 4.9% | 11 |
| {Acetone,N-hexane,Xylene} | {Toluene} | 131.8 | 100.0% | 6.5% | 4.9% | 11 |
| {Acetone,N-hexane,Xylene} | {Benzene} | 131.8 | 100.0% | 6.5% | 4.9% | 11 |
| {Acetone,N-hexane,Toluene} | {Benzene} | 131.8 | 100.0% | 7.1% | 5.4% | 12 |
| {Acetone,Toluene,Xylene} | {Benzene} | 131.8 | 100.0% | 7.1% | 5.4% | 12 |
| {Chloroethylene,Ethylbenzene,Toluene} | {Benzene} | 131.8 | 100.0% | 8.2% | 6.2% | 14 |
| {Chloroethylene,Toluene,Xylene} | {Benzene} | 131.8 | 100.0% | 8.8% | 6.7% | 15 |
| {1,2,3-Trimethylbenzene,1,2,4-Trimethylbenzene,Mesitylene} | {Benzene} | 131.8 | 100.0% | 8.2% | 6.2% | 14 |
| {1,2,3-Trimethylbenzene,Heptane,Mesitylene} | {Toluene} | 131.8 | 100.0% | 5.9% | 4.5% | 10 |
| {1,2,3-Trimethylbenzene,Heptane,Mesitylene} | {Benzene} | 131.8 | 100.0% | 5.9% | 4.5% | 10 |
| {1,2,3-Trimethylbenzene,Mesitylene,N-hexane} | {Benzene} | 131.8 | 100.0% | 5.9% | 4.5% | 10 |
| {1,2,3-Trimethylbenzene,Ethylbenzene,Mesitylene} | {Benzene} | 131.8 | 100.0% | 7.6% | 5.8% | 13 |
| {1,2,3-Trimethylbenzene,Mesitylene,Xylene} | {Toluene} | 131.8 | 100.0% | 5.9% | 4.5% | 10 |
| {1,2,3-Trimethylbenzene,Mesitylene,Xylene} | {Benzene} | 131.8 | 100.0% | 5.9% | 4.5% | 10 |
| {1,2,3-Trimethylbenzene,Mesitylene,Toluene} | {Benzene} | 131.8 | 100.0% | 7.1% | 5.4% | 12 |
| {1,2,4-Trimethylbenzene,Heptane,Mesitylene} | {Toluene} | 131.8 | 100.0% | 6.5% | 4.9% | 11 |
| {1,2,4-Trimethylbenzene,Heptane,Mesitylene} | {Benzene} | 131.8 | 100.0% | 6.5% | 4.9% | 11 |
| {1,2,4-Trimethylbenzene,Mesitylene,N-hexane} | {Benzene} | 131.8 | 100.0% | 6.5% | 4.9% | 11 |
| {1,2,4-Trimethylbenzene,Mesitylene,Xylene} | {Toluene} | 131.8 | 100.0% | 7.1% | 5.4% | 12 |
| {Heptane,Mesitylene,N-hexane} | {Toluene} | 131.8 | 100.0% | 5.9% | 4.5% | 10 |
| {Heptane,Mesitylene,N-hexane} | {Benzene} | 131.8 | 100.0% | 5.9% | 4.5% | 10 |
| {Ethylbenzene,Heptane,Mesitylene} | {Toluene} | 131.8 | 100.0% | 6.5% | 4.9% | 11 |
| {Ethylbenzene,Heptane,Mesitylene} | {Benzene} | 131.8 | 100.0% | 6.5% | 4.9% | 11 |
| {Heptane,Mesitylene,Xylene} | {Toluene} | 131.8 | 100.0% | 5.9% | 4.5% | 10 |
| {Heptane,Mesitylene,Xylene} | {Benzene} | 131.8 | 100.0% | 5.9% | 4.5% | 10 |
| {Heptane,Mesitylene,Toluene} | {Benzene} | 131.8 | 100.0% | 6.5% | 4.9% | 11 |
| {Ethylbenzene,Mesitylene,N-hexane} | {Benzene} | 131.8 | 100.0% | 6.5% | 4.9% | 11 |
| {Mesitylene,N-hexane,Toluene} | {Benzene} | 131.8 | 100.0% | 5.9% | 4.5% | 10 |
| {Ethylbenzene,Mesitylene,Xylene} | {Toluene} | 131.8 | 100.0% | 7.1% | 5.4% | 12 |
| {1,2,3-Trimethylbenzene,1,2,4-Trimethylbenzene,Pentane} | {Benzene} | 131.8 | 100.0% | 6.5% | 4.9% | 11 |
| {1,2,3-Trimethylbenzene,1,2,4-Trimethylbenzene,N-hexane} | {Benzene} | 131.8 | 100.0% | 7.6% | 5.8% | 13 |
| {1,2,3-Trimethylbenzene,1,2,4-Trimethylbenzene,Toluene} | {Benzene} | 131.8 | 100.0% | 8.8% | 6.7% | 15 |
| {1,2,3-Trimethylbenzene,N-hexane,Pentane} | {Benzene} | 131.8 | 100.0% | 5.9% | 4.5% | 10 |
| {1,2,3-Trimethylbenzene,Ethylbenzene,Pentane} | {Benzene} | 131.8 | 100.0% | 6.5% | 4.9% | 11 |
| {1,2,3-Trimethylbenzene,Heptane,N-hexane} | {Toluene} | 131.8 | 100.0% | 7.1% | 5.4% | 12 |
| {1,2,3-Trimethylbenzene,Heptane,N-hexane} | {Benzene} | 131.8 | 100.0% | 7.1% | 5.4% | 12 |
| {1,2,3-Trimethylbenzene,Heptane,Toluene} | {Benzene} | 131.8 | 100.0% | 7.6% | 5.8% | 13 |
| {1,2,3-Trimethylbenzene,Ethylbenzene,N-hexane} | {Benzene} | 131.8 | 100.0% | 7.6% | 5.8% | 13 |
| {1,2,3-Trimethylbenzene,N-hexane,Xylene} | {Toluene} | 131.8 | 100.0% | 6.5% | 4.9% | 11 |
| {1,2,3-Trimethylbenzene,N-hexane,Xylene} | {Benzene} | 131.8 | 100.0% | 6.5% | 4.9% | 11 |
| {1,2,3-Trimethylbenzene,N-hexane,Toluene} | {Benzene} | 131.8 | 100.0% | 7.1% | 5.4% | 12 |
| {1,2,3-Trimethylbenzene,Ethylbenzene,Toluene} | {Benzene} | 131.8 | 100.0% | 8.2% | 6.2% | 14 |
| {1,2,3-Trimethylbenzene,Toluene,Xylene} | {Benzene} | 131.8 | 100.0% | 8.2% | 6.2% | 14 |
| {1,2,4-Trimethylbenzene,Cyclohexane,Pentane} | {Toluene} | 131.8 | 100.0% | 8.8% | 6.7% | 15 |
| {1,2,4-Trimethylbenzene,Cyclohexane,Pentane} | {Benzene} | 131.8 | 100.0% | 8.8% | 6.7% | 15 |
| {1,2,4-Trimethylbenzene,Cyclohexane,Heptane} | {Toluene} | 131.8 | 100.0% | 10.6% | 8.0% | 18 |
| {1,2,4-Trimethylbenzene,Cyclohexane,Heptane} | {Benzene} | 131.8 | 100.0% | 10.6% | 8.0% | 18 |
| {1,2,4-Trimethylbenzene,Cyclohexane,N-hexane} | {Toluene} | 131.8 | 100.0% | 10.0% | 7.6% | 17 |
| {1,2,4-Trimethylbenzene,Cyclohexane,N-hexane} | {Benzene} | 131.8 | 100.0% | 10.0% | 7.6% | 17 |
| {1,2,4-Trimethylbenzene,Cyclohexane,Ethylbenzene} | {Toluene} | 131.8 | 100.0% | 10.6% | 8.0% | 18 |
| {1,2,4-Trimethylbenzene,Cyclohexane,Ethylbenzene} | {Benzene} | 131.8 | 100.0% | 10.6% | 8.0% | 18 |
| {1,2,4-Trimethylbenzene,Cyclohexane,Xylene} | {Toluene} | 131.8 | 100.0% | 10.6% | 8.0% | 18 |
| {1,2,4-Trimethylbenzene,Cyclohexane,Xylene} | {Benzene} | 131.8 | 100.0% | 10.6% | 8.0% | 18 |
| {1,2,4-Trimethylbenzene,Cyclohexane,Toluene} | {Benzene} | 131.8 | 100.0% | 10.6% | 8.0% | 18 |
| {1,2,4-Trimethylbenzene,Heptane,Pentane} | {Toluene} | 131.8 | 100.0% | 10.6% | 8.0% | 18 |
| {1,2,4-Trimethylbenzene,Heptane,Pentane} | {Benzene} | 131.8 | 100.0% | 10.6% | 8.0% | 18 |
| {1,2,4-Trimethylbenzene,N-hexane,Pentane} | {Benzene} | 131.8 | 100.0% | 11.2% | 8.5% | 19 |
| {1,2,4-Trimethylbenzene,Ethylbenzene,Pentane} | {Benzene} | 131.8 | 100.0% | 12.9% | 9.8% | 22 |
| {1,2,4-Trimethylbenzene,Pentane,Xylene} | {Toluene} | 131.8 | 100.0% | 11.2% | 8.5% | 19 |
| {1,2,4-Trimethylbenzene,Pentane,Xylene} | {Benzene} | 131.8 | 100.0% | 11.2% | 8.5% | 19 |
| {1,2,4-Trimethylbenzene,Pentane,Toluene} | {Benzene} | 131.8 | 100.0% | 11.8% | 8.9% | 20 |
| {1,2,4-Trimethylbenzene,Heptane,N-hexane} | {Toluene} | 131.8 | 100.0% | 12.9% | 9.8% | 22 |
| {1,2,4-Trimethylbenzene,Heptane,N-hexane} | {Benzene} | 131.8 | 100.0% | 12.9% | 9.8% | 22 |
| {1,2,4-Trimethylbenzene,Heptane,Toluene} | {Benzene} | 131.8 | 100.0% | 14.7% | 11.2% | 25 |
| {1,2,4-Trimethylbenzene,Ethylbenzene,N-hexane} | {Benzene} | 131.8 | 100.0% | 14.7% | 11.2% | 25 |
| {1,2,4-Trimethylbenzene,N-hexane,Xylene} | {Toluene} | 131.8 | 100.0% | 14.1% | 10.7% | 24 |
| {1,2,4-Trimethylbenzene,N-hexane,Xylene} | {Benzene} | 131.8 | 100.0% | 14.1% | 10.7% | 24 |
| {1,2,4-Trimethylbenzene,N-hexane,Toluene} | {Benzene} | 131.8 | 100.0% | 14.7% | 11.2% | 25 |
| {Cyclohexane,Heptane,Pentane} | {Toluene} | 131.8 | 100.0% | 12.4% | 9.4% | 21 |
| {Cyclohexane,Heptane,Pentane} | {Benzene} | 131.8 | 100.0% | 12.4% | 9.4% | 21 |
| {Cyclohexane,N-hexane,Pentane} | {Toluene} | 131.8 | 100.0% | 12.4% | 9.4% | 21 |
| {Cyclohexane,N-hexane,Pentane} | {Benzene} | 131.8 | 100.0% | 12.4% | 9.4% | 21 |
| {Cyclohexane,Ethylbenzene,Pentane} | {Toluene} | 131.8 | 100.0% | 12.4% | 9.4% | 21 |
| {Cyclohexane,Pentane,Xylene} | {Toluene} | 131.8 | 100.0% | 12.9% | 9.8% | 22 |
| {Cyclohexane,Ethylbenzene,Heptane} | {Toluene} | 131.8 | 100.0% | 13.5% | 10.3% | 23 |
[truncated: 2,118,365 more chars]
